# Vitamin D intakes and health outcomes in infants and preschool children: Summary of an evidence report

**DOI:** 10.1080/07853890.2022.2111602

**Published:** 2022-08-17

**Authors:** Andrew R. Beauchesne, Kelly Copeland Cara, Danielle M. Krobath, Laura Paige Penkert, Shruti P. Shertukde, Danielle S. Cahoon, Belen Prado, Ruogu Li, Qisi Yao, Jing Huang, Tee Reh, Mei Chung

**Affiliations:** a School of Medicine, Tufts University, Boston, Massachusetts, USA; b Friedman School of Nutrition Science and Policy, Tufts University, Boston, Massachusetts, USA

**Keywords:** Vitamin D, nutritional requirements, infant, pre-school child, child development, bone density, asthma, autoimmune diseases, communicable diseases, systematic review

## Abstract

**Background:**

A systematic review was commissioned to support an international expert group charged to update the Food and Agriculture Organisation of the United Nations (FAO)/World Health Organisation (WHO)’s vitamin D intake recommendations for children aged 0–4 years.

**Materials and methods:**

Multiple electronic databases were searched to capture studies published from database inception to the 2^nd^ week of June 2020 according to key questions formulated by the FAO/WHO. Relevant studies were summarised and synthesised by key questions and by health outcomes using the Grades of Recommendation, Assessment, Development, and Evaluation (GRADE) approach.

**Results:**

The 146 included studies examined the effects of different vitamin D intake levels on a variety of health outcomes (e.g. infectious disease, growth, neurodevelopment, rickets, and bone mineral density), and on outcomes for setting vitamin D upper limits (e.g. hypercalcemia, hypercalciuria, and nephrocalcinosis). For most outcomes, the strength of evidence was *low* or *very low*. Evidence was rated *moderate* for the effect of daily vitamin D supplementation on raising serum 25(OH)D concentrations, and a random-effects meta-regression analysis of 28 randomised controlled trials (mostly in infants 0–12 months) showed that each 100 IU/d increase in vitamin D supplementation was associated with an average of 1.92 (95% CI 0.28, 3.56) nmol/L increase in achieved 25-hydroxy-vitaminn D (25[OH]D) concentration (*n* = 53 intervention arms; *p* = .022) with large residual heterogeneity (*I^2^* = 99.39%). Evidence was *very low* on two of the upper limit outcomes – hypercalcemia and hypercalciuria.

**Conclusions:**

The evidence report provided the expert group with a foundation and core set of data to begin their work to set vitamin D nutrient reference values. To move the field forward, future studies should use standardised 25(OH)D assay measurements and should examine the relationship between long-term vitamin D status and health outcomes.

**Key Messages**
- Results of a large complex systematic review suggest the current totality of evidence from trials and prospective observational studies do not reach sufficient certainty level to support a causal relationship between vitamin D intake and asthma, wheeze, eczema, infectious diseases, or rickets (most trials reported no rickets) in generally healthy infants and young children.
- In this systematic review, the only body of evidence that reached a *moderate* level of certainty was regarding the effect of daily vitamin D supplementation (vitamin D_3_ or D_2_ supplements to infants/children) on increasing serum 25(OH)D concentrations. However, currently there is no consensus on the definitions of vitamin D status, e.g. deficiency, insufficiency, sufficiency and toxicity, based on serum 25(OH)D concentrations.
- This systematic review provided an international expert group a foundation and core set of data through intake-response modelling to help set vitamin D nutrient reference values for infants and children up to 4 years of age.

## Introduction

### Background and objectives

The Food and Agriculture Organisation of the United Nations (FAO) and the World Health Organisation (WHO) established recommended vitamin and mineral intakes for all age groups in 2004 [1]. However, at that time, there was no consensus approach to making nutrient intake recommendations. There have been two more recent efforts to set nutrient reference values (NRVs) for vitamin D – the Dietary Reference Intake values (DRIs) issued by the Institute of Medicine (IOM) of the United States (U.S.) National Academies, and the dietary reference values set by the European Food Safety Authority (EFSA) [2,3]. In 2009, the U.S. Agency for Healthcare Quality and Research (AHRQ) commissioned an evidence report (i.e. a large complex systematic review with several linked key questions) on health outcomes related to vitamin D and calcium [4], and the evidence report was later used by the 2010 DRI committee to update vitamin D and calcium DRI values for all life stages [3]. The IOM’s DRI values are 400 IU per day (Adequate Intake [AI]) for both infants 0–6 months and 7–12 months of age, and 600 IU per day (Recommended Daily Allowance [RDA]) for children 1–4 years. AI is the average daily intake based on observed nutrient intake by a group (or groups) of apparently healthy people that are assumed to be adequate. AI is used when a recommended intake (such as RDA) cannot be determined due to insufficient data to establish an intake-response association between a nutrient and a physiological outcome. The Tolerable Upper Intake Level (UL) values are 1000 IU, 1500 IU, and 2500 IU per day for infants 0–6 months, infants 7–12 months, and children 1–4 years of age, respectively. UL values are not recommended intake levels; rather, they are the highest average daily nutrient intake that is likely to pose no risk of adverse health effects to almost all individuals in the general population. EFSA’s report, published in 2016, concluded that there was insufficient data to set an Average Requirement (the average daily nutrient intake that is estimated to meet the requirements of half of the healthy individuals in a particular life stage and gender group) for vitamin D, so instead set an AI for all population groups [2,5]. Both the 2009 AHRQ commissioned evidence report and the 2016 EFSA report identified a paucity of studies conducted on infants and children [4,5]. New data have emerged warranting a re-evaluation of vitamin D and calcium NRVs, particularly for young children. The results of the WHO-commissioned calcium systematic review have been published separately [6], so the remainder of this document is focussed on the vitamin D investigations.

**Figure 1.**
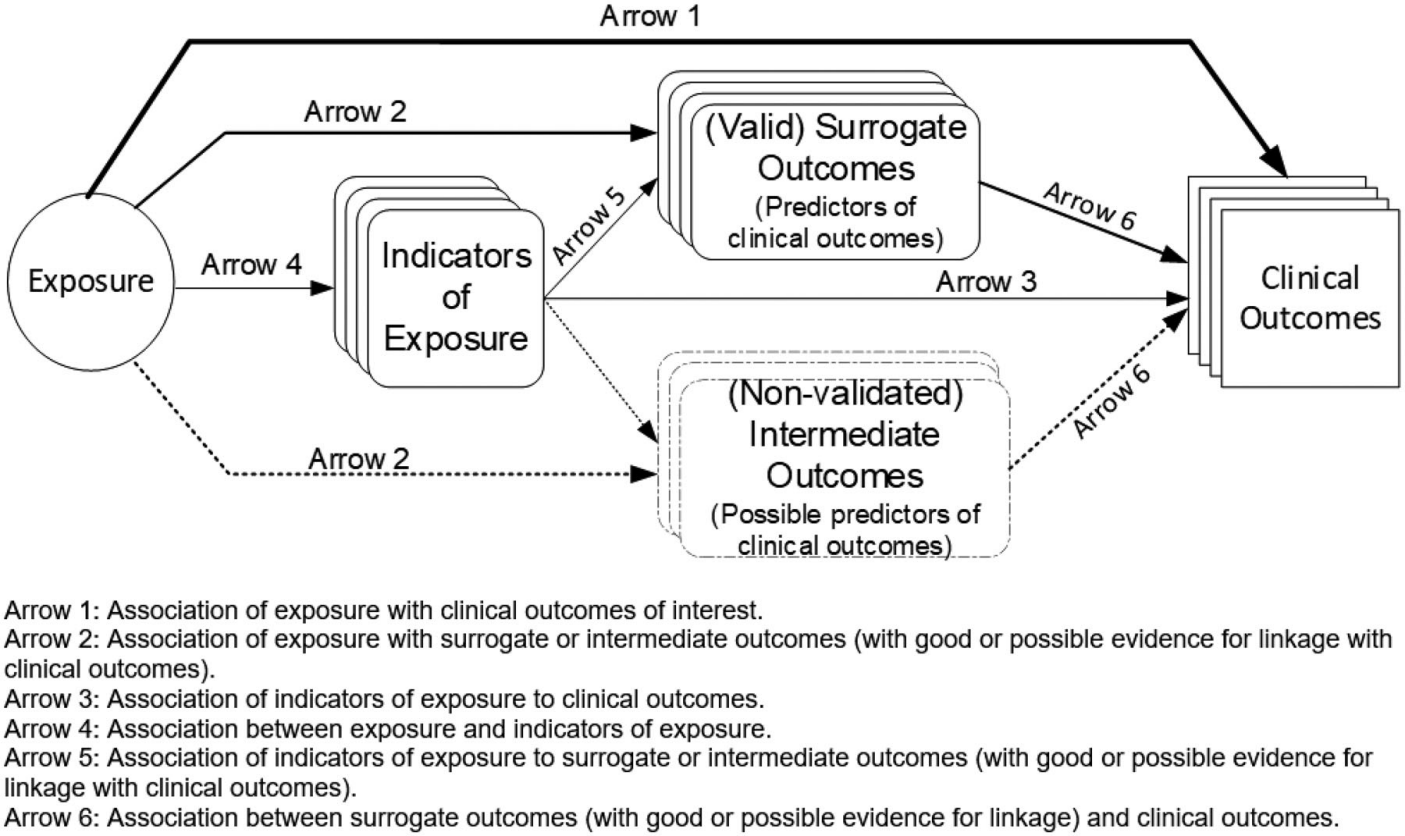
A generic analytic framework to assist the formulation of systematic review key questions for the development of nutrients reference intake values.

In 2017, the WHO and FAO, partnered with the U.S. National Academies of Science, Engineering, and Medicine (NASEM, formerly IOM), convened an international workshop with the goal of achieving global harmonisation of the methodological approaches used to derive NRVs across countries [7]. Followed by this effort, the WHO and FAO established an international expert group in 2019 to update nutrient intake recommendations for children aged 0–4 years [8]. Nutrients prioritised for the first round of updates include vitamin D and calcium, which are being investigated simultaneously due to the synergism of these two nutrients. During phase I of this work, the FAO/WHO commissioned a scoping review on vitamin D and calcium research reporting health outcomes in children 0–36 months [9]. In the scoping review, we found that dose-response randomised controlled trials (RCTs) that assessed the effects of vitamin D intake on age-specific clinical outcomes of public health importance were scarce. According to the generic analytic framework (Figure 1) [10], when evidence of the association between exposure and clinical outcomes of interest is lacking (Figure 1, Arrow 1), a “piecemeal approach” (also known as the “dose-response approach” [11]) that uses indicators of exposure (Arrow 4) and surrogate outcomes (Arrow 5) has been suggested as an option for setting NRVs [12]. Therefore, the FAO/WHO expert group determined that a dose-response approach would be appropriate for setting vitamin D requirements for the target age group. Based on results from the Phase I scoping review and other supporting documents, the expert group identified suitable indicators of exposure (e.g. serum 25-hydroxyvitamin D [25(OH)D] concentrations) and outcomes of interest (e.g. growth and development indices) and then formulated key questions (KQs) to guide an evidence report as indicated below.

#### Vitamin D requirements

- KQ 1. What is the effect of different levels of vitamin D intake on health outcomes in children aged 0–4 years?
- KQ 2. What is the association between serum 25(OH)D concentrations and health outcomes in children aged 0–4 years?
- KQ 3. What is the effect of vitamin D intake on serum 25(OH)D concentrations in children aged 0–4 years?

#### Vitamin D tolerable upper intake level (UL)

- KQ UL1a. At what levels of vitamin D intake are adverse effects observed in children aged 0–4 years?
- KQ UL1b. What are levels of vitamin D intake at which a prespecified threshold of serum 25(OH)D is reached in children aged 0–4 years?

The resulting evidence report was provided to the expert group and is summarised below. The full report is presented in the Supplemental File.

### Scope and objectives

The overall objective of this evidence report was to synthesise all available evidence that met predefined eligibility criteria to help support an FAO/WHO expert group charged with updating the vitamin D NRVs for children aged 0–4 years [8]. The evidence report was focussed on indicators of vitamin D exposure including dietary intake (e.g. vitamin D_2_ and D_3_), sunlight or ultraviolet-B exposure (or it is proxy such as latitude), and 25(OH)D concentrations, as well as a range of important health outcomes for the target populations, including growth and development (e.g. anthropometric indices, failure to thrive, etc.), neurological development, infectious disease, autoimmune disease, asthma, wheezing, atopic dermatitis, fracture, bone mineral density, bone mineral content, rickets, blood pressure, and calcium absorption and retention. The following specific objectives were based on the expert group KQs and were focussed on children aged 0–4 years.

- Identify the effect of different levels of vitamin D intake on pre-defined health outcomes (KQ1), serum 25(OH)D concentrations (KQ3), and reported adverse effects (KQ UL1a).
- Determine the association between serum 25(OH)D concentrations and pre-defined health outcomes (KQ2).
- Identify levels of vitamin D intake needed to reach prespecified thresholds of serum 25(OH)D (KQ UL1b).

## Materials and methods

We followed the methods for conducting a systematic review outlined in the Institute of Medicine’s Standards for Systematic Reviews [13] and reported the results according to the Preferred Reporting Items for Systematic Reviews and Meta-Analyses (PRISMA) statement [14]. A complete description of the methods, including planned subgroup analyses and a full description of study eligibility criteria for the KQs, appears in the full evidence report presented in the Supplemental File. A prospectively developed protocol was published in The International Prospective Register of Systematic Reviews, PROSPERO (https://www.crd.york.ac.uk/prospero/; CRD42020198843).

### Literature search strategy and study selection process

Database searches were conducted in MEDLINE®, Embase, and Cochrane Central databases to capture studies from the inception of each database to the 2^nd^ week of June 2020. Searches were not restricted by language or publication date. Search strategies were developed according to the KQs and are shown in Supplemental Appendix A. The same search strategy was used to identify articles relevant for KQs regarding calcium requirements and upper limits formulated by the FAO/WHO expert panel; however, only studies meeting the criteria for the vitamin D KQs are summarised herein. Study investigators rescreened both the excluded and included full-text articles from the Phase I scoping review [9] using the systematic review study eligibility criteria presented in Tables 1–4. In addition to database searches, relevant authoritative reports and systematic reviews were used for reference mining.

**Table 1.** Vitamin D requirements key question 1 (KQ1) eligibility criteria.

Category	Inclusion criteria	Exclusion criteria
Study designs of interest	Randomised (paralleled or crossover) controlled trials, or nonrandomized controlled trials Intervention duration ≥2 weeks	*In vitro* (cell) and animal studies Observational studies [Note: Dietary assessments of vitamin D intake levels were not included due to inadequacy of nutrient composition tables for vitamin D [15]] Single-arm trials Studies that used non-concurrent cohorts or non-concurrent controls Unpublished studies (e.g. conference abstracts, posters)
Populations of interest	Generally healthy^a^ children 0–4 years old	Critically ill children admitted to intensive care unit Studies that enrolled exclusively premature infants (≤32 weeks gestational age) or very low birth weight infants (≤1500 grams) Studies conducted exclusively in children with moderate or severe acute malnutrition (MAM/SAM)
Interventions of interest	Dietary vitamin D intake (with or without calcium) from foods or supplements UV exposure to manipulate 25(OH)D levels	Non-oral intake of vitamin D such as injections or peripheral parenteral nutrition Intervention studies in which effects of vitamin D and/or calcium cannot be isolated Vitamin D analogs (e.g. calcifedio, calcijex, calcipotriol, calcitriol, doxercalciferol, hectorol, paricalcitrol, rayaldee, rocaltrol, zemplar)
Comparators of interest	Any	None
Outcomes of interest	Growth and development (anthropometric indices, failure to thrive, etc.)^b^ Neurological development^c^ Infectious disease Autoimmune disease Asthma, wheezing, or atopic dermatitis Fracture Bone mineral density or bone mineral content (irrespective of the method employed, for example, ultrasonography, DEXA etc.) Rickets (including “nutritional rickets”) Blood pressure Calcium absorption and retention^d^	Maternal health-related outcomes Any outcome measured only at birth in mothers or in infants Lead concentration Health-service utilisation outcomes

DEXA = Dual-energy X-ray absorptiometry; MAM = moderate acute malnutrition; SAM = severe acute malnutrition; UV = ultraviolet.

^a^“Generally healthy” populations are defined as having ≤20% of the study population with disease at the study’s baseline with the exception of the case-control study design. Nutrition deficiencies, overweight, and obesity are not considered diseases in this systematic review.

^b^For growth and development outcomes, the populations of interest are expanded to include children 0–9 years old because growth and development outcomes are also considered outcomes of interest for vitamin D and calcium ULs. All anthropometric measures are considered outcomes of interest, such as height, weight, length/height for age, weight for age, weight for height/length, BMI, related z-scores, waist circumference, mid-arm circumference (MUAC), skinfold thickness, head circumference.

^c^Autism is not an outcome of interest, but cognitive or intellectual development assessed by IQ is of interest.

^d^For the calcium absorption and retention outcomes, the minimal intervention duration of 2 weeks criterion does not apply because calcium absorption is also an outcome of interest for calcium requirements.

**Table 2.** Vitamin D requirements key question 2 (KQ2) eligibility criteria.

Category	Inclusion criteria	Exclusion criteria
Study designs of interest	Cohort, nested case-control, or case-cohort studies in which 25(OH)D concentrations were measured before outcome ascertainment. Follow-up duration ≥2 weeks	*In vitro* (cell) and animal studies Intervention studies Cross-sectional studies reporting only prevalence data (i.e. no correlation or association analyses) Retrospective case-control studies Case reports or case series
Populations of interest	Generally healthy^a^ children 0–4 years old	Critically ill children admitted to intensive care unit Studies that enrolled exclusively premature infants (≤32 weeks gestational age) or very low birth weight infants (≤1500 grams) Studies conducted exclusively in children with moderate or severe acute malnutrition (MAM/SAM)
Exposures of interest	25(OH)D concentrations (irrespective of measurement assay)	Dietary assessments of vitamin D intake only [Note: Dietary assessments of vitamin D intake levels were not included due to inadequacy of nutrient composition tables for vitamin D [15]]
Comparators of interest	Different levels of 25(OH)D concentrations	None
Outcomes of interest	Growth and development (anthropometric indices, failure to thrive, etc.)^b^ Neurological development^c^ Infectious disease Autoimmune disease Asthma, wheezing, or atopic dermatitis Fracture Bone mineral density or bone mineral content (irrespective of the method employed, for example, ultrasonography, DEXA etc.) Rickets (including “nutritional rickets”) Blood pressure Calcium absorption and retention^d^	Maternal health-related outcomes Any outcome measured only at birth in mothers or in infants Lead concentration Health-service utilisation outcomes

DEXA = Dual-energy X-ray absorptiometry; MAM = moderate acute malnutrition; SAM = severe acute malnutrition.

^a^“Generally healthy” populations are defined as having ≤20% of the study population with disease at the study’s baseline with the exception of the case-control study design. Nutrition deficiencies, overweight, and obesity are not considered diseases in this systematic review.

^b^For growth and development outcomes, the populations of interest are expanded to include children 0–9 years old because growth and development outcomes are also considered outcomes of interest for vitamin D and calcium ULs. All anthropometric measures are considered outcomes of interest, such as height, weight, length/height for age, weight for age, weight for height/length, BMI, related z-scores, waist circumference, mid-arm circumference (MUAC), skinfold thickness, head circumference.

^c^Autism is not an outcome of interest.

^d^For the calcium absorption and retention outcomes, the minimal follow-up duration of 2 weeks criterion does not apply because calcium absorption is also an outcome of interest for calcium requirements.

**Table 3.** Vitamin D requirements key question 3 (KQ3) and vitamin D upper limits key question 1 b (KQ UL1b) eligibility criteria.

Category	Inclusion criteria	Exclusion criteria
Study designs of interest	Randomised (paralleled or crossover) controlled trials, or nonrandomized controlled trials Intervention duration ≥2 weeks	*In vitro* (cell) and animal studies Observational studies Single-arm trials Studies that used non-concurrent cohorts or non-concurrent controls Unpublished studies (e.g. conference abstracts, posters)
Populations of interest	Generally healthy children 0–9 years old^a^	Critically ill children admitted to intensive care unit Studies that enrolled exclusively premature infants (≤32 weeks gestational age) or very low birth weight infants (≤1500 grams) Studies conducted exclusively in children with moderate or severe acute malnutrition (MAM/SAM)
Interventions of interest	Dietary vitamin D intake (with or without calcium) from foods or supplements	Non-oral intake of vitamin D such as injections or peripheral parenteral nutrition Intervention studies in which effects of vitamin D and/or calcium cannot be isolated Vitamin D analogs
Comparators of interest	Any	None
Outcomes of interest	25(OH)D concentrations (irrespective of measurement assay)	None

MAM = moderate acute malnutrition; SAM = severe acute malnutrition.

^a^“Generally healthy” populations are defined as having ≤20% of the study population with disease at the study’s baseline with the exception of the case-control study design. Nutrition deficiencies, overweight, and obesity are not considered diseases in this systematic review. For KQ3 and KQ UL 1 b, the populations of interest were expanded to include children 4–9 years old.

**Table 4.** Vitamin D upper limits key question 1a (KQ UL1a) eligibility criteria.

Category	Inclusion criteria	Exclusion criteria
Study designs of interest	Intervention studies of any design Observational studies of any design Case reports of excess vitamin intake (as defined in the original studies)	*In vitro* (cell) and animal studies Unpublished studies (e.g. conference abstracts, posters)
Populations of interest	Generally healthy children 0–9 years old^a^	Critically ill children admitted to intensive care unit Studies that enrolled exclusively premature infants (≤32 weeks gestational age) or very low birth weight infants (≤1500 grams) Studies conducted exclusively in children with moderate or severe acute malnutrition (MAM/SAM)
Interventions or exposures of interest	Intervention studies: Dietary vitamin D intake (with or without calcium) from foods or supplements Observational studies: 25(OH)D concentrations (irrespective of measurement assay)	Non-oral intake of calcium and/or vitamin D such as injections or peripheral parenteral nutrition Intervention studies in which effects of vitamin D and/or calcium cannot be isolated Vitamin D analogs
Comparators of interest	Any	None
Outcomes of interest	Growth and development^b^ Hypercalcaemia Hypercalciuria Kidney stones Nephrocalcinosis All-cause mortality	None

MAM = moderate acute malnutrition; SAM = severe acute malnutrition.

^a^“Generally healthy” populations are defined as having ≤20% of the study population with disease at the study’s baseline with the exception of the case-control study design. Nutrition deficiencies, overweight, and obesity are not considered diseases in this systematic review. For KQ UL 1a, the populations of interest were expanded to include children 4–9 years old.

^b^Any definition for categorical growth and development outcomes associated with high levels of vitamin D intake or 25(OH)D concentrations, such as overweight or obesity (usually defined by BMI cut-off).

After duplicate citations were removed, titles and abstracts were screened by two independent investigators using Rayyan abstract screening software [16]. Relevant full-text articles were screened by one investigator according to the study eligibility criteria (Tables 1–4), and rejected articles were reviewed by a second investigator to confirm exclusion. Disagreements were adjudicated by a third investigator or group consensus. A list of excluded studies and exclusion reasons are documented in Supplemental Appendix B.

### Data extraction

Standardised forms were created to extract individual study data regarding study characteristics (type of study [controlled trial, prospective cohort, nested case-control, case-cohort], design [parallel/crossover; randomised/non-randomised], study arms [intervention/exposure; control/comparator], duration, outcomes), population characteristics (mean age, percent male, race or ethnicity, country, health status, baseline diet, breastfeeding status), data required for planned subgroup analyses (level of vitamin D intake, source of vitamin D, supplement formulation, sun exposure and/or latitude, age, breastfeeding status, race/ethnicity, skin colour), and results for all outcomes of interest (see Tables 1–4 for the complete list of outcomes). Data were extracted by one investigator and spot-checked by a second investigator.

### Risk of bias assessment

Two independent investigators performed a risk of bias (ROB) assessment for each included study outcome with disagreements resolved *via* discussion between the two investigators. Cochrane Collaboration’s tool (ROB 2.0) [17] was used to assess ROB for interventional studies. An overall ROB rating (high, medium [some concerns], or low risk for biases) was established for each interventional study using the Cochrane overall risk-of-bias criteria [17]. The Newcastle Ottawa Scale (NOS) was used to assess ROB for cohort, case-cohort, and nested case-control studies [18]. Modifications to the NOS were made, including the tailoring, addition, and removal of prompting questions, to better suit the needs of the review and to assess ROB for case-cohort and nested case-control study designs.

### Data synthesis and strength of evidence rating

Results for each study were reported in separate summary tables for each KQ and outcome. Where possible, narrative reporting and analyses were divided into age subgroups (e.g. 0–12 months and 1–4 years) to account for differences in vitamin D source (e.g. primarily human or formula milk in infants versus solid foods in young children) and outcomes. The Grades of Recommendation, Assessment, Development, and Evaluation (GRADE) approach [19,20] was used to determine the strength of evidence (high, medium, low, very low, or insufficient) for each outcome. GRADE evidence profile tables [21] were used to present synthesised data for each KQ.

#### Meta-analysis

For vitamin D requirement KQ 3 (What is the effect of vitamin D intake on serum 25(OH)D concentrations in children aged 0–4 years?), we performed a random-effects meta-regression [22,23] to examine the intake-response associations across studies. No meta-analyses were performed for all other KQs due to large heterogeneity in exposure and outcome definitions or ascertainment methods across included studies.

## Results

**Figure 2.**
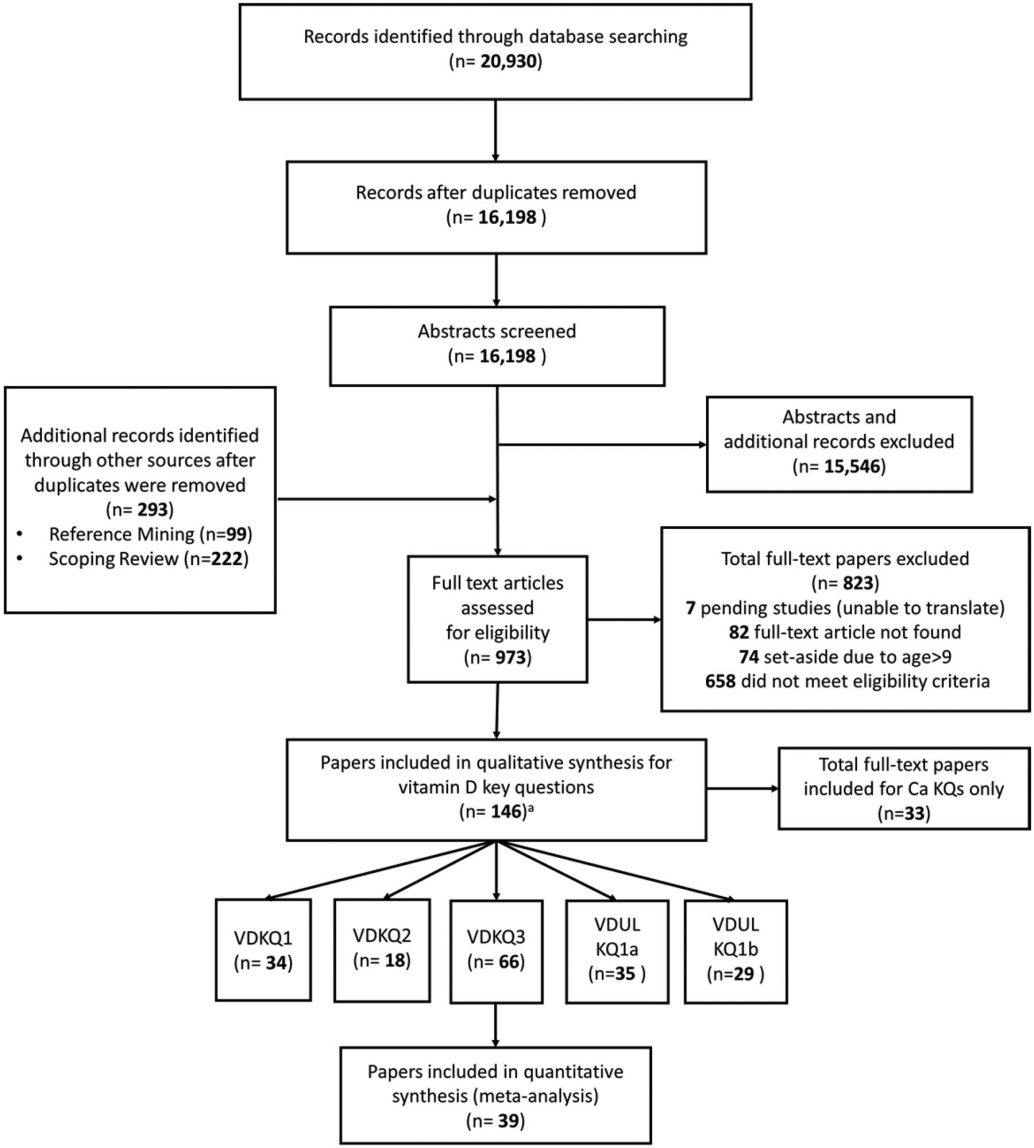
Literature search and study selection process. Legend: VDKQ = vitamin D requirement key question; VDUL = vitamin D upper limits. ^a^The sum of papers for listed key questions is greater than 146, as some papers were included in more than one key question.

Altogether, 146 publications were included in this systematic review (see Appendix B in the Supplemental File for bibliography). This comprised 34 randomised and non-randomised controlled trials (RCTs and non-RCTs) on the effect of different vitamin D intake levels on health outcomes in children aged 0–4 years (KQ1). There were 18 observational studies included that examined the association between serum 25(OH)D concentrations and health outcomes in children aged 0–4 years (KQ2). A total of 65 unique RCTs (in 66 reports) on the effect of vitamin D on serum 25(OH)D concentrations in children aged 0–9 years were included for KQ3. Lastly, 64 studies (any study design including case reports) reporting the effect of vitamin D on upper limit outcomes were also included in the review. A flow chart summarising our literature search and study selection process is shown in Figure 2.

Strength of evidence assessments was completed using the GRADE approach, and an evidence profile table organised by KQs and by outcomes is provided in Table 5. Evidence for the outcomes that were rated as *insufficient* was not included in the evidence profile table. Below is an overview of findings for all KQs, but additional findings, including detailed results, summary tables for all included studies, and risk of bias assessments, are provided in the full evidence report (see Supplemental File).

**Table 5.** GRADE evidence profile table: vitamin D requirements and upper limits.

Quality assessment							Summary of findings	Strength of evidence
No. of studies	Design	Limitations	Inconsistency	Indirectness	Imprecision	Dose-response		
**KQ1. Atopic outcomes: asthma, wheeze, eczema**								
4	RCTs	**Some limitations**: 100% of trials have some or high ROB in at least 1 ROB domain, and 50% of trials have high or some ROB in 3 domains.	**No serious inconsistency**: Most trials reported no significant differences in atopic outcomes comparing higher to lower doses of Vit D supplementation.	**Direct**: Clinical outcomes.	**Imprecise**: Small number of events with large confidence intervals.	No dose-response is present.	**Asthma:** 3 RCTs reported mixed results comparing higher to lower doses of VD supplementation. Two RCTs found no significant differences between groups. One RCT found participants who received 400 IU/d of vitamin D3 were at lower risk of developing asthma at 6 months, compared to those who received placebo (R*R* = 0.055; 95% CI 0.003, 0.94), but there were no significant differences in the risk of asthma when comparing 800 IU/d of vitamin D_3_ to placebo, or when comparing 800 IU/d to 400 IU/d of vitamin D_3_ supplementation [24]. **Wheeze:** 2 RCTs reported mixed results. One study reported no significant findings [25], but another RCT in preterm Black infants, found significantly reduced risk of recurrent wheezing at 12 months with sustained vitamin D supplementation compared to diet-limited supplementation (adjusted R*R* = 0.62; 95% CI 0.44, 0.87; *p* = .005) [26]. **Eczema:** 3 RCTs found no significant differences between groups.	Low
**KQ1. Infectious diseases**								
8	RCTs	**Some limitations**: 88% of trials have some or high ROB in at least 2 ROB domains. The other trial (12%) has high risk in one ROB domain.	**No serious inconsistency**: Most trials reported no significant differences in infectious disease outcomes comparing higher to lower doses of vitamin D supplementation	**Direct**: Clinical outcomes.	**Imprecise**: Studies reported variable effect measures with large confidence intervals.	No dose-response is present.	Out of the 20 infectious disease outcomes (respiratory, *n* = 15; gastrointestinal, *n* = 1; and other or unspecified infections, *n* = 4), 19 were not significantly different between intervention groups. One RCT found participants who received 1,200 IU/d of vitamin D_3_ were significantly less likely to develop influenza A after 4 months compared to those receiving 400 IU/d of vitamin D_3_ (R*R* = 0.54; 95% CI 0.42, 0.77) [27].	Low
**KQ1. Growth and neurodevelopment**								
13	RCT (1 study with a non-randomised control group [28])	**Serious limitations:** All trials have some concern or high ROB in at least 2 domains, and 85% had high ROB for de*via*tions from intended intervention. For all ROB domains, ≥ 30% of studies had some concern or high ROB.	**Consistent:** 85% of studies showed no significant association between VD intervention and growth outcomes. Only 2 studies reported on neurological development outcomes.	**Direct:** Clinical outcome.	**Some imprecision:** Most studies reported small confidence intervals, but studies were not powered for development outcomes.	No dose-response is present.	Eleven RCTs reported no association between VD interventions and growth and development outcomes when comparing higher to lower doses or when comparing VD supplementation to a placebo.	Low
**KQ1. Rickets**								
9	RCT, non-RCT	**Serious limitations:** All trials have some concern or high ROB in at least 3 domains. For all ROB domains, > 50% of studies had some concern or high ROB.	**Consistent:** 89% of trials reported no rickets, and 11% reported no significant association between rickets and VD supplements.	**Direct:** Clinical outcome.	**Imprecise:** Very small number of events.	No dose-response is present.	Eight RCTs reported no rickets. One non-RCT reported rickets i*n* < 2% of the study population, and while there was no association with study arm (calcium, vitamin D, or calcium plus vitamin D supplementation) (*p* = .214), there was a significant interaction between time and supplementation over the three-year study period (*p* = .001) [29].	Low
**KQ1. Bone mineral content and bone mineral density (BMC/BMD)**								
10	RCT (1 study with a non-randomised control group [28]), non-randomised controlled trial	**Serious limitations:** All trials have some or high ROB in at least 1 ROB domain. For each ROB domain, except for measurement of outcome, at least 50% of studies have some or high ROB.	**Some inconsistency:** 50% of the studies reported no association between VD interventions and BMC/BMD outcomes. 30% reported benefits of VD supplementation vs. placebo for BMC/BMD outcomes (.05 < *p* < .1, or p-values and 95% CI not reported). 20% reported significant associations between higher vs. lower VD doses or VD supplementation vs. breast milk alone and BMC/BMD outcomes.	**Indirect:** Surrogate outcome.	**Some imprecision:** 50% of studies with BMC/BMD as primary outcomes; 70% of studies with small sample sizes per study group; Mostly narrow confidence intervals for BMD/BMC outcomes.	Dose-response is present.	There were mixed results for BMC/BMD outcomes. Five RCTs from six publications reported no difference in BMC/BMD outcomes between any study groups [30–35]. Two studies reported benefits to BMC/BMD outcomes for VD supplementation vs. placebo but did not report *p*-values or confidence intervals, and one included a non-randomised comparison group [26,28]. Two studies reported statistically significant (*p* < .05) benefits for various BMC/BMD measures when comparing randomised groups with higher vs. lower doses of VD (1,600 IU/d vs. 400 IU/d; 1,600 IU/d vs. 1,200 IU/d) [36] or when comparing non-randomised groups with VD supplementation or breast milk alone [37]. One study reported moderately significant (.05 < *p* < .1) benefits for one bone measurement when comparing VD supplementation to a placebo [38].	Low
**KQ2. Atopic outcomes: asthma, wheezing, and eczema**								
4	Cohorts, case-cohorts	**Serious limitations**: 63% of outcomes of interest were not demonstrated to be absent at start of study, 75% of outcomes were assessed by self-report, and 75% of outcomes had significant lost to follow-up	**No serious inconsistency**: Most studies reported no significant association between serum 25(OH)D and risk of atopic outcomes.	**Direct**: Clinical outcomes.	**Imprecise**: Wide confidence intervals.	No dose-response is present.	**Asthma:** Three cohort studies had mixed results measuring the association between serum 25(OH)D and asthma outcomes. Two cohort studies found no association. A third cohort study found participants with higher numbers of follow-up visits with deficient serum 25(OH)D had significantly increased risk of asthma, but not medicated asthma [39]. **Wheeze:** Two cohort studies had mixed results measuring serum 25(OH)D and wheeze outcomes. One study found no association, while the other found participants with higher numbers of follow-up visits with deficient serum 25(OH)D had significantly increased risk of wheeze [39]. **Eczema:** Two studies had mixed results measuring serum 25(OH)D and eczema outcomes. One case-cohort study found no association, while the other cohort found participants with higher numbers of follow-up visits with deficient serum 25(OH)D had significantly increased risk of eczema [39].	Very low
**KQ2. Autoimmune diseases**								
7	Case-cohorts, nested case-controls	**Serious limitations**: 57% of studies with significant lost to follow-up or no statement, 71% not selecting all cases, and 71% using non-optimal or poorly described analytic methods	**No serious inconsistency**: Most studies reported no significant association between serum 25(OH)D and risk of autoimmune disease outcomes	**No serious indirectness**: Clinical outcomes or immediate precursor to clinical outcome (e.g. islet autoimmunity)	**Imprecise**: Most studies with wide confidence intervals or large measures of variability.	No dose-response is present.	**Type 1 diabetes:** Four observational studies found no association between serum vitamin D and type 1 diabetes. **Islet autoimmunity:** Two observational studies reported mixed results. One case-cohort found no association between serum 25(OH)D and islet autoimmunity [40]. One nested case-control study found an association between serum 25(OH)D (in the first year of life and in childhood) and decreased risk of islet autoimmunity [41]. **Juvenile idiopathic arthritis (JIA):** One case-cohort study found no association between serum 25(OH)D and oligoarticular and polyarticular JIA [42].	Very low
**KQ2. Infectious diseases**								
4	Cohorts	**Some limitations**: One study reporting one outcome (14% of outcomes) had major limitations: outcome assessed *via* self-report, outcome not demonstrated to be absent at start of study, analysis not optimally controlled, and poor adequacy of cohort follow-up	**Some inconsistency**: Most studies found no significant association or an association between serum 25(OH)D and decreased risk of infection, with one study reporting increased risk for one infectious disease outcome (oral candidiasis)	**Direct**: Clinical outcomes.	**Imprecise**: Most studies with wide confidence intervals or large measures of variability.	No dose-response is present.	Most associations between serum 25(OH)D and infectious disease outcomes were not significant. Significant associations were found for three of eight total infectious disease outcomes, with higher serum 25(OH)D associated with a reduced risk of oral candidiasis [43] but an increased risk for URTI (in underweight children) and malaria infection (between highest and second highest quartiles of serum 25OHD) [43,44].	Very low
**KQ2. Growth and neurological development**								
6	Cohort (*n* = 4), nested case-control (*n* = 2)	**Some limitations**: 83% of studies had ROB in at least 1 domain, and 50% had ROB in two or more domains. 50% reported high loss to follow-up rates or gave no statement.	**Consistent:** 100% of studies reported no significant linear association between 25(OH)D and growth and development outcomes.	**Direct:** Clinical outcome.	**Some imprecision:** Power calculations not reported for most studies, but most had large sample sizes; studies reported wide Cis or did not report Cis.	Dose response, but relationship with growth and development and neurological development appears to be non-linear.	In 6 observational studies assessing 25(OH)D levels and growth and development or neurological development outcomes, no linear association was found between 25(OH)D in infancy and any development outcomes. Categorical 25(OH)D analyses showed some statistically significant benefits in development outcomes with higher 25(OH)D levels compared to the lowest levels.	Low
**KQ3. Daily vitamin D supplementation on serum 25(OH)D**								
39	RCTs	**Some limitations:** In 4 of 5 ROB domains, greater than 50% of trials were assessed as having some or high ROB.	**No serious inconsistency:** Consistency in direction but some inconsistency in magnitude of the achieved 25(OH)D concentration at the end of the intervention period.	**Indirect**: serum 25(OH)D, a marker of vitamin D status.	**Some imprecision:** Meta-regression analysis demonstrated wide Cis. Also, the residual heterogeneity in meta-regressions was large.	Dose-response is present within most studies comparing different levels of daily vitamin D supplementation.	In infants 0–12 months old, random-effects meta-regression analysis showed that each 100 IU/d increase in vitamin D supplementation was associated with an average of 1.92 (95% CI 0.28, 3.56) nmol/L increase in achieved 25(OH)D concentration (*n* = 53 intervention arms; *p* = .022; adjusted *R*^2^ = 9.07%). Only one study was in infants 1–4 years, which showed serum 25(OH)D unchanged in the 400 IU/d group but significantly increased from 89.6 to 121.6 nmol/L in the 2000 IU/d group after 16 weeks. In children 3–9 years old, random-effects meta-regression showed that each 100 IU/d increase in vit D supplementation was associated with an average of 2.49 (95% CI −0.24, 5.22) nmol/L increase in achieved 25(OH)D concentration (*n* = 16 intervention arms; *p* = .071; adjusted *R*^2^ = 19.96%).	Moderate
**KQ3. Non-daily vitamin D supplementation on serum 25(OH)D**								
11	RCTs	**Some limitations:** In 4 of 5 ROB domains, approximately 50% or more trials were assessed as having some or high ROB.	**Some inconsistency:** Consistency of direction but some inconsistency in magnitude of the achieved 25(OH)D concentration.	**Indirect**: serum 25(OH)D, a marker of vitamin D status.	**Some Imprecision:** Wide Cis within some trial arms.	Dose-response is present within most studies comparing different levels of vitamin D supplementation.	Single doses of 200,000 IU of vitamin D_3_ increased serum 25(OH)D to 317 nmol/L at one week and 246 nmol/L at 5 weeks in one study [45], and 150 nmol/L at 2 weeks in another study [46]. A single dose of 100,000 IU of vitamin D_3_ resulted in serum 25(OH)D levels of 92 at 2 weeks [46]. Single doses of 50,000 IU of vitamin D_3_ resulted in serum 25(OH)D levels of 154 and 62 nmol/L at 1.5 and 14 weeks in one study [47], and 85 and 91 nmol/L at 8 and 14 weeks in the other study [48]. Single doses of 300,000 and 600,000 IU resulted in serum 25(OH)D levels of 16.1 and 17.6 nmol/L, respectively, after 12 weeks [49]. Other dose regimens, including weekly or monthly doses of vitamin D, resulted in increased 25(OH)D. 14,000 IU of vitamin D_3_ weekly to resulted in mean 25(OH)D increased to 91.8 nmol/L in the vitamin D_3_ supplementation [50].	Low
**KQ3. Supplementation to post-partum mothers on infant serum 25(OH)D**								
4	RCTs	**Some limitations:** In 4 of 5 ROB domains, at least 50% of trials were assessed as having some or high ROB.	**Serious inconsistency:** Studies reported differences in magnitude and direction of effect across maternal supplementation arms.	**Indirect**: serum 25(OH)D, a marker of vitamin D status.	**Some Imprecision:** Some studies with wide Cis or small sample sizes.	Dose-response unable to assess.	Breastfed infant serum 25(OH)D had decreased in one maternal 1000 IU/d supplementation group in one trial [51]. Baseline serum 25(OH)D was not provided in the other tree trials; however, maternal supplementation of 400 IU daily resulted in higher infant serum 25(OH)D compared to placebo at 14 weeks in one study [52], and no significant difference between maternal 6400 IU/d and infant 300 IU/d (with maternal 400 IU/d) supplementation in another study [53]. In the last trial, maternal supplementation of 1000 IU daily, but not 2000 IU daily, resulted in infant serum 25(OH)D significantly lower than that of infants receiving direct 400 IU vitamin D_2_ daily at 8 weeks. This difference was also significant at 15 weeks, but differences between the other groups were not significant [54].	Very low
**KQ3. Food interventions containing vitamin D on serum 25(OH)D**								
3	RCTs	**Some limitations:** 100% of studies had some or high ROB in 2 ROB domains.	**Some inconsistency:** Studies reported differences in the effect magnitude despite food intervention arms containing similar amounts of vitamin D.	**Indirect**: serum 25(OH)D, a marker of vitamin D status.	**Some imprecision:** Some studies with wide Cis or small sample sizes.	Dose-response unable to assess.	Serum 25(OH)D decreased in groups receiving both fortified (with mean vitamin D dose of 466–486 IU/d) and non-fortified food, although none of the changes were significant in one study [55]. In a second study, fortified formula (400 IU/L) saw no significant increase in serum 25(OH)D [33]. The last trial reported significant increases in 25(OH)D after 12 weeks of food fortified with 1,000 IU daily and 400 IU daily in both fair- and dark-skinned children, but no significant increase in the groups receiving 80 IU daily in food [56].	Very low
**KQ3. Combined vitamin D and calcium supplementation on infant serum 25(OH)D**								
3	RCTs	**Some limitations:** 100% of studies had some or high ROB in 3 ROB domains.	**No serious inconsistency:** Direction of effect consistent; unable to assess consistency of effect magnitude.	**Indirect**: serum 25(OH)D, a marker of vitamin D status.	**Some imprecision:** Studies with wide Cis.	Dose-response unable to assess.	In one study, serum 25(OH)D levels increased significantly more in the 200 IU/d of vitamin D_3_ plus 700 mg/d of calcium supplementation group compared to the calcium only group after 12 weeks (+12.7 nmol/L [5.09 ng/mL]; 95% CI 1.3, 24.1) [57]. In another study, mean serum 25(OH)D levels did not significantly differ between groups that received 30,000 IU once monthly of vitamin D_3_ plus either 405 mg or 156 mg of calcium 5 times weekly after 48 weeks; however, both groups resulted in significantly higher 25(OH)D at the end of the study [58]. In the last study where both groups got 50 mg/kg/d of calcium supplementation, there was no significant difference in mean serum 25(OH)D levels at 48 weeks between the 30,000 IU once weekly group and the 4,000 IU/d group.	Very low
**UL KQ1a. Adverse effects: hypercalcemia, hypercalciuria, nephrocalcinosis, mortality, and kidney stones**								
47	RCTs, single-arm interventions, cohorts, case-cohorts, nested case-controls, cross-sectional studies, and case reports	**Some limitations**: 100% of outcomes from RCTs have high or some concerns in at least 2 ROB domains	**Some inconsistency**: Studies showed consistency among hypercalcemia outcome, but inconsistency among hypercalciuria outcome. Other upper limit outcomes were unable to be assessed due to few data (i.e. mortality, nephrocalcinosis).	**Direct**: Clinical outcomes.	**Imprecise**: Rates of upper limit outcomes are variable across studies, even among groups with similar dose and follow-up durations.	Dose-response is present within some studies assessing hypercalcemia and hypercalciuria	**Hypercalcemia:** Generally, the rate of hypercalcemia increased with the dose of vitamin D administered; however, the rate of hypercalcemia was variable, even comparing the same or similar intervention dose and durations. **Hypercalciuria:** The rate of hypercalciuria was variable among studies and interventions arms. **Other upper limit outcomes:** few high-quality studies reported on nephrocalcinosis, kidney stones, and mortality.	Very low

BMC = bone mineral content; BMD = bone mineral density; CI = confidence interval; d = day; GRADE = Grading of Recommendations, Assessment, Development and Evaluations; IU = international units; KQ = key question; non-RCT = non-randomised controlled trial; RCT = randomised controlled trial; ROB = risk of bias; UL = upper limit; URTI = upper respiratory tract infection; VD = Vitamin D.

### Vitamin D requirements

#### KQ1. What is the effect of different levels of vitamin D intake on health outcomes in children aged 0 to 4 years?

No trials reported on autoimmune disease or fracture outcomes, and only one randomised controlled trial (RCT) reported on blood pressure outcomes [38] resulting in an *insufficient* evidence rating for these outcomes. Evidence was *low* for the effect of different levels of vitamin D intake on several health outcomes including atopic outcomes (i.e. asthma, wheeze, eczema), infectious diseases, growth and neurodevelopment, rickets, and bone mineral content and bone mineral density. The low certainty level of evidence ratings was because most of the evidence was imprecise, inconsistent, and with some or serious limitations based on risk-of-bias assessment. Dose-response RCTs were scarce. Brief summaries of key findings by outcomes are included below.

#### Atopic outcomes

Four RCTs (3 high risks and 1 medium risk for biases) reported asthma, wheeze, and/or eczema outcomes in children aged 0–4 years. All trials included an intervention arm of 400 IU/d of vitamin D_3_ with other arms being 800 IU/d, 1,200 IU/d, or placebo. Asthma and wheeze showed mixed results, but all three RCTs reporting on eczema found no significant differences for groups with different levels of vitamin D [25,26,59]. Two studies reported no significant findings for asthma [26,59], while one study reported a lower risk of asthma in neonates receiving 400 IU/d of vitamin D compared to a placebo [24]. For wheeze, one RCT reported no significant findings [25], while another found a significantly reduced risk of recurrent wheezing at 12 months in preterm black infants given sustained vitamin D supplementation compared to diet-limited supplementation [26].

##### Infectious disease outcomes

Eight RCTs (6 high risks and 2 medium risks for biases) reporting on 20 total infectious disease outcomes (respiratory infection outcomes, gastroenteritis, and other or unspecified infectious disease) were identified in 9 publications [24,26,27,30,60–63]. All RCTs included a daily regimen of vitamin D_3_, ranging from 400 to 1,200 IU/d, except for one group that received a bolus dose of 100,000 IU of vitamin D_3_ once every three months [63]. No between-group differences were found for 19 of these outcomes, but one study found a lower risk of developing influenza A after four months with 1,200 IU/d vs. 400 IU/d of vitamin D_3_ [27].

##### Growth and neurodevelopment outcomes

Thirteen RCTs (12 high risks and 1 medium risk for biases) assessed growth or neurodevelopment outcomes in healthy (*n* = 11 studies), low birth weight (*n* = 1), or preterm (*n* = 1) infants between ages 0 and 1 month. Five RCTs compared various daily doses of vitamin D (400, 800, 1,200, and/or 1,600 IU) [31,32,36,53,64,65], and one RCT compared a daily dose of 400 IU vitamin D with a bolus dose of 50,000 IU [47], and one RCT compared a weekly dose of 1,400 IU vitamin D to a placebo [66]. Three RCTs compared human milk or infant formula supplemented with vitamin D (400 IU/d supplement or 400–427 IU/L formulations) to human milk alone [33,67] or with placebo [28]. The remaining two RCTs included various combinations of vitamin D supplementation or placebo for both infants fed human milk (400 IU/d or placebo) and their lactating mothers (600 IU/d, 6,000 IU/d, 120,000 IU/month, or placebo) [52,68]. Overall, 11 RCTs reported no significant findings, while the remaining three reported mixed results, as follows. One RCT in healthy infants reported length improvements with vitamin D vs. a placebo (a non-randomised comparison group) [28], and another RCT in healthy infants reported significantly lower Alberta Infant Motor Scale scores (total, prone, and/or sitting scores) with higher (800 or 1,200 IU/d) vs. lower (400 IU/d) vitamin D_3_ doses [69]. An RCT in low-birthweight infants (1.8–2.5 kg) found significant benefits with 1,400 IU/week of vitamin D_3_ vs. placebo for some measurements (weight- and length-for-age z-scores, arm circumference) but no difference in others (weight-for-length z-scores or head circumference) at 6 months [66]. At 3–6 years post intervention, the vitamin D supplemented group had significantly lower body mass index (BMI), BMI z-scores, and arm muscle area but no other significant differences compared to the placebo group [38].

##### Rickets

Eight RCTs (2 high risks and 6 medium risks for biases) and one non-RCT (high risk for biases) reported the effect of different vitamin D intake levels on rickets. Most of these RCTs assigned vitamin D interventions as daily doses ranging from 200 to 1,000 IU/d [26,51,61,70–73] except for one RCT which assigned newborns to 1,400 IU of vitamin D per week [66]. All eight RCTs reported no rickets cases during the duration of the trials (1.5–36 months). The non-RCT assigned older infants (mean age of 2.26 years) to a dose of 25,000 IU per month combined with 15 mmol/d of calcium and found rickets in <2% of the study population by the end of the trial [29]. This study reported no between-group differences in rickets incidence for participants receiving calcium, vitamin D, calcium plus vitamin D, or no supplementation.

##### Bone mineral content or density (BMC or BMD) outcomes

Nine RCTs (8 high risks and 1 medium risk for biases) and one non-RCT (high risk for biases) assessed outcomes related to BMC or BMD outcomes. Two studies compared a single vitamin D dosing group to a placebo group [26,38], three compared infants fed human milk or formula fed with a vitamin D supplement to a group fed human milk with no supplement [28,33,37], and the remaining five studies compared groups with different doses of VD [30–32,34,36]. For most of these studies, vitamin D dosing was given as a daily regimen of 400 IU to 1,600 IU, while one study used a dose of 1,400 IU per week [38]. Two studies did not specify the total daily vitamin D dose for study groups given infant formula but reported vitamin D IU per litre of formula [28,33]. In all 10 studies, intervention duration was 2.5–23.5 months.

Five RCTs reported no difference in BMD or BMC outcomes when comparing vitamin D supplementation to human milk only [33] or when comparing groups with different vitamin D supplement doses [30–32,34]. Two studies reported benefits to BMC or BMD outcomes when comparing 400 IU/d vitamin D supplementation with placebo but did not report p-values or confidence intervals [26,28]. One dose-response RCT reported statistically significant benefits for most BMD measurements when comparing the highest dose of vitamin D with lower doses (1,600 IU/d vs. 400 IU/d; 1,600 IU/d vs. 1,200 IU/d) [36]. One RCT reported moderately significant (.05 < *p* < .1) benefits for distal radius (but not tibia) bone measurements when comparing 1,400 IU vitamin D per week to a placebo [38]. The non-RCT reported statistically significant (*p* < .05) benefits for BMC/BMD measures when comparing vitamin D supplementation in human milk or formula-fed infants with human milk alone [37].

#### KQ 2. What is the association between serum 25(OH)D concentrations and health outcomes in children aged 0–4 years?

Evidence was *very low* for the association between serum 25(OH)D concentration and atopic outcomes (i.e. asthma, wheeze, eczema), autoimmune disease, and infectious disease. Evidence was *low* for growth and neurodevelopment outcomes. Overall, the very low or low certainty of evidence ratings was due to concerns for potential biases due to absent or unclear demonstration that the outcome was not present at the start of the study, poor adjustment of possible confounders, and poor or unclear assessment of the outcome, and significant loss to follow up of participants.

For all other outcomes, the number of identified studies was *insufficient* (less than 3 studies per outcome) to perform strength of evidence assessments. Only one case-cohort study reported on serum 25(OH)D concentration and fracture outcomes [74], one cohort study reported on blood pressure outcomes [75], and no studies reported on bone mineral density, bone mineral content or rickets outcomes.

##### Atopic outcomes

Three cohort and one case-cohort study assessed the association between 25(OH)D concentrations at age 0–4 years and subsequent asthma, wheeze, and/or eczema outcomes. Of these, three studies found no association with asthma [76,77], wheezing [76], or eczema [78], while one study reported higher odds of asthma (but not medicated asthma), wheezing, and eczema at 10 years of age in cohort members with more frequent deficient 25(OH)D concentrations (<50 nmol/L) between age 6 months and 10 years [39].

##### Infectious disease outcomes

Three cohort studies assessed the association between serum 25(OH)D concentrations and various infectious diseases including upper respiratory tract infection (URTI), acute lower respiratory tract infection (ALRI), respiratory tract infection, malaria infection, and oral candidiasis [39,43,44]. Most reported associations between serum 25(OH)D concentrations and infectious disease outcomes were not statistically significant, except for the following. In one cohort, infant serum 25(OH)D levels of 20–29.9 ng/mL were associated with decreased risk of oral candidiasis compared to lower levels (<10 ng/mL) but increased risk of malaria infection compared to higher levels (≥30 ng/mL) [43]. Another cohort found that underweight children with sufficient 25(OH)D levels (> =75 nmol/l) at birth and early childhood had an increased risk for URTI compared to those with insufficient (> =50 and <75 nmol/l) or deficient (<50 nmol/L) levels [44].

##### Autoimmune disease outcomes

Seven observational studies (in six publications) that assessed the association between serum vitamin D levels and three autoimmune outcomes including type 1 diabetes (T1D), islet autoimmunity, and juvenile idiopathic arthritis (JIA) were identified. One case-cohort [79] and three nested case-control studies [79–81] reported no associations with T1D, and another case-cohort found no associations with JIA [42]. There were mixed results for islet autoimmunity. One case-cohort reported no association [40], while one nested case-control study of children at increased genetic risk of T1D found higher serum 25(OH)D in infancy and early childhood was associated with lower odds of islet autoimmunity [41].

##### Growth and neurodevelopment outcomes

Six observational studies (four cohort and two nested case-control studies) that assessed growth and/or neurodevelopment outcomes were identified. No significant linear associations 25(OH)D concentrations and growth and development outcomes in all six studies [43,81–85], but two cohort studies found positive associations between categorical serum 25(OH)D levels (at different cutoffs) and growth or neurodevelopment. Specifically, one study reported better weight-for-length z-scores at 20 months of age for infants with higher (20–29.9 ng/mL) vs. the lowest (<10 ng/mL) serum 25(OH)D levels [43]. Another cohort study found that newborns with the highest (≥21.8–30.3 nmol/L) 25(OH)D levels at birth had greater IQ scores at age 19 than those with 25(OH)D levels ≤13.3 nmol/L at birth [84].

#### KQ 3. What is the effect of vitamin D intake on serum 25(OH)D concentrations in children aged 0–4 years?

Altogether, 66 RCTs assessed the effect of vitamin D intake on serum 25(OH)D concentrations in children aged 0–9 years. Of these, 51 RCTs were conducted in children ages 0–4 years [25–28,30–33,35,36,38,45–49, 51–54,58,60–70,72,73,86–103]. Results and meta-regression of RCTs in children ages 4–9 years were not summarised here but are reported in the full evidence report (see Supplemental File). Evidence was found to be *moderate* for the effect of daily vitamin D supplementation (vitamin D_3_ or D_2_ supplements to infants/children) on raising serum 25(OH)D concentrations, but evidence for non-daily vitamin D supplementation (including single dose) was *low*. Evidence was *very low* for infant/child serum 25(OH)D concentrations associated with vitamin D supplementation given to post-partum lactating mothers, food interventions containing vitamin D, or combined vitamin D and calcium supplementation.

##### Daily vitamin D supplementation

**Figure 3.**
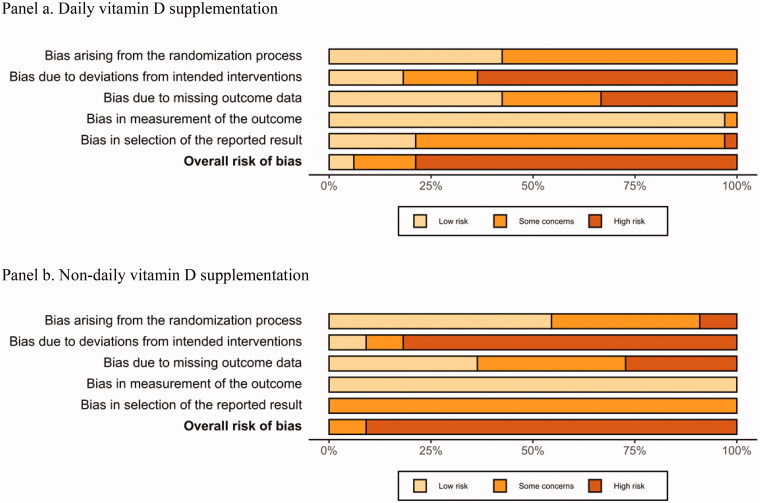
Summary risk-of-bias assessments for randomised controlled trials reporting the effect of daily vitamin D supplementation (panel a) or non-daily vitamin D supplementation (panel b) on serum 25(OH)D concentrations in children 0–4 years.

This systematic review identified 38 unique studies (in 39 reports) that examined the effects of daily vitamin D intake on serum 25(OH)D concentration. Of these, 30 trials (in 31 publications) were conducted in children 0–12 months [25–28,30–33,36,45,47,52,54,61,64,65,67,68,72,73,87–90,93–96,98,100,101], and one RCT was conducted in children 1–4 years (mean age 2.7 years) [60]. The overall ROB assessment of these RCTs is shown in Figure 3a.

**Figure 4.**
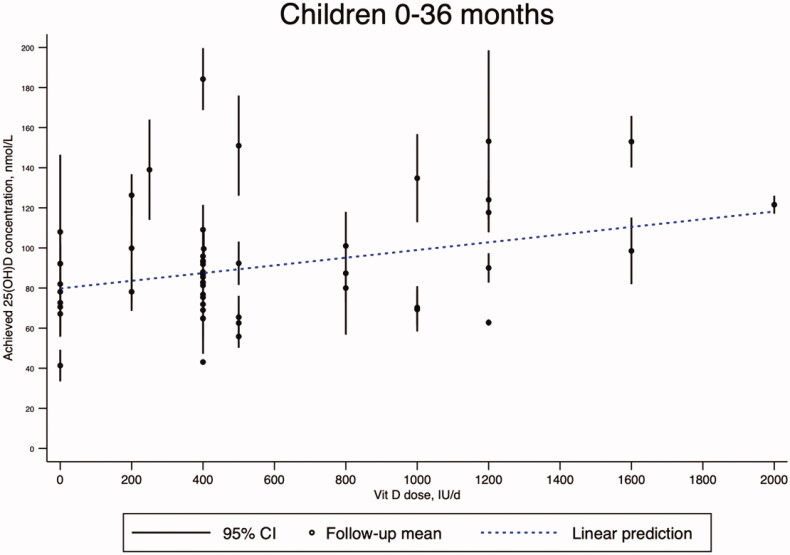
Random-effects meta-regression analysis on the association between daily vitamin D supplementation and 25(OH)D concentrations achieved post-intervention in children 0–4 years. Legend: CI = confidence interval; IU/d = international units per day; Vit D = vitamin D from supplements.

Our random effects meta-regression analysis of 28 RCTs in children under 4 years of age (27 studies with mean age of 0–12 months and one study with mean age of 2.7 years) showed that each 100 IU/d increase in vitamin D supplementation was associated with an average of 1.92 (95% CI: 0.28, 3.56) nmol/L increase in achieved 25(OH)D concentration (*n* = 53 intervention arms; *p* = .022; adjusted *R*^2^ = 9.07%) with large residual heterogeneity (*I^2^* = 99.39%) (Figure 4). Three RCTs were not included in this meta-regression analysis due to insufficient quantitative data reporting [26,89,93].

##### Non-daily vitamin D supplementation

There were 11 included RCTs that assessed the effect of non-daily vitamin D (from supplements or fortified foods) on 25(OH)D in children aged 0–4 years [45–50,56,66,89,98,104]. The overall ROB assessment of these RCTs is shown in Figure 3b. These studies assigned participants to intermittent dosing regimens (weekly, monthly, or bimonthly dosing) for variable durations or to single bolus doses of vitamin D_3_ (ranging from 50,000 to 600,000 IU). Most of the studies comparing different levels of vitamin D supplementation identified dose-response such that higher intakes of vitamin D supplementation resulted in higher 25(OH)D levels. The degree of change in 25(OH)D depended on the vitamin D dose, intervention and follow-up duration, study site latitude, and season of assessment.

##### Vitamin D supplementation to post-partum mothers

Four RCTs (all high risk for biases) assessed the effect of maternal vitamin D supplementation on the 25(OH)D levels of breastfeeding infants. Maternal supplementation included daily doses (400–6,400 IU) of vitamin D or a monthly dose (120,000 IU) of vitamin D_3_, while the mothers’ breastfeeding infants received no vitamin D supplement or were given a placebo. These infants’ 25(OH)D concentrations were compared to infants who were given vitamin d supplements directly (300–1,000 IU/d) and were breastfed by non-supplemented mothers (*n* = 3 studies) or mothers given 400 IU/d of vitamin D (*n* = 1 study). Results from these studies were mixed [51–54].

##### Food interventions

Three RCTs (2 medium and 1 high risk for biases) assessed the effect of food fortified with vitamin D (80–1,000 IU/d) on 25(OH)D concentrations. One study comparing fortified and non-fortified foods reported no group differences [55]. One study found that direct vitamin D supplementation to breastfed infants resulted in significantly increased 25(OH)D compared to neonates exclusively fed with fortified infant formula and those with no intervention [33]. The final study reported significantly increased 25(OH)D in fair- and dark-skinned children given foods fortified with 400 or 1,000 IU/d vitamin D but no change in 25(OH)D if foods were fortified with 80 IU/d [56].

##### Combined vitamin D and calcium supplementation

Three RCTs (all high risk for biases) reported the effect of combined vitamin D and calcium supplementation on serum 25(OH)D concentrations. Two studies found no significant group differences for children given equal vitamin D doses but different doses of calcium [58] or equal calcium doses with different doses of vitamin D [34]. The other study found significantly higher 25(OH)D in children given vitamin D plus calcium compared to calcium supplementation only [57].

#### Vitamin D upper limits

##### KQ UL1a. At what levels of vitamin D intake are adverse effects observed in children aged 0–4 years?

Altogether, 47 studies with various designs (RCTs, single-arm interventions, cohorts, case-cohorts, nested case-controls, cross-sectional studies, and case reports) reported on the association between vitamin D intake or serum 25(OH)D and adverse effects (see Supplemental File for references). Evidence was *very low* on two of the upper limit outcomes – hypercalcemia and hypercalciuria. Generally, the rate of hypercalcemia increased with the dose of vitamin D; however, studies were inconsistent and imprecise. The rate of hypercalciuria was variable among studies and intervention arms. It should be noted that the definitions of hypercalcemia and hypercalciuria were variable across included studies. For all other UL KQ1a outcomes (i.e. growth and development, nephrocalcinosis, kidney stones, and mortality), evidence was deemed *insufficient* due to limited high-quality studies identified by this systematic review.

##### KQ UL1b. What are levels of vitamin D intake at which a prespecified threshold of serum 25(OH)D is reached in children aged 0–4 years?

For KQ 1 b, RCTs included in KQ3 (described earlier) that reported the effect of vitamin D intake on achieving prespecified thresholds of serum 25(OH)D, as defined by the original studies, are included. Studies were organised separately by vitamin D intervention types: daily dose interventions, single and intermittent large dose interventions, or interventions with fortified and non-fortified foods. Additionally, a variety of 25(OH)D assay methods were used across included studies, so no meta-analysis was performed. Prespecified thresholds of serum 25(OH)D included 30 nmol/L (12 ng/mL), 50 nmol/L (20 ng/mL), 75 nmol/L (30 ng/mL), 125 nmol/L (50 ng/mL), and 150 nmol/L (60 ng/mL). Results varied by a study where each reported the percentage of participants achieving prespecified serum 25(OH)D thresholds as the outcome, and the percentage of participants reaching the prespecified threshold was variable and may have depended on the 25(OH)D assay methods, threshold level, intervention dose, and intervention duration. Therefore, we concluded that evidence is *insufficient* for answering KQ UL1b despite having found a *moderate* level of evidence for the effect of daily vitamin D supplementation on raising serum 25(OH)D concentrations (KQ3).

## Discussion

Vitamin D is a conditionally essential micronutrient because the amount synthesised in the skin under sunlight (ultraviolet [UV]-B light) exposure is often not sufficient to meet our needs, and thus humans need to consume dietary forms of vitamin D under certain circumstances. Vitamin D content in human milk is highly variable and might be affected by season, maternal dietary intake of vitamin D, and ethnicity [105], and there is little vitamin D that occurs naturally in the food supply. The efficacy of conversion of 7-dehydrocholesterol in the skin after exposure to UV-B light to cholecalciferol (vitamin D_3_) is dependent on the time of day, the season of the year, latitude, skin colour, and age. Vitamin D_2_ (ergocalciferol) is produced in mushrooms and yeast. The native form of vitamin D is not biologically active. The active form of vitamin D is 1,25(OH)_2_D (calcitriol), which is first hydroxylated from vitamin D to 25(OH)D in the liver and then hydroxylated by the kidney. One of the major biological functions of vitamin D is to maintain calcium homeostasis. Calcitriol also acts as a hormone working through the activation of signal transduction pathways linked to vitamin D receptors on cell membranes. Major sites of action include the intestine, bone, parathyroid, liver, and pancreatic beta cells. Thus, vitamin D could be considered a prohormone that can affect the risks of disease development.

In the past decade, many high-income countries have adopted the NRV framework as well as the methodological approach to deriving two core NRVs, the Average Requirement (AR) and the Tolerable Upper Intake Level (UL), that are needed to assess the nutritional adequacy and safety of nutrient intakes by population groups [106]. However, due to constraints by a lack of resources and access to data, particularly for conducting systematic reviews, low- and middle-income countries and some global organisations, such as the WHO and the FAO, could not carry out the full process of deriving the AR and UL [107]. The first step in the decision-making process associated with the development of NRVs is the identification of potentially useful measures or “indicators” that reflect a health outcome causally linked to the intake of the nutrient. Integrating systematic reviews in the NRV framework can provide a transparent and reproducible process. The evidence report summarised herein was commissioned by the FAO/WHO in 2020 and was prepared to support an international expert group to derive AR and UL values for children aged 0–4 years across the globe. It is important to note that the evidence report does not make nor was it intended to make recommendations for NRVs concerning vitamin D, as this responsibility lies with the expert group. The evidence report was the core source of data but not the only data source that the expert group considered during their deliberations. To derive vitamin D NRVs and make intake recommendations, the FAO/WHO expert group applied the dose-response approach, which is an intake–response assessment describing how a known physiological outcome changes according to the intake of a nutrient. The physiological outcome may be a biomarker of function, disease, or other health outcomes. The highest strength of evidence for intake-response assessment is high-quality, dose-response trials with relevant physiological outcomes. The list of relevant physiological outcomes was selected by the expert group *a priori* to define the health outcomes of interest in each key question of the present systematic review. In this systematic review, we found an *insufficient* or *low* certainty level of evidence from RCTs or non-RCTs regarding the effect of different levels of vitamin D intake on clinical outcomes (KQ1). With scarce dose-response trials, data from observational studies relating a dose-response relationship between 25(OH)D concentrations and clinical outcomes can be used to complement RCT data for causal inference. However, we found the observational evidence (KQ2) is of lower quality than evidence from trials (KQ1). Taken together (Table 5), the current totality of evidence from trials and prospective observational studies do not reach sufficient certainty level to support a causal relationship between vitamin D intake and asthma, wheeze, eczema, infectious diseases, or rickets (most trials reported no rickets) in generally healthy infants and young children. Low-quality evidence suggests the effect of vitamin D intake on growth and neurodevelopment outcomes in generally healthy infants and young children is mostly null, and the effect on bone mineral content and bone mineral density outcomes is inconsistent. Evidence regarding adverse outcomes (growth and development, nephrocalcinosis, kidney stones, mortality, hypercalcemia and hypercalciuria) of excessive vitamin D intake was deemed *insufficient* or *very low* certainty (KQ UL1a) due to poor-quality data. The only body of evidence that reached *moderate* level of certainty was regarding the effect of daily vitamin D supplementation (vitamin D_3_ or D_2_ supplements to infants/children) on increasing serum 25(OH)D concentration, which is a measure of vitamin D status (KQ3). However, evidence is *insufficient* to answer the key question regarding are levels of vitamin D intake at which a prespecified threshold of serum 25(OH)D is reached (KQ UL1b).

Scientific communities have reached a consensus that serum total 25(OH)D concentration, which reflects the amount of vitamin D from both dietary sources and cutaneous synthesis, can be used as a biomarker of vitamin D status. Yet, there is currently no consensus on the definitions of vitamin D status, e.g. deficiency, insufficiency, sufficiency and toxicity, based on serum 25(OH)D concentrations [108]. Variability of 25(OH)D assays is widely recognised; therefore, only standardised 25(OH)D data can provide the necessary level of accuracy and precision essential to the process of developing vitamin D guidelines and policies including vitamin D NRVs [109]. A standardised 25(OH)D measurement is defined as one that provides the ‘true’ total 25(OH)D concentration as measured by the three Joint Committee for Traceability in Laboratory Medicine (JCTLM)-recognised reference measurement procedures [110]. Standardisation of 25(OH)D measurements requires individual data, so it cannot be done using the group or summary data extracted from the published literature. Therefore, the meta-regression results (Figure 4) should be interpreted with caution due to our inability to account for the variability of the 25(OH)D assays in our analysis. The meta-regression analysis presented in the evidence report was only the first step in modelling the association between vitamin D supplementation doses and achieved 25(OH)D concentrations. The analysis did not adjust for known confounding factors such as baseline 25(OH)D, duration of intervention, and calcium intake levels. However, it should be stressed that meta-regression analysis of summary data has limited ability to properly adjust for confounding and may suffer from ecological fallacy.

Another limitation of this systematic review is that many included RCTs and observational studies were of poor quality, often due to challenges in conducting vitamin D research. Many included RCTs were rated as high risk for bias due to deviations from the intended interventions. Unlike clinical guidelines, nutrient intake recommendations prefer data on the effect of adhering to intervention (efficacy data) than the ‘intention to treat’ data (effectiveness data). This is because setting NRVs needs accurate and precise measurement of nutrient intake levels in both RCTs and observational studies. To observe sufficient changes in clinical outcomes, long intervention durations are needed. This makes adherence during the intervention period a challenge when conducting RCTs. Per Cochrane ROB assessment instructions, both naïve ‘per-protocol’ analyses (excluding trial participants who did not receive their assigned intervention) and ‘as treated’ analyses (in which trial participants are grouped according to the intervention that they received, rather than according to their assigned intervention) should be considered inappropriate. While observational studies are more feasible to examine the effect of vitamin D on long-term outcomes, they have their own challenges and limitations. Specifically, dietary assessments of vitamin D intake levels are not accurate due to the inadequacy of nutrient composition tables for vitamin D [15], and multiple 25(OH)D measurements over time are needed to more accurately estimating long-term vitamin D status.

The evidence report provided the expert group with a foundation and core set of data to help set vitamin D NRVs for infants and children up to 4 years of age. Modelling the intake-response relationship of vitamin D intake level to achieve a 25(OH)D level that is linked to an adequacy outcome, such as the prevention of rickets (osteomalacia), would be required to set a vitamin D AR. Similar to setting a vitamin D UL, an intake-response model is needed to better estimate vitamin D intake levels that increase the risk of adverse outcomes. Unlike IOM’s DRIs, FAO/WHO’s intake recommendations do not assume no vitamin D from VU-B exposure. Thus, another systematic review was commissioned to quantify the effects of UV-B exposure on vitamin D status [111] so the amount of cutaneous synthesis of vitamin D can be accounted for in the intake-response modelling. Finally, since the FAO/WHO’s intake recommendations are intended to be used by countries across the globe, information about the local context, such as risk of malnutrition or latitude (a proxy for UV-B exposure level), is critical for making the most appropriate adjustments to vitamin D NRVs to suit the intended population.

### Disclaimer

The opinions expressed in this manuscript should not be construed as an official endorsement by the FAO/WHO.

## Supplementary Material

Supplemental Material

## Data Availability

All data are available in the Supplemental File.
