# Supplementary material for: Vitamin D intakes and health outcomes in infants and preschool children: Summary of an evidence report
Source: Ann Med. 2022 Aug 17;54(1):2278–301. doi: 10.1080/07853890.2022.2111602 (PMC9387322; doi:10.1080/07853890.2022.2111602)
Supplement: Supplemental Material [file IANN_A_2111602_SM2422.pdf]

Vitamin D Requirements and Upper Limits in Children 0-36 months: A Systematic Review

Investigators:

Andrew R. Beauchesne, MD, MS[1]

Kelly Copeland Cara, MS[2]

Danielle M. Krobath, MS[2]

Laura Paige Penkert, MS, MPH[1,2]

Shruti P. Shertukde, MS[2]

Danielle S. Cahoon, MS, PhD[2]

Belen Prado, MBA, PhD[2]

Ruogu Li, MS, MPH[1,2]

Qisi Yao, MPH[1]

Jing Huang, MS, MPH[1,2]

Tee Reh, MPH[1]

Principal Investigator:

Mei Chung, PhD, MPH[1,2*]

Research Librarian:

Amy E. LaVertu, MLS[1,2]

Affiliations:

[1] School of Medicine, Tufts University, Boston, Massachusetts (U.S.A.)

[2] Friedman School of Nutrition Science and Policy, Tufts University, Boston, Massachusetts (U.S.A.)

*Corresponding to:

150 Harrison Ave, Boston, Masshachusetts 02111 (U.S.A.). Email: Mei_Chun.Chung@tufts.edu. Work TEL: 1+617-636-2966

Funding: This work was supported by the Food and Agriculture Organization of the United Nations (FAO) and the World Health Organization (WHO).

Conflicts of interest: None to disclose.

Table of Contents

Background **4**
Key Questions and Study Eligibility Criteria **5**
I. Vitamin D Requirements 5
II. Vitamin D Upper Limits 10

Methods **12**
Literature Searches and Study Selection Process **12**
Data Extraction **12**
Risk of Bias Assessment **12**
Data Synthesis and Strength of Evidence Rating **13**
Meta-analysis **13**
Organization of this report **13**

Results **14**
I. Vitamin D Requirements **15**
KQ1. What is the effect of different levels of vitamin D intake on health outcomes in children aged 0 to 4 years? 15
KQ1. Atopic Outcomes: Asthma, Wheezing, and Eczema. 15
KQ1. Infectious Disease Outcomes 18
KQ1. Growth and Neurological Development Outcomes 26
KQ1. Rickets 35
KQ1. Bone Mineral Content and Bone Mineral Density Outcomes 40
KQ1. Blood Pressure Outcomes 47
KQ1. UV Light Studies 49
KQ2: What is the association between serum 25(OH)D concentrations and health outcomes in children aged 0 to 4 years? 50
KQ2. Atopic Outcomes: Asthma, Wheezing, and Eczema 50
KQ2. Infectious Disease Outcomes 55
KQ2. Autoimmune Disease Outcomes 59
KQ2. Growth and Neurological Development Outcomes 65
KQ2. Fracture Outcome 71
KQ2. Blood Pressure 71
KQ3. What is the effect of vitamin D intake on serum 25(OH)D concentrations in children aged 0 to 4 years? 75
KQ3. Studies comparing different levels of daily vitamin D supplementation 83
KQ3 Sub-section 2. Studies using non-daily vitamin D supplementation dosing regimens 98
KQ3 Sub-section 3. Studies comparing supplementation for post-partum mothers to supplementation for infants 103
KQ3 Sub-section 4. Studies using food interventions with different levels of vitamin D or comparing food interventions with vitamin D supplements 108
KQ3 Sub-section 5. Studies examining the effects of combined vitamin D and calcium supplementation 111
II. Vitamin D Upper Limits **114**
ULs KQ1a. At what levels of vitamin D intake are adverse effects observed in children aged 0 to 4 years? 114

ULs KQ1b. What are levels of vitamin D intake at which a prespecified threshold of serum 25(OH)D is reached in children aged 0 to 4 years? ... 128

Conclusions ... 138

Vitamin D Requirements ... 138

KQ1. What is the effect of different levels of vitamin D intake on health outcomes in children aged 0 to 4 years? ... 138

KQ2. What is the association between serum 25(OH)D concentrations and health outcomes in children aged 0 to 4 years? ... 139

KQ3. What is the effect of vitamin D intake on serum 25(OH)D concentrations in children aged 0 to 4 years? ... 140

Vitamin D Upper Limits ... 140

ULs KQ1a. At what levels of vitamin D intake are adverse effects observed in children aged 0 to 4 years? ... 140

ULs KQ1b. What are levels of vitamin D intake at which a prespecified threshold of serum 25(OH)D is reached in children aged 0 to 4 years? ... 140

References ... 142

Appendix 1: GRADE Evidence Profile Tables ... 150

Appendix 2: Bibliography ... 160

Background

The objective of this systematic review was to synthesize all available evidence that met predefined eligibility criteria for informing a Food and Agriculture Organization of the United Nations (FAO)/World Health Organization (WHO) expert group, whose charge was to update the vitamin D requirements for children aged 0 to 4 years.[1] During phase I of this work, the FAO/WHO expert group reviewed and discussed the results of a scoping review[2] and other background documents. The group then formulated the key questions (KQs) of this systematic review using a generic analytic framework (**Figure 1**).[3] In this analytic framework, the term "Indicators of Exposure (Nutrient Intake)," as defined within the Dietary Reference Intake (DRI) context, are measures that correlate with dietary intake of a nutrient, such as nutrient biomarkers, nutritional status, or markers of nutritional status. Specifically, the indicators of vitamin D exposure (i.e., vitamin D intake and sun exposure) included serum 25-hydroxy vitamin D [25(OH)D] concentrations.[4] Dose-response randomized controlled trials (RCTs) assessing the effects of nutrient intake levels on age-specific clinical outcomes of public health importance (Arrow 1 in Figure 1) would provide the best direct evidence for setting nutrient reference values (NRVs). However, such evidence is often lacking for chronic disease endpoints, as shown in the scoping review of vitamin D and health outcomes in children 0 to 4 years.[2] In the absence of direct evidence, a "piecemeal approach" has been suggested as an option for setting NRVs.[5] For example, data that address the dose-response relationship between nutrient intake and indicators of exposure or surrogate outcomes (Arrows 4 and 5 in Figure 1) can be synthesized with data that address the relation between indicators of exposure and clinical outcomes (Arrow 3 in Figure 1) to set NRVs. This "piecemeal approach" (also known as the "dose-response approach"[6]) has the advantage of relying on a wider breadth of available evidence but also has many uncertainties.[5]

In phase I of this work, the FAO/WHO expert group determined a dose-response approach would be appropriate for setting vitamin D requirements. Thus, the expert group utilized the analytic framework[3] (Figure 1) to formulate KQs for vitamin D requirements and upper limits (ULs).

Figure 1. A generic analytic framework to assist formulation of Key Questions for the development of DRIs using a dose-response approach

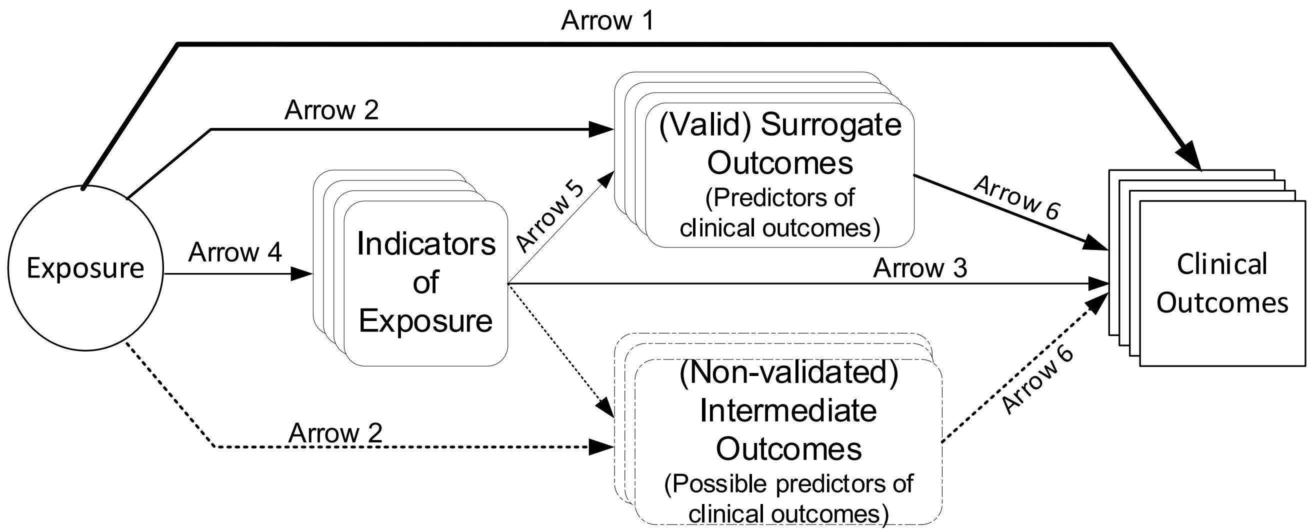

Arrow 1: Association of exposure with clinical outcomes of interest.
Arrow 2: Association of exposure with surrogate or intermediate outcomes (with good or possible evidence for linkage with clinical outcomes).
Arrow 3: Association of indicators of exposure to clinical outcomes.
Arrow 4: Association between exposure and indicators of exposure.
Arrow 5: Association of indicators of exposure to surrogate or intermediate outcomes (with good or possible evidence for linkage with clinical outcomes).
Arrow 6: Association between surrogate outcomes (with good or possible evidence for linkage) and clinical outcomes.

Key Questions and Study Eligibility Criteria

I. Vitamin D Requirements

KQ1. What is the effect of different levels of vitamin D intake on health outcomes in children aged 0 to 4 years? (See **Table 1** for KQ1 eligibility criteria.)

Planned subgroup analyses:

- Level of vitamin D intake (i.e., dose-response)
- Source of vitamin D (e.g., food, fortified food, supplements, formula, breast milk)
- For supplements, different formulation of supplements
- Different forms of vitamin D (e.g., D_2, D_3)
- Calcium intake
- Sun/UV-B exposure, latitude, months of year when 25(OH)D levels were assessed
- Age
- Breastfeeding (BF) status (e.g., exclusive BF, any BF, exclusive formula)
- Analytical method for determining circulating 25(OH)D (including by calibration to NIST standard, etc., where appropriate)

- Race and ethnicity
- Skin color

Table 1. Vitamin D requirements KQ1 eligibility criteria

Category	Inclusion Criteria	Exclusion Criteria
Study designs of interest	• Randomized (paralleled or crossover) controlled trials, or nonrandomized controlled trials<br>• Intervention duration ≥ 2 weeks	• In vitro (cell) and animal studies<br>• Observational studies [Note: Dietary assessments of vitamin D intake levels were not included due to inadequacy of nutrient composition tables for vitamin D[7]]<br>• Single-arm trials<br>• Studies that used non-concurrent cohorts or non-concurrent controls<br>• Unpublished studies (e.g., conference abstracts, posters)
Populations of interest	Generally healthy[a] children 0 to 4 years old	• Critically ill children admitted to intensive care unit<br>• Studies that enrolled exclusively premature infants (≤ 32 weeks gestational age) or very low birth weight infants (≤ 1500 grams)<br>• Studies conducted exclusively in children with moderate or severe acute malnutrition (MAM/SAM)
Interventions of interest	• Dietary vitamin D intake (with or without calcium) from foods or supplements<br>• UV exposure to manipulate 25(OH)D levels	• Non-oral intake of vitamin D such as injections or peripheral parenteral nutrition<br>• Intervention studies in which effects of vitamin D and/or calcium cannot be isolated<br>• Vitamin D analogs (e.g., calcifedio, calcijex, calcipotriol, calcitriol, doxercalciferol, hectorol, paricalcitrol, rayaldee, rocaltrol, zemplar)
Comparators of interest	Any	None
Outcomes of interest	• Growth and development (anthropometric indices, failure to thrive, etc.)[b]<br>• Neurological development[c]<br>• Infectious disease	• Maternal health-related outcomes<br>• Any outcome measured only at birth in mothers or in infants<br>• Lead concentration<br>• Health-service utilization outcomes

Category	Inclusion Criteria	Exclusion Criteria
	• Autoimmune disease<br>• Asthma, wheezing, or atopic dermatitis<br>• Fracture<br>• Bone mineral density or bone mineral content (irrespective of the method employed, for example, ultrasonography, DEXA etc.)<br>• Rickets (including "nutritional rickets")<br>• Blood pressure<br>• Calcium absorption and retention[d]<br>• COVID-19	

DEXA = Dual-energy X-ray absorptiometry; MAM = moderate acute malnutrition; SAM = severe acute malnutrition; UV = ultraviolet

[a] "Generally healthy" populations are defined as having ≤20% of the study population with disease at the study's baseline with the exception of the case-control study design. Nutrition deficiencies, overweight, and obesity are not considered diseases in this systematic review.

[b] For growth and development outcomes, the populations of interest are expanded to include children 0-9 years old because growth and development outcomes are also considered outcomes of interest for vitamin D and calcium ULs. All anthropometric measures are considered outcomes of interest, such as height, weight, length/height for age, weight for age, weight for height/length, BMI, related z-scores, waist circumference, mid-arm circumference (MUAC), skinfold thickness, head circumference.

[c] Autism is not an outcome of interest, but cognitive or intellectual development assessed by IQ is of interest.

[d] For the calcium absorption and retention outcomes, the minimal intervention duration of 2 weeks criterion does not apply because calcium absorption is also an outcome of interest for calcium requirements.

KQ2. What is the association between serum 25(OH)D concentrations and health outcomes in children aged 0 to 4 years? (See **Table 2** for KQ2 eligibility criteria.)

Planned subgroup analyses:

- Level of vitamin D intake (i.e., dose-response)
- Source of vitamin D (e.g., food, fortified food, supplements, formula)
- Different forms of vitamin D (e.g., D_2, D_3)
- Sun/UV-B exposure, latitude, time of year assessed
- Age
- Breastfeeding status
- Analytical method for determining circulating 25(OH)D (including by calibration to NIST standard, etc., where appropriate)
- Race and ethnicity
- Skin color

Table 2. Vitamin D requirements KQ2 eligibility criteria

Category	Inclusion Criteria	Exclusion Criteria
Study designs of interest	• Cohort, nested case-control, or case-cohort studies in which 25(OH)D concentrations were measured before outcome ascertainment.<br>• Follow-up duration ≥ 2 weeks	• In vitro (cell) and animal studies<br>• Intervention studies<br>• Cross-sectional studies reporting only prevalence data (i.e., no correlation or association analyses)<br>• Retrospective case-control studies<br>• Case reports or case series
Populations of interest	Generally healthy[a] children 0 to 4 years old	• Critically ill children admitted to intensive care unit<br>• Studies that enrolled exclusively premature infants (≤ 32 weeks gestational age) or very low birth weight infants (≤ 1500 grams)<br>• Studies conducted exclusively in children with moderate or severe acute malnutrition (MAM/SAM)
Exposures of interest	25(OH)D concentrations (irrespective of measurement assay)	Dietary assessments of vitamin D intake only [Note: Dietary assessments of vitamin D intake levels were not included due to inadequacy of nutrient composition tables for vitamin D[7]]
Comparators of interest	Different levels of 25(OH)D concentrations	None
Outcomes of interest	• Growth and development (anthropometric indices, failure to thrive, etc.)[b]<br>• Neurological development[c]<br>• Infectious disease<br>• Autoimmune disease<br>• Asthma, wheezing, or atopic dermatitis<br>• Fracture<br>• Bone mineral density or bone mineral content (irrespective of the method employed, for example, ultrasonography, DEXA etc.)<br>• Rickets (including "nutritional rickets")<br>• Blood pressure	• Maternal health-related outcomes<br>• Any outcome measured only at birth in mothers or in infants<br>• Lead concentration<br>• Health-service utilization outcomes

Category	Inclusion Criteria	Exclusion Criteria
	• Calcium absorption and retention[d]	

DEXA = Dual-energy X-ray absorptiometry; MAM = moderate acute malnutrition; SAM = severe acute malnutrition

[a] "Generally healthy" populations are defined as having ≤20% of the study population with disease at the study's baseline with the exception of the case-control study design. Nutrition deficiencies, overweight, and obesity are not considered diseases in this systematic review.
[b] For growth and development outcomes, the populations of interest are expanded to include children 0-9 years old because growth and development outcomes are also considered outcomes of interest for vitamin D and calcium ULs. All anthropometric measures are considered outcomes of interest, such as height, weight, length/height for age, weight for age, weight for height/length, BMI, related z-scores, waist circumference, mid-arm circumference (MUAC), skinfold thickness, head circumference.
[c] Autism is not an outcome of interest.
[d] For the calcium absorption and retention outcomes, the minimal follow-up duration of 2 weeks criterion does not apply because calcium absorption is also an outcome of interest for calcium requirements.

KQ3. What is the effect of vitamin D intake on serum 25(OH)D concentrations in children aged 0 to 4 years? (See **Table 3** for KQ3 eligibility criteria.)

Planned subgroup analyses:

- Level of vitamin D intake (i.e., dose-response)
- Source of vitamin D (e.g., food, fortified food, supplements, formula)
- For supplements, different formulation of supplements
- Different forms of vitamin D (e.g., D_2, D_3)
- Calcium intake
- Sun/UV-B exposure, latitude, time of year assessed
- Age
- Breastfeeding status
- Analytical method for determining circulating 25(OH)D (including by calibration to NIST standard, etc., where appropriate)
- Race and ethnicity
- Skin color

Table 3. Vitamin D requirements KQ3 and ULs KQ1b eligibility criteria

Category	Inclusion Criteria	Exclusion Criteria
Study designs of interest	• Randomized (paralleled or crossover) controlled trials, or nonrandomized controlled trials<br>• Intervention duration ≥ 2 weeks	• In vitro (cell) and animal studies<br>• Observational studies<br>• Single-arm trials<br>• Studies that used non-concurrent cohorts or non-concurrent controls<br>• Unpublished studies (e.g., conference abstracts, posters)
Populations of interest	Generally healthy[a] children 0 to 9 years old	• Critically ill children admitted to intensive care unit

Category	Inclusion Criteria	Exclusion Criteria
		• Studies that enrolled exclusively premature infants (≤ 32 weeks gestational age) or very low birth weight infants (≤ 1500 grams)<br>• Studies conducted exclusively in children with moderate or severe acute malnutrition (MAM/SAM)
Interventions of interest	Dietary vitamin D intake (with or without calcium) from foods or supplements	• Non-oral intake of vitamin D such as injections or peripheral parenteral nutrition<br>• Intervention studies in which effects of vitamin D and/or calcium cannot be isolated<br>• Vitamin D analogs
Comparators of interest	Any	None
Outcomes of interest	25(OH)D concentrations (irrespective of measurement assay)	None

MAM = moderate acute malnutrition; SAM = severe acute malnutrition

[a] "Generally healthy" populations are defined as having ≤20% of the study population with disease at the study's baseline with the exception of the case-control study design. Nutrition deficiencies, overweight, and obesity are not considered diseases in this systematic review.

II. Vitamin D Upper Limits

KQ1a. At what levels of vitamin D intake are adverse effects observed in children aged 0 to 4 years? (See **Table 4** for ULs KQ1a eligibility criteria.)

KQ1b. What are levels of vitamin D intake at which a prespecified threshold of serum 25(OH)D is reached in children aged 0 to 4 years? (See Table 3 for ULs KQ1b eligibility criteria.)

Table 4. Vitamin D ULs KQ1a eligibility criteria

Category	Inclusion Criteria	Exclusion Criteria
Study designs of interest	• Intervention studies of any design<br>• Observational studies of any design<br>• Case reports of excess vitamin intake (as defined in the original studies)	• In vitro (cell) and animal studies<br>• Unpublished studies (e.g., conference abstracts, posters)
Populations of interest	Generally healthy[a] children 0 to 9 years old	• Critically ill children admitted to intensive care unit<br>• Studies that enrolled exclusively premature infants (≤ 32 weeks gestational age) or very low birth weight infants (≤ 1500 grams)<br>• Studies conducted exclusively in children with moderate or severe acute malnutrition (MAM/SAM)
Interventions or exposures of interest	• Intervention studies: Dietary vitamin D intake (with or without calcium) from foods or supplements<br>• Observational studies: 25(OH)D concentrations (irrespective of measurement assay)	• Non-oral intake of calcium and/or vitamin D such as injections or peripheral parenteral nutrition<br>• Intervention studies in which effects of vitamin D and/or calcium cannot be isolated<br>• Vitamin D analogs
Comparators of interest	Any	None
Outcomes of interest	• Growth and development[b]<br>• Hypercalcaemia<br>• Hypercalciuria<br>• Kidney stones<br>• Nephrocalcinosis<br>• All-cause mortality	None

MAM = moderate acute malnutrition; SAM = severe acute malnutrition

[a] "Generally healthy" populations are defined as having ≤20% of the study population with disease at the study's baseline with the exception of the case-control study design. Nutrition deficiencies, overweight, and obesity are not considered diseases in this systematic review.

[b] Any definition for categorical growth and development outcomes associated with high levels of vitamin D intake or 25(OH)D concentrations, such as overweight or obesity (usually defined by BMI cut-off).

Methods

We followed the methods for conducting a systematic review outlined in the Institute of Medicine's Standards for Systematic Reviews[8] and reported the study results according to the Preferred Reporting Items for Systematic Reviews and Meta-Analyses (PRISMA) statement.[9] We organized the results into two chapters (I. Vitamin D: requirements; II. Vitamin D: upper limits) corresponding to the sections described in Key Questions and Study Eligibility Criteria above. A prospectively developed protocol was published on the International Prospective Register of Systematic Reviews, PROSPERO (https://www.crd.york.ac.uk/prospero/; registration number: CRD42020198843).

Literature Searches and Study Selection Process

We developed search strategies according to the KQs (see the PROSERO: CRD42020198843) and implemented the searches in MEDLINE®, Embase, and Cochrane Central databases to capture studies from the inception of these databases to the 2nd week of June 2020. All searches had no language restrictions but were limited to human studies. We performed reference mining for the included studies in the relevant authoritative reports and systematic reviews.[10] Finally, we rescreened the excluded and included full-text articles from the scoping review[2] using the systematic review study eligibility criteria (Tables 1-4).

Duplicated citations across databases were removed prior to the screening process. Titles and abstracts were screened by two independent investigators using the Rayyan app for systematic reviews.[11] Full-text articles of screened-in abstracts were retrieved and screened by one investigator according to the study eligibility criteria (Tables 1-4). All rejected articles were reviewed by a second investigator to confirm or refute their exclusion. Disagreements between the two investigators were adjudicated by a third investigator or by group consensus. A list of excluded studies and exclusion reasons are available upon request.

Data Extraction

To extract data from each included study, we created standardized data extraction forms comprising study design and population characteristics, study results for all outcomes of interest, and data that are required for the planned subgroup analyses (see Key Questions and Study Eligibility Criteria). Data were extracted by one investigator and spot-checked by another investigator.

Risk of Bias Assessment

Two independent assessors performed a risk of bias (ROB) assessment for each included study, and the disagreements were resolved via discussions between the two assessors.

We used the Cochrane Collaboration's tool (ROB 2.0)[12] to assess ROB for each included interventional study. Overall ROB was rated for individual interventional studies using the Cochrane criteria.[12] We used the Newcastle Ottawa Scale (NOS) to assess the ROB for included cohort, case-cohort, and nested case-control studies.[13] Several NOS prompting questions were tailored or defined for specific systematic review topics, including "ascertainment of exposure," "comparability of cohorts based on the design and analysis," and "adequacy of follow-up of cohorts." The "representative of exposed cohort" question was removed due to the broad nature of the populations of interest in this review. In addition, the NOS "ascertainment of exposure" question was modified in order to assess the validity and uncertainty of intake assessment, which is one of the unique challenges that should be

considered in nutrition-related systematic reviews.[14] In addition to the tailored prompting questions, the Selection and Outcome domains of the NOS were further adapted to assess ROB for included case-cohort and nested case-control studies. A new domain was also created to address the analyses recommended to reduce bias in these observational designs.[15-18]

Data Synthesis and Strength of Evidence Rating

Data were synthesized for each KQ and for each outcome separately. Summary tables were created to present key study features and results and to facilitate qualitative synthesis. The Grades of Recommendation, Assessment, Development, and Evaluation (GRADE) approach[19,20] was utilized to determine strength of evidence for each outcome. GRADE evidence profile tables[21] were used to present synthesized data for each KQ and can be found in the Supplement.

Meta-analysis

For vitamin D requirement KQ3 (What is the effect of vitamin D intake on serum 25(OH)D concentrations in children aged 0 to 4 years?), we performed random-effects meta-regression[22,23] to examine the intake-response associations across studies. No meta-analyses were performed for all other KQs due to large heterogeneity in exposure and outcome definitions or ascertainment methods across included studies.

Organization of this report

Results were organized into one of the two main sections: I. Vitamin D Requirements and II: Vitamin D Upper Limits. Within each main section, the results were then organized in the order of key questions.

Results

Literature search and study selection processes of this systematic review are summarized in **Figure 2**.

Figure 2. Literature search and study selection process. Legends: VDKQ = vitamin D requirement key question; VDUL = vitamin D upper limits

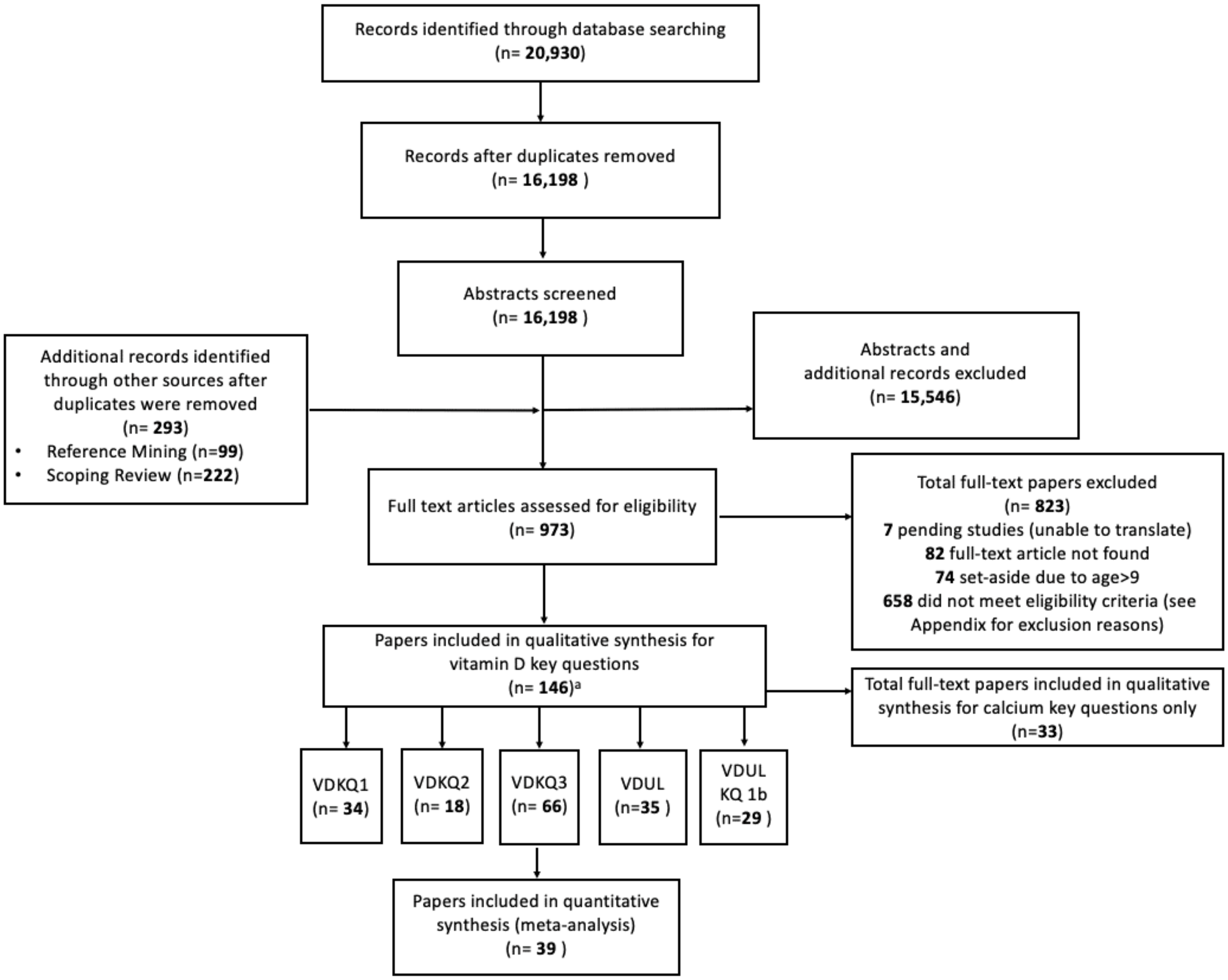

[a] The sum of papers for listed key questions is greater than 146, as some papers were included in more than one key question.

I. Vitamin D Requirements

KQ1. What is the effect of different levels of vitamin D intake on health outcomes in children aged 0 to 4 years?

A total of 34 interventional studies met the inclusion criteria for the effect of different levels of vitamin D intake on health outcomes in children aged 0 to 4 years. No interventional studies reported autoimmune disease or fracture as an outcome.

KQ1. Atopic Outcomes: Asthma, Wheezing, and Eczema.

Table KQ1-1 shows the characteristics and results of interventional studies reporting the effects of different levels of vitamin D intake on asthma, wheezing, and eczema outcomes. All studies were parallel randomized controlled trials (RCTs) in design with the number of randomized participants ranging from 195 to 987 in each trial. All trials were conducted in neonates and took place in the Northern and Southern Hemispheres. One trial was conducted in exclusively Black or African American preterm infants,[24] one with 100% of mothers of Northern European ethnicity,[25] and the other two trials (conducted in New Zealand and Australia) did not report race or ethnicity of the infants.[26,27] The majority of infants included in one trial were deficient in vitamin D at baseline.[25] All trials included an intervention arm of 400 IU/d of vitamin D_3 with other arms being 800 IU/d, 1,200 IU/d, or placebo. Intervention duration was six months for three studies and 12 months for the last study.

Figure KQ1-1 shows the summary ROB and individual study ROB ratings for the included studies in this section. Although all four trials have low ROB for measurement of the outcome, half of the trials have some or high ROB from at least one ROB domain including bias arising from the randomization process, bias due to deviations from intended interventions, bias due to missing outcome data, and bias in the selection of the reported results.

Asthma

The three RCTs that investigated the effects of vitamin D on asthma reported mixed results. One study found participants who received 400 IU/d of vitamin D_3 were at lower risk of developing asthma at six months compared to those who received placebo (RR = 0.055; 95% CI 0.003, 0.94).[26] However, there were no significant differences in the risk of asthma when comparing 800 IU/d of vitamin D_3 to placebo or when comparing 800 IU/d to 400 IU/d of vitamin D_3 supplementation. The other two RCTs that investigated the effects of vitamin D on asthma, both of which enrolled more participants for the same or longer duration, found no significant difference between groups.[24,25]

Wheeze

Two RCTs reported the effects of vitamin D supplementation on wheezing. One RCT comparing 400 IU/d of vitamin D_3 to placebo found no significant difference in the risk of developing wheezing at six months (RR = 1.08; 95% CI 0.06, 2.07).[27] In another RCT, Black or African American preterm infants who had achieved 200 IU/d of vitamin D from formula or human milk fortification were given additional supplementation or placebo.[24] Those given 400 IU/d vitamin D_3 until 6 months' adjusted age showed reduced risk of recurrent wheezing compared to the placebo group (adjusted RR = 0.62; 95% CI 0.44, 0.87).

Eczema

Three RCTs reported the effects of vitamin D supplementation on eczema. None found a significant difference in risk of eczema between groups (RR ranged from 0.74 to 1.12).[24,25,27]

Table KQ1-1. Characteristics and key findings of interventional studies reporting the effects of different levels of vitamin D intake on asthma, wheezing, and eczema outcomes

Authors (year)	Study design; N randomized	Enrollment years	Location; latitude	Mean age (SD) [range]	Male (%)	Breastfeeding status	Race or ethnicity	Health status; nutrition status	Vitamin D intervention groups [a]	Intervention duration	Comparison groups [b]	Key findings [c]
Grant et al. (2016) [26]	RCT; N= 260	2010-2011	Auckland, New Zealand; -36°	Neonates	46.5	Any BF	NR	100% Healthy; NR	VD3: 400 IU/d; 800 IU/d	6 months	Placebo	Asthma: RR=0.055 (0.003, 0.94) (400 IU/d vs. placebo); RR=0.35 (0.01, 1.25 (800 IU/d vs. placebo); RR=6.36 (0.32, 124.8) (800 IU/d vs. 400 IU/d)
Hibbs et al. (2018) [24]	RCT; N= 300	2013-2016	Cleveland, Charleston, and Bronx, US; ~38°	Neonates	55.3	Any BF	100% Black or African American	100% preterm	VD3 400 IU/d until 6 months' adjusted age (sustained group); VD3 400 IU/d until taking at least 200 IU/d of VD from formula or human milk fortifier (diet-limited group)	6 months	None	Asthma: RR=0.84 (0.41, 1.7)<br><br>Asthma or wheezing: RR=0.94 (0.65, 1.35)<br><br>Eczema: RR=0.82 (0.62, 1.088)

												Recurrent wheezing: adj. RR=0.62 (0.44, 0.87) [d]
Rosendahl et al. (2019) [25]	RCT; N= 987	2013-2014	Helsinki, Finland; 60°	Neonates	50.3	Any BF	100% of Mothers were northern European ethnicity	Generally healthy; 95.7% with vitamin D deficiency	VD3: 400 IU/d; 1200 IU/d	12 months	None	Asthma: 0.49 (0.02, 14.5) (1200 IU/d vs. 400 IU/d)<br><br>Eczema: 0.74 (0.53, 1.02) (1200 IU/d vs. 400 IU/d)
Rueter et al. (2019) [27]	RCT; N= 195	2012-2017	Perth, Australia; -32°	Neonates	53%	NR	NR	NR	VD3 400 IU/d	6 months	Placebo	Eczema: 1.12 (0.62, 2.02)<br><br>Wheeze: 1.08 (0.06, 2.07)

adj. = adjusted; BF = breastfeeding; d = day; IU = international units; N = sample size; NR = not reported; RCT = randomized controlled trial; RR = risk ratio; SD = standard deviation; VD = vitamin D; VD3 = vitamin D_3

[a] Administered in the form of a supplement unless otherwise stated.

[b] Comparison group: Non-vitamin D or non-calcium intervention group.

[c] Results compare higher dose groups to lower dose groups unless otherwise noted; Results are reported as: effect measure = effect size (95% confidence interval).

[d] Adjusted for randomization strata, time in study, gestational age, and the variables associated with recurrent wheezing in bivariate analysis.

Figure KQ1-1. Summary ROB plot (panel a) and individual study ROB (panel b) for trials examining the effects of different levels of vitamin D on wheezing, asthma, and eczema outcomes

a.

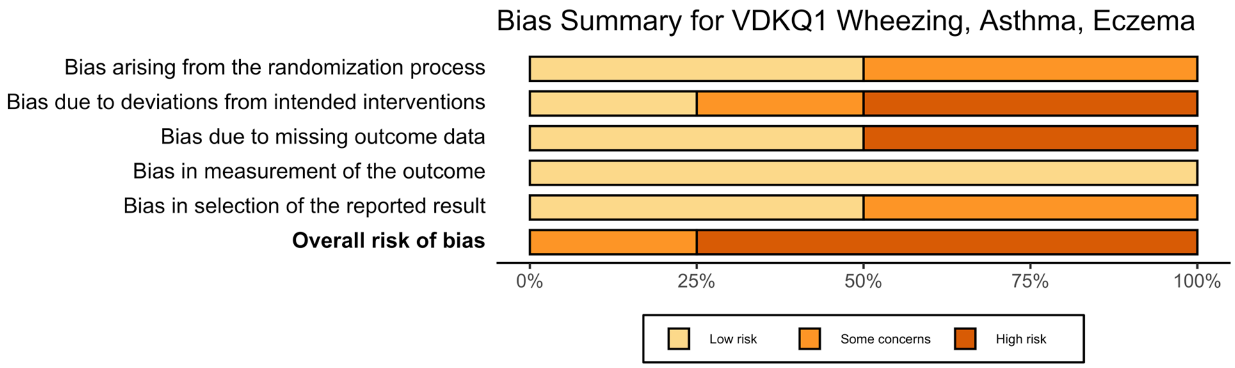

b.

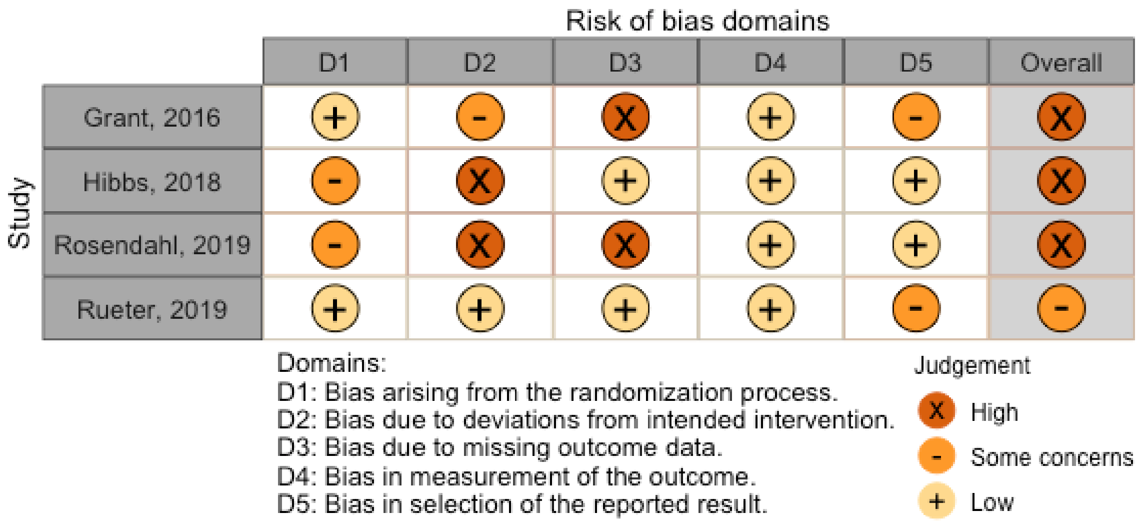

KQ1. Infectious Disease Outcomes

Table **KQ1-2** shows study characteristics of 8 interventional studies reporting the effects of different levels of vitamin D intake on infectious disease outcomes. All studies were RCTs that compared different levels of vitamin D3 (n = 4), compared vitamin D3 to controls receiving no vitamin D intervention (i.e, placebo or no intervention) (n = 2), or both (n = 2). The number of participants randomized ranged from 88 to 3,046 in each trial, with half the studies randomizing 300 or fewer infants. All studies were conducted in the northern hemisphere with latitudes ranging from ~29 to 60.2. In most studies, baseline health status of participants was either not reported and presumed healthy or generally healthy. One study included preterm infants with a mean gestational age of 33 weeks.[24] Nutritional status was not discussed in most studies; however, one study included exclusively malnourished infants,[28] and another study reported nearly all participants were deficient in vitamin D at baseline.[29] All study interventions included a daily regimen of vitamin D3, ranging from 400 to 1,200 IU/d, except for one group that received a bolus dose of 100,000 IU vitamin D3 once every three

months.[28] Intervention durations ranged from 4 to 23.5 months. Two follow-up studies reported different outcomes 12 months after the same trial consisting of a six-month intervention.[26,30] Almost all studies reported on respiratory infections with classification either by general anatomic location (e.g., upper respiratory tract, lower respiratory tract), specific anatomic location (e.g., pneumonia, bronchitis, otitis media), or causative agent (e.g., influenza A). Other reported infectious diseases included gastroenteritis, "other infections," or "all infections."

Figure KQ1-2 shows the summary ROB and individual study ROB for trials included in this section. Three-quarters of the included studies were prone to bias due to deviations from intended interventions. This was often due to high levels of non-adherence to the intervention or to the absence of statistical analyses assessing the effect of adherence to the intervention. Half of the studies were prone to bias due to missing outcome data with no evidence to suggest results were not biased due to missingness. Other potential bias arose from poorly described or inappropriate randomization processes and from a lack of evidence or indication that detailed analysis and statistical plans were pre-specified before unblinded outcome data were available.

The eight included studies collectively reported 20 total infectious disease outcomes, which included 15 respiratory infection outcomes, one gastroenteritis outcome, and four other or unspecified infectious disease outcomes. Of all 20 reported outcomes, 19 were not significantly different between intervention groups. One RCT found infants receiving 1,200 IU/d of vitamin D_3 were significantly less likely to develop influenza A over 4 months compared to infants receiving 400 IU/d of vitamin D_3 (RR = 0.54; 95% CI 0.42, 0.77).[31]

Table KQ1-2. Characteristics and key findings of interventional studies reporting the effects of different levels of vitamin D on infectious disease outcomes

Author (year)	Study design; N randomized	Enroll-ment years	Location; latitude	Mean age (SD) [range]	Male (%)	Breast-feeding status	Race or ethnicity	Health status; nutrition status	Vitamin D intervention groups [a]	Intervention duration	Comparison groups [b]	Key findings [c]
Aglipay et al. (2017) [32]	RCT; N= 703	2011-2015	Toronto, Canada; 43°	2.7 (1.5) years	57.4	Any BF	NR	100% Healthy; NR	VD3: 400 IU/d; 2000 IU/d	4 months	None	URTI: IRR=0.97 (0.80, 1.16)
Alonso et al. (2011) [33]	RCT; N= 88	2007-2008	Spain; 43°	Neonates	52.3	Any BF	NR	100% Healthy; NR	VD3: 402 IU/d	12 months	Without intervention (no placebo was used)	Infectious disease: "not significantly different between groups"
Grant et a. (2015) [30d]	RCT; N= 260	2010-2011	Auckland, New Zealand; -36°	Neonates	NR	Any BF	NR	NR; NR	VD3: 400 IU/d; 800 IU/d	6 months	Placebo	Healthcare visit for any respiratory infection: RR=0.87 (0.65, 1.16) (400 IU/d vs. placebo); RR=1.07 (0.82, 1.39) (800 IU/d vs placebo)<br><br>Healthcare visit for any other infection: RR=1.51 (0.82, 2.76) (400 IU vs placebo); RR=1.39 (0.74, 2.61)

												(800 IU vs placebo)
Grant et al. (2016) [26d]	RCT; N= 260	2010-2011	Auckland, New Zealand; -36°	Neonates	46.5	Any BF	NR	Generally healthy; NR	VD3: 400 IU/d; 800 IU/d	6 months	Placebo	Bronchiolitis: RR=1.00 (0.64, 1.65) (400 IU/d vs placebo); RR=0.86 (0.50, 1.48) (800 IU/d vs placebo)<br><br>Bronchitis: RR=0.43 (0.11, 1.60) (400 IU/d vs. placebo); RR=0.75 (0.25, 2.27) (800 IU/d vs. placebo)<br><br>Cold or influenza: RR=0.94 (0.65, 1.26) (400 IU/d vs. placebo); RR=1.02 (0.71, 1.47) (800 IU/d vs. placebo)<br><br>Croup: RR=1.29 (0.80, 2.08) (400 IU/d vs. placebo); RR=1.00

												(0.59, 1.70) (800 IU/d vs. placebo)<br><br>Otitis media: RR=0.86 (0.55, 1.34) (400 IU/d vs. placebo); RR=0.94 (0.61, 1.46) (800 IU/d vs. placebo)<br><br>URTI: RR=0.89 (0.73, 1.08) (400 IU/d vs. placebo); RR=0.90 (0.74, 1.09) (800 IU vs. placebo)<br><br>Wheezy lower respiratory infection: RR=0.89 (0.50, 1.59) (400 IU/d vs. placebo); RR=1.11 (0.64, 1.91) (800 IU/d vs placebo)
Hibbs et a. (2018) [24]	RCT; N= 300	2013-2016	Cleveland, USA; Charleston, USA; Bronx, NY, USA; ~38°	Neonates	55	Any BF	100% Black or African American	100% preterm (mean GA=33); NR	VD3: 400 IU/d	6 months	Placebo	URTI: RD=3.6 (-16.4, 4.4)<br><br>LRTI: RD=08.3 (-20.6, 2.7)

												Other infection: RD=-1.6 (-17.1, 7.0)
Manaseki-Holland et al. (2012) [28]	RCT; N= 3046	2008-2009	Kabul, Afghanistan; 34.6°	~0.54 [0.17-1] years	52	Any BF	Father ethnicity: Tajik, Pashton, Uzbek, Hazara	NR: Mal-nourished	VD3: 100,000 IU/3 mo	18 months	Placebo	Incidence of first pneumonia: IRR=1.07 (0.90, 1.27)
Rosendahl et al. (2018) [29]	RCT; N= 987	2013-2014	Helsinki, Finland; 60.2°	Neonates	50.3	Any BF	100% White	Generally healthy; 95.7% with vitamin D sufficiency	VD3: 400 IU/d; 1200 IU/d	23.5 months	None	Respiratory infection: IRR=1.00 (0.93, 1.07)<br><br>Gastroenteritis: IRR=0.92 (0.79, 1.08)<br><br>Other infections: IRR=1.04 (0.91, 1.19)<br><br>All infections: 1.00 (0.93, 1.06)
Zhou et al. (2018) [31]	RCT; N= 400	2015-2016	Yongkang, China; Wenzhou, China; Jinhua, China; ~29°	0.65 (0.22) years	52.3	Any BF	Presumed 100% Chinese	Generally healthy; Presumed normal	VD3: 400 IU/d; 1200 IU/d	4 months	None	Influenza A: RR=0.56 (0.42, 0.77)

BF = breastfeeding; d = day; GA = gestational age; IRR = incident rate ratio; IU = international units; LRTI = lower respiratory tract infection; N = sample size; NR = not reported; RCT = randomized controlled trial; RD = risk difference; RR = risk ratio; SD = standard deviation; URTI = upper respiratory tract infection; VD3 = vitamin D_3

[a] Administered in the form of a supplement unless otherwise stated.
[b] Comparison group: Non-vitamin D or non-calcium intervention group.
[c] Results compare higher dose groups to lower dose groups unless otherwise noted; Results are reported as: effect measure = effect size (95% confidence interval).
[d] Follow up to Grant et al. (2014) study.

Figure KQ1-2. Summary ROB plot (panel a) and individual study ROB (panel b) for trials examining the effects of different levels of vitamin D on infectious disease outcomes

a.

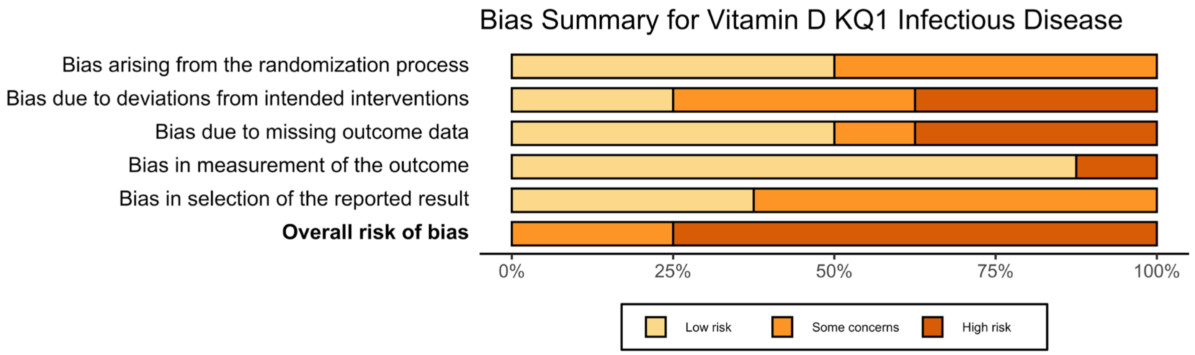

b.

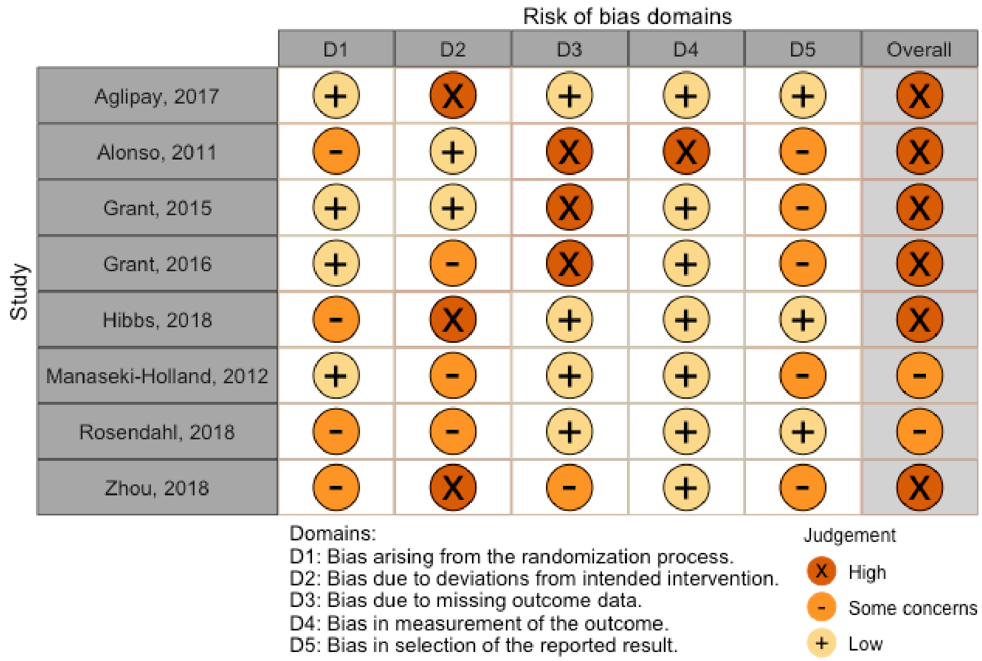

KQ1. Growth and Neurological Development Outcomes

The characteristics and results for 16 publications (13 unique studies, one ancillary study, and two follow-up studies) reporting on associations between vitamin D intake and growth or neurological development outcomes in children aged 0 to 4 years are presented in **Table KQ1-3**. Most studies were conducted at northern latitudes ranging from 29° to 46°. Two studies were conducted at 60° N,[34,35] and one study was conducted at 38° S.[36] Twelve of the 13 unique studies were RCTs while the other was a non-randomized controlled trial comparing certain growth outcomes.[37] Intervention durations varied widely from 2.5 to 23.5 months with approximately half the unique study interventions lasting eight or more months. Five unique studies compared various daily doses of vitamin D (400, 800, 1,200, and/or 1,600 IU),[34,35,38-41] one study compared a daily dose of 400 IU vitamin D with a bolus dose of 50,000 IU,[36] and one study compared a weekly dose of 1,400 IU vitamin D to a placebo.[42] Three studies compared human milk or infant formula supplemented with vitamin D (400 IU/d supplement or 400-427 IU/L formulations) to human milk alone[43,44] or with a placebo.[37] The remaining two studies included various combinations of vitamin D supplementation or placebo for both infants fed human milk (400 IU/d or placebo) and their lactating mothers (600 IU/d, 6,000 IU/d, 120,000 IU/month, or placebo).[45,46] Most studies recruited 100% healthy infants, but two studies enrolled preterm or low birth weight infants who were otherwise generally healthy.[39,42]

Growth and development outcomes for these studies were defined by measurements of the body and head. Body measurements included weight; body length or height; lean mass; fat mass; body fat percentage; leg length; and circumferences of the chest, arm (mid-arm or mid-upper arm), leg, waist, hip, and thigh. Head measurements included head circumference, occipitofrontal circumference, and anterior fontanelle maximum diameter. From these measurements, many studies calculated *Z* scores, standard deviation scores, or indices (body mass index, lean mass index, and/or fat mass index). Neurological development outcomes were defined by gross motor skills and measured in one ancillary study of six-month-old infants[47] and one follow-up study for participants under five years old.[48]

Growth and Development Results

Eleven of the 13 unique studies (85%) reported no association between vitamin D interventions and any growth and development outcomes ($P>0.05$). One study reported statistically significant benefits for length at 12 months of age in infants given a formula with 427 IU/L (range of intake was 382-480 IU/L) compared to a placebo ($P = 0.02$).[37] This study also showed a moderately significant benefit for length at 12 months when comparing infants fed human milk supplemented with 400 IU/d vitamin D to infants given human milk plus a placebo ($P<0.1$). There was no difference in weight and head circumference between groups. It is important to note that formula fed infants in this study were a non-randomized control group and that at six months of age, all randomized infants fed human milk (including the previous placebo group) were given 400 IU/d vitamin D until weaned or until the study ended when the infants were 12 months of age. Another study reported significant improvements in weight-for-age and length-for-age *Z* scores as well as for arm circumference at age six months for neonates assigned to a weekly dose of 1,400 IU vitamin D compared to a placebo ($P<0.05$).[42] According to the follow-up study, when participants were 3-6 years old, those originally assigned to the placebo group had significantly better BMI, BMI *Z* score, and arm muscle area measurements than those from the vitamin D group.[48] No other growth and development measurements showed significant associations with study group.

Neurological Development Results

One of the two studies measuring neurological development found an association with vitamin D intake and reported an inverse relationship.[47] This study reported that one-month-old infants assigned to 400 IUd vitamin D had statistically significantly higher total scores on the Alberta Infant Motor Scale (AIMS) at age 6 months compared to both the 800 IU/d and 1,200 IU/d groups ($P<0.05$). The 400 IU/d group also performed significantly better than the 800 IU/d group for the prone and sitting subscores and better than the 1,200 IU/d group on the sitting subscore ($P<0.05$). There were no group differences for the supine and standing subscores.

Risk of Bias Results

Results for the ROB assessment conducted for all 16 publications are presented in **Figure KQ1-3**. In all ROB domains, at least four studies were assessed as having some concern or high risk of bias, and for three domains, 12 or more studies (≥80%) had some concern or high risk of bias. The randomization process and measurement of the outcome domains showed the lowest risk for bias. In most studies, high dropout rates and a general lack of published pre-specified analysis plans or protocols resulted in some concern or high risk of bias for missing outcome data and selection of the reported results. Nearly all studies showed high risk of bias due to deviations from intended interventions. In most cases, low adherence rates and a failure to conduct analyses to detect effects of non-adherence led to risk of bias in this domain.

Table KQ1-3. Characteristics of vitamin D interventional studies that assessed growth and development and/or neurological development outcomes

Author (year)	Study design ; N enrolle d	Enroll ment years	Location; latitude	GA or mean age (SD) / median [range]	Male (%)	Breastf eeding Status	Race or ethnicity	Health status; nutritio nal status	Growth/dev elopment outcome(s) analyzed	Vitamin D or calcium intervention group	Interven tion duration (months)	Compariso n group(s)[a]	Key findings[b]
100% Healthy Infants													
Chan et al. (1982) [43]	RCT; N=91	NR	Salt Lake City, United States; 41°	GA: NR [38-41] weeks	NR	Any BF (mixed feeding)	White: 100%	100% Healthy; NR	Weight; length	VD + Ca: Infant formula with 400 IU/L (VD) + 51 mg/dl (Ca); VD: BF + 400 IU/d supp	12	BF only	Weight: 0 Length: 0
Chandy et al. (2016) [45]	RCT; N= 230	2012-2014	Lucknow, India; 26°	~0 (NR) days	NR	Any BF (mixed feeding)	100% Asian Indian	100% Healthy; NR	Weight; length; head cir.; chest cir.; anterior fontanelle max. diameter	VD3: BF + 400 IU/d (infant) and placebo (mother)	9	BF + placebo (infant) and 120,000 IU VD3/month (mother); BF + placebo (infant and mother)	All growth/ dev. outcomes (at 3.5 months): 0
Dawodu et al. (2019) [46]	RCT; N= 190	2013-2016	Doha, Qatar; 25°	≤4 (NR) weeks	NR	Exclusi ve BF	100% Ara b	100% Healthy; low vitamin D intake	Weight; length; head cir.	VD3: BF + 400 IU/d (infant) and 600 IU/d (mother)	6	BF + placebo (infant) and 6,000 IU VD3/d (mother)	Weight: 0 Length: 0 Head cir.: 0
Enlund-Cerullo et al. (2019) [34]	RCT; N=987	2013-2014	Helsinki, Finland; 60°	GA: 40.2 (1.1) weeks	50.3	Any BF (mixed)	White: 100 (Northern European)	100% Healthy; Normal	Weight; length-adj. weight SDS	VD3: 400 IU/d; 1,200 IU/d	23.5	None	Weight: 0 Length-adj. weight SDS: +
Gallo et al.	RCT; N=132 Follow	2007-2010	Montréal, Canada; 46°	~34.3 (95% CIs ranged	57.6 56.3 at 3-	Exclusi ve BF	White: 84.1%; Other:	100% Healthy; NR	WAZ; HAZ; HCAZ;	VD3: 400 IU/d; 800 IU/d; 1,200	11	None	All growth/ dev. outcomes (at 12 and 36

Author (year)	Study design; N enrolled	Enrollment years	Location; latitude	GA or mean age (SD) / median [range]	Male (%)	Breastfeeding Status	Race or ethnicity	Health status; nutritional status	Growth/development outcome(s) analyzed	Vitamin D or calcium intervention group	Intervention duration (months)	Comparison group(s)[a]	Key findings[b]
(2013)b [38]<br>Hazell et al. (2017)[c49]	-up study; N=87			from 31 to 38) days ~36.7 (1.1) months at 3-year follow-up	year follow-up		14.4% (includes Black, Hispanic, First Nations, Asian, Hawaiian/ Pacific Islander, and nonwhite mixed race)		36-month follow-up Height; HAZ; weight; WAZ; BMI; BAZ; lean mass; fat mass; body fat percentage; lean mass index; fat mass index	IU/d; 1,600 IU/d			months of age): 0
Greer et al. (1982) [37]	RCT; N=18<br>Non-randomized comparison group; N=12	1979	Madison, United States; 43°	GA: NR [38-40] weeks (reported for RCT groups only)	44.4 (reported for RCT groups only)	Any BF (mixed feeding)	White: 83%; Asian-Indian: 3%; Non-White: 13%	100% Healthy; NR	Weight; length; head cir.	VD2: BF + 400 IU/d supp;<br>VD (Non-randomized comparison group): Infant formula with an average of 427 IU/L [range 382-480 IU/L]	6-12[d]	BF + placebo	Weight (at 12 months): 0<br>Length (at 6 months): VD2 supp > formula > placebo (P-value or 95% CI NR)<br>Length (at 12 months): ++ (formula vs. placebo), + (VD2 supp vs. placebo), 0 (formula vs. VD2 supp)<br>Head cir. (at 12 months): 0
Holmlund-Suila et al.	RCT; N=113	2010-2011	Helsinki, Finland; 60°	GA: ~40.4 (~0.8-1.3) weeks	50.4	Any BF (mixed feeding)	NR	100% Healthy; NR	Weight; length; head cir.; leg	VD3: 400 IU/d; 1,200 IU/d; 1,600 IU/d	2.5	None	All growth/ dev. outcomes (at age 3 months): 0

Author (year)	Study design; N enrolled	Enrollment years	Location; latitude	GA or mean age (SD) / median [range]	Male (%)	Breastfeeding Status	Race or ethnicity	Health status; nutritional status	Growth/development outcome(s) analyzed	Vitamin D or calcium intervention group	Intervention duration (months)	Comparison group(s)[a]	Key findings[b]
(2012)[35]									length; leg cir.				
Huynh et al. (2017)[36]	RCT; N= 70	2013-2014	St. Albans, Australia; -38°	GA: 39 (1.1-1.2) / 40 [IQR: 1.2-1.3] weeks; ~0 [0-2] days at enrollment	NR	Any BF (mixed feeding	NR	100% Healthy; Normal	Weight; length; head cir.	VD3: 400 IU/d; 50,000 IU bolus	4	None	Weight (at 3-4 months of age): + (400 IU/d > 50,000 bolus)<br>Length: 0<br>Head cir.: 0
Singh et al. (2018)[44]	RCT; N= 100	2013-2014	New Delhi, India; 29°	GA: 38.2 (0.82-0.87) weeks; ~0 [0-2] days at enrollment	55	Exclusive BF	100% Asian Indian	100% Healthy; 60% (intervention group) and 34% (control group) were VD deficient at birth	Head cir.; length; weight; mid-arm cir.	VD3: BF + 400 IU/d	6	BF only	Head cir.: 0<br>Length: 0<br>Weight: 0<br>Mid-arm cir.: -
Wagner et al. (2006)[41]	RCT; N= 19	NR	Charleston, South Carolina, United States; 33°	GA: ~39.0 (~0.7-1.2)	47	Exclusive or fully BF	White: 79%<br>Hispanic: 11%<br>Black: 11%	100% Healthy; NR;	BMI; weight; length; head cir.	Maternal VD3: 400 IU/d; infant BF with 300 IU/d supplement	6	Maternal VD3: 6400 IU/d; infant BF, not supplemented	BMI: 0<br>Weight: 0<br>Length: 0<br>Head cir.: 0
Wicklow et al. (2016)[e47]	RCT; N= 55	2009-2011	Montreal, Canada; 46°	1 (NR) month	56.4	Any BF (mixed)	White: 87.3%<br>Other: 12.7%	100% Healthy; Normal	WAZ; LAZ; HCAZ; WLZ; Gross motor skills (AIMS total and	VD3: 400 IU/d, 800 IU/d, 1,200 IU/d	11	None	At 6 months<br>WAZ: 0<br>LAZ: 0<br>HCAZ: 0<br>WLZ: 0

Author (year)	Study design; N enrolled	Enrollment years	Location; latitude	GA or mean age (SD) / median [range]	Male (%)	Breastfeeding Status	Race or ethnicity	Health status; nutritional status	Growth/development outcome(s) analyzed	Vitamin D or calcium intervention group	Intervention duration (months)	Comparison group(s)[a]	Key findings[b]
									subscores)[f]; all measures taken at 6 months of age				AIMS total score: -- (800 IU/d vs. 400 IU/d; 1,200 IU/d vs. 400 IU/d)<br>AIMS prone: -- (800 IU/d vs. 400 IU/d)<br>AIMS supine: 0<br>AIMS sitting: -- (800 IU/d vs. 400 IU/d; 1,200 IU/d vs. 400 IU/d)<br>AIMS standing: 0
Ziegler et al. (2014) [40]	RCT; N= 213	2006-2010	Iowa City, United States; 41°	1 (NR) month	NR	Exclusive BF to 4 months of age	Native: 2.3%; Asian: 0.9%; Black: 2.8%; Native: 0 White: 89.7%; Hispanic: 4.2%	100% Healthy; low 25(OH) D levels	Weight; length	VD3: 200 IU/d, 400 IU/d, 600 IU/d, 800 IU/d	8	None	Δ weight (4-9 months): 0<br>Δ length (4-9 months): 0
Generally Healthy Infants with Low Birth Weight (>1.5 kg) and/or Preterm Birth (≥32 gestation weeks)													
Kumar et al. (2011) [42]<br>Trilok-Kumar et al.	RCT; N=2,079<br>N=912 at 3-6 year	2007-2010	New Delhi, India; 29°	~2.0 [0-2] days at enrollment<br>5.0 (1.0) years at 3-	46.7 at enrollment<br>47.9 at 3-6 year	NA at follow-up	Presumed 100% Asian Indian	100% with low birthweight (1.8-2.5 kg) and at-risk for	WAZ; LAZ; weight/length z-score; head cir.; arm cir.<br>At 3-6 year follow-up	VD3: 1,400 IU/week	6	Placebo	WAZ[i]: ++<br>LAZ[i]: ++<br>Weight/length Z score[i]: 0<br>Head cir.[i]: -<br>Arm cir.[i]: ++

Author (year)	Study design; N enrolled	Enrollment years	Location; latitude	GA or mean age (SD) / median [range]	Male (%)	Breastfeeding Status	Race or ethnicity	Health status; nutritional status	Growth/development outcome(s) analyzed	Vitamin D or calcium intervention group	Intervention duration (months)	Comparison group(s)[a]	Key findings[b]
(2015)[g] [48]	follow-up			6 year follow-up	follow-up			infant mortality; 100% severely VD deficient at enrollment ~50% were VD deficient at 3-6 year follow-up	Weight; WAZ; height; HAZ; BMI; BMIZ; waist circ; hip cir.; MUAC; thigh cir.; triceps skinfold; subscapular skinfold; arm muscle area; gross motor dev.[h] (ASQ score) only for children <5 years old (N=571)				At 3-6 year follow-up<br>BMI[j]: --<br>BMIZ[j]: --<br>MUAC[j]: -<br>Thigh cir.[j]: -<br>Arm muscle area[j]: --<br>All other growth/dev. measures[j]: 0<br>ASQ Score[j]: 0
Natarajan et al. (2014) [39]	RCT; N=96	2011-2012	North India; ~29°	3.0 [~1-14] days	56.3	Any BF (mixed feeding)	Presumed 100% Asian Indian	Preterm infants (mean GA=32.5 weeks); VD deficiency at birth	Weight; length; OFC	VD3: 400 IU/d; 800 IU/d	3	None	Weight: 0<br>Length: 0<br>OFC: 0

adj. = adjusted; AIMS = Alberta Infant Motor Scale; ASQ = Ages and Stages Questionnaire; BAZ = BMI-for-age *Z* score; BF = breastfeeding; BMI = body mass index; BMIZ = BMI *Z* score; Ca = calcium; cir. = circumference; CI = confidence interval; d = day; dev. = development; GA = gestational age; HAZ = height-for-age *Z* score; HCAZ: head circumference-for-age *Z* score; IU = international units; LAZ = length-for-age *Z* score; MUAC = mid-upper arm circumference; N = number of participants; NA = not applicable; NR = not reported; OFC = occipitofrontal circumference; RCT = randomized controlled trial; SDS = standard deviation scores; supp = supplement; VD = vitamin D; VD3 = vitamin D_3; WAZ = weight-for-age *Z* score; WLZ = weight-for-length *Z* score

[a] Comparison group: Non-vitamin D or non-calcium intervention group.

[b] Results compare higher dose groups to lower dose groups unless otherwise noted: ++ Significant beneficial effects ($p < 0.05$); + Marginally significant beneficial effects ($0.05 < p < 0.1$); 0 No effects; - Marginally significant detrimental effects ($0.05 < p < 0.1$); -- Significant detrimental effects ($p < 0.05$); results=0 means there were no significant differences observed for any outcomes of interest.
[c] Follow-up to the Gallo et al. (2013)b[38] study conducted 3 years after enrollment.
[d] At age 6 months, infants randomized to the placebo group were also given 400 IU VD/d after which all randomized infants received supplementation until weaned from breastfeeding or until age 12 months when the study ended.
[e] Ancillary study to Gallo et al. (2013)b.[38]
[f] Assessed with the Alberta Infant Motor Scale (AIMS) which includes a total score and four subscores: prone, supine, sitting, and standing.
[g] Follow-up to the Kumar et al. (2011) [42] study conducted when participants were 3-6 years of age.
[h] Assessed with the Ages and Stages Questionnaire (ASQ), second edition.
[i] Results from multivariate linear regression models adjusting for baseline anthropometric z score (except for arm circumference where birth arm circumference was used instead), sex, quintile of socioeconomic status, family type, maternal education, exposure to sunlight, and breast feeding for more than six months.
[j] Results from multivariate linear regression models adjusted for age at follow-up, family size (total number of adults + children in the household), family type, socioeconomic status at baseline, maternal and paternal education (factors associated with being lost to follow-up) for *Z* scores and ASQ score; other variables also adjusted for sex.

Figure KQ1-3. Summary ROB plot (panel a) and individual study ROB (panel b) for vitamin D intervention studies reporting on growth or neurological development outcomes

a.

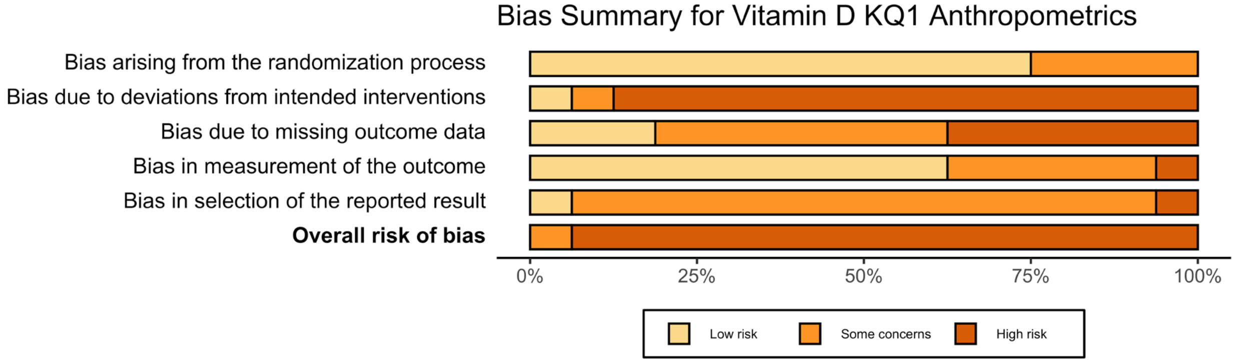

b.

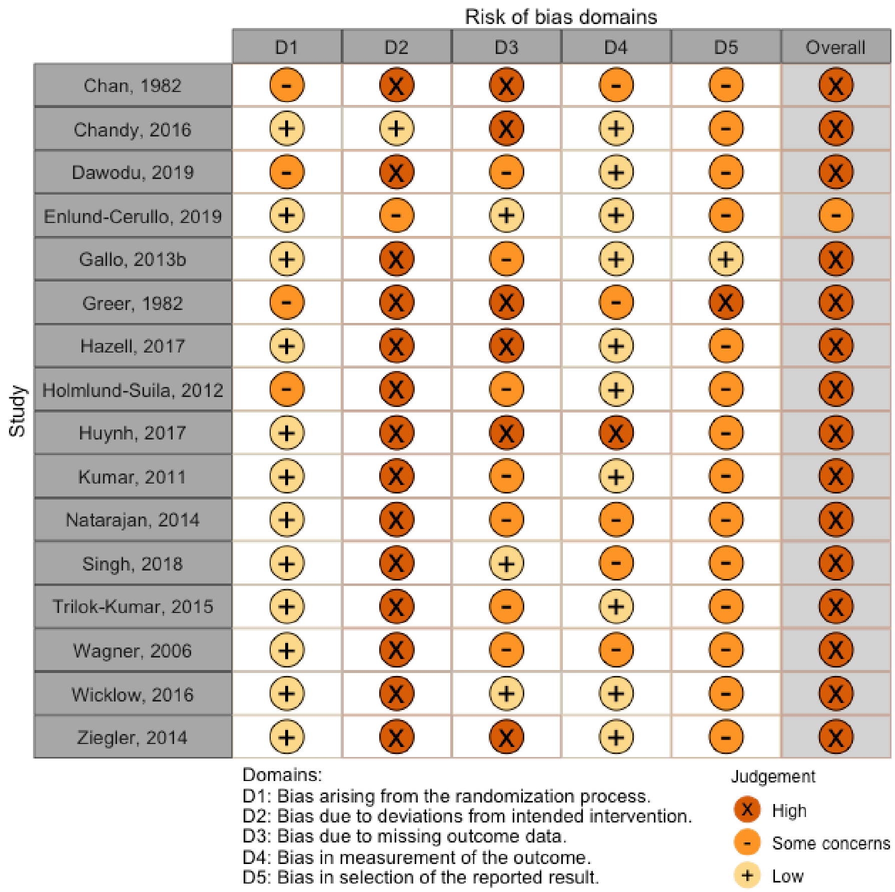

KQ1. Rickets

Nine interventional studies conducted in children (0 to 4 years old) reported on the association between vitamin D supplementation and the development of rickets. **Table KQ1-4** presents each study's definition of rickets, although most studies failed to provide much detail on how rickets was determined and by whom (e.g., physician or research staff). Eight of these studies (89%) were RCTs while one was a non-randomized controlled trial.[50] Study sample sizes ranged from 18 to 2,079 participants, and all studies were conducted between latitudes of 29° and 61° north. Two of these studies included only exclusively human milk fed infants,[51,52] while the rest (78%) studied infants with a mix of feeding styles (i.e., human milk, formula, and/or solid foods). Four studies compared participants assigned to a vitamin D intervention to a placebo group or participants with no intervention.[24,33,37,42] Two studies compared multiple vitamin D intervention arms with a placebo or no intervention group.[51,53] One study compared infants assigned to calcium, vitamin D, or combined supplementation (calcium + vitamin D) with a group receiving no intervention.[50] Two studies compared two vitamin D arms.[52,54] Most of these studies assigned vitamin D interventions as daily doses ranging from 200 to 1,000 IU/d. One study assigned newborns to 1,400 IU vitamin D per week while another study assigned older infants (mean age of 2.26 years) to a dose of 25,000 IU per month. Intervention durations ranged from 1.5 to 36 months.

Only one study reported any clinical cases of rickets.[50] This study found no effect of supplementation on the prevalence of rickets ($P = 0.214$). It is important to note that 1.25% of participants (n ≈ 2) had severe rickets (defined as more than three signs of rickets) after one year in the study while 1.33% (n ≈ 2) had severe rickets at the end of the study. The highest reported prevalence of rickets in this study was 1.77% (n ≈ 3) at the end of study year two.

For most of these studies, all five domains of the ROB tool presented some concern for bias (**Figure KQ1-4**). While just one of these studies was not randomized,[50] the others generally did not report details on randomization processes or efforts to conceal allocation sequences until patient enrollment and assignment. In several studies, there was some concern for bias due to deviations from intended interventions resulting from low levels of participant adherence to supplementation protocols and no analysis of low adherence impact. The amount of missing data for the rickets outcome in these studies resulted in some concern for bias in Domain 3. Most studies had dropout or loss to follow-up rates higher than 90-95% likely due to the long study durations, but few studies reported the impact of that missing data on outcomes assessment. In terms of outcome measurement bias, there was less concern, but the lack of details on rickets evaluation led to some concern for bias. Finally, due to a lack of reporting on analysis plans for rickets outcomes by all nine studies, there was some concern for bias in selection of reported results.

Table KQ1-4. Characteristics of vitamin D interventional studies that assessed rickets

Author (year)	Study design; N enrolled	Enrollment years	Location; latitude	GA or mean age (SD) / median [range]	Male (%)	Breastfeeding Status	Race or ethnicity	Health status; nutritional status	Vitamin D or calcium intervention group(s)	Intervention duration (months)	Comparison group(s)[a]	Rickets definition
Ala-Houhala et al. (1985) [51]	RCT; N=92	1982	Tampere, Finland; 61°	0 (0) days	NR	Exclusive BF	Presumably 100% Finnish	100% Healthy; NR	VD: BF + 400 IU/d supp, BF + 1000 IU/d supp	5	BF + 1,000 IU VD/d (mother)	Biochemical signs determined by mineral and AP levels
Alizade et al. (2006) [54]	RCT; N=68	2001-2002	Tehran, Iran; 36˚	GA: ~32.7 (28-37) weeks	50	NR	NR	GA 29-38 weeks, birth weight 1500-2000 g; NR	VD: 400 IU/d; 1000 IU/d	2	None	Physical examination and x-ray analysis for signs and symptoms (craniotabes, rickets rosary, wide fontanel, Harrison groove, kyphosis/scoliosis, Potts belly)
Alonso et al. (2011) [33]	RCT; N=102	2007 - 2008	Northern Spain; 43°	1 (NR) month	52.2	Any BF (mixed feeding)	Presumably 100% Spanish	100% Healthy; NR	VD: 402 IU/d	12	No intervention	Determined by physical examination
Greer et al. (1982) [37]	RCT; N=18	1979	Madison, United States; 43°	GA: NR [38-40] weeks	44.4	Any BF (mixed feeding)	White: 94.4%; Asian-Indian: 5.6%	100% Healthy; NR	VD2: BF + 400 IU/d supp	6-12[b]	BF + placebo	Clinical signs including craniotabes, rachitic rosary, or widened wrists
Hibbs et al. (2018) [24]	RCT; N=300	2013-2016	Cleveland, Charleston, and Bronx, United States; 41°, 33°, 41°	~12.0 [IQR ~6-21] days	55.3	Any BF (mixed feeding)	Black or African American: 100 (~7.6% of all parents reporting race in the	Preterm infants (mean GA=33 weeks); Normal	VD3: 400 IU/d [sustained]	6	Placebo [diet-limited]	Diagnosis

Author (year)	Study design; N enrolled	Enrollment years	Location; latitude	GA or mean age (SD) / median [range]	Male (%)	Breastfeeding Status	Race or ethnicity	Health status; nutritional status	Vitamin D or calcium intervention group(s)	Intervention duration (months)	Comparison group(s)[a]	Rickets definition
							study were Hispanic)					
Kumar et al. (2011) [42]	RCT; N=2,079	2007-2010	New Delhi, India; 29°	~2.0 [0-2] days	46.7	Any BF (mixed feeding)	Presumed 100% Asian Indian	100% low birthweight (1.8-2.5 kg) infants at-risk for infant mortality; 100% severely VD deficient	VD3: 1,400 IU/week	6	Placebo	Determined by physical examination
Ponnapakkam et al. (2010) [53]	RCT; N=80	NR	Southern Louisiana, United States; ~30°	0 (0) [0] years	NR	Any BF (mixed feeding)	NR	Generally healthy (<20% disease); Normal	VD3: 200 IU/d from birth, 200 IU/d from age 2 months	6	Placebo	Elevated AP levels and evidence of rachitic changes on hand X-rays
Rooze et al. (2016) [50]	Non-randomized trial; N=207	2010	Lhasa, Tibet; 30°	2.26 (NR) [0-5] years	48	Any BF (mixed feeding)	Presumed 100% Tibetan	Generally healthy (<20% disease); NR	Ca: 15 mmol/d<br>VD: 25,000 IU/month<br>VD + Ca: 25,000 IU/month (VD), 15 mmol/d (Ca)	36	No intervention	Open fontanelle after 1 year of age, craniotabes, frontal bossing, enlargement of the wrists, bowing of the legs, Harrison's groove, pigeon chest, or costochondral swelling
Siafarikas et al. (2011) [52]	RCT; N=40	NR	Berlin, Germany; 53°	GA: 39 [39-40] weeks	NR	Exclusive BF	Presumed 100% German	100% Healthy; VD insufficiency	VD3: 250 IU/d; 500 IU/d	1.5	None	Determined by physical examination

AP = alkaline phosphatase; BF = breastfeeding; Ca = Calcium; d = day; GA = gestational age; IQR = interquartile range; IU = international units; N = sample size; NR = not reported; RCT = randomized controlled trial; SD = standard deviation; supp = supplement; VD = vitamin D; VD3 = vitamin D_3

[a] Comparison group: Non-vitamin D or non-calcium intervention group.

[b] At age 6 months, infants randomized to the placebo group were also given 400 IU VD/d after which all randomized infants received supplementation until weaned from breastfeeding or until age 12 months when the study ended.

Figure KQ1-4. Summary ROB plot (panel a) and individual study ROB (panel b) for vitamin D interventional studies that assessed rickets as an outcome

a.

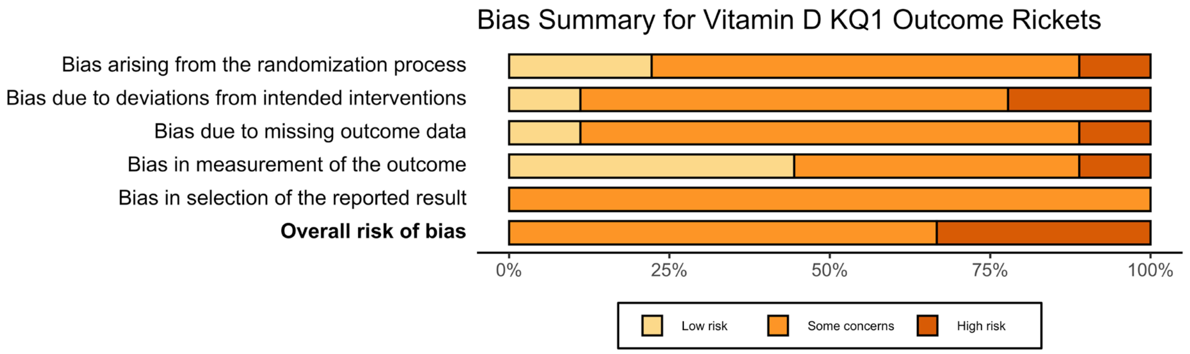

b.

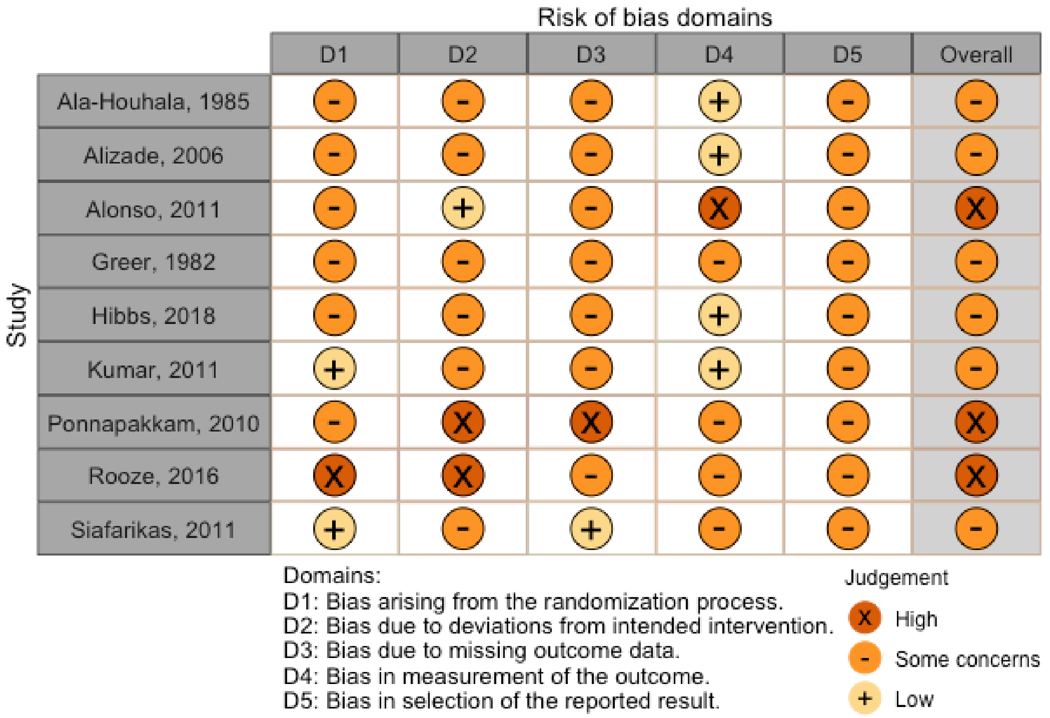

KQ1. Bone Mineral Content and Bone Mineral Density Outcomes

Table KQ1-5 shows characteristics and results for 10 unique vitamin D interventional studies in our review that assessed outcomes related to bone mineral content (BMC) or bone mineral density (BMD). Sample size at enrollment ranged from 30 to 2,079, but seven of these studies (67%) had sample sizes below 200. Eight studies (80%) were RCTs and 1 was a non-randomized trial.[55] The remaining study randomized human milk fed infants to either a vitamin D or placebo intervention for the first 6 months of life, and then all were given vitamin D supplementation until weaned.[37] This study also included a non-randomized group of formula fed infants as a comparison group for bone mineral outcomes only. All these interventional studies were conducted in the northern hemisphere with latitudes ranging from 26° to 60°. Two studies compared a single vitamin D dosing group to a placebo group,[24,48] three compared infants fed human milk or formula fed with a vitamin D supplement to a group fed human milk with no supplement,[37,43,55] and the remaining five studies compared groups with different doses of VD.[29,35,38,39,56] For most of these studies, vitamin D dosing was given as a daily regimen of 400 IU to 1,600 IU, while one study used a dose of 1,400 IU per week.[48] Two studies did not specify total daily vitamin D dose for study groups given infant formula but reported vitamin D IU per liter of formula.[37,43] In all 10 studies, intervention duration was 2.5 months or greater with the longest duration at 23.5 months. Three of the included studies were long term post-intervention follow-up studies of RCTs with follow-up duration ranging from 3 months to 6 years.[48,55,57]

Other than general BMD and BMC measurements, bone structure and strength outcomes reported in these studies included the following: speed of sound (SOS), bone transmission time (BTT), stress and strain index (SSI), cross-sectional area of the bone (CSA), and polar moment of inertia. Areas of measurement specified in the studies included metacarpal,[55] radius,[37,43,48] ulna,[37] femur,[38] tibia,[24,29,35,48] lumbar spine vertebrae,[38,57] and whole body.[38,39,57] Five studies reported no difference in BMD/BMC outcomes when comparing vitamin D supplementation to human milk only[43] or when comparing groups with different vitamin D supplement doses (1,600, 1,200, 800, and 400 IU/d;[38] 800 and 400 IU/d;[39] 1,200 and 400 IU/d;[29] 4,000 IU/d and 30,000 IU/wk[56]). Two studies reported benefits to BMC/BMD outcomes when comparing 400 IU/d vitamin D supplementation with a placebo but did not report p-values or confidence intervals.[24,37] One study reported statistically significant ($P<0.05$) benefits for most BMC/BMD measurements when comparing the highest dose of vitamin D with lower doses (1,600 IU/d vs. 400 IU/d; 1,600 IU/d vs. 1,200 IU/d).[35] Another study reported statistically significant ($P<0.05$) benefits for BMC/BMD measures when comparing vitamin D supplementation in human milk or formula fed infants with human milk alone.[55] One study reported moderately significant ($0.05 < P < 0.1$) benefits for distal radius (but not tibia) measurements when comparing 1,400 IU vitamin D per week to a placebo.[48]

Figure KQ1-5 presents summary results for the ROB assessment for all 10 vitamin D intervention studies that reported BMC and BMD outcomes. In all studies, there was a low ROB for how the BMC and BMD outcomes were measured, but there was a high risk of bias or some concern due to deviations from intended interventions. Risk of bias in this latter domain generally resulted from low adherence to daily supplementation by participants or a lack of analysis conducted in the study to account for the effect of adherence. For over half of these studies (60%), there was some concern with the randomization process and the reporting of results – generally owing to no publicly available protocols showing pre-specified analysis plans. Finally, due to missing outcome data, three studies had some concern for bias, and three had high risk of bias. These ratings were likely a product of long intervention and follow-up durations that increase the possibility for participant drop-out and loss to follow-up.

Table KQ1-5. Characteristics and key findings of vitamin D interventional studies with bone mineral density or bone mineral content outcomes reported

Author (year)	Study design; N enrolled	Enrolment years	Location; latitude	GA or mean age (SD) / median [range]	Male (%)	Breast feeding status	Race or ethnicity	Health status; nutritional status	BMD/BMC outcome analyzed	Vitamin D or calcium intervention group(s)	Intervention duration (months)	Comparison group(s) [a]	Key findings[b]
Bagnoli et al. (2013) [55]	Non-randomized Trial; N=73 (at 3-month follow-up)	NR	Siena, Italy; 43°	GA: ~39.4 (~1.3-1.7) weeks at enrollment; 3.0 (NR) months at follow-up	49.3	Any BF (mixed feeding)	NR	100% Healthy; NR	Metacarpal SOS (m/sec); Metacarpal BTT (µsec); used four metacarpal measurements	VD: BF + 400 IU/d supp [BFD]; Formula milk with or without 400 IU/d supp [FF]	3	BF only [BF]	SOS: ++ (BFD vs. BF; FF vs. BF), 0 (BFD vs. FF) BTT: ++ (BFD vs. BF; FF vs. BF), 0 (BFD vs. FF)
Chan et al. (1982) [43]	RCT; N=91	NR	Salt Lake City, United States; 41°	GA: NR [38-41] weeks	NR	Any BF (mixed feeding)	White: 100%	100% Healthy; NR	BMC from distal left radius	VD + Ca: Infant formula with 400 IU/L (VD) + 51 mg/dl (Ca); VD: BF + 400 IU/d supp	12	BF only	BMC: 0
Gallo et al. (2013)b [38] Gallo et al. (2016)[c] [57]	RCT; N=132 N=87 in follow-up study	2007-2010	Montréal, Canada; 46°	~34.3 (NR) days ~36.7 (NR) months at follow-up study	57.6	Exclusive BF	White: 84.1%; Other: 14.4% (includes Black, Hispanic, First Nations, Asian, Hawaiian/Pacific Islander, and nonwhite mixed race)	100% Healthy; NR	BMC of the whole body, lumbar spine vertebrae 1-4, and whole femur; BMD of lumbar spine (BMC of femur NR in follow-up study)	VD3: 400 IU/d; 800 IU/d; 1200 IU/d; 1600 IU/d	11	None	BMC (at 12 and 36 months of age): 0 BMD (at 12 and 36 months of age): 0

Author (year)	Study design; N enrolled	Enrolment years	Location; latitude	GA or mean age (SD) / median [range]	Male (%)	Breastfeeding status	Race or ethnicity	Health status; nutritional status	BMD/BMC outcome analyzed	Vitamin D or calcium intervention group(s)	Intervention duration (months)	Comparison group(s) [a]	Key findings[b]
Greer et al. (1982) [37]	RCT; N=18<br><br>Non-randomized comparison group for BMC outcome; N=12	1979	Madison, United States; 43°	GA: NR [38-40] weeks (reported for RCT groups only)	44.4 (reported for RCT groups only)	Any BF (mixed feeding)	White: 83%; Asian-Indian: 3%; Non-White: 13%	100% Healthy; NR	BMC of the one-third distal radius and ulna of the left hand	VD2: BF + 400 IU/d supp<br><br>VD (Non-randomized comparison group): Infant formula with an average of 427 IU/L [range 382-480 IU/L]	6-12[d]	BF + placebo	BMC (at 6 months): 0 (VD2 supp vs. placebo), + or ++ (formula vs. placebo reported as "significant"; P-value or 95% CI NR) BMC (at 12 months): formula > placebo > VD2 supp (P-value or 95% CI NR)
Hibbs et al. (2018) [24]	RCT; N=300	2013-2016	Cleveland, Charleston, and Bronx, United States; 41°, 33°, 41°	~12.0 [IQR ~6-21] days	55.3	Any BF (mixed feeding)	Black or African American: 100 (~7.6% of all parents reporting race in the study were Hispanic)	Preterm infants (mean GA=33 weeks); Normal	Tibial SOS	VD3: 400 IU/d [sustained]	6	Placebo [diet-limited]	SOS (at 12 months)[c]: sustained > diet-limited (P-value or 95% CI NR)

Author (year)	Study design; N enrolled	Enrolment years	Location; latitude	GA or mean age (SD) / median [range]	Male (%)	Breastfeeding status	Race or ethnicity	Health status; nutritional status	BMD/BMC outcome analyzed	Vitamin D or calcium intervention group(s)	Intervention duration (months)	Comparison group(s) [a]	Key findings[b]
Holmlund-Suila et al. (2012) [35]	RCT; N=113	2010-2011	Helsinki, Finland; 60°	GA: ~40.4 (~0.8-1.3) weeks	50.4	Any BF (mixed feeding)	NR	100% Healthy; NR	CALCBD (density and area; total and trabecular bone); CORTBD (density and area); Polar SSI; Site for analyzed measurements was at 65% of the length of the left tibia	VD3: 400 IU/d; 1,200 IU/d; 1,600 IU/d	2.5	None	CALCBD area[f]: + (overall); ++ (1,600 IU/d vs. 400 IU/d); 0 (1,600 IU/d vs. 1,200 IU/d; 1,200 IU/d vs. 400 IU/d) CALCBD density[f]: 0 CORTBD area[f]: + (overall); ++ (1,600 IU/d vs. 400 IU/d; 1,600 IU/d vs. 1,200 IU/d); 0 (1,200 IU/d vs. 400 IU/d) CORTBD density[f]: 0 Polar SSI[f]: + (overall); ++ (1,600 IU/d vs. 400 IU/d; 1,600 IU/d vs. 1,200 IU/d); 0 (1,200 IU/d vs. 400 IU/d)

Author (year)	Study design; N enrolled	Enrolment years	Location; latitude	GA or mean age (SD) / median [range]	Male (%)	Breastfeeding status	Race or ethnicity	Health status; nutritional status	BMD/BMC outcome analyzed	Vitamin D or calcium intervention group(s)	Intervention duration (months)	Comparison group(s) [a]	Key findings[b]
Natarajan et al. (2014) [39]	RCT; N=96	2011-2012	North India; ~29°	3.0 [~1-14] days	56.3	Any BF (mixed feeding)	Presumed 100% Asian Indian	Preterm infants (mean GA=32.5 weeks); VD deficiency at birth	BMC; BMD; Measurements were for whole body	VD3: 400 IU/d; 800 IU/d	3	None	BMC: 0<br>BMD: 0
Rao et al. (2016) [56]	RCT; N=45	NR	Kanpur, India; 26°	~3.1 (~0.7-0.9) [2-5] years	NR	NR	Presumed 100% Asian Indian	NR; 100% with VD deficiency (<20 ng/mL)	BMC; BMD; Measurements were for total body less head	VD + Ca: 4000 IU/d VD3 + 50 mg/kg/d Ca<br><br>VD + Ca: 30,000 IU/d VD3 + 50 mg/kg/d Ca	3	None	BMC: 0<br>BMD: 0
Rosendahl et al. (2018) [29]	RCT; N=987	2013-2014	Helsinki, Finland; 60°	GA: 40.2 (1.1) weeks	50.3	Any BF (mixed feeding)	White: 100 (Northern European)	100% Healthy; Normal	BMC; BMD; CSA; Polar moment of inertia; All measurements were of the left tibia at 20% distal proximal length	VD3: 400 IU/d; 1200 IU/d	23.5	None	BMC: 0<br>BMD: 0<br>CSA: 0<br>Polar moment: 0
Trilok-Kumar et al. (2015) [48]	RCT; N=2,079 (N=912 at 3-6 year follow-up)	2007-2010	New Delhi, India; 29°	~2.0 [0-2] days at enrollment; 5.0 (1.0) years at follow-up	46.7 at enrolment; 47.9 at	NA at follow-up	Presumed 100% Asian Indian	Low birth weight (1.8 to <2.5 kg) at enrollment;	Bone structure and strength; Measurements taken at distal radius and mid-shaft tibia	VD3: 1,400 IU/week	6	Placebo	Distal radius: +<br>Tibia: 0

Author (year)	Study design; N enrolled	Enrollment years	Location; latitude	GA or mean age (SD) / median [range]	Male (%)	Breastfeeding status	Race or ethnicity	Health status; nutritional status	BMD/BMC outcome analyzed	Vitamin D or calcium intervention group(s)	Intervention duration (months)	Comparison group(s) [a]	Key findings[b]
					follow-up			~50% were VD deficient at follow-up					

BF = breastfeeding; BMC = bone mineral content; BMD = bone mineral density; BTT = Bone Transmission Time; Ca = Calcium; CALCBD = calculated bone density; CI = confidence interval; CORTBD = cortical bone density; CSA = cross-sectional area of the bone; GA = gestational age; IQR = interquartile range; IU/d = international units per day; N = sample size; NA = not applicable; NR = not reported; RCT = randomized controlled trial; SD = standard deviation; SOS = Speed of Sound; SSI = stress and strain index; Supp = supplement; VD = vitamin D; VD2 = vitamin D_2; VD3 = vitamin D_3

[a] Comparison group: Non-vitamin D or non-calcium intervention group.

[b] Results compare higher VD dose groups to lower dose groups unless otherwise noted: ++ Significant beneficial effects of higher VD dose ($P < 0.05$); + Marginally significant beneficial effects ($0.05 < P < 0.1$); 0 No effects; - Marginally significant detrimental effects ($0.05 < P < 0.1$); -- Significant detrimental effects ($P < 0.05$).

[c] Follow-up to the Gallo et al. (2013) study conducted 3 years after enrollment.

[d] At age 6 months, infants randomized to the placebo group were also given 400 IU VD/d after which all randomized infants received supplementation until weaned from breastfeeding or until age 12 months when the study ended.

[e] According to Hibbs et al. (2018) [24], "No participants had a tibial speed of sound measurement more than 2 SDs below the mean, based on previously published norms, at 12 months" (p. 2092).

[f] Results from multivariate analysis of covariance with gender and quality of pQCT measurement as covariates.

Figure KQ1-5. Summary ROB plot (panel a) and individual study ROB (panel b) for vitamin D interventional studies reporting bone mineral density or bone mineral content outcomes

a.

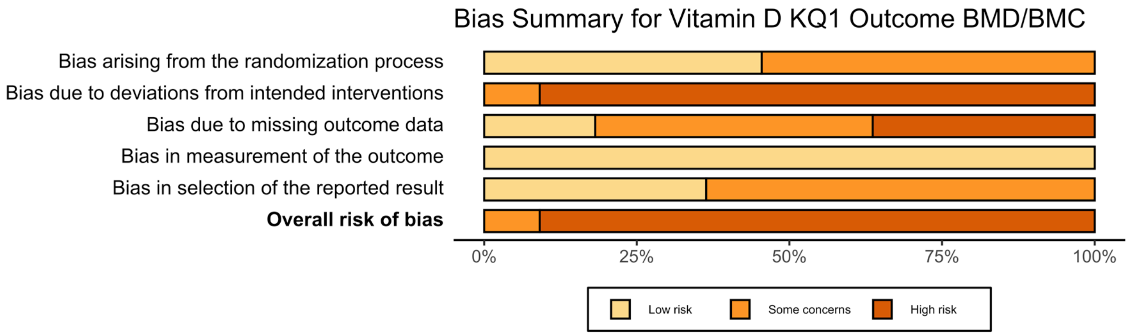

b.

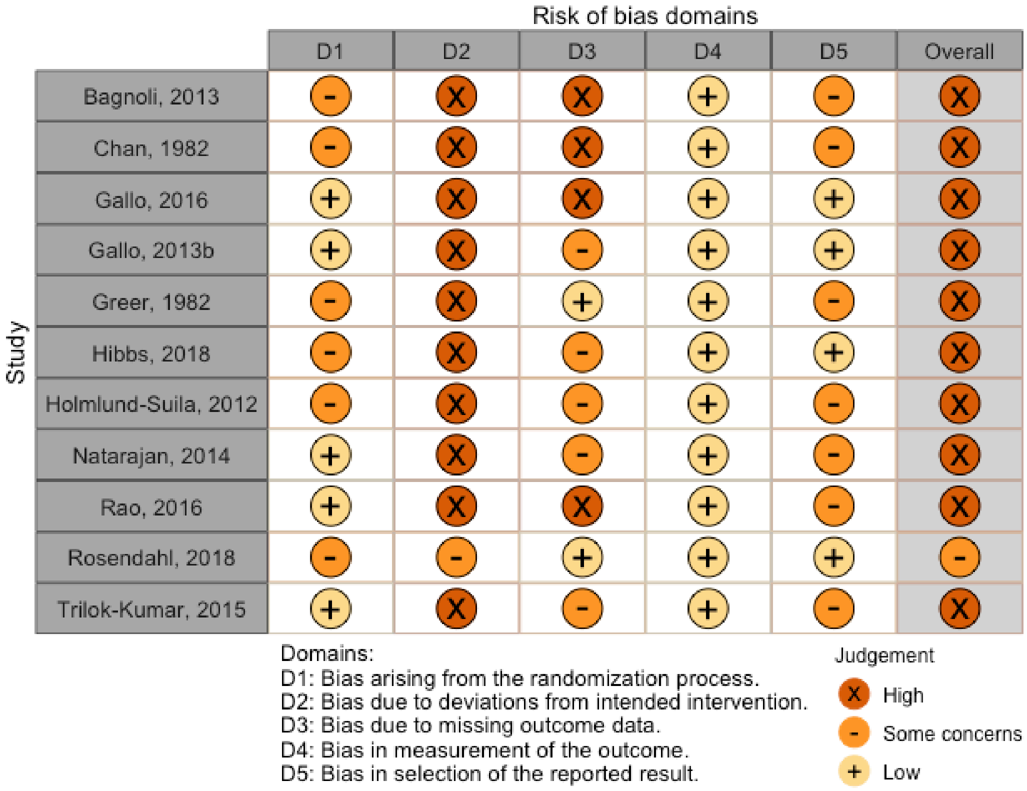

KQ1. Blood Pressure Outcomes

In a follow-up study (n = 912; 47.9% male) of a large RCT (n = 2,079; 46.7% male) conducted in newborns (mean age = 2 days) in New Delhi, India (29° N), blood pressure outcomes were measured when participants were age 3-6 years (mean [SD] = 5.0 [1.0] years).[48] The characteristics of this follow-up study are presented in **Table KQ1-6**. In the original study, newborn participants were assigned to either 1,400 IU vitamin D per week or a placebo for a duration of six months. After 3 to 6 years of follow-up, mean differences between the vitamin D and placebo arms for systolic (95% CI -0.63, 1.69; *P* = 0.37) and diastolic (95% CI -0.96, 0.87; *P* = 0.92) blood pressure were not statistically significant. Risk of bias for this study was assessed as "low" for the randomization and measurement of the outcome domains. There was some risk of bias due to a high proportion of missing data, and there was some risk of bias for selection of the reported result due to the lack of a pre-specified analysis plan. A lack of adherence to intended interventions in more than 20% of participants with no report of analyses addressing this issue led to high risk of bias in this final domain.

Table KQ1-6. Characteristics and key findings of one interventional study reporting on blood pressure outcomes

Author (year)	Study design; N enrolled	Enrollment years	Location; latitude	Mean age (SD) [range]	Male (%)	Breastfeeding Status	Race or ethnicity	Health status; nutritional status	Study groups	Study duration	Key findings[a]
Trilok-Kumar et al. (2015) [48]	RCT; N=2,079 (N=912 at follow-up)	2007-2010	New Delhi, India; 29°	~2.0 (0) [2] days at enrollment; 5.0 (1.0) [3-6] years at follow-up	46.7 at enrollment; 47.9 at follow-up	NR; NA at follow-up	Presumed 100% Asian Indian	Low birth weight (1.8 to <2.5 kg) at enrollment; ~50% were VD deficient at follow-up	VD3: 1400 IU/week Placebo	Intervention: 6 months; Follow-up: 3-6 years	Systolic BP: 0 Diastolic BP: 0

BP = blood pressure; IU = international units; N = sample size; NA = not applicable; NR = not reported; RCT = randomized controlled trial; VD3 = vitamin D_3

[a] Results compare vitamin D (VD) intervention group to placebo group: ++ Significant beneficial effects of VD ($P < 0.05$); + Marginally significant beneficial effects ($0.05 < P < 0.1$); 0 No effects; - Marginally significant detrimental effects ($0.05 < P < 0.1$); -- Significant detrimental effects ($P < 0.05$).

KQ1. UV Light Studies

In this section, we describe intervention studies reporting the effect of UV or sunlight on serum 25(OH)D and clinical outcomes. Two RCTs were identified that reported on the effect of UV or sunlight on motor development and serum 25(OH)D.[58,59] In a nonrandomized trial conducted in Xi'an, China (latitude, 35.4° N), 229 generally healthy neonates born from depressed mothers were divided into groups receiving either 400 IU/d vitamin D_3, 1,000 IU/d vitamin D_3, 400 IU/d vitamin D_3 supplementation plus sunlight exposure of various durations, or no intervention (control group). Infants who received 400 IU/d vitamin D_3 plus sunlight exposure achieved significantly higher scores in motor development compared to other groups (P<0.05). Within the sunlight exposure group (7-14 hours per week, <7 hours per week, and <2 hours per week), a dose-response relationship was evident, with infants receiving the most amount of sunlight per week achieving the highest motor development scores (P<0.05 for all comparisons). In a randomized trial conducted in Beijing, China (latitude, 39° N), 54 healthy infants were randomized to receive two hours of sunlight each day or the usual amount of sunlight (control). After two months, mean serum 25(OH)D had increased significantly in infants in the 2-hour sunlight group, but did not significantly change in the control group (30 +/- 37.5 ng/mL vs. -7.5 +/- 20 ng/mL). Further, a dose-response relationship was evident, with infant serum 25(OH)D concentration at two months correlating with UV exposure scores (r=0.61, P<0.001).

Figure KQ1-6 shows the individual study ROB for the two trials in this section. Both studies had high ROB due to deviations from intended intervention and some ROB in selection of the reported results. One study also had high ROB due to a nonrandomized design and missing outcome data. Both trials had low ROB in measurement of the outcome.

Figure KQ1-6. Individual study ROB for studies reporting the effect of UV or sunlight on serum 25(OH)D or clinical outcomes of interest

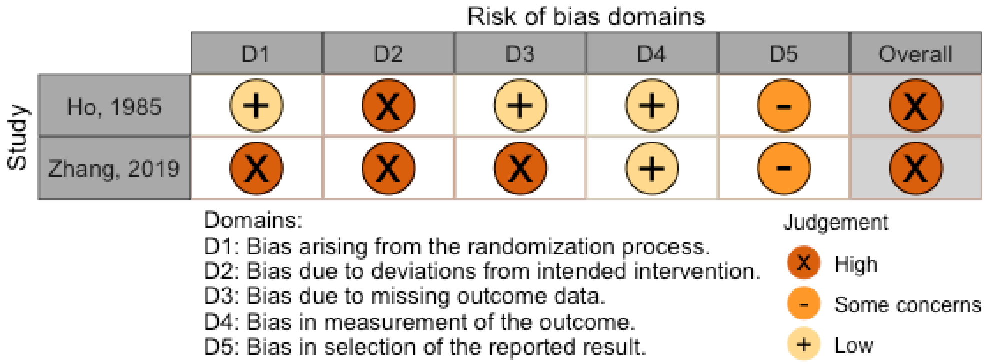

KQ2: What is the association between serum 25(OH)D concentrations and health outcomes in children aged 0 to 4 years?

A total of 18 observational studies examining the association between serum 25(OH)D concentrations and health outcomes in children aged 0 to 4 years were included. Findings below are organized by outcomes.

KQ2. Atopic Outcomes: Asthma, Wheezing, and Eczema

Table KQ2-1 shows the study characteristics and results of four observational studies reporting the association between serum 25(OH)D and asthma, wheezing, and eczema outcomes included in our review. The studies include three cohort studies and one case-cohort study, with the number of enrolled participants ranging from 263 to 5,044 for each study. Two of the articles were conducted in neonates in Australia, one in infants in Canada, and one in infants the United States. All studies either reported background diets as any (mixed) breastfeeding, or breastfeeding status was not reported. Race or ethnicity was not reported in two studies, one study enrolled primarily white participants,[60] and the last study enrolled primarily Hispanic and Black participants.[61]

Table KQ2-2 shows the ROB assessment of cohort studies in this section. Overall, there was concern for ROB due to absent or unclear demonstration that the outcome was not present at the start of the study, poor adjustment of possible confounders, poor or unclear assessment of the outcome, and significant loss to follow up of participants. **Table KQ2-3** shows the ROB assessment of the one case-cohort study reporting on asthma.[60] There was concern for ROB due to selection of cases, as 100% of cases were not included in the study. There was also concern for risk of bias since reporting lacked description of how the outcome was assessed. Lastly, there was concern for ROB due to the analytic methods.

Asthma

Table KQ2-1 shows two cohort studies that investigated the association between serum 25(OH)D at baseline and asthma as an outcome. Neither study found a significant association.[61,62] A third cohort study reported significantly increasing odds of asthma at age 10 years for each additional follow-up visit from age six months to 10 years where participants were vitamin D deficient (<50 nmol/L [<20 ng/mL]) (adjusted OR not reported; *P*<.05).[63]

Wheeze

Table KQ2-1 shows two cohort studies that investigated the association between serum 25(OH)D and wheeze outcome. One study found no association between serum 25(OH)D and wheezing.[62] The other study reported significantly increasing odds of wheezing at age 10 years for every vitamin D deficient (<50 nmol/L [<20 ng/mL]) follow-up visit when participants were six months to 10 years (adjusted OR not reported; *P*<.01).[63]

Eczema

Table KQ2-1 shows one case-cohort study that found no association between serum 25(OH)D and eczema outcome.[60] The other study was a cohort study which reported significantly increasing odds of eczema at age 10 years for each additional vitamin D deficient (<50 nmol/L [<20 ng/mL]) follow-up visit between age six months and 10 years (adjusted OR not reported; *P*<.05).[63]

Table KQ2-1. Characteristics and key findings of studies of observational studies reporting the association of serum 25(OH)D and asthma, wheezing, and eczema outcomes

**Authors (year)**	**Study design; N analyzed**	**Enrollment years**	**Location; latitude**	**Mean age (SD) [range]**	**Male (%)**	**Breastfeeding status**	**Race or ethnicity**	**Health status; nutritional status**	**Mean follow-up (SD)**	**Exposure or comparisons**	**Key findings [a]**
Anderson et al. (2015) [62]	Cohort; N= 5044	2008-2012	Canada; 44°	2.54 (1.5) [0-5]	51.1	Any BF	NR	100% Healthy; NR	2.3 (1.2) years	Serum 25(OH)D at baseline	Asthma: 0 [b]<br><br>Wheeze: 0 [c]
Hollams et al. (2017) [63]	Cohort; N= 263	1996-1998	Western Australia; NR	Neonates	NR	Any BF	NR	Generally healthy; NR	10 years	Number of deficient [25(OH)D <50 nmol/L] follow ups by 10 years of age	Asthma: ++ [d]<br><br>Medicated asthma 0 [d]<br><br>Wheeze: ++ [d]<br><br>Eczema: ++ [d]
Molloy et al. (2017) [60]	Case-cohort; N= 1074	NR	Southeast Australia; -30°	Neonates	52	Any BF	72% White, 28% Non-White	Generally healthy; NA	6 months (12 months of age)	Serum 25(OH)D levels at 6 months of age (nmol/L): <50 vs. >= 50	Eczema: 0 [e]
Navas-Nazario et al. (2011) [61]	Cohort; N= 601	2005-2008	New Haven, CT; 41.3°	1.8 (0.7)	46.6	NR	72.1% Hispanic, 21.2% Black, 6.7% White	NR	5.9 (1.0) years of age	Serum 25(OH)D levels at baseline	Asthma: 0 [f]

BF = breastfeeding; N = sample size; NA = not applicable; NR = not reported; SD = standard deviation

[a] Key findings: ++ Significant difference indicating benefit of higher serum 25(OH)D levels ($p < 0.05$); + Marginally significant difference indicating benefit ($0.05 < p < 0.1$); 0 No significant difference; - Marginally significant difference indicating detriment ($0.05 < p < 0.1$); -- Significant difference indicating detriment ($p < 0.05$).
[b] Adjusted for child sex, neighborhood income, smoker in household, maternal ethnicity, child in licensed daycare, child's age in months, z-BMI, birth weight, hours of outdoor free play, breastfeeding duration and family history of asthma.
[c] Adjusted for child sex, family income, smoker in household, maternal ethnicity, child in licensed daycare, age in months, z-BMI, birth weight, hours of outdoor free play, breastfeeding duration and family history of asthma (both mother and father).
[d] Adjusted for sex, month of birth, cesarian birth, birth weight, breast-feeding for less than 3 months, antenatal and/or childhood smoke exposure, childcare attendance, and living with older children by age 5 years.
[e] Adjusted for family history of allergy, ethnicity, number of siblings, domestic pets, and formula feeding at 6 months.
[f] Unadjusted.

Table KQ2-2. Risk of bias assessment results for cohort studies reporting the association between serum 25(OH)D and asthma, wheezing, and eczema outcomes[a]

Author (year)	Outcome assessed	Selection of the non-exposed cohort	Ascertainment of 25(OH)D concentrations	Demonstration that outcome of interest was not present at start of study	Comparability of cohorts on the basis of the design or analysis	Assessment of outcome	Was follow-up long enough for outcomes to occur?	Adequacy of follow up of cohorts
Anderson et al. (2015) [62]	Asthma	a) Drawn from the same community as the exposed cohort	b) Method used was HPLC, RIA kits, LC-MS/MS OR EIA/Chemiluminescence	b) Present at start or unknown	c) Both a) and b) [b]	c) Self-report only with no reference to original health records or no documented source	a) Yes	c) Lost to follow up rate ⩾ 20% and no description of those lost
Anderson et al. (2015) [62]	Wheeze	a) Drawn from the same community as the exposed cohort	b) Method used was HPLC, RIA kits, LC-MS/MS OR EIA/Chemiluminescence	b) Present at start or unknown	c) Both a) and b) [b]	c) Self-report only with no reference to original health records or no documented source	a) Yes	c) Lost to follow up rate ⩾ 20% and no description of those lost
Hollams et al. (2017) [63]	Asthma	a) Drawn from the same community as the exposed cohort	b) Method used was HPLC, RIA kits, LC-MS/MS OR EIA/Chemiluminescence	b) Present at start or unknown	a) Study controls for at least 4/6 of the important factors or gives justification for non-inclusion	c) Self-report only with no reference to original health records or no documented source	a) Yes	c) Lost to follow up rate ⩾ 20% and no description of those lost
Hollams et al. (2017) [63]	Medicated asthma	a) Drawn from the same community as the exposed cohort	b) Method used was HPLC, RIA kits, LC-MS/MS OR EIA/Chemiluminescence	b) Not present at start	a) Study controls for at least 4/6 of the important factors or gives justification for non-inclusion	c) Self-report only with no reference to original health records or no documented source	a) Yes	c) Lost to follow up rate ⩾ 20% and no description of those lost
Hollams et al. (2017) [63]	Wheeze	a) Drawn from the same community as the exposed cohort	b) Method used was HPLC, RIA kits, LC-MS/MS OR EIA/Chemiluminescence	b) Present at start or unknown	a) Study controls for at least 4/6 of the important factors or gives justification for non-inclusion	c) Self-report only with no reference to original health records or no documented source	a) Yes	c) Lost to follow up rate ⩾ 20% and no description of those lost
Hollams et al. (2017) [63]	Eczema	a) Drawn from the same community as	b) Method used was HPLC, RIA kits, LC-MS/MS	b) Present at start or unknown	a) Study controls for at least 4/6 of the important factors or gives	c) Self-report only with no reference to original	a) Yes	c) Lost to follow up rate ⩾ 20%

Author (year)	Outcome assessed	Selection of the non-exposed cohort	Ascertainment of 25(OH)D concentrations	Demonstration that outcome of interest was not present at start of study	Comparability of cohorts on the basis of the design or analysis	Assessment of outcome	Was follow-up long enough for outcomes to occur?	Adequacy of follow up of cohorts
		the exposed cohort	OR EIA/Chemiluminescence		justification for non-inclusion	health records or no documented source		and no description of those lost
Navas-Nazario et al. (2011) [61]	Asthma	a) Drawn from the same community as the exposed cohort	b) Method used was HPLC, RIA kits, LC-MS/MS OR EIA/Chemiluminescence	b) Not present at start	d) Neither a) nor b) *	e) Both a) and b)	a) Yes	a) Complete follow up - all subjects were accounted for

EIA = Enzyme immunoassay; HPLC = High performance liquid chromatography; LC-MS = Liquid chromatography mass spectrometry; RIA = Radioimmunoassay

[a] Assessed with *The Newcastle-Ottawa Scale (NOS) for assessing the quality of nonrandomized studies in meta-analyses.*
[b] Study controls for 3+ important factors AND any of the other factors, gives justification for non-inclusion.

Table KQ2-3. Risk of bias assessment results for a case-cohort study reporting the association between serum 25(OH)D and asthma, wheezing, and eczema outcomes[a,b]

Author (year)	Study design; outcome assessed	Follow-up in the original cohort	Selection of cases	Selection of controls	Ascertainment of 25(OH)D concentrations	Demonstration that outcome of interest was not present at start of study	Comparability of cohorts on the basis of the design or analysis	Assessment of outcome	Was follow-up long enough for outcomes to occur?	Adequacy of follow up of cohorts	Weighting	Adjusted variance
Molloy et al. (2017) [60]	Case-cohort; Eczema	b) Subjects lost to follow up unlikely to introduce bias	b) Not all cases from the cohort were selected or no description	a) Drawn randomly or matched with cases AND drawn from the same cohort as cases	b) Method used was HPLC, RIA kits, LC-MS/MS OR EIA/Chemiluminescence	a) Not present at start	c) Both a) and b)	d) No description	a) Yes	b) Controls lost to follow up unlikely to introduce bias	c) Analysis used calibration or estimation to adjust sampling weights	b) Analysis does not include adjusted variance, or no statement

EIA = Enzyme immunoassay; HPLC = High performance liquid chromatography; LC-MS = Liquid chromatography mass spectrometry; RIA = Radioimmunoassay

[a] Assessed with an adapted version of *The Newcastle-Ottawa Scale (NOS) for assessing the quality of nonrandomised studies in meta-analyses.*

[b] No nested case-control studies included were included in this section.

KQ2. Infectious Disease Outcomes

Table KQ2-4 shows study characteristics and results of four observational studies from three publications reporting the association between serum 25(OH)D and infectious disease outcomes. Two studies were conducted in Bangladesh, one in Western Australia, and one in Tanzania. All four were cohort studies that compared rates of infectious disease outcomes by serum 25(OH)D sufficiency status or quartile.[63-65] One paper reported results stratified by weight status (normal vs. underweight), and these are presented on separate rows in Table KQ2-4.[64] Sample sizes of the included studies ranged from 263 to 948. In all studies, participants were 24 months or younger at enrollment. Follow-up duration was precisely reported in two publications[63,64] while the other reported age of last follow-up.[65]

Reported outcomes included upper respiratory tract infection (URTI), acute lower respiratory tract infection (ALRI), respiratory tract infection, malaria infection, and oral candidiasis. Most reported associations between serum 25(OH)D and infectious disease outcomes were not statistically significant. Mixed results with significant associations were found for three of eight total infectious disease outcomes as follows. One study found that serum 25(OH)D concentrations <10 ng/mL were associated with an increased incidence of oral candidiasis in HIV-exposed infants (adjusted IRR = 1.47; 95% CI 1.00, 2.15; *P*=0.046).[65] However, the same study reported that serum 25(OH)D concentrations ≥30ng/mL were significantly associated with an increased incidence of clinical (adjusted IRR = 1.34; 95% CI 1.06, 1.70; *P*=0.02) and confirmed (adjusted IRR = 1.71; 95% CI 1.15, 2.54; *P*<0.01) malaria diagnoses. Another study reported lower risk of URTI in underweight children who were vitamin D-deficient (adjusted IRR = 0.73; 95% CI 0.61, 0.89; *P*<0.001) or vitamin D-insufficient (adjusted IRR = 0.80; 95% CI 0.68, 0.94; *P*<0.05) at baseline compared to those who were vitamin D-sufficient.[64,65]

Table KQ2-5 shows the ROB assessment of cohort studies reporting infectious disease outcomes. In one study, there was ROB due to lack of reporting that the outcome was not present at the start of the study, possible insufficient adjustment for potentially relevant confounders, and a significant number of participants lost to follow-up.[63] There was concern for ROB in a second study due to unclear adequacy of follow-up of participants.[65]

Table KQ2-4. Characteristics and results of observational studies reporting the association between vitamin D intake and infectious disease outcomes

**Author (year)**	**Study design; N analyzed**	**Enrollment years**	**Location; latitude**	**Age range [h]**	**Male (%)**	**Breastfeeding status**	**Race or ethnicity**	**Health status [h]; nutritional status [h]**	**Mean follow-up (SD)**	**Exposure**	**Key findings [b]**
Ahmed et al. (2016) [64]	Cohort; N= 446	2010-2012	Dhaka, Bangladesh; 23.8°	6-24 months	51.3	NR	100% Asian Indian	NR; NR	0.38 years (NR)	Serum 25(OH)D levels at 6-24 months at birth, 6 months, and years 1, 2, 3, and 4: sufficiency, insufficiency, and deficiency [i]	URTI: 0 (deficiency and insufficiency vs. sufficiency (ref.)) [j]<br><br>ALRI: 0 (deficiency and insufficiency vs. sufficiency (ref.)) [j]
Ahmed et al. (2016) [64]	Cohort; N= 466	2010-2012	Dhaka, Bangladesh; 23.8°	6-24 months	50.2	NR	100% Asian Indian	NR; 100% underweight (weight-for-age z-score < -2.00 SD)	0.37 years (NR)	Serum 25(OH)D levels at birth, 6 months, and years 1, 2, 3, and 4: sufficiency, insufficiency, and deficiency [i]	URTI: -- (deficiency and insufficiency vs. sufficiency (ref.)) [j]<br><br>ALRI: 0 (deficiency and insufficiency vs. sufficiency (ref.)) [j]
Hollams et al. (2017) [63]	Cohort; N= 263	1996-1998	Western, Australia; NR	Neonates	NR	Any BF	NR	Generally healthy; NR	Proceeding 6 or 12 months after exposure assessment	Serum 25(OH)D levels at birth, 6 months, and years 1, 2, 3, and 4: sufficiency, insufficiency, and deficiency [i]	Respiratory tract infection: 0 (sufficiency vs insufficiency vs deficiency at all follow ups) [k]
Sudfeld et al. (2015) [65] (HIV-exposed, uninfected cohort)	Cohort; N= 948	NR	Tanzania; ~-6°	5-7 weeks	53.5	Any BF	100% Tanzanian	Generally healthy; low VD status (mean serum 25(OH)D 18.1 ng/mL and SD 9.2 ng/mL)	20.9 months [a] (IQR: 17.0–23.9)	Serum 25(OH)D levels at 5-7 weeks of age (ng/mL): <10, 10-19.9, 20-29.9 (ref.), >=30	ALRI: 0 (serum 25(OH)D quartiles vs. second highest quartile (ref.)) [g];<br><br>Malaria infection: -- (highest quartile vs second highest quartile of serum 25(OH)D (ref.)) [g];<br><br>Oral candidiasis: ++ (lowest quartile of serum 25(OH)D vs. second highest quartile (ref.)) [g]

ALRI = acute lower respiratory tract infection; BF = breast-feeding; HIV = human immunodeficiency virus; IQR = interquartile range; n = sample size; NR = not reported; ref. = reference group; SD = standard deviation; URTI = upper respiratory tract infection; VD = vitamin D

[a] Median age at follow up.
[b] Key findings: ++ Significant difference indicating benefit of higher serum 25(OH)D levels ($p < 0.05$); + Marginally significant difference indicating benefit ($0.05 < p < 0.1$); 0 No significant difference; - Marginally significant difference indicating detriment ($0.05 < p < 0.1$); -- Significant difference indicating detriment ($p < 0.05$).
[g] Adjusted for baseline maternal factors, including age, education, marital status, number of prior pregnancies, household assets, food expenditure per person, underweight, anemia, CD4 T-cell count, and use of antiretrovirals during pregnancy, and baseline child factors, including sex, exclusive breastfeeding, stunting, wasting, low birth weight, anemia, season of 25(OH)D measurement, and randomized treatment regimen.
[h] At baseline or first exposure assessment; no studies reported the mean or median age.
[i] Defined as <50 nmol/L, >=50 and <75 nmol/l, >=75 nmol/l, respectively.
[j] Adjusted for serum retinol, serum zinc, maternal education, household wealth index, and season of vitamin D measurement.
[k] Unadjusted.

Table KQ2-5. Risk of bias assessment results for cohort studies reporting the association between vitamin D intake and infectious disease outcomes[a]

Author (year)	Outcome(s) assessed	Selection of the non-exposed cohort	Ascertainment of 25(OH)D concentrations	Demonstration that outcome of interest was not present at start of study	Comparability of cohorts on the basis of the design or analysis	Assessment of outcome	Was follow-up long enough for outcomes to occur?	Adequacy of follow up of cohorts
Ahmed et al. (2016) [64]	URTI; ALRI	a) Drawn from the same community as the exposed cohort	b) Method used was HPLC, RIA kits, LC-MS/MS OR EIA/Chemilumin escence	a) Not present at start	c) Both a) and b) [b]	a) Independent blind assessment	a) Yes (for both URTI and ALRI outcomes)	a) Complete follow up - all subjects were accounted for
Hollams et al. (2017) [63]	Respiratory tract infection	a) Drawn from the same community as the exposed cohort	b) Method used was HPLC, RIA kits, LC-MS/MS OR EIA/Chemilumin escence	b) Present at start or unknown	a) Study controls for at least 4/6 of the important factors or gives justification for non-inclusion	c) Self-report only with no reference to original health records or no documented source	a) Yes	c) Lost to follow up rate ≥ 20% and no description of those lost
Sudfeld et al. (2015) [65]	ALRI; malaria infection; oral candidiasis	a) Drawn from the same community as the exposed cohort	b) Method used was HPLC, RIA kits, LC-MS/MS OR EIA/Chemilumin escence	a) Not present at start	c) Both a) and b) [b]	a) Independent blind assessment	a) Yes	d) No statement

ALRI = acute lower respiratory tract infection; EIA = Enzyme immunoassay; HPLC = High performance liquid chromatography; LC-MS = Liquid chromatography mass spectrometry; RIA = Radioimmunoassay; URTI = upper respiratory tract infection

[a] Assessed with *The Newcastle-Ottawa Scale (NOS) for assessing the quality of non-randomised studies in meta-analyses.*
[b] Study controls for 3+ important factors AND any of the other factors, gives justification for non-inclusion.

KQ2. Autoimmune Disease Outcomes

Table KQ2-6 shows study characteristics and results of seven observational studies from six articles reporting the association between serum vitamin D and autoimmune disease outcomes. Reported outcomes included type 1 diabetes, islet autoimmunity (a precursor to type 1 diabetes), and juvenile idiopathic arthritis (JIA). Four references reported on nested case-control studies,[66-69] one reported a case-cohort study design,[67] and two reported case-cohort studies.[70,71] Sample sizes in the included studies ranged from 128 to 8,676, and the mean age of participants at baseline or first measure of serum vitamin D was under 12 months for all studies. Follow-up duration was precisely reported in two studies,[66,70] and the other studies either did not report duration or reported only the age or year of last follow-up.[67-69,71]

Four articles reported no association between serum vitamin D level (including serum 25(OH)D_2, 25(OH)D_3, or serum 25(OH)D) and either type 1 diabetes or islet autoimmunity outcomes,[66-68,70] and a fifth article reported no association between 25(OH)D levels and odds of JIA.[71] One nested case-control study of 8,676 children at increased genetic risk of type 1 diabetes found that higher serum 25(OH)D, as measured in infancy and in early childhood, was associated with lower odds of islet autoimmunity (adjusted OR = 0.93 per 5 nmol/L difference; 95% CI 0.89, 0.97).[69]

Table KQ2-7 shows the ROB assessment of case-cohort and nested case-control studies reporting autoimmune disease outcomes. At least one study had ROB due to each of the following reasons: unknown follow-up of the original cohort, incomplete or poor description of case selection, non-random or unmatched selection of controls, non-optimal adjustment of possible confounders, poor description of how outcomes were assessed, incomplete or poor description of subject follow-up rate, or inappropriate or poorly described analytic methods.

Table KQ2-6. Characteristics and results of observational studies reporting the association between vitamin D intake and autoimmune disease outcomes

Author (year)	Study design; N enrolled	Enrollment years	Location [latitude]	Mean age (SD) [range] [h]	Male (%)	Breastfeeding status	Race or ethnicity	Health status [h]; nutritional status [h]	Mean follow-up (SD)	Exposure	Key findings [b]
Cadario et al. (2015) [66]	Nested case-control; N= 303	Since 1990	Italy	Neonates	48	NR	NR	Generally healthy; NR	Cases: 7.0 (0.25); Controls 7.2 (0.49)	Serum 25(OH)D at birth	Type 1 diabetics vs. nondiabetics: 0 [o]
Jacobsen et al. 2016 [67]	Case-cohort; N= 3778	1981-2002	Denmark; ~56°	Neonates	51.2	NR	Mothers >90% Danish	Generally healthy; NR	Until end of 2012; Cases: 10.2; Controls: 10.5 [n]	Serum 25(OH)D, 25(OH)D_2, and 25(OH)D_3 at birth	Type 1 diabetics vs. nondiabetics: 0 (for all three exposures) [g]
Jacobsen et al. 2016 [67]	Nested case-control; N=1054	1981-2002	Denmark; ~56°	Neonates	52.3	NR	Mothers >95% Danish	Generally healthy; NR	Until May 2012	Serum 25(OH)D, 25(OH)D_2, and 25(OH)D_3 at birth	Type 1 diabetics vs. nondiabetics: 0 (for all three exposures) [m]
Makinen et al. (2016) [68]	Nested case-control; N= 252	1994-2004	Tampere, Finland; 61°	3 (0) months	51	NR	NR	Generally healthy, 100% with HLA-conferred susceptibility to T1D; NR	Cases: 2.53 y of age; Controls: 2.92 y of age [a]	Serum 25(OH)D in infancy	Type 1 diabetics vs. nondiabetics: 0 [l]
Norris et al. (2018) [69]	Nested case-control; N= 8676	2004-2010	USA and Europe	95% age 3-12 months at first sample	55.6	NR	NR	Generally healthy, 100% with high-risk HLA haplotype or first-degree relative with T1D; NR	NR	Serum 25(OH)D in first year of life and in childhood	Islet autoimmunity vs. non-cases: ++ (for first year of life exposure) [d]<br><br>Islet autoimmunity vs. non-cases: ++ (for childhood exposure) [e]
Simpson et al. (2011) (study 1c) [70]	Case-cohort; N= 128	1993-2006	Denver, USA; 39.7°	NR [0.1-0.75] years	50	NR	76% Non-Hispanic white	Generally healthy, 100% with high-risk allele in HLA region or first-degree relative with T1D; NR	Cases: 3.5 (2.6) y; Controls 7.3 (4.0) y	Serum 25(OH)D at 9 months of age; VD insufficiency at 9 months of age	Islet autoimmunity vs. non-cases: 0 [f] (for both exposures)

Thorsen et al. (2017) [71]	Case-cohort; N= 600	1983-2012	Denmark	Neonates	40.2	NR	89.3% ethnic Dane	Generally healthy; NR	Median age 5-8.5 years	Serum 25(OH)D at birth	Oligoarticular JIA groups vs. controls: 0 [j]<br><br>Polyarticular JIA groups vs. controls: 0 [j]

HLA = human leukocyte antigen; JIA = juvenile idiopathic arthritis; NR = not reported; ref. = reference group; T1D = type 1 diabetes; VD = vitamin D; y = years

[a] Median age at follow up.

[b] Results: ++ Significant difference indicating benefit of higher serum 25(OH)D levels ($p < 0.05$); + Marginally significant difference indicating benefit ($0.05 < p < 0.1$); 0 No significant difference; - Marginally significant difference indicating detriment ($0.05 < p < 0.1$); -- Significant difference indicating detriment ($p < 0.05$).

[c] Multiple serum 25(OH)D measures were collected in each subject.

[d] Controls matched for clinical center, sex, and family history of type 1 diabetes; adjusted for HLA-DR3/4 status and the first two PCs indicating ancestry, age, and season of sample collection at the first visit.

[e] Adjusted for HLA-DR3/4 status and the first two PCs indicating ancestry.

[f] Adjusted for family history of T1D and HLA-DR3/4, DQB1*0302 genotype.

[g] Adjusted for maternal and paternal type 1 diabetes status.

[h] At baseline or first exposure assessment.

[j] Controls matched for date of birth; adjusted for 25(OH)D, gender, ethnicity, weight (categorical), gestational age (categorical), and mother’s age (categorical).

[k] Controls matched for date of birth and ethnic group.

[l] Controls matched for age, sex, study site, and HLA-conferred; adjusted for sample month, sample year, age of child at sample draw, and sex.

[m] Controls matched for season of birth; adjusted for HLA genotype.

[n] Median age at last follow-up.

[o] Controls matched for birth date and ethnicity, model unadjusted.

Table KQ2-7. Risk of bias assessment results for case-cohort and nested case-control studies reporting the association between serum 25(OH)D and autoimmune disease outcomes[a]

Author (year)	Study design; outcome assessed	Follow-up in the original cohort	Selection of cases	Selection of controls	Ascertainment of 25(OH)D concentrations	Demonstration that outcome of interest was not present at start of study	Comparability of cohorts on the basis of the design or analysis	Assessment of outcome	Was follow-up long enough for outcomes to occur?	Adequacy of follow up of cohorts	C-C: Weighting; NCC: Accounts for unequal probability sampling methods	Adjusted variance
Cadario et al. (2015) [66]	NCC; Type 1 diabetes	d) No statement	a) All cases from the cohort were selected	a) Drawn randomly or matched with cases AND drawn from the same cohort as cases	b) Method used was HPLC, RIA kits, LC-MS/MS OR EIA/Chemiluminescence	a) Not present at start	a) Study controls for at least 4/6 of the important factors, OR the authors described the variable selection process or gives justification for non-inclusion	e) Both a) and b)	a) Yes	d) No statement	b) Conditional logistic regression analysis with matching variables or stratification	b) Analysis does not include adjusted variance, or no statement
Jacobsen et al. (2016) – Study 1 [67]	C-C; Type 1 diabetes	b) Subjects lost to follow up unlikely to introduce bias	b) Not all cases from the cohort were selected or no description	a) Drawn randomly or matched with cases AND drawn from the same cohort as cases	b) Method used was HPLC, RIA kits, LC-MS/MS OR EIA/Chemiluminescence	a) Not present at start	c) Both a) and b)	e) Both a) and b)	a) Yes	a) Complete follow up - all controls were accounted for	b) Analysis involves weighted likelihood approach	a) Analysis includes robust estimation of the variance
Jacobsen et al. (2016) – Study 2 [67]	NCC; Type 1 diabetes	b) Subjects lost to follow up unlikely to introduce bias	b) Not all cases from the cohort were selected	a) Drawn randomly or matched with cases AND drawn	b) Method used was HPLC, RIA kits, LC-MS/MS	a) Not present at start	c) Both a) and b)	e) Both a) and b)	a) Yes	a) Complete follow up - all controls were	b) Conditional logistic regression analysis with matching	b) Analysis does not include adjusted variance,

Author (year)	Study design; outcome assessed	Follow-up in the original cohort	Selection of cases	Selection of controls	Ascertainment of 25(OH)D concentrations	Demonstration that outcome of interest was not present at start of study	Comparability of cohorts on the basis of the design or analysis	Assessment of outcome	Was follow-up long enough for outcomes to occur?	Adequacy of follow up of cohorts	C-C: Weighting; NCC: Accounts for unequal probability sampling methods	Adjusted variance
			or no description	from the same cohort as cases	OR EIA/Chemiluminescence					accounted for	variables or stratification	or no statement
Makinen et al. (2016) [68]	NCC; Type 1 diabetes	c) Lost to follow up rate ≥ 20% and no description of those lost	b) Not all cases from the cohort were selected or no description	a) Drawn randomly or matched with cases AND drawn from the same cohort as cases	b) Method used was HPLC, RIA kits, LC-MS/MS OR EIA/Chemiluminescence	a) Not present at start	a) Study controls for at least 4/6 of the important factors or gives justification for non-inclusion	a) Independent blind assessment (e.g., by physician/ nurse or from health records)	a) Yes	b) Controls lost to follow up unlikely to introduce bias	b) Conditional logistic regression analysis with matching variables or stratification	b) Analysis does not include adjusted variance, or no statement
Norris et al. (2018) [69]	NCC; Islet autoimmunity	d) No statement	a) All cases from the cohort were selected	a) Drawn randomly or matched with cases AND drawn from the same cohort as cases	d) Both a) and b)	a) Not present at start	c) Both a) and b)	a) Independent blind assessment (e.g., by physician/ nurse or from health records)	a) Yes	b) Controls lost to follow up unlikely to introduce bias	b) Conditional logistic regression analysis with matching variables or stratification	b) Analysis does not include adjusted variance, or no statement
Simpson et al. (2011) – Study 1c [70]	C-C; Islet autoimmunity	c) Lost to follow up rate ≥ 20% and no description of those lost	b) Not all cases from the cohort were selected or no	b) Neither drawn randomly nor matched OR drawn from a different	b) Method used was HPLC, RIA kits, LC-MS/MS OR EIA/Chem	a) Not present at start	c) Both a) and b)	a) Independent blind assessment (e.g., by physician/ nurse or from	a) Yes	d) No statement	b) Analysis involves weighted likelihood approach	b) Analysis does not include adjusted variance, or no statement

**Author (year)**	**Study design; outcome assessed**	**Follow-up in the original cohort**	**Selection of cases**	**Selection of controls**	**Ascertainment of 25(OH)D concentrations**	**Demonstration that outcome of interest was not present at start of study**	**Comparability of cohorts on the basis of the design or analysis**	**Assessment of outcome**	**Was follow-up long enough for outcomes to occur?**	**Adequacy of follow up of cohorts**	**C-C: Weighting; NCC: Accounts for unequal probability sampling methods**	**Adjusted variance**
			description	source than cases	iluminescence			health records)				
Thorsen et al. (2017) – Birth cohort [71]	NCC; Juvenile idiopathic arthritis	b) Subjects lost to follow up unlikely to introduce bias	b) Not all cases from the cohort were selected or no description	a) Drawn randomly or matched with cases AND drawn from the same cohort as cases	b) Method used was HPLC, RIA kits, LC-MS/MS OR EIA/Chemiluminescence	a) Not present at start	c) Both a) and b)	e) Both a) and b)	a) Yes	a) Complete follow up - all controls were accounted for	b) Conditional logistic regression analysis with matching variables or stratification	a) Analysis uses adjusted variance

C-C = case-cohort; EIA = Enzyme immunoassay; HPLC = High performance liquid chromatography; LC-MS = Liquid chromatography mass spectrometry; NCC = nested case-control; RIA = Radioimmunoassay

[a] Assessed with an adapted version of *The Newcastle-Ottawa Scale (NOS) for assessing the quality of nonrandomised studies in meta-analyses.*

KQ2. Growth and Neurological Development Outcomes

Six observational studies included in this systematic review assessed the association between serum 25(OH)D concentrations in infancy and outcomes related to growth or neurological development outcomes (**Table KQ2-8**). These studies ranged in size from 134 to 1,550 participants, and study latitudes ranged from 7° S to 65° N. Four cohort studies assessed weight, length/height, or body mass index (BMI) outcomes after six months,[72,73] 18 months,[65] or 19 years.[74] Two of these studies also assessed neurological development determined with the Ages and Stages Questionnaire-3[72] or Børge Priens IQ scores.[74] One nested case-control study assessed changes in 25(OH)D and BMI over three years for children diagnosed with type 1 diabetes and healthy controls[68] while another compared 25(OH)D in children with or without intellectual disabilities at about age three.[75]

All six studies reported no significant linear associations between 25(OH)D levels and growth and development outcomes; however, some subgroup and categorical analyses provided support for possible associations. One study found an inverse relationship between 25(OH)D at infancy and BMI at age 3, but this study did not report if these findings were statistically significant.[68] Another study[65] reported significant benefits in weight-for-length Z-scores at 20 months if 25(OH)D levels at age 5-7 weeks were between 20-29.9 ng/mL compared to less than 10 ng/mL. For all other group comparisons, no significant associations were found.

Two studies reporting on neurological development outcomes found no linear association with 25(OH)D levels from infancy.[72,74] After dividing neonatal 25(OH)D into quintiles, one of these studies[74] found infants with 25(OH)D levels in quintiles three (21.8–30.3 nmol/L) and four (30.3–43.9 nmol/L) had significantly higher IQ scores around age 19 than infants in quintile one (0-13.3 nmol/L). In another study that analyzed 25(OH)D_3 deciles, models predicted the lowest relative risk for intellectual disability by age three years was at the 72nd percentile (48.1 nmol/L).[75]

Among the included cohort studies, the potential for ROB was generally assessed as low (**Table KQ2-9**). Two studies showed potential for bias due to a loss to follow-up rate greater than 20% for certain outcomes with no description of those lost.[72,73] One study used an uncommon method to assess 25(OH)D.[73] Another cohort study included underweight infants in the study population but also measured underweight as an outcome.[65] In this study, unclear follow-up rates were another source of potential bias. One nested case-control was assessed as having high possibility for ROB in three domains due to unclear reporting (**Table KQ2-10**).[68] Regarding selection, it was not clear if 100% of cases from the original cohort were included in the study. While some description of analysis methods was provided, details for how BMI was assessed and analyzed were not clearly presented. Neither nested case-control study mentioned the use of adjusted or robust estimation of the variance which is recommended for studies of this design.

Table KQ2-8. Characteristics and key findings of observational studies reporting the association between serum 25(OH)D and growth or neurological development outcomes

Author (year)	Study design; country (latitude)	Enrollment years	N analyzed (male %)	Mean age (SD) [range][a] at baseline	Breastfeeding status	Race or ethnicity	Health status; nutritional status	Growth outcome analyzed	Mean age (SD) [range] at follow-up	Exposure	Key findings[b]
Chowdhury et al. (2017) [72]	Cohort; Delhi, India (29°)	2010-2012	960 (51)<br>N=401 with neurological measures<br>N=919 with growth measures	~16.2 (7.0-7.1) [6-30] months	Any BF (mixed feeding)	Presumed 100% Asian Indian	34.5% with vitamin D deficiency	Neurodevelopment (ASQ-3 score); weight; length	NR (NR) [12-36] months	Serum 25(OH)D status at 6-30 months: deficient (<25 nmol/L), non-deficient (≥25 nmol/L)	ASQ-3: 0<br>HAZ: 0<br>WHZ: 0<br>WAZ: 0
Makinen et al. (2016) [68]	Nested case control; Oulu, Tampere, and Turku, Finland (60-65°)	1994-2004	252 (50.8)<br>Cases: N=126 (50.8)<br>Controls: N=126 (50.8)	3 (0) months	NR	Presumed 100% Finnish	100% with HLA-conferred susceptibility to T1D, and cases were those who developed T1D; NR	BMI	Median [IQR]:<br>Cases: 3.24 [1.55-5.74] years<br>Controls: 2.53 [1.10-5.20] years	Changes in 25(OH)D from baseline to follow-up	ΔBMI: Inverse association (P-value NR)[c]
Pludowski et al. (2011) [73]	Cohort; Bialystok, Rzeszow, Warsaw, and Kielce, Poland (49-54.5°)	NR	134 (51.5) at enrollment; 98 (NR) at last follow-up	183 (7) days	Any BF (mixed feeding)	Presumed 100% Polish	100% Healthy;	Body weight; body length	365 (7) [NR] days	Changes in 25(OH)D from age 6 months to 12 months	ΔBody weight: 0 (No correlation)<br>ΔBody length: 0 (No correlation)
Specht et al. (2020) [74]	Cohort; Denmark (56°)	1988-1998	818 (95.8)	0 (0) [0-7] days	NR	100% Danish	Generally healthy; NR	BMI; neurological development (IQ score)	19.4 (NR) [17.7–27.8] years	25(OH)D from neonatal DBS as quintiles (nmol/L): 0-13.3 (Q1), 13.3-21.8 (Q2), 21.8–30.3 (Q3), 30.3–43.9 (Q4), and	BMI: 0<br>IQ[d]: 0 (overall), ++ (Q3 vs. Q1; Q4 vs. Q1); 0 (all other quintile comparisons)

Author (year)	Study design; country (latitude)	Enrollment years	N analyzed (male %)	Mean age (SD) [range][a] at baseline	Breastfeeding status	Race or ethnicity	Health status; nutritional status	Growth outcome analyzed	Mean age (SD) [range] at follow-up	Exposure	Key findings[b]
										43.9-104.7 (Q5)	
Sudfeld et al. (2015) [65]	Cohort; Dar es Salaam, Tanzania (-7°)	2004-2007	948 (53.5) in the HIV-exposed, uninfected cohort	[5-7] weeks	Any BF (mixed feeding)	100% Tanzanian	Generally healthy; Low VD status, mean (SD) 25(OH)D at baseline = 18.1 (9.2) ng/mL	Height; weight	Median [IQR]: 20.9 [17.0–23.9] months	Serum 25(OH)D levels (ng/mL) at 5-7 weeks: < 10, 10-19.9, 20-29.9 (ref.), and ≥ 30	LAZ: 0<br>WLZ: 0 (ref. vs. 10-19.9 ng/mL; ref. vs. ≥ 30 ng/mL), ++ (ref. vs. <10 ng/mL[e])<br>WAZ: 0
Wu et al. (2018) [75]	Nested case-control; Beijing, China (39°)	2008-2010	1,550 (77.4)<br>Cases: N=310 (77.4)<br>Controls: N=1,240 (77.4)	3.3 (1.6-1.8) [1-7] days	Any BF (mixed feeding)	100% Chinese	Cases were diagnosed with ASDs; NR	ID (based on IQ[f])	3 (NR) [NR] years	$25(OH)D_3$ from neonatal DBS	ID: ++

ASDs = autism spectrum disorders; ASQ = Ages and Stages Questionnaire; BF = breastfeeding; BMI = body mass index; DBS = dried blood spots; HAZ = height-for-age z-score; HIV = human immunodeficiency virus; ID: intellectual disability; IQ = intelligence quotient; IQR = interquartile range; LAZ = length-for-age z-score; N = sample size; NR = not reported; Q = quintile; ref. = reference group; RR = relative risk; SD = standard deviation; T1D = type 1 diabetes; VD = vitamin D; WAZ = weight-for-age z-score; WHZ = weight-for-height z-score; WLZ = weight-for-length z-score

[a] Measure from baseline or first exposure assessment.
[b] Results: ++ Significant difference indicating benefit of higher serum 25(OH)D levels ($P < 0.05$); + Marginally significant difference indicating benefit ($0.05 < P < 0.1$); 0 No significant difference; - Marginally significant difference indicating detriment ($0.05 < P < 0.1$); -- Significant difference indicating detriment ($P < 0.05$)
[c] According to Makinen et al. (2016) [68], "when BMI increased with 1 kg/m^2, median 25(OH)D concentration decreased with 1.3 nmol/L. The effect was similar in cases and controls ($P = .57$)" (p. 727).
[d] Adjusted mean Børge Priens IQ test scores according to quintiles of neonatal vitamin D levels. Results for linear regression model adjusted for maternal age at birth, maternal and paternal education level, gestational age at birth, and season of birth.
[e] According to Sudfeld et al. (2015) [65], "In multivariate analysis, the trajectory of WLZ significantly differed for infants with 25(OH)D concentrations of <10 ng/mL compared with those with concentrations of 20–29.9 ng/mL ($P < 0.01$). Infants with 25(OH)D concentrations of <10 ng/mL experienced a rapid decrease in WLZ from 10–38 wk of age, but then experienced catch-up, and WLZ was comparable to other groups by 110 wk of age" (p. 126).
[f] Intellectual disability defined as IQ < 80 on the Combined Raven's Test after conversion to Chinese children's norms.

Table KQ2-9. Risk of bias assessment results for cohort studies[a] reporting on growth or neurological development outcomes

Author (year)	Outcome assessed	Selection of the non-exposed cohort	Ascertainment of 25(OH)D concentrations	Demonstration that outcome of interest was not present at start of study	Comparability of cohorts on the basis of the design or analysis	Assessment of outcome	Was follow-up long enough for outcomes to occur?	Adequacy of follow up of cohorts
Chowdhury et al. (2017) [72]	Weight; length	a) Drawn from the same community as the exposed cohort	b) Method used was HPLC, RIA kits, LC-MS/MS OR EIA/Chemiluminescence	a) Not present at start	c) Both a) and b)	a) Independent blind assessment	a) Yes	b) Subjects lost to follow up unlikely to introduce bias
Chowdhury et al. (2017) [72]	Neurological development	a) Drawn from the same community as the exposed cohort	b) Method used was HPLC, RIA kits, LC-MS/MS OR EIA/Chemiluminescence	a) Not present at start	b) Study controls for 3+ important factors AND any of the other factors, gives justification for non-inclusion	a) Independent blind assessment	a) Yes	c) Lost to follow up rate ≥ 20% and no description of those lost
Pludowski et al. (2011) [73]	Body weight; body length	a) Drawn from the same community as the exposed cohort	c) Other methods were used to assess 25(OH)D or no description	a) Not present at start	c) Both a) and b)	a) Independent blind assessment	a) Yes	c) Lost to follow up rate ≥ 20% and no description of those lost
Specht et al. (2020) [74]	Body mass index	a) Drawn from the same community as the exposed cohort	b) Method used was HPLC, RIA kits, LC-MS/MS OR EIA/Chemiluminescence	a) Not present at start	c) Both a) and b)	b) Record linkage	a) Yes	a) Complete follow up - all subjects were accounted for
Specht et al. (2020) [74]	Neurological development (IQ score)	a) Drawn from the same community as the exposed cohort	b) Method used was HPLC, RIA kits, LC-MS/MS OR EIA/Chemiluminescence	a) Not present at start	c) Both a) and b)	b) Record linkage	a) Yes	a) Complete follow up - all subjects were accounted for
Sudfeld et al. (2015) [65]	Height; weight	a) Drawn from the same	b) Method used was HPLC, RIA	b) Present at start or unknown	c) Both a) and b)	a) Independent blind assessment	a) Yes	d) No statement

Author (year)	Outcome assessed	Selection of the non-exposed cohort	Ascertainment of 25(OH)D concentrations	Demonstration that outcome of interest was not present at start of study	Comparability of cohorts on the basis of the design or analysis	Assessment of outcome	Was follow-up long enough for outcomes to occur?	Adequacy of follow up of cohorts
		community as the exposed cohort	kits, LC-MS/MS OR EIA/Chemiluminescence					

EIA = Enzyme immunoassay; HPLC = High performance liquid chromatography; LC-MS = Liquid chromatography mass spectrometry; RIA = Radioimmunoassay

[a] Assessed with *The Newcastle-Ottawa Scale (NOS) for assessing the quality of nonrandomised studies in meta-analyses.*

Table KQ2-10. Risk of bias assessment results for nested case-control studies[a] reporting on growth or neurological development outcomes

**Author (year)**	**Outcome assessed**	**Follow-up in the original cohort**	**Selection of cases**	**Selection of controls**	**Ascertainment of 25(OH)D concentrations**	**Demonstration that outcome of interest was not present at start of study**	**Comparability of cohorts on the basis of the design or analysis**	**Assessment of outcome**	**Was follow-up long enough for outcomes to occur?**	**Adequacy of follow up of cohorts**	**Accounts for unequal probability sampling methods**	**Adjusted variance**
Makinen et al. (2016) [68]	Body mass index	c) Lost to follow up rate ≥ 20% and no description of those lost	b) Not all cases from the cohort were selected or no description	a) Drawn randomly or matched with cases AND drawn from the same cohort as cases	b) Method used was HPLC, RIA kits, LC-MS/MS OR EIA/Chemiluminescence	a) Not present at start	Not clear from reporting	d) No description	a) Yes	b) Controls lost to follow up unlikely to introduce bias	Not clear from reporting	b) Analysis does not include adjusted variance, or no statement
Wu et al. (2018) [75]	Intellectual development (IQ)	b) Subjects lost to follow up unlikely to introduce bias	a) All cases from the cohort were selected	a) Drawn randomly or matched with cases AND drawn from the same cohort as cases	d) Both a) and b)	a) Not present at start	c) Both a) and b)	a) Independent blind assessment (e.g., by physician/ nurse or from health records)	a) Yes	a) Complete follow up - all controls were accounted for	b) Conditional logistic regression analysis with matching variables or stratification	b) Analysis does not include adjusted variance, or no statement

EIA = Enzyme immunoassay; HPLC = High performance liquid chromatography; LC-MS = Liquid chromatography mass spectrometry; RIA = Radioimmunoassay

[a] Assessed with an adapted version of *The Newcastle-Ottawa Scale (NOS) for assessing the quality of nonrandomised studies in meta-analyses.*

KQ2. Fracture Outcome

One large case-cohort study (n = 2,604; ~47.7% male) conducted in Denmark (~55°-58° N) reported on the association between 25(OH)D_3 levels at birth and bone fracture outcomes when participants were 6-13 years old.[76] **Table KQ2-11** shows the characteristics of this study where cases were identified through the Danish National Patient Registry (NPR), and fractures of interest were forearm, wrist, or scaphoid bone (International Classification of Diseases, 10th edition [ICD-10]: S52, S62.0); the clavicle (ICD-10: S42.0); or the ankle (ICD-10:S82.5, S82.6, S82.8). This study found no association between serum 25(OH)D_3 at birth as a continuous measure and fracture risk at age 6-13 years. When 25(OH)D_3 quintiles were compared, participants in the middle quintile (mean 25[OH]D_3 = 23.90 nmol/L) had significantly lower odds of fracture compared to participants in the lowest quintile (mean 25[OH]D_3 = 7.88 nmol/L; crude OR = 0.76; 95% CI 0.60, 0.98). This trend remained after adjusting for age, sex, parity, maternal education level, maternal ethnicity, and maternal age (adjusted OR = 0.75; 95% CI 0.58, 0.96). Other models adding either maternal smoking during pregnancy, season of birth, or birth weight to the adjusted model also resulted in lower odds of fracture for the middle quintile compared to the lowest quintile. In global tests, no significant overall association was found for any model (*P*-value range: 0.08 – 0.16).

There was some potential for risk of bias in the selection of participants, as 100% of cases were not included in the study; rather, a sample was selected randomly from all eligible cases (**Table KQ2-12**). Due to this deviation from the classic case-cohort design, the study analysis did not include weighting or robust adjustments for variance which resulted in additional potential for risk of bias.

KQ2. Blood Pressure

A cohort study (n = 284, 56.3% male) conducted in Sweden (60° N) assessed the association between 25(OH)D_3 levels at birth and blood pressure outcomes at age 35 years (**Table KQ2-13**).[77] This study utilized stored dried blood samples which, when tested, showed signs of potential for 25(OH)D_3 degradation. Due to this, 25(OH)D_3 was assessed only as a continuous measure. Regression results suggested no association between 25(OH)D_3 at birth and systolic (95% CI -1.32, 6.21; P = 0.20) or diastolic (95% CI -2.46, 3.22; P = 0.79) blood pressure at age 35 years after adjusting for sex, postnatal age at sample collection, season of birth, preterm birth, maternal age, education, smoking, fish consumption per week, exercise per week, and current 25(OH)D. A similar trend was found when current BMI was added to the model (systolic 95% CI -2.67, 4.79; P = 0.58; diastolic 95% CI -3.56, 2.04; P = 0.60).

In this study, there was a likelihood of low ROB for both the selection and comparability domains (**Table KQ2-14**). There were some concerns for the outcome domain due to a loss to follow-up rate of nearly 80%. With a 35-year follow-up duration, "rejection or no response to invitation" accounted for 79% of those lost to follow-up. The paper states, "Response rates were not influenced by sex or season of birth";[77] however, nothing was reported regarding differences between levels of 25(OH)D at birth for those who did or did not participate.

Table KQ2-11. Characteristics and key findings of one case-cohort study reporting on fracture outcomes

Author (year)	Study design; country (latitude)	Enrollment year(s)	N analyzed (male %)	Mean age (SD) at baseline	Breastfeeding status	Race or ethnicity[a]	Health status; nutritional status	Exposure	Mean age [range] at follow-up	Key findings[b]
Handel et al. (2017) [76]	Case-cohort; Denmark (~55°-58°)	1989-1999	2,604 (~47.7%)<br>Cases: N=1,039 (49.9)<br>Controls: N=1,565 (52.3)	Cases GA: 39.5 (1.8) weeks<br>Controls GA: 39.6 (2.0) weeks	NR	Cases: 95.7% European;<br>Controls: 92.3% European	NR; low VD3 levels	25(OH)D_3 from DBS at birth	NR [6-13] y	Odds of fracture at age 6-13 y by mean 25(OH)D_3 at birth: ++ (middle quintile [23.90 nmol/L] vs. lowest quintile [7.88 nmol/L])<br>Risk of fracture at age 6-13 y by continuous 25(OH)D_3 at birth: 0

GA = gestational age; DBS = dried blood spots; N = sample size; NR = not reported; SD = standard deviation; VD3 = vitamin D_3; y = years

[a] Maternal race/ethnicity reported.

[b] Results comparing higher to lower levels of serum 25(OH)D: ++ Significant beneficial effects of higher 25(OH)D levels ($P < 0.05$); + Marginally significant beneficial effects ($0.05 < P < 0.1$); 0 No effects; - Marginally significant detrimental effects ($0.05 < P < 0.1$); -- Significant detrimental effects ($P < 0.05$).

Table KQ2-12. Risk of bias assessment results for one case-cohort study[a] reporting on fracture outcomes

Author (year)	Study design; outcome assessed	Follow-up in the original cohort	Selection of cases	Selection of controls	Ascertainment of 25(OH)D concentrations	Demonstration that outcome of interest was not present at start of study	Comparability of cohorts on the basis of the design or analysis	Assessment of outcome	Was follow-up long enough for outcomes to occur?	Adequacy of follow up of cohorts	Weighting	Adjusted variance
Handel et al. (2017) [76]	C-C; Fracture	b) Subjects lost to follow up unlikely to introduce bias	b) Not all cases from the cohort were selected or no description	a) Drawn randomly or matched with cases AND drawn from the same cohort as cases	b) Method used was HPLC, RIA kits, LC-MS/MS OR EIA/Chemiluminescence	a) Not present at start	a) Study controls for at least 4/6 of the important factors or gives justification for non-inclusion	e) Both a) and b)	a) Yes	b) Controls lost to follow up unlikely to introduce bias	d) Analysis does not include weighting or no statement	b) Analysis does not include adjusted variance, or no statement

EIA = Enzyme immunoassay; HPLC = High performance liquid chromatography; LC-MS = Liquid chromatography mass spectrometry; RIA = Radioimmunoassay

[a] Assessed with an adapted version of *The Newcastle-Ottawa Scale (NOS) for assessing the quality of nonrandomised studies in meta-analyses.*

Table KQ2-13. Characteristics and key findings of a cohort study reporting on blood pressure outcomes

Author (year)	Study design; country (latitude)	Enrollment year(s)	N analyzed (male %)	Mean age (SD) at baseline	Breastfeeding Status	Race or ethnicity	Health status; nutritional status	Exposure	Mean age (SD) at follow-up	Key findings[a]
Tornhammar et al. (2014) [77]	Cohort; Sweden (60°)	1975; 2010 (follow-up)	N=284 (56.3%)	~4.8 (1.2-1.3) days	NR	100% Swedish	NR; NR	25(OH)D_3 from DBS at birth	35 (NR) y	Systolic BP at age 35 y: 0<br>Diastolic BP at age 35 y: 0

BP = blood pressure; DBS = dried blood spots; N = sample size; NR = not reported; SD = standard deviation; y = years

[a] Results comparing higher to lower levels of serum 25(OH)D: ++ Significant beneficial effects of higher 25(OH)D levels ($P < 0.05$); + Marginally significant beneficial effects ($0.05 < P < 0.1$); 0 No effects; - Marginally significant detrimental effects ($0.05 < P < 0.1$); -- Significant detrimental effects ($P < 0.05$).

Table KQ2-14. Risk of bias assessment results for one cohort study[a] reporting on blood pressure outcomes

Author (year)	Outcome assessed	Selection of the non-exposed cohort	Ascertainment of 25(OH)D concentrations	Demonstration that outcome of interest was not present at start of study	Comparability of cohorts on the basis of the design or analysis	Assessment of outcome	Was follow-up long enough for outcomes to occur?	Adequacy of follow up of cohorts
Tornhammar et al. (2014) [77]	Blood pressure	a) Drawn from the same community as the exposed cohort	b) Method used was HPLC, RIA kits, LC-MS/MS OR EIA/Chemiluminescence	a) Not present at start	c) Both a) and b)	a) Independent blind assessment	a) Yes	c) Lost to follow up rate ≥ 20% and no description of those lost

EIA = Enzyme immunoassay; HPLC = High performance liquid chromatography; LC-MS = Liquid chromatography mass spectrometry; RIA = Radioimmunoassay

[a] Assessed with *The Newcastle-Ottawa Scale (NOS) for assessing the quality of nonrandomised studies in meta-analyses.*

KQ3. What is the effect of vitamin D intake on serum 25(OH)D concentrations in children aged 0 to 4 years?

Table KQ3-1 reports the characteristics of interventional studies reporting the effect of vitamin D intake on serum 25-hydroxyvitamin D [25(OH)D] included in our review. Of the 67 studies (from 66 publications), 51 studies were conducted in children ages 0 to 4 years,[24,27-49,51-54,57,78-100] while 16 studies (from 14 publications) were conducted in children ages 3-9 years.[56,101-114]

To perform planned subgroup analyses (see Background), we first categorized the included studies into 5 sub-sections: 1) studies comparing different levels of daily vitamin D supplementation (vitamin D_3 or D_2 supplements), 2) studies using non-daily vitamin D supplementation dosing regimens (including single dose), 3) studies comparing supplementation to post-partum mothers to supplementation to infants, 4) studies using food interventions with different levels of vitamin D or comparing food interventions with vitamin D supplements, and 5) studies examining the effects of combined vitamin D and calcium supplementation. Within each section, we then plotted the mean 25(OH)D concentration and its 95% confidence interval at baseline (if reported) and at follow-up time points for all intervention arms of each included study to summarize the individual study results. Results of all follow-up time points were included; however, results from the post-intervention follow-up time points were excluded.

Table KQ3-1. Characteristics of all studies reporting the effect of vitamin D on serum 25(OH)D included in our review

Author (year)	Study design; N randomized	Enrollment years	Location; latitude	Mean age (SD) [range]	Male (%)	Breastfeeding status	Race or ethnicity	Health status; nutritional status	Assay method	Assay standards
Abrams et al. (2013) [101]	RCT; N= 64	2009-2011	Houston, USA; 29.7°	6.6 (1.4) [4-8.9] years	NR	NR	NR	100% Healthy; NR	EIA/Chemi-luminescence	CDC: yes; NIST: no
Aglipay et al. (2017) [32]	RCT; N= 703	2011-2015	Toronto, Canada; 43°	2.7 (1.5) years	57.4	Any BF	NR	100% Healthy; NR	Protein-binding assay	CDC: yes; NIST: no
Ala-houhala et al. (1985) [51]	RCT; N= 92	1982	Tampere, Finland; 61°	Neonates	NR	Exclusively BF	NR	100% Healthy; NR	HPLC	CDC: no; NIST: no
Ala-Houhala et al. (1986) [78]	RCT; N= 16	1984	Tampere, Finland; 61°	Neonates	NR	Exclusively BF	NR	100% Healthy; NR	Competitive protein binding assay	CDC: no; NIST: no
Alahouhala et al. (1988) [102]	RCT; N= 51	1984-1985	Tampere, Finland; 61°	[8-10 years]	NR	NA	NR	100% Healthy; NR	HPLC	CDC: no; NIST: no
Alonso et al. (2011) [33]	RCT; N= 88	2007-2008	Spain; 43°	Neonates	52.3	Any BF	NR	100% Healthy; NR	EIA/Chemi-luminescence	CDC: yes; NIST: no
Aluisio et al. (2013) [79]	RCT; N= 3046	2007-2009	Kabul, Afghanistan; 34.5°	0.5 [0.08-0.92] years	NR	NR	NR	100% Healthy; NR	EIA/Chemi-luminescence	CDC: yes; NIST: no
Atas et al. (2013) [80]	RCT; N= 169	2006-2007	Istanbul, Turkey; 40°	Neonates	NR	Exclusively BF	NR	100% Healthy; NR	HPLC	CDC: no; NIST: no
Brett et al. (2016) [103]	RCT; N= 77	2014	Montreal, Canada; 45.5°	5.1 (1.9) years	54.5	NA	NR	100% Healthy; NA	EIA/Chemi-luminescence	CDC: yes; NIST: yes (mean bias: NR) "
Brett et al. (2018) [104]	RCT; N= 51	2014	Montreal, Canada; 46°	~5.2 (1.8-2.0) [1.9-8.6] years	52.9	NA	60.8% White; 39.4% non-white (Hispanic, Black, or Asian)	100% Healthy; <8% with low vitamin D	HPLC	CDC: no; NIST: yes (mean bias: <5%)
Chan et al. (1982) [43]	RCT; N= 91	NR	Salt Lake City, USA; NR	Neonates	NR	Any BF	100% White	100% Healthy; NR	Competitive protein	CDC: no; NIST: no

Author (year)	Study design; N randomized	Enrollment years	Location; latitude	Mean age (SD) [range]	Male (%)	Breastfeeding status	Race or ethnicity	Health status; nutritional status	Assay method	Assay standards
									binding radioassay	
Chandy et al. (2016) 45	RCT; N= 230	2012-2014	India; 26°	Neonates	NR	Any BF	100% Asian Indian	100% Healthy; NR	RIA kits	CDC: yes; NIST: no
Dawodu et al. (2019) 46	RCT; N= 190	2013-2016	Dohar, Qatar; 25°	Neonates	NR	Exclusively BF	NR	100% Healthy; Low vitamin D intake	EIA/Chemi-luminescence	CDC: yes; NIST: no
Economos et al. (2014) 105	RCT; N= 176	2005-2006	Massachusetts, USA; 42°	8.0 (1.4) years	61	NA	2.1% Asian; 44.0% Black; 24.8% White; 12.8% Hispanic; 16.3% other	100% Healthy; NR	Protein binding assay	CDC: yes; NIST: no
Ekbote et al. (2011) 81	RCT; N= 60	2007-2008	Pune, India; 19°	2.7 (0.52) [1-5] years	52	NR	Presumed 100% Asian Indian	100% Healthy; NR	EIA/Chemi-luminescence	CDC: yes; NIST: no
Enlund-Cerullo et al. (2019) 34	RCT; N= 913	2013-2016	Helsinki, Finland; 60.2°	NR [0-2 years]	50.3	Any BF	100% of mothers of Northern European Origin	100% Healthy; NR	EIA/Chemi-luminescence	CDC: yes; NIST: yes (mean bias: <8% positive)
Gallo et al. (2013)a 82	RCT; N= 52	2010-2011	Montreal, Canada; 46°	1 month	48	Any BF	NR	100% Healthy; NR	LC-MS/MS	CDC: no; NIST: no
Gallo et al. (2013)b 38	RCT; N= 132	2007-2010	Montreal, Canada; 46°	Neonates	57.6	Exclusively BF	84% White, 16% non-white	100% Healthy; NR	LC-MS	CDC: yes; NIST: no
Gordon et al. (2008) 83	RCT; N= 40	2005-2007	Boston, USA; 42°	Neonates	45	NR	NR	100% Healthy; 100% vitamin D deficiency	EIA/Chemi-luminescence	CDC: yes; NIST: no
Grant et al. (2014) 84	RCT; N= 260	2010-2011	Auckland, New Zealand; -36°	Neonates	48.6	Any BF	NR	100% Healthy; NR	LC-MS/MS	CDC: yes; NIST: no
Grant et al. (2015) 30	RCT; N= 236	2010-2011	Auckland, New Zealand; -36°	Neonates	NR	Any BF	NR	100% Healthy; NR	LC-MS/MS	CDC: yes; NIST: no
Greer et al. (1981) 98	RCT; N=30	1978	NR; NR	Neonates	NR	Exclusively BF	94% Caucasian; 6% Asian Indian	100% Healthy; NA	NR	CDC: no; NIST: no

**Author (year)**	**Study design; N randomized**	**Enrollment years**	**Location; latitude**	**Mean age (SD) [range]**	**Male (%)**	**Breastfeeding status**	**Race or ethnicity**	**Health status; nutritional status**	**Assay method**	**Assay standards**
Greer et al. (1982) [37]	RCT; N= 30	1978	NR; NR	Neonates	NR	Exclusively BF	94% White; 6% Asian Indian	100% Healthy; NA	NR	CDC: no; NIST: no
Harnot et al. (2017) [85]	RCT; N= 60	2012-2013	Chandigarh, India; 31°	1.2 (0.8) [0-3] years	68.3	NR	Presumed 100% Asian Indian	100% with evidence of VitD deficiency; Low vitamin D intake	EIA/Chemi-luminescence	CDC: yes; NIST: no
Hibbs et al. (2018) [24]	RCT; N= 300	2013-2016	Cleveland, USA; Charleston, USA; Bronx, USA; ~38°	Neonates	55	Any BF	100% Black or African American	100% preterm (mean GA=33); NR	EIA/Chemi-luminescence	NIST: no
Hirschler et al. (2014) [106]	Cluster RCT; N= 96	2011-2013	Salta, Argentina; -24°	8.8 (1.8) years	46.9	NA	100% South American indigenous children	100% Healthy; Low vitamin D intake	EIA/Chemi-luminescence	CDC: yes; NIST: no
Hollis et al. (2015) [86]	RCT; N= 216	2005 -2012	Charleston, Rochester, Morgantown, USA; 33°, 43°	NR [4-6] weeks	NR	Any BF	23% Black; 51% White; 26% Hispanic	100% Healthy; NR	RIA kits	CDC: no; NIST: no
Holmlund-Suila et al. (2012) [35]	RCT; N= 113	2010-2011	Helsinki, Finland; 60.2°	Neonates	50.4	Any BF	NR	100% Healthy; NR	EIA/Chemi-luminescence	CDC: unclear; NIST: no
Holstgemeiner et al. (1978) [87]	RCT; N= 21	1976	Vienna, Austria; 48°	Neonates	23.8	Any BF	Presumed 100% Austrian	Generally healthy;	NR	CDC: no; NIST: no
Huynh et al. (2017) [36]	RCT; N= 70	2013-2014	St. Albans, Australia; -38°	Neonates	NR	Any BF	NR	100% Healthy; VD deficiency	EIA/Chemi-luminescence	CDC: no; NIST: no
Karlsland Akeson et al. (dark skinned children) (2018) [107]	RCT; N= 206	NR	Malmo and Umea Sweden; 55° and 63°	6.3 years	NR	NR	Presumed 100% Swedish	100% Healthy; NA	LC-MS/MS	CDC: yes; NIST: no

Author (year)	Study design; N randomized	Enrollment years	Location; latitude	Mean age (SD) [range]	Male (%)	Breastfeeding status	Race or ethnicity	Health status; nutritional status	Assay method	Assay standards
Karlsland Akeson et al. (fair-skinned children) (2018) [107]	RCT; N= 206	NR	Malmo and Umea Sweden; 55° and 63°	6.3 years	NR	NR	Presumed 100% Swedish	100% Healthy; NA	LC-MS/MS	CDC: yes; NIST: no
Kumar et al. (2011) [42]	RCT; N= 2079	2007-2010	New Delhi, India; 29°	Neonates	46.7	Any BF	Presumed 100% Asian Indian	100% with low birthweight (range 1.8-2.5 kg); 100% with severe VD deficiency	RIA kits	CDC: yes; NIST: no
Kunz et al. (1982) [88]	RCT; N= 29	NR	Bonn, Germany; 48°	Neonates	NR	NR	Presumed 100% German	Generally healthy; Low vitamin D intake	NR	CDC: no; NIST: no
Loeb et al. (2019) [108]	RCT; N= 1,300	2013-2016	Thanh Ha, Vietnam; 21.1°	8.6 [3-17] years	50	NR	NR	100% Healthy; NR	EIA/Chemi-luminescence	CDC: yes; NIST: no
Madar et al. (2009) [89]	RCT; N= 66	2004-2006	Oslo, Norway; 60°	0.13 (0.03) years	NR	Any BF	Pakistani, Turkish or Somali background	100% Healthy; Low vitamin D intake	HPLC	CDC: no; NIST: no
Manaseki-Holland et al. (2012) [28]	RCT; N= 3046	2008-2009	Kabul, Afghanistan; 34.6°	~0.54 [0.17-1] years	52	Any BF	Father ethnicity: Tajik, Pashton, Uzbek, Hazara, or other	Generally healthy; Malnourished	EIA/Chemi-luminescence	CDC: yes; NIST: no
Mandlik et al. (2019) [109]	Controlled Trial; N= 435	2014-2015	Pune, Western India; 18.5°	~8.12 (~1.2) years	54.5	NR	100% Asian Indian	66% vit D deficient; NR	EIA/Chemi-luminescence	CDC: no; NIST: no
Marwaha et al. (2018) [110]	RCT; N= 240	2015-2016	Delhi, India; 29°	8.9 [6.1-11.8] years	0	NA	Presumed 100% Asian Indian	100% Healthy; Low vitamin D intake	EIA/Chemi-luminescence	CDC: yes; NIST: no
Mittal et al. (2014) [99]	RCT; N= 76	2010-2012	Delhi, India; ~29°	~17.5 (13.2-14.4) months	55	NR	Presumed 100% Asian Indian	100% with clinical evidence of rickets	RIA kits	CDC: yes NIST: no

Author (year)	Study design; N randomized	Enrollment years	Location; latitude	Mean age (SD) [range]	Male (%)	Breastfeeding status	Race or ethnicity	Health status; nutritional status	Assay method	Assay standards
Mittal et al. (2018) [100]	RCT; N= 110	NR	Delhi, India; ~29°	10.5 months [6 months – 5 years]	60	NR	Presumed 100% Asian Indian	100% with radiographic evidence of rickets	EIA/Chemi-luminescence	CDC: yes NIST: no
Moodley et al. (2015) [90]	RCT; N= 51	2011-2012	Tijuana, Mexico; ~32.5°	Neonates	51	Any BF	100% Hispanic	100% Healthy; Low vitamin D intake	LC-MS/MS	CDC: no NIST: no
Mortensen et al. (2016) [111]	RCT; N= 130	2014-2015	Copenhagena and Frederiksberg Denmark; 55°	6.6 (1.5) [4-8] years	47	NR	NR	100% Healthy; NR	LC-MS/MS	CDC: yes; NIST: no
Natarajan et al. (2014) [39]	RCT; N= 96	2011-2012	Northern India; ~29°	Neonates	56.3	Any BF	Presumed 100% Asian Indian	100% preterm infants (mean GA = 32.5); 81% with VD deficiency	EIA/Chemi-luminescence	CDC: yes; NIST: no
Ohlund et al. (2017) [112]	RCT; N= 206	2012-2013	Sweden; NA°	6.3 [5-7] years	51.1	NR	NR	100% Healthy; NR	LC-MS/MS	CDC: yes; NIST: no
Pittard (term infants only) et al. (1991) [91]	RCT; N= 25	NR	Charleston, US; 32.8°	Neonates	44	Exclusively formula	NR	100% Healthy; NR	Competitive protein binding assays	CDC: no; NIST: no
Ponnapakkam et al. (2010) [53]	RCT; N= 80	NR	Louisiana, USA; ~30°	Neonates	NR	Any BF	NR	Not specified but presumably healthy; Presumably normal	EIA/Chemi-luminescence	CDC: yes; NIST: no
Rao et al. (2016) [56]	RCT; N= 45	NR	Kanpur, India; 26°	~3.1 (~0.7-0.9) [2.5] years	NR	NR	Presumed 100% Asian Indian	NR; 100% with VD deficiency (<20 ng/mL)	EIA/Chemi-luminescence	CDC: no NIST: no
Rosendahl et al. (2018)	RCT; N= 975	2013-2014	Helsinki, Finland; 60.2°	Neonates	50.3	Any BF	100% of mothers of	100% Healthy; 95.7% with	EIA/Chemi-luminescence	CDC: yes; NIST: yes

Author (year)	Study design; N randomized	Enrollment years	Location; latitude	Mean age (SD) [range]	Male (%)	Breastfeeding status	Race or ethnicity	Health status; nutritional status	Assay method	Assay standards
29							Northern European Origin	vitamin D sufficiency		(mean bias: <8%)
Rueter et al. (2019) 27	RCT; N= 195	2012-2017	Perth, Australia; 32°	Neonates	53	NR	NR	100% Healthy; NR	EIA/Chemi-luminescence	CDC: yes; NIST: no
Shajari et al. (2009) 92	RCT; N= 90	NR	Yazd, Iran; 32°	Neonates	NR	Exclusively BF	Presumed 100% Iranian	100% Healthy; Presumably normal	NR	CDC: no NIST: no
Shakiba et al. (2010) 93	RCT; N= 75	2007	Yazd, Iran; 32°	Neonates	46.7	Any BF	NR	100% Healthy; NR	EIA/Chemi-luminescence	CDC: yes; NIST: no
Shakiba et al. (2014) 94	Non-randomized trial; N= 83	2010	Yazd, Iran; 32°	Neonates	49.4	Exclusively BF	NR	100% Healthy; NR	EIA/Chemi-luminescence	CDC: yes; NIST: no
Sharma et al. (2016) 95	RCT; N= 132	2007-2010	Montreal, Canada; 46°	1 month	57.6	Any BF	84% White; 15% non-white	100% Healthy; NR	LC-MS/MS	CDC: no; NIST: no
Siafarikas et al. (2011) 52	RCT; N= 40	NR	Berlin, Germany; 52.5°	Neonates	NR	Any BF	NR	100% Healthy; NR	RIA kits	CDC: no; NIST: no
Singh et al. (2018) 44	RCT; N= 100	2013-2014	New Delhi, India; 29°	Neonates	55	Exclusively BF	Presumed 100% Asian Indian	100% Healthy; ~47% with VD deficiency	EIA/Chemi-luminescence	CDC: no; NIST: no
Stellinga-Boelen et al. (2007) 113	RCT; N= 135	2003	Groningen, The Netherlands; 53°	7.1 [2-12] years	58	NR	NR	100% Healthy; estimated VD intake ranged: 0.1-6.0 µg (median 1.2 µg)	RIA kits	CDC: yes; NIST: no
Talaat et al. (2016) 114	RCT; N= 637	2014	Taif region, KSA; 21.4°	8.5 (3.53) [2-18] years	49.3	NR	NR	100% Healthy; Low vitamin D intake	EIA/Chemi-luminescence	CDC: yes; NIST: no
Trilok-Kumar et al. (2015) 48	RCT; N= 912	2007-2010	New Delhi, India; 29°	Neonates	52.1	Any BF	Presumed 100% Asian Indian	Low birth weight (1.8 to <2.5 kg);	RIA kits	CDC: yes; NIST: no

Author (year)	Study design; N randomized	Enrollment years	Location; latitude	Mean age (SD) [range]	Male (%)	Breastfeeding status	Race or ethnicity	Health status; nutritional status	Assay method	Assay standards
								~50% with vitamin D deficiency at follow-up		
Wagner et al. (2006) [41]	RCT; N= 19	NR	Charleston, South Carolina, United States; 33°	Neonates	47	Exclusive or fully BF	White: 79% Hispanic: 11% Black: 11%	100% Healthy; NR	NR	NR
Wicklow et al. (2016) [47]	RCT; N= 55	2009-2011	Montreal, Canada; 46°	Neonates	56.4	Any BF	87.3% White; 12.7% non-white	100% Healthy; NR	LC-MS/MS	CDC: no; NIST: no
Zeghoud et al. (1991-1992 study) (1994) [96]	RCT; N= 30	1991-1992	Constantine, Algeria; 36°	NR [0-0.75 years]	NR	NR	NR	100% Healthy; Low vitamin D intake	RIA kits	CDC: no; NIST: no
Zeghoud et al. (1997) [97]	RCT; N= 80	1994	Compiegne, France; 49°	Neonates	NR	NR	99% Mothers of European extraction	100% Healthy; NR	RIA kits	CDC: no; NIST: no
Zhou et al. (2018) [31]	RCT; N= 400	2015-2016	Yongkang, China; Wenzhou, China; Jinhua, China; ~29°	Neonates	52.3	Any BF	Presumed 100% Chinese	Generally healthy; Presumed normal	NR	CDC: no NIST: no
Ziegler et al. (2014) [40]	RCT; N= 213	2006-2010	Iowa City, USA; 41°	1 month	NR	Exclusively BF		100% Healthy; Normal	RIA kits	CDC: yes; NIST: no

BF = Breast Feeding; CDC = Centers for Disease Control and Prevention; EIA = Enzyme immunoassay; GA = gestational age; HPLC = High performance liquid chromatography; LC-MS = Liquid chromatography mass spectrometry; NIST = National Institute of Standards and Technology; NA = Not applicable; NR = Not reported; RCT = Randomized controlled trial; RIA = Radioimmunoassay; VD = Vitamin D

KQ3. Studies comparing different levels of daily vitamin D supplementation

Total of 38 unique studies (in 39 publications) examining the effects of daily vitamin D intake on serum 25(OH)D concentration were included, of which, 30 trials (in 31 publications) were conducted in children 0 to 12 months,[24,27,29,31,33-40,43-46,52,53,78,80,82-84,86-89,91,93,95,97] one was conducted in children 1 to 4 years,[32] and seven were conducted in children 3 to 9 years.[101-103,109-111,114] The characteristics of these studies are reported in Table KQ3-1.

Children 0-12 months old

Thirty trials reported in 31 publications (all RCTs) examining the effects of daily vitamin D intake on serum 25(OH)D concentration in children 0 to 12 months were included.[24,27,29,31,33-40,43-46,52,53,78,80,82-84,86-89,91,93,95,97] The individual study results are shown in **Figures KQ3-1a and KQ3-1b**. The doses of vitamin D supplementation (mostly vitamin D_3) ranged from 200 to 2,000 IU/d across the 30 RCTs, and of these, 11 RCTs included a non-vitamin D supplementation comparison group (placebo, no intervention, or maternal supplementation). Intervention durations ranged from six to 94 weeks. Sample sizes ranged widely from five to 459 subjects per intervention arm.

Figure KQ3-2 shows the summary and individual risk of bias (ROB) plots for trials reporting the effect of vitamin D intake on serum 25(OH)D concentration in children from birth to 12 months of age. More than 75% of trials had some or high ROB due to deviations from intended interventions, especially issues of non-adherence and how they were accounted for in the statistical methods. More than 75% of trials also had some or high ROB due to selection of the reported results. More than 50% of trials had some ROB in the randomization process and some or high ROB due to missing outcome data. Nearly all studies (>95% of studies) had low ROB in measurement of the outcome.

Children 1 to 4 years old

Only one study examining the effects of daily vitamin D intake on serum 25(OH)D concentration conducted in children 1 to 4 years was included.[32] The study results are shown in Figure KQ3-1b. In the study, children were randomized to either 400 IU/d or 2,000 IU/d of vitamin D_3. At 16 weeks follow-up, serum 25(OH)D remained unchanged in the 400 IU/d group but had significantly increased from 89.6 to 121.6 nmol/L in the 2,000 IU/d group. The study was assessed as having high ROB due to deviations from intended intervention, specifically surrounding to issues of adherence and statistical analysis; however, the study was assessed as having low ROB in all other domains.

Random-effects meta-regression analysis of studies conducted in children 0 to 4 years showed that each 100 IU/d increase in vitamin D supplementation was associated with an average of 1.92 (95% CI 0.28, 3.56) nmol/L increase in achieved 25(OH)D concentration (n = 53 intervention arms; P = 0.022; adjusted R^2 = 9.07%). However, the residual heterogeneity is large (I^2 = 99.39%) (see **Table KQ3-2**; **Figure KQ3-3**).

Table KQ3-2. Random-effects meta-regression analysis to examine the association between daily vitamin D supplementation doses and achieved 25(OH)D concentrations at the end of intervention period in children 0 to 4 years[a]

Meta-regression					Number of observation = 53[b]	
REML estimate of between-study variance					tau^2 = 662.6	
% residual variation due to heterogeneity					I^2 residual = 99.39%	
Proportion of between-study variance explained with Knapp-Hartung modification					Adj R-squared = 9.07%	
	Coef.	Std. Err.	t	P>t	[95% CI]	
Vitamin D daily dose (IU)	0.0192	0.0082	2.3500	0.0220	0.0028	0.0356

Adj = adjusted; IU = international units; REML = restricted maximum likelihood

[a] Meta-regression analysis includes 27 studies with mean age 0-12 months and one study with mean age 2.7 years.
[b] Meta-regression analysis included 28 unique RCTs with total 53 intervention arms.

Three RCTs examining the effects of daily vitamin D intake on serum 25(OH)D concentration in children 0 to 4 years cannot be included in the meta-regression analysis due to insufficient quantitative data.[24,83,86] One RCT showed that 2,000 IU/d of vitamin D_2 and 2,000 IU/d of vitamin D_3 supplementation had similar effects on changes in 25(OH)D concentrations (both groups had about 150% increase from baseline; data were reported in the figure only).[83] Another RCT showed that neonates receiving 400 IU/d of vitamin D_3 had significantly higher median serum 25(OH)D levels after three months compared to placebo group (38.0 vs. 36.8 ng/mL; $P<0.001$), but there was no significant difference in 25(OH)D concentrations at six or 12 months.[24] Lastly, an RCT compared exclusively breastfed infant-mother dyads both receiving 400 IU/d of vitamin D_3 to exclusively breastfed infant-mother dyads with infants receiving placebo and mothers receiving 2,400 IU or 6,400 IU/d of vitamin D_3. Analysis revealed no difference in serum 25(OH)D between the groups.[86]

Figure KQ3-1a. Results of studies reporting the effect of daily vitamin D supplementation on serum 25(OH)D (nmol/L) by study arms in children 0-12 months. Legend: BF = breastfeeding; wk = weeks

Trial ID and Study	BF status	Arm	Vit D dose (IU/d)	25(OH)D assay	Follow-up, wk	n	Mean 25(OH)D, nmol/L (95% CI)
ID: 4							
Ala-Houhala et al. (1986)	exclusively BF	other: specify	0	other (specify)	0	17	23.35 (14.93, 31.77)
Ala-Houhala et al. (1986)	exclusively BF	other: specify	0	other (specify)	0	16	18.52 (12.21, 24.83)
Ala-Houhala et al. (1986)	exclusively BF	other: specify	0	other (specify)	8	17	52.87 (41.83, 63.92)
Ala-Houhala et al. (1986)	exclusively BF	other: specify	0	other (specify)	8	16	30.60 (25.86, 35.33)
Ala-Houhala et al. (1986)	exclusively BF	other: specify	0	other (specify)	15	16	41.33 (33.44, 49.22)
Ala-Houhala et al. (1986)	exclusively BF	other: specify	0	other (specify)	15	17	70.59 (55.86, 85.31)
Ala-Houhala et al. (1986)	exclusively BF	vit D2	400	other (specify)	0	16	19.86 (12.50, 27.23)
Ala-Houhala et al. (1986)	exclusively BF	vit D2	400	other (specify)	8	16	59.85 (43.54, 76.16)
Ala-Houhala et al. (1986)	exclusively BF	vit D2	400	other (specify)	15	16	82.66 (57.94, 107.39)
ID: 6							
Alonso et al. (2011)	any BF	no intervention	0	EIA/Chemiluminsecense	12	44	68.89 (59.45, 78.33)
Alonso et al. (2011)	any BF	no intervention	0	EIA/Chemiluminsecense	24	45	81.12 (74.63, 87.61)
Alonso et al. (2011)	any BF	no intervention	0	EIA/Chemiluminsecense	48	43	92.10 (81.96, 102.25)
Alonso et al. (2011)	any BF	vit D only	402	EIA/Chemiluminsecense	12	39	104.33 (91.25, 117.41)
Alonso et al. (2011)	any BF	vit D only	402	EIA/Chemiluminsecense	24	31	109.32 (97.20, 121.45)
Alonso et al. (2011)	any BF	vit D only	402	EIA/Chemiluminsecense	48	28	99.59 (90.72, 108.47)
ID: 8							
Atas et al. (2013)	exclusively BF	vit D only	200	HPLC	14	75	126.30 (115.79, 136.80)
Atas et al. (2013)	exclusively BF	vit D only	400	HPLC	14	64	184.20 (168.73, 199.68)
ID: 9							
Holmlund-Suila et al. (2012)	any BF	vit D3	1200	EIA/Chemiluminsecense	0	38	35.00 (30.23, 39.77)
Holmlund-Suila et al. (2012)	any BF	vit D3	1200	EIA/Chemiluminsecense	10	35	124.00 (114.06, 133.94)
Holmlund-Suila et al. (2012)	any BF	vit D3	1600	EIA/Chemiluminsecense	0	37	37.00 (32.81, 41.19)
Holmlund-Suila et al. (2012)	any BF	vit D3	1600	EIA/Chemiluminsecense	10	37	153.00 (140.11, 165.89)
Holmlund-Suila et al. (2012)	any BF	vit D3	400	EIA/Chemiluminsecense	0	37	35.00 (30.49, 39.51)
Holmlund-Suila et al. (2012)	any BF	vit D3	400	EIA/Chemiluminsecense	10	34	88.00 (81.95, 94.05)
ID: 13							
Chan et al. (1982)	any BF	no intervention	0	other (specify)	0	20	49.92 (35.24, 64.60)
Chan et al. (1982)	any BF	no intervention	0	other (specify)	6	22	42.43 (32.65, 52.22)
Chan et al. (1982)	any BF	no intervention	0	other (specify)	14	23	42.43 (27.76, 57.11)
Chan et al. (1982)	any BF	no intervention	0	other (specify)	22	19	47.42 (37.64, 57.21)
Chan et al. (1982)	any BF	vit D3	400	other (specify)	0	29	34.94 (30.05, 39.84)
Chan et al. (1982)	any BF	vit D3	400	other (specify)	6	23	47.42 (37.64, 57.21)
Chan et al. (1982)	any BF	vit D3	400	other (specify)	14	24	54.91 (40.24, 69.59)
Chan et al. (1982)	any BF	vit D3	400	other (specify)	22	20	57.41 (42.73, 72.08)
ID: 14							
Chandy et al. (2016)	exclusively BF	placebo	0	RIA kits	14	54	42.30 (34.67, 49.93)
Chandy et al. (2016)	exclusively BF	other: specify	0	RIA kits	14	51	60.90 (52.69, 69.11)
Chandy et al. (2016)	exclusively BF	vit D3	400	RIA kits	14	47	59.30 (51.87, 66.73)
ID: 15							
Dawodu et al. (2019)	exclusively BF	placebo	0	EIA/Chemiluminsecense	0	93	31.90 (27.49, 36.31)
Dawodu et al. (2019)	exclusively BF	placebo	0	EIA/Chemiluminsecense	12	67	81.40 (75.05, 87.75)
Dawodu et al. (2019)	exclusively BF	placebo	0	EIA/Chemiluminsecense	24	55	92.20 (82.82, 101.58)
Dawodu et al. (2019)	exclusively BF	vit D3	400	EIA/Chemiluminsecense	0	94	29.60 (26.18, 33.02)
Dawodu et al. (2019)	exclusively BF	vit D3	400	EIA/Chemiluminsecense	12	60	105.50 (92.75, 118.25)
Dawodu et al. (2019)	exclusively BF	vit D3	400	EIA/Chemiluminsecense	24	47	109.10 (96.72, 121.48)

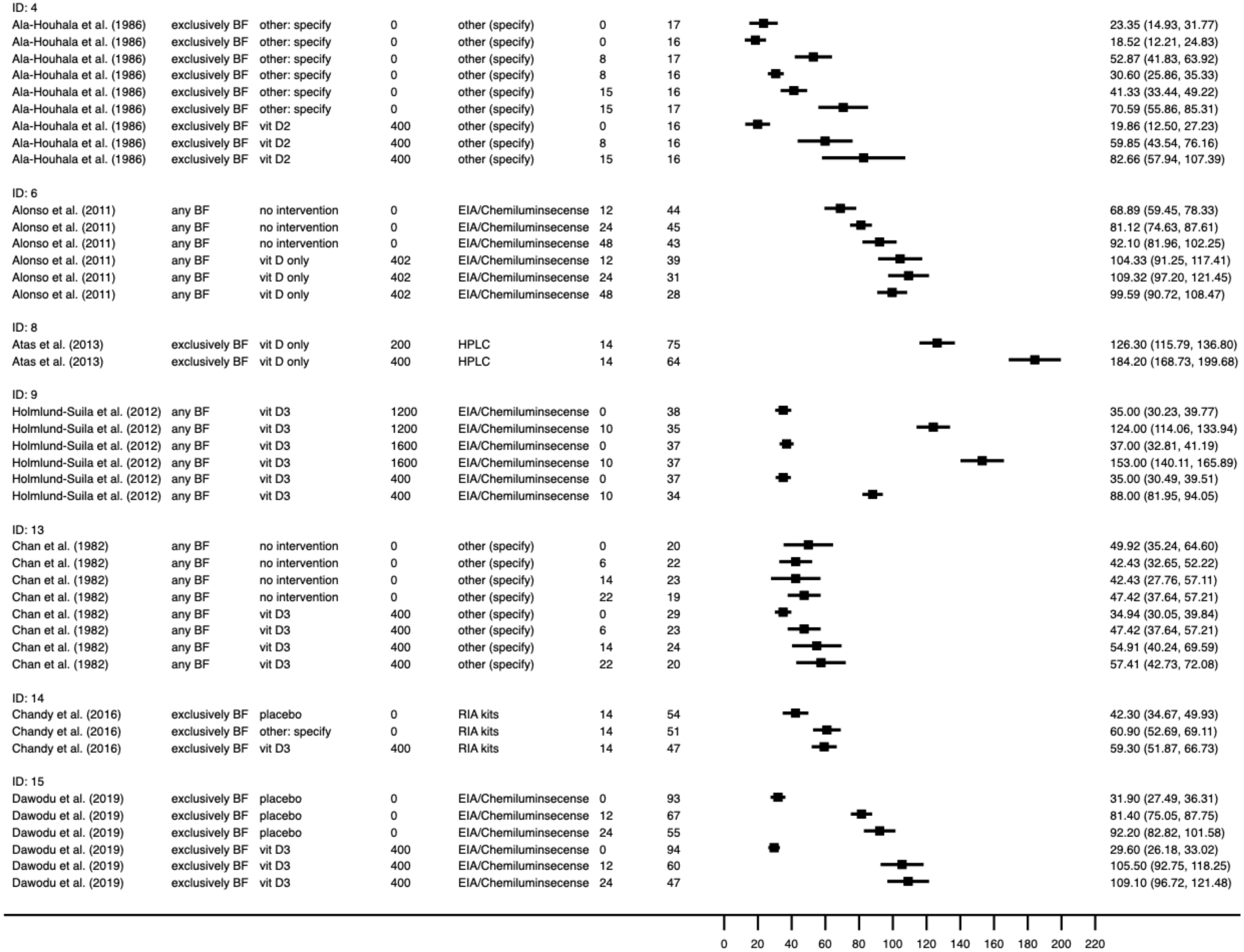

Figure KQ3-1a (continued). Results of studies reporting the effect of daily vitamin D supplementation on serum 25(OH)D (nmol/L) by study arms in children 0-12 months. Legend: BF = breastfeeding; wk = weeks

Trial ID and Study	BF status	Arm	Vit D dose (IU/d)	25(OH)D assay	Follow-up, wk	n	Mean 25(OH)D, nmol/L (95% CI)
ID: 18							
Enlund-Cerullo et al. (2019)	any BF	vit D3	1200	EIA/Chemiluminsecense	0	454	81.20 (79.01, 83.39)
Enlund-Cerullo et al. (2019)	any BF	vit D3	1200	EIA/Chemiluminsecense	48	407	114.40 (111.62, 117.18)
Enlund-Cerullo et al. (2019)	any BF	vit D3	400	EIA/Chemiluminsecense	0	459	81.40 (78.86, 83.94)
Enlund-Cerullo et al. (2019)	any BF	vit D3	400	EIA/Chemiluminsecense	48	409	82.70 (80.76, 84.64)
Rosendahl et al. (2018)	any BF	vit D3	1200	EIA/Chemiluminsecense	46	404	115.00 (112.30, 117.70)
Rosendahl et al. (2018)	any BF	vit D3	1200	EIA/Chemiluminsecense	94	410	117.70 (115.17, 120.23)
Rosendahl et al. (2018)	any BF	vit D3	400	EIA/Chemiluminsecense	46	401	82.70 (80.76, 84.64)
Rosendahl et al. (2018)	any BF	vit D3	400	EIA/Chemiluminsecense	94	404	86.60 (84.69, 88.51)
ID: 19							
Gallo et al. (2013)a	any BF	vit D2	400	EIA/Chemiluminsecense	0	25	56.90 (43.69, 70.11)
Gallo et al. (2013)a	any BF	vit D3	400	EIA/Chemiluminsecense	0	26	69.50 (57.74, 81.26)
Gallo et al. (2013)a	any BF	vit D3	400	LC-MS/MS	0	26	54.60 (45.49, 63.71)
Gallo et al. (2013)a	any BF	vit D2	400	LC-MS/MS	0	26	44.20 (35.05, 53.35)
Gallo et al. (2013)a	any BF	vit D2	400	EIA/Chemiluminsecense	12	24	69.00 (57.00, 81.00)
Gallo et al. (2013)a	any BF	vit D3	400	EIA/Chemiluminsecense	12	26	82.90 (74.02, 91.78)
Gallo et al. (2013)a	any BF	vit D3	400	LC-MS/MS	12	26	76.80 (70.11, 83.49)
Gallo et al. (2013)a	any BF	vit D2	400	LC-MS/MS	12	24	64.80 (54.32, 75.28)
ID: 20							
Gallo et al. (2013)b	any BF	vit D3	1200	LC-MS	4	29	64.64 (55.94, 73.33)
Gallo et al. (2013)b	any BF	vit D3	1200	LC-MS	8	31	112.17 (96.81, 127.54)
Gallo et al. (2013)b	any BF	vit D3	1200	LC-MS	12	27	134.00 (118.00, 150.00)
Gallo et al. (2013)b	any BF	vit D3	1200	LC-MS	24	30	119.13 (105.80, 132.46)
Gallo et al. (2013)b	any BF	vit D3	1200	LC-MS	36	27	109.28 (88.70, 129.86)
Gallo et al. (2013)b	any BF	vit D3	1600	LC-MS	4	11	63.64 (51.27, 76.00)
Gallo et al. (2013)b	any BF	vit D3	1600	LC-MS	8	13	120.85 (84.00, 157.70)
Gallo et al. (2013)b	any BF	vit D3	1600	LC-MS	12	13	180.00 (153.50, 206.50)
Gallo et al. (2013)b	any BF	vit D3	1600	LC-MS	24	10	160.12 (130.30, 189.94)
Gallo et al. (2013)b	any BF	vit D3	1600	LC-MS	36	13	122.79 (100.48, 145.09)
Gallo et al. (2013)b	any BF	vit D3	400	LC-MS	4	35	55.18 (48.67, 61.69)
Gallo et al. (2013)b	any BF	vit D3	400	LC-MS	8	25	66.27 (59.52, 73.01)
Gallo et al. (2013)b	any BF	vit D3	400	LC-MS	12	29	78.00 (71.50, 84.50)
Gallo et al. (2013)b	any BF	vit D3	400	LC-MS	24	29	81.69 (73.01, 90.36)
Gallo et al. (2013)b	any BF	vit D3	400	LC-MS	36	29	75.90 (69.40, 82.41)
Gallo et al. (2013)b	any BF	vit D3	800	LC-MS	4	30	52.84 (44.02, 61.67)
Gallo et al. (2013)b	any BF	vit D3	800	LC-MS	8	30	84.71 (74.41, 95.00)
Gallo et al. (2013)b	any BF	vit D3	800	LC-MS	12	32	102.00 (90.00, 114.00)
Gallo et al. (2013)b	any BF	vit D3	800	LC-MS	24	29	98.43 (86.42, 110.44)
Gallo et al. (2013)b	any BF	vit D3	800	LC-MS	36	29	87.65 (80.29, 95.00)
Wicklow et al. (2016)	any BF	vit D3	1200	LC-MS/MS	0	18	65.64 (54.11, 77.18)
Wicklow et al. (2016)	any BF	vit D3	1200	LC-MS/MS	8	18	135.53 (116.51, 154.56)
Wicklow et al. (2016)	any BF	vit D3	1200	LC-MS/MS	20	18	115.56 (96.89, 134.24)
Wicklow et al. (2016)	any BF	vit D3	400	LC-MS/MS	0	19	56.41 (47.21, 65.61)
Wicklow et al. (2016)	any BF	vit D3	400	LC-MS/MS	8	19	82.62 (74.20, 91.03)
Wicklow et al. (2016)	any BF	vit D3	400	LC-MS/MS	20	19	81.12 (69.67, 92.57)
Wicklow et al. (2016)	any BF	vit D3	800	LC-MS/MS	0	18	52.17 (41.67, 62.66)
Wicklow et al. (2016)	any BF	vit D3	800	LC-MS/MS	8	18	101.34 (87.96, 114.71)
Wicklow et al. (2016)	any BF	vit D3	800	LC-MS/MS	20	16	100.34 (87.74, 112.94)

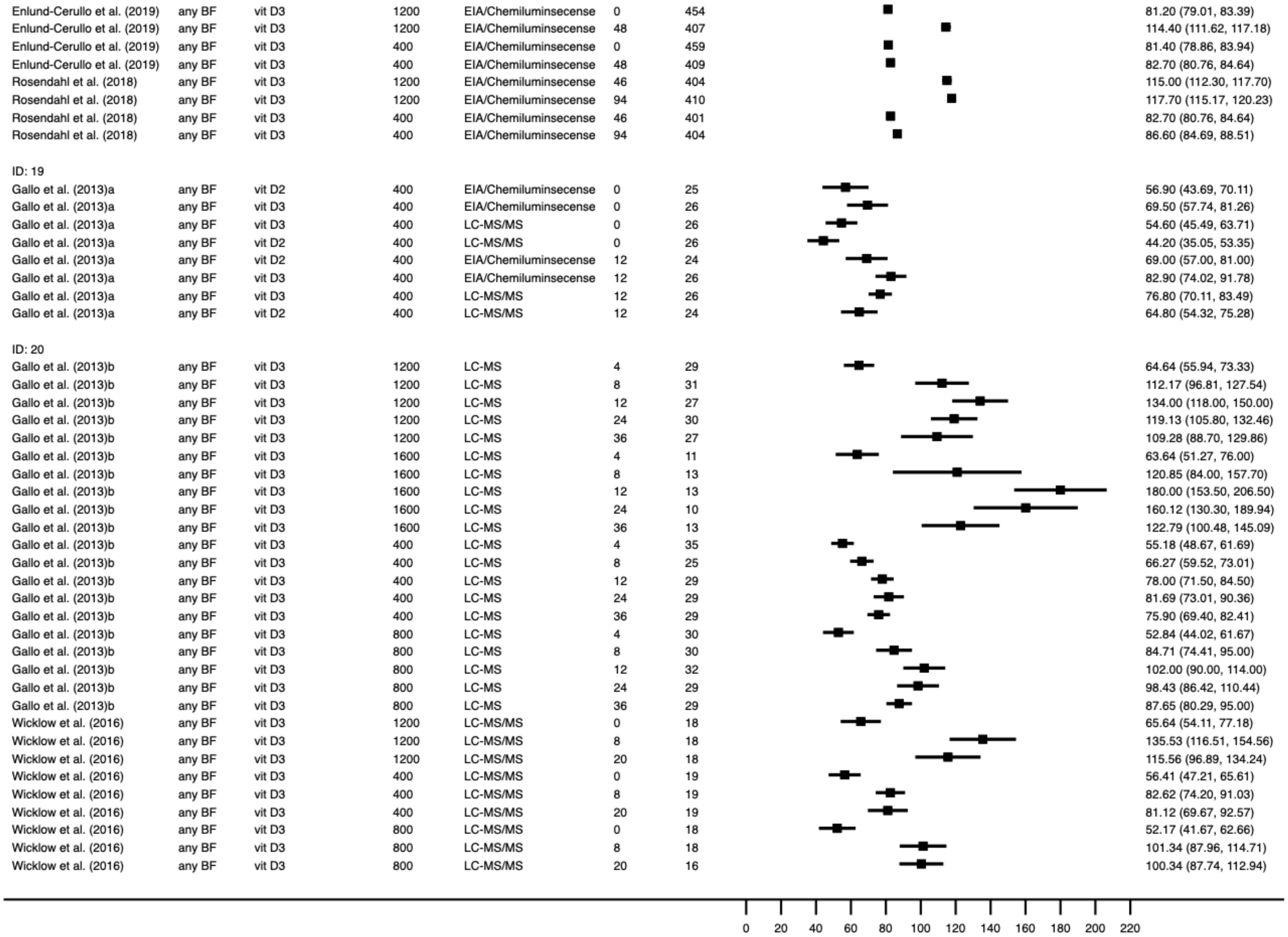

Figure KQ3-1a (continued). Results of studies reporting the effect of daily vitamin D supplementation on serum 25(OH)D (nmol/L) by study arms in children 0-12 months. Legend: BF = breastfeeding; wk = weeks

Trial ID and Study	BF status	Arm	Vit D dose (IU/d)	25(OH)D assay	Follow-up, wk	n	Mean 25(OH)D, nmol/L (95% CI)
ID: 22							
Gordon et al. (2008)	nr	vit D2	2000	EIA/Chemiluminsecense	0	12	39.31 (33.69, 44.93)
Gordon et al. (2008)	nr	vit D3	2000	EIA/Chemiluminsecense	0	14	38.06 (33.08, 43.05)
ID: 23							
Grant et al. (2014)	any BF	placebo	0	LC-MS/MS	0	63	37.19 (36.13, 38.24)
Grant et al. (2014)	any BF	placebo	0	LC-MS/MS	8	70	54.28 (51.98, 56.57)
Grant et al. (2014)	any BF	placebo	0	LC-MS/MS	16	68	70.61 (68.40, 72.82)
Grant et al. (2014)	any BF	placebo	0	LC-MS/MS	24	77	78.15 (76.46, 79.83)
Grant et al. (2014)	any BF	vit D3	400	LC-MS/MS	0	74	59.80 (59.12, 60.49)
Grant et al. (2014)	any BF	vit D3	400	LC-MS/MS	8	74	87.70 (86.21, 89.18)
Grant et al. (2014)	any BF	vit D3	400	LC-MS/MS	16	61	91.46 (90.27, 92.66)
Grant et al. (2014)	any BF	vit D3	400	LC-MS/MS	24	67	85.43 (84.47, 86.40)
Grant et al. (2014)	any BF	vit D3	800	LC-MS/MS	0	63	67.59 (66.60, 68.59)
Grant et al. (2014)	any BF	vit D3	800	LC-MS/MS	8	61	120.36 (118.28, 122.44)
Grant et al. (2014)	any BF	vit D3	800	LC-MS/MS	16	60	109.05 (107.21, 110.90)
Grant et al. (2014)	any BF	vit D3	800	LC-MS/MS	24	70	101.01 (99.72, 102.31)
ID: 27							
Greer et al. (1981)	exclusively BF	placebo	0	other (specify)	12	9	20.00 (18.04, 21.96)
Greer et al. (1981)	exclusively BF	vit D3	400	other (specify)	12	9	38.00 (36.04, 39.96)
ID: 30							
Hibbs et al. (2018)	any BF	vit D3	400	EIA/Chemiluminsecense	0	147	52.42 (50.00, 54.83)
Hibbs et al. (2018)	any BF	vit D3	400	EIA/Chemiluminsecense	0	153	52.24 (48.60, 55.88)
ID: 33							
Hollis et al. (2015)	exclusively BF	placebo	0	RIA kits	0	106	94.60 (83.53, 105.67)
Hollis et al. (2015)	exclusively BF	vit D3	400	RIA kits	0	110	84.12 (73.15, 95.08)
ID: 35							
Holstgemeiner et al. (1978)	any BF	vit D3	1200	nr	0	10	30.70 (26.70, 34.70)
Holstgemeiner et al. (1978)	any BF	vit D3	1200	nr	5	8	153.19 (107.80, 198.59)
ID: 36							
Huynh et al. (2017)	any BF	vit D3	400	EIA/Chemiluminsecense	14	23	81.29 (73.53, 89.05)
ID: 40							
Kunz et al. (1982)	any BF	vit D3	1000	nr	6	16	134.78 (112.77, 156.80)
Kunz et al. (1982)	any BF	vit D3	500	nr	6	13	92.35 (81.50, 103.21)
ID: 45							
Madar et al. (2009)	any BF	no intervention	0	HPLC	0	29	55.80 (42.77, 68.83)
Madar et al. (2009)	any BF	no intervention	0	HPLC	7	29	72.70 (60.29, 85.11)
Madar et al. (2009)	any BF	vit D2	400	HPLC	0	22	38.80 (24.26, 53.34)
Madar et al. (2009)	any BF	vit D2	400	HPLC	7	22	93.50 (80.63, 106.37)

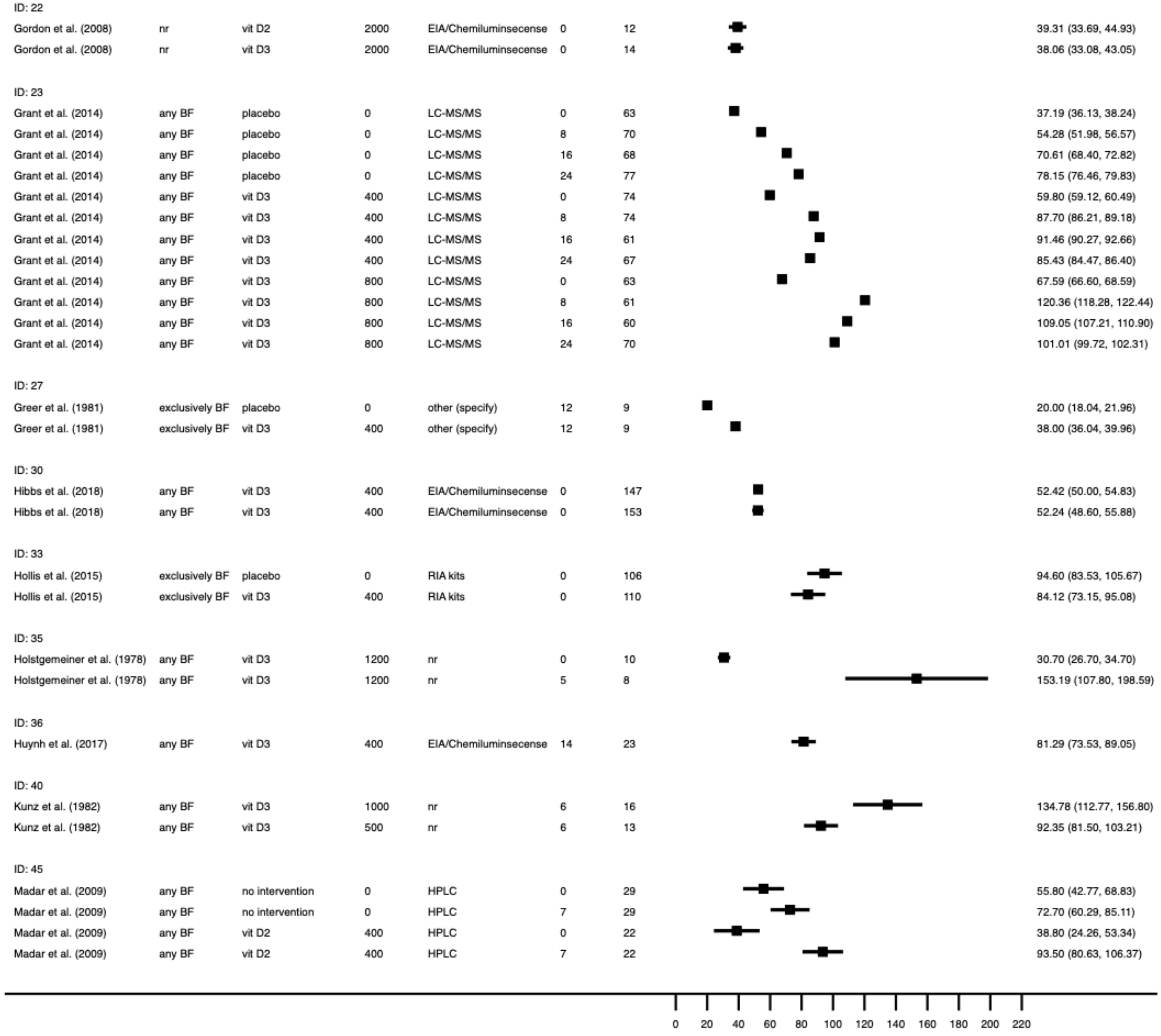

Figure KQ3-1a (continued). Results of studies reporting the effect of daily vitamin D supplementation on serum 25(OH)D (nmol/L) by study arms in children 0-12 months. Legend: BF = breastfeeding; wk = weeks

Trial ID and Study	BF status	Arm	Vit D dose (IU/d)	25(OH)D assay	Follow-up, wk	n	Mean 25(OH)D, nmol/L (95% CI)
ID: 52							
Natarajan et al. (2014)	any BF	vit D3	400	EIA/Chemiluminsecense	7.5	45	49.00 (42.52, 55.47)
Natarajan et al. (2014)	any BF	vit D3	800	EIA/Chemiluminsecense	7.6	43	93.48 (77.67, 109.28)
ID: 54							
Pittard (term infants only) et al. (1991)	exclusively formula	vit D only	400	other (specify)	2	11	57.41 (45.61, 69.21)
Pittard (term infants only) et al. (1991)	exclusively formula	vit D only	400	other (specify)	4	11	52.42 (40.62, 64.22)
Pittard (term infants only) et al. (1991)	exclusively formula	vit D only	400	other (specify)	6	11	62.40 (47.65, 77.15)
Pittard (term infants only) et al. (1991)	exclusively formula	vit D only	400	other (specify)	8	11	69.89 (50.71, 89.06)
Pittard (term infants only) et al. (1991)	exclusively formula	vit D only	400	other (specify)	10	11	67.39 (54.12, 80.67)
Pittard (term infants only) et al. (1991)	exclusively formula	vit D only	400	other (specify)	12	11	67.39 (54.12, 80.67)
Pittard (term infants only) et al. (1991)	exclusively formula	vit D only	400	other (specify)	14	11	72.38 (53.21, 91.56)
Pittard (term infants only) et al. (1991)	exclusively formula	vit D only	400	other (specify)	16	11	64.90 (47.20, 82.60)
Pittard (term infants only) et al. (1991)	exclusively formula	vit D only	800	other (specify)	2	5	52.42 (41.48, 63.36)
Pittard (term infants only) et al. (1991)	exclusively formula	vit D only	800	other (specify)	4	5	64.90 (47.39, 82.40)
Pittard (term infants only) et al. (1991)	exclusively formula	vit D only	800	other (specify)	6	5	69.89 (56.76, 83.01)
Pittard (term infants only) et al. (1991)	exclusively formula	vit D only	800	other (specify)	8	5	69.89 (50.20, 89.58)
Pittard (term infants only) et al. (1991)	exclusively formula	vit D only	800	other (specify)	10	5	82.37 (53.93, 110.81)
Pittard (term infants only) et al. (1991)	exclusively formula	vit D only	800	other (specify)	12	5	84.86 (49.86, 119.87)
Pittard (term infants only) et al. (1991)	exclusively formula	vit D only	800	other (specify)	14	5	82.37 (47.36, 117.37)
Pittard (term infants only) et al. (1991)	exclusively formula	vit D only	800	other (specify)	16	5	87.36 (56.73, 117.99)
ID: 55							
Ponnapakkam et al. (2010)	any BF	vit D3	.	EIA/Chemiluminsecense	8	8	88.75 (37.77, 139.73)
Ponnapakkam et al. (2010)	any BF	vit D3	.	EIA/Chemiluminsecense	16	7	132.86 (87.87, 177.84)
Ponnapakkam et al. (2010)	any BF	placebo	0	EIA/Chemiluminsecense	8	6	44.00 (27.55, 60.45)
Ponnapakkam et al. (2010)	any BF	placebo	0	EIA/Chemiluminsecense	16	6	57.83 (43.74, 71.93)
Ponnapakkam et al. (2010)	any BF	placebo	0	EIA/Chemiluminsecense	24	8	108.00 (69.51, 146.49)
Ponnapakkam et al. (2010)	any BF	vit D3	200	EIA/Chemiluminsecense	8	6	83.00 (36.92, 129.08)
Ponnapakkam et al. (2010)	any BF	vit D3	200	EIA/Chemiluminsecense	16	6	122.33 (53.04, 191.62)
Ponnapakkam et al. (2010)	any BF	vit D3	200	EIA/Chemiluminsecense	24	8	99.86 (73.55, 126.16)
ID: 59							
Rueter et al. (2019)	nr	placebo	0	EIA/Chemiluminsecense	12	90	59.20 (54.51, 63.89)
Rueter et al. (2019)	nr	placebo	0	EIA/Chemiluminsecense	24	87	82.00 (76.14, 87.86)
Rueter et al. (2019)	nr	vit D3	400	EIA/Chemiluminsecense	12	90	83.20 (77.46, 88.94)
Rueter et al. (2019)	nr	vit D3	400	EIA/Chemiluminsecense	24	86	93.10 (87.03, 99.17)

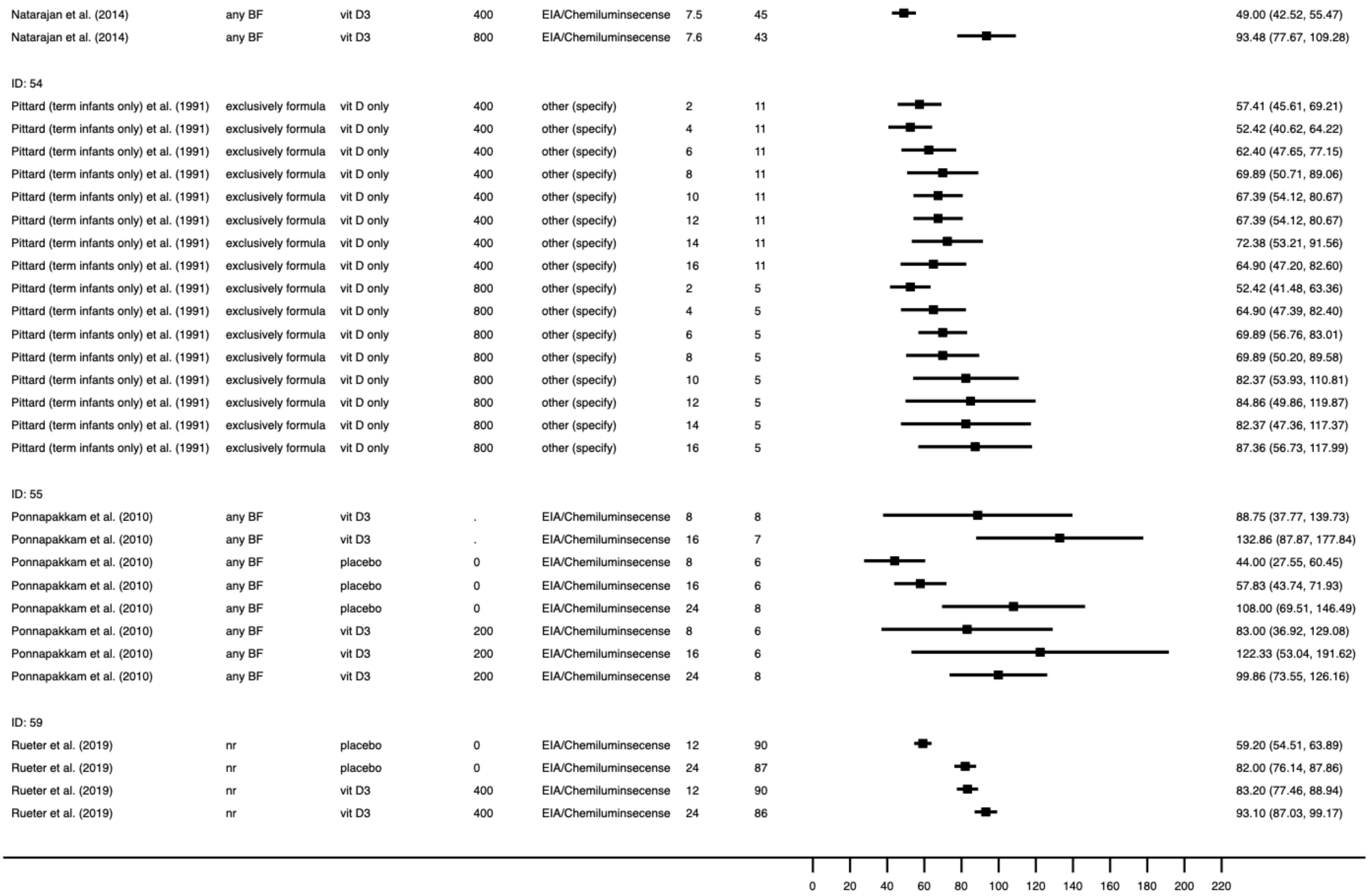

Figure KQ3-1a (continued). Results of studies reporting the effect of daily vitamin D supplementation on serum 25(OH)D (nmol/L) by study arms in children 0-12 months. Legend: BF = breastfeeding; wk = weeks

Trial ID and Study	BF status	Arm	Vit D dose (IU/d)	25(OH)D assay	Follow-up, wk	n	Mean 25(OH)D, nmol/L (95% CI)
ID: 61							
Shakiba et al. (2010)	any BF	vit D3	200	EIA/Chemiluminsecense	24	19	78.12 (68.59, 87.66)
Shakiba et al. (2010)	any BF	vit D3	400	EIA/Chemiluminsecense	24	26	95.85 (84.91, 106.78)
ID: 63							
Sharma et al. (2016)	any BF	vit D3	1200	LC-MS/MS	0	29	64.65 (56.71, 72.58)
Sharma et al. (2016)	any BF	vit D3	1200	LC-MS/MS	4	30	120.24 (106.00, 134.49)
Sharma et al. (2016)	any BF	vit D3	1200	LC-MS/MS	8	27	130.85 (114.56, 147.14)
Sharma et al. (2016)	any BF	vit D3	1200	LC-MS/MS	20	30	125.42 (112.69, 138.16)
Sharma et al. (2016)	any BF	vit D3	1200	LC-MS/MS	32	27	139.96 (115.37, 164.55)
Sharma et al. (2016)	any BF	vit D3	1200	LC-MS/MS	44	28	90.04 (82.71, 97.37)
Sharma et al. (2016)	any BF	vit D3	1600	LC-MS/MS	0	11	62.15 (48.73, 75.57)
Sharma et al. (2016)	any BF	vit D3	1600	LC-MS/MS	4	13	122.30 (84.35, 160.26)
Sharma et al. (2016)	any BF	vit D3	1600	LC-MS/MS	8	13	179.65 (156.66, 202.64)
Sharma et al. (2016)	any BF	vit D3	1600	LC-MS/MS	20	10	165.92 (142.07, 189.78)
Sharma et al. (2016)	any BF	vit D3	1600	LC-MS/MS	32	12	124.80 (103.47, 146.13)
Sharma et al. (2016)	any BF	vit D3	1600	LC-MS/MS	44	12	98.53 (81.90, 115.16)
Sharma et al. (2016)	any BF	vit D3	400	LC-MS/MS	0	35	57.72 (51.84, 63.60)
Sharma et al. (2016)	any BF	vit D3	400	LC-MS/MS	4	24	62.90 (55.72, 70.08)
Sharma et al. (2016)	any BF	vit D3	400	LC-MS/MS	8	29	75.88 (69.02, 82.74)
Sharma et al. (2016)	any BF	vit D3	400	LC-MS/MS	20	29	75.32 (64.18, 86.46)
Sharma et al. (2016)	any BF	vit D3	400	LC-MS/MS	32	29	72.20 (65.49, 78.90)
Sharma et al. (2016)	any BF	vit D3	400	LC-MS/MS	44	29	71.88 (67.67, 76.10)
Sharma et al. (2016)	any BF	vit D3	800	LC-MS/MS	0	29	56.35 (47.90, 64.80)
Sharma et al. (2016)	any BF	vit D3	800	LC-MS/MS	4	30	87.36 (77.38, 97.34)
Sharma et al. (2016)	any BF	vit D3	800	LC-MS/MS	8	32	105.27 (93.36, 117.17)
Sharma et al. (2016)	any BF	vit D3	800	LC-MS/MS	20	30	103.02 (92.32, 113.73)
Sharma et al. (2016)	any BF	vit D3	800	LC-MS/MS	32	28	83.87 (76.51, 91.22)
Sharma et al. (2016)	any BF	vit D3	800	LC-MS/MS	44	27	80.00 (72.92, 87.07)
ID: 64							
Siafarikas et al. (2011)	any BF	vit D3	250	RIA kits	0	14	68.00 (53.00, 83.00)
Siafarikas et al. (2011)	any BF	vit D3	250	RIA kits	6	14	139.00 (114.00, 164.00)
Siafarikas et al. (2011)	any BF	vit D3	500	RIA kits	0	14	68.00 (53.00, 83.00)
Siafarikas et al. (2011)	any BF	vit D3	500	RIA kits	6	14	151.00 (126.00, 176.00)
ID: 65							
Singh et al. (2018)	exclusively BF	no intervention	0	EIA/Chemiluminsecense	24	48	67.14 (55.70, 78.58)
Singh et al. (2018)	exclusively BF	vit D3	400	EIA/Chemiluminsecense	24	49	75.38 (62.59, 88.17)

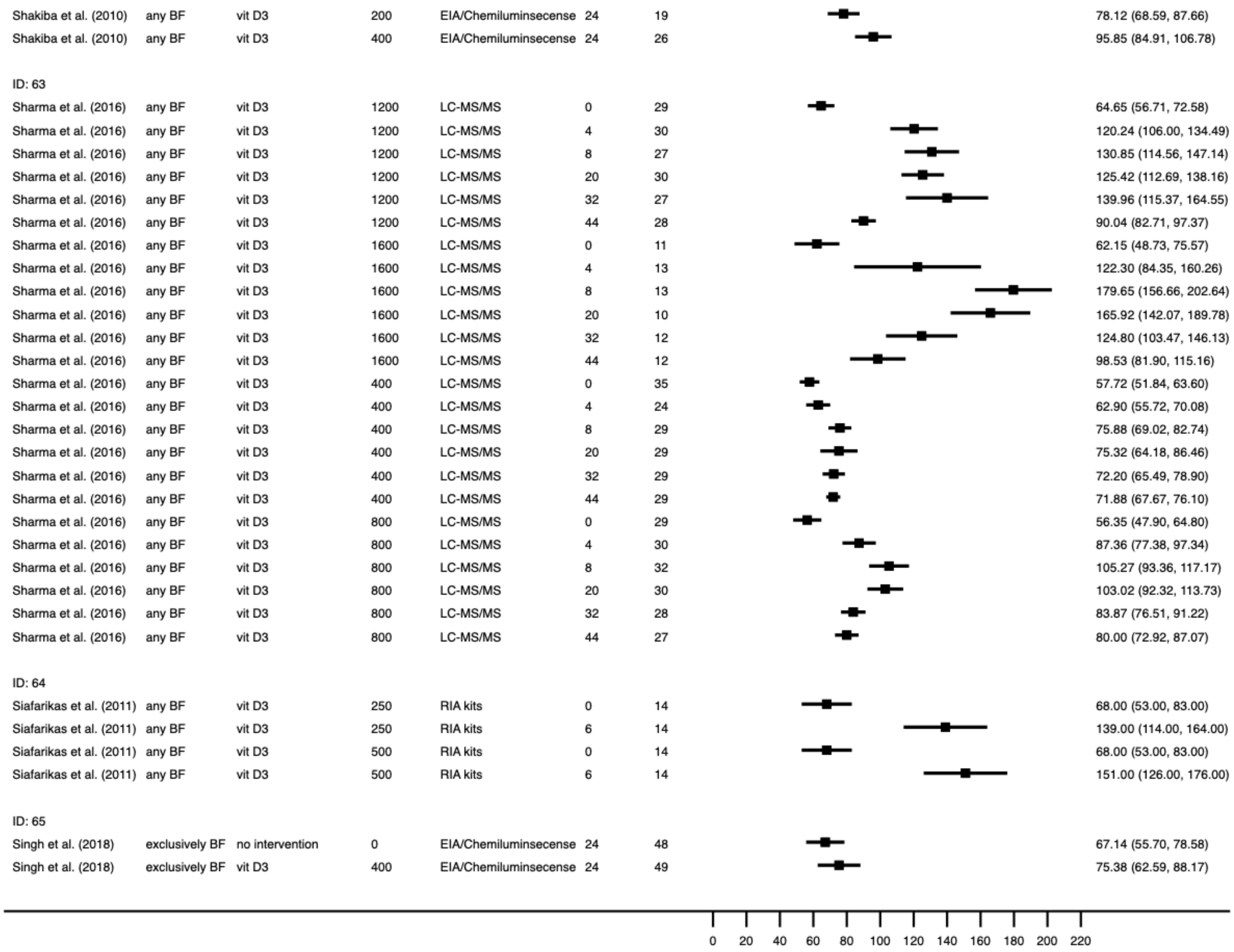

Figure KQ3-1a (continued). Results of studies reporting the effect of daily vitamin D supplementation on serum 25(OH)D (nmol/L) by study arms in children 0-12 months. Legend: BF = breastfeeding; wk = weeks

Trial ID and Study	BF status	Arm	Vit D dose (IU/d)	25(OH)D assay	Follow-up, wk	n	Mean 25(OH)D, nmol/L (95% CI)
ID: 77							
Zeghoud et al. (Hypovitaminosis group 16-30 nmol/L)	nr	vit D2	1000	RIA kits	0	11	21.82 (18.67, 24.96)
Zeghoud et al. (Hypovitaminosis group 16-30 nmol/L)	nr	vit D2	1000	RIA kits	4	11	59.80 (49.34, 70.26)
Zeghoud et al. (Hypovitaminosis group 16-30 nmol/L)	nr	vit D2	1000	RIA kits	12	11	69.63 (58.31, 80.95)
Zeghoud et al. (Hypovitaminosis group 16-30 nmol/L)	nr	vit D2	500	RIA kits	0	9	22.14 (18.99, 25.28)
Zeghoud et al. (Hypovitaminosis group 16-30 nmol/L)	nr	vit D2	500	RIA kits	4	9	58.72 (52.43, 65.01)
Zeghoud et al. (Hypovitaminosis group 16-30 nmol/L)	nr	vit D2	500	RIA kits	12	9	65.45 (54.76, 76.15)
Zeghoud et al. (Vit D deficient group <=15 nmol/L)	nr	vit D2	1000	RIA kits	0	4	17.97 (12.94, 23.00)
Zeghoud et al. (Vit D deficient group <=15 nmol/L)	nr	vit D2	1000	RIA kits	4	4	51.30 (39.54, 63.06)
Zeghoud et al. (Vit D deficient group <=15 nmol/L)	nr	vit D2	1000	RIA kits	12	4	70.27 (61.46, 79.07)
Zeghoud et al. (Vit D deficient group <=15 nmol/L)	nr	vit D2	500	RIA kits	0	8	18.29 (14.52, 22.06)
Zeghoud et al. (Vit D deficient group <=15 nmol/L)	nr	vit D2	500	RIA kits	4	8	45.50 (40.37, 50.63)
Zeghoud et al. (Vit D deficient group <=15 nmol/L)	nr	vit D2	500	RIA kits	12	8	55.83 (50.17, 61.49)
Zeghoud et al. (Vit D sufficient group >30 nmol/L)	nr	vit D2	1000	RIA kits	0	13	46.84 (43.07, 50.62)
Zeghoud et al. (Vit D sufficient group >30 nmol/L)	nr	vit D2	1000	RIA kits	4	13	59.20 (53.00, 65.40)
Zeghoud et al. (Vit D sufficient group >30 nmol/L)	nr	vit D2	1000	RIA kits	12	13	69.30 (61.13, 77.48)
Zeghoud et al. (Vit D sufficient group >30 nmol/L)	nr	vit D2	500	RIA kits	0	7	47.81 (42.78, 52.84)
Zeghoud et al. (Vit D sufficient group >30 nmol/L)	nr	vit D2	500	RIA kits	4	7	59.68 (54.65, 64.71)
Zeghoud et al. (Vit D sufficient group >30 nmol/L)	nr	vit D2	500	RIA kits	12	7	62.57 (57.54, 67.60)
ID: 79							
Zhou et al. (2018)	any BF	vit D3	1200	nr	0	164	42.60 (41.70, 43.50)
Zhou et al. (2018)	any BF	vit D3	1200	nr	6	164	61.30 (59.82, 62.78)
Zhou et al. (2018)	any BF	vit D3	1200	nr	16	164	62.80 (61.24, 64.36)
Zhou et al. (2018)	any BF	vit D3	400	nr	0	168	43.40 (42.48, 44.32)
Zhou et al. (2018)	any BF	vit D3	400	nr	6	168	43.80 (42.89, 44.71)
Zhou et al. (2018)	any BF	vit D3	400	nr	16	168	43.10 (42.13, 44.07)
ID: 80							
Ziegler et al. (2014)	exclusively BF	vit D3	200	RIA kits	0	56	35.10 (30.18, 40.02)
Ziegler et al. (2014)	exclusively BF	vit D3	200	RIA kits	8	53	64.30 (58.65, 69.95)
Ziegler et al. (2014)	exclusively BF	vit D3	200	RIA kits	16	46	76.10 (67.95, 84.25)
Ziegler et al. (2014)	exclusively BF	vit D3	200	RIA kits	22	45	75.10 (66.31, 83.89)
Ziegler et al. (2014)	exclusively BF	vit D3	200	RIA kits	30	42	85.70 (78.47, 92.93)
Ziegler et al. (2014)	exclusively BF	vit D3	400	RIA kits	0	60	42.20 (37.49, 46.91)
Ziegler et al. (2014)	exclusively BF	vit D3	400	RIA kits	8	55	72.20 (67.05, 77.35)
Ziegler et al. (2014)	exclusively BF	vit D3	400	RIA kits	16	44	81.30 (72.67, 89.93)
Ziegler et al. (2014)	exclusively BF	vit D3	400	RIA kits	22	37	84.50 (74.87, 94.13)
Ziegler et al. (2014)	exclusively BF	vit D3	400	RIA kits	30	34	94.30 (84.25, 104.35)
Ziegler et al. (2014)	exclusively BF	vit D3	600	RIA kits	0	56	43.40 (37.90, 48.90)
Ziegler et al. (2014)	exclusively BF	vit D3	600	RIA kits	8	49	77.30 (71.25, 83.35)
Ziegler et al. (2014)	exclusively BF	vit D3	600	RIA kits	16	42	101.60 (91.80, 111.40)
Ziegler et al. (2014)	exclusively BF	vit D3	600	RIA kits	22	40	97.60 (89.14, 106.06)
Ziegler et al. (2014)	exclusively BF	vit D3	600	RIA kits	30	32	101.60 (90.17, 113.03)
Ziegler et al. (2014)	exclusively BF	vit D3	800	RIA kits	0	41	44.20 (38.14, 50.26)
Ziegler et al. (2014)	exclusively BF	vit D3	800	RIA kits	8	37	82.70 (72.81, 92.59)
Ziegler et al. (2014)	exclusively BF	vit D3	800	RIA kits	16	33	100.90 (91.62, 110.18)
Ziegler et al. (2014)	exclusively BF	vit D3	800	RIA kits	22	30	103.00 (94.56, 111.44)
Ziegler et al. (2014)	exclusively BF	vit D3	800	RIA kits	30	28	115.80 (106.61, 124.99)

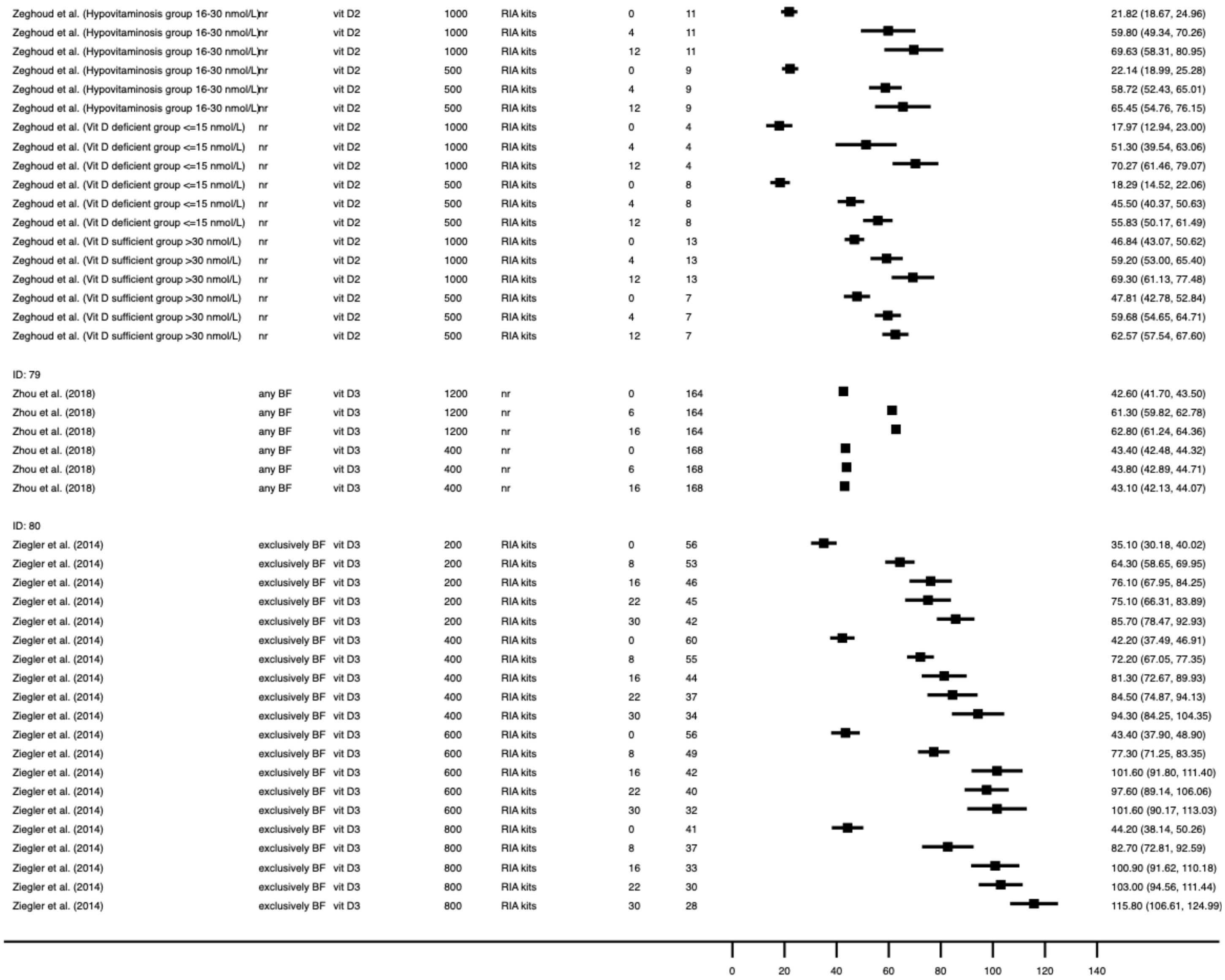

Figure KQ3-1b. Results of studies reporting the effect of daily vitamin D supplementation on serum 25(OH)D (nmol/L) by study arms in children 1 to 4 years. Legend: BF = breastfeeding; wk = weeks

Trial ID and Study	BF status	Arm	Vit D dose (IU/d)	25(OH)D assay	Follow-up, wk	n	Mean 25(OH)D, nmol/L (95% CI)
ID: 2							
Aglipay et al. (2017)	any BF	vit D3	2000	other (specify)	0	349	89.61 (86.39, 92.83)
Aglipay et al. (2017)	any BF	vit D3	2000	other (specify)	16	349	121.56 (117.06, 126.05)
Aglipay et al. (2017)	any BF	vit D3	400	other (specify)	0	354	92.10 (89.06, 95.14)
Aglipay et al. (2017)	any BF	vit D3	400	other (specify)	16	354	91.85 (88.36, 95.35)

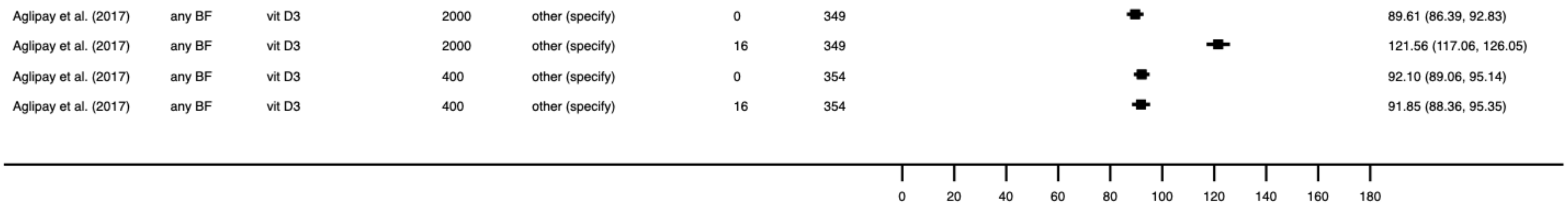

Figure KQ3-2. Summary (panel a) and individual study (panel b) ROB for studies reporting the effect of vitamin D on serum 25(OH)D in children ages 0-12 months

a.

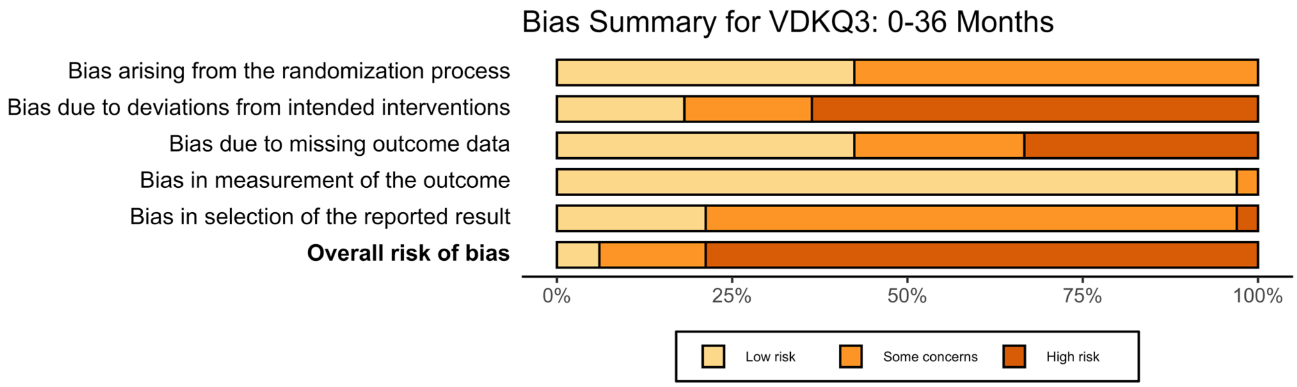

b.

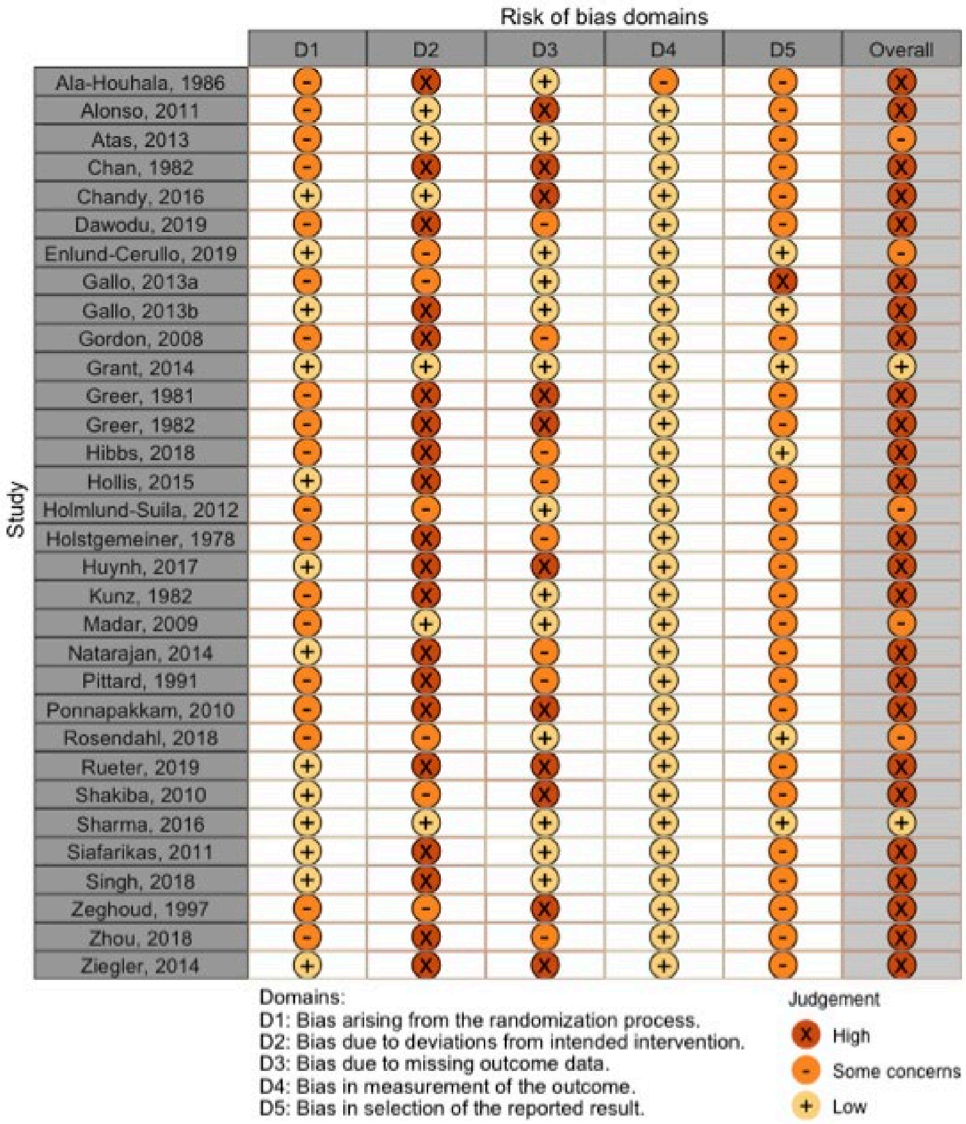

Figure KQ3-3. Random-effects meta-regression analysis to examine the association between daily vitamin D supplementation doses and achieved 25(OH)D concentrations at the end of intervention period in children 0 to 4 years

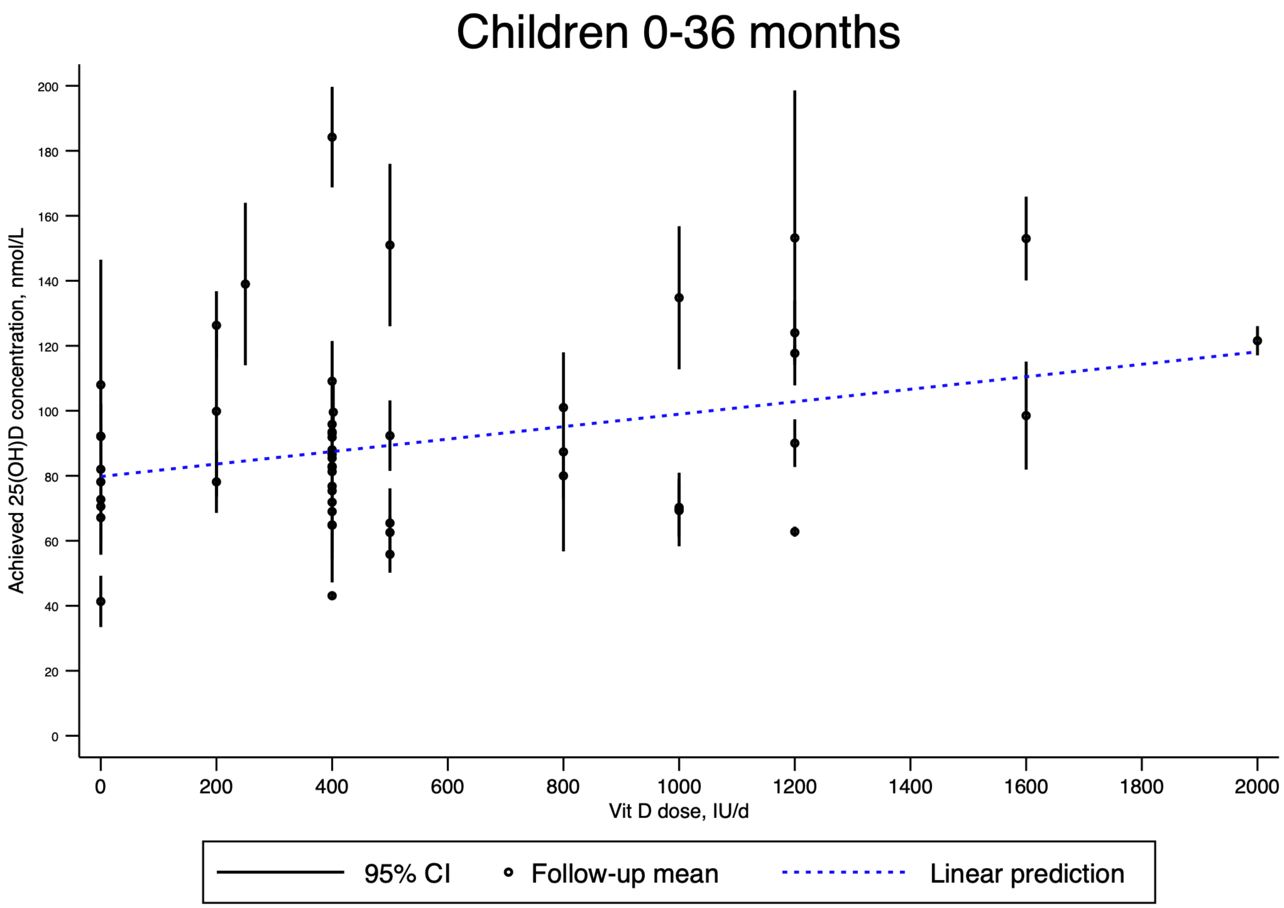

Children 3-9 years old

Seven unique studies (all RCTs) examining the effects of daily vitamin D intake on serum 25(OH)D concentration in children three to nine years old were included.[101-103,109-111,114] The individual study results are shown in **Figure KQ3-4**. The doses of vitamin D supplementation (mostly vitamin D_3) ranged from 400 to 2,000 IU/d across the seven RCTs, and of these, five RCTs included a non-vitamin D supplementation comparison group (placebo, no intervention, or maternal supplementation). Intervention durations ranged from eight to 52 weeks. Sample sizes ranged widely from 24 to 199 subjects per intervention arm.

Figure KQ3-5 shows the summary and individual ROB plots for trials reporting the effect of vitamin D intake on serum 25(OH)D concentration in children ages three to nine years. More than 75% of trials had some or high ROB due to deviations from intended interventions, often due to issues of non-adherence and how non-adherence was accounted for in the statistical methods. All (100%) of the trials also had some ROB due to selection of the reported results. More than 50% of trials had some or high ROB in the randomization process, and more than 25% had some or high ROB due to missing outcome data. All studies (100%) had low ROB in measurement of the outcome.

Table KQ3-3 shows the ROB for one cluster-randomized trial reporting the effect of vitamin D intake on serum 25(OH)D in children ages 3-9 years. The study had high ROB due to the randomization process and low ROB in all other ROB domains.

Random-effects meta-regression showed that each 100 IU/d increase in vit D supplementation was associated with an average of 2.49 (95% CI -0.24, 5.22) nmol/L increase in achieved 25(OH)D concentration in children three to nine years old (n = 16 intervention arms; $P = 0.071$; adjusted R^2 = 19.96%). However, the residual heterogeneity is large (I^2 = 97.75%) (**Table KQ3-4**; **Figure KQ3-6**).

One RCT comparing the effects of three daily vitamin D_3 supplementation doses (600, 1,000, and 2,000 IU/d) on serum 25(OH)D concentrations in pre-pubertal girls aged 6.1 to 11.8 years old cannot be included in the meta-regression.[110] The study found that the median serum 25(OH)D increased by 37.25, 45.0, and 55.5 nmol/L (14.9, 18.0, and 22.2 ng/mL), respectively, for the three supplementation groups, and analysis of variance revealed these changes were significantly different ($P<0.05$).

Table KQ3-3. ROB for one cluster-randomized trial reporting the effect of vitamin D intake on serum 25(OH)D in children ages 3-9 years[a]

Author (year)	Bias arising from the randomization process	Bias arising from the timing of identification and recruitment of participants	Bias due to deviations from intended interventions	Bias due to missing data	Bias in measurement of the outcome	Bias due to selection of the reported result
Mandlik et al. (2020)[115]	High	Low	Low	Low	Low	Low

[a] Assessment based on *Revised Cochrane risk of bias tool for randomized trials (RoB 2.0): Additional considerations for cluster-randomized trials.*

Figure KQ3-4. Results of studies reporting the effect of daily vitamin D supplementation on serum 25(OH)D (nmol/L) in children 3-9 years old. Legend: BF = breastfeeding; wk = weeks

Study	BF status	Arm	Vit D dose (IU/d)	25(OH)D assay	Follow-up, wk	n	Mean 25(OH)D, nmol/L (95% CI)
ID: 1							
Abrams et al. (2013)	n/a	placebo	0	EIA/Chemiluminescence	0	31	68.89 (62.48, 75.30)
Abrams et al. (2013)	n/a	placebo	0	EIA/Chemiluminescence	8	31	74.63 (63.74, 85.53)
Abrams et al. (2013)	n/a	vit D3	1000	EIA/Chemiluminescence	0	32	69.14 (62.74, 75.54)
Abrams et al. (2013)	n/a	vit D3	1000	EIA/Chemiluminescence	8	32	89.86 (80.95, 98.76)
ID: 5							
Alahouhala et al. (1988)	n/a	placebo	0	HPLC	0	27	45.93 (40.09, 51.76)
Alahouhala et al. (1988)	n/a	placebo	0	HPLC	32	27	58.91 (52.22, 65.59)
Alahouhala et al. (1988)	n/a	placebo	0	HPLC	52	27	43.18 (35.84, 50.52)
Alahouhala et al. (1988)	n/a	vit D2	400	HPLC	0	24	49.17 (41.58, 56.76)
Alahouhala et al. (1988)	n/a	vit D2	400	HPLC	32	24	77.88 (68.19, 87.56)
Alahouhala et al. (1988)	n/a	vit D2	400	HPLC	52	24	71.14 (61.65, 80.62)
ID: 11							
Brett et al. (2016)	nr	no intervention	0	EIA/Chemiluminsecense	12	24	55.80 (50.88, 60.72)
Brett et al. (2016)	nr	vit D3	400	EIA/Chemiluminsecense	12	25	64.10 (60.18, 68.02)
Brett et al. (2016)	nr	vit D3	600	EIA/Chemiluminsecense	12	25	63.70 (58.84, 68.56)
ID: 47							
Mandlik et al. (2019)	nr	placebo	0	EIA/Chemiluminsecense	0	150	57.70 (56.10, 59.30)
Mandlik et al. (2019)	nr	placebo	0	EIA/Chemiluminsecense	24	145	58.30 (55.74, 60.86)
Mandlik et al. (2019)	nr	placebo	0	EIA/Chemiluminsecense	48	49	42.60 (35.46, 49.74)
Mandlik et al. (2019)	nr	vit D plus calcium	1000	EIA/Chemiluminsecense	0	135	60.20 (58.19, 62.21)
Mandlik et al. (2019)	nr	vit D plus calcium	1000	EIA/Chemiluminsecense	24	124	83.90 (78.60, 89.20)
Mandlik et al. (2019)	nr	vit D plus calcium	1000	EIA/Chemiluminsecense	48	79	54.40 (48.62, 60.18)
ID: 49							
Marwaha et al. (2018)	n/a	vit D3	600	EIA/Chemiluminsecense	0	74	25.28 (23.29, 27.28)
Marwaha et al. (2018)	n/a	vit D3	1000	EIA/Chemiluminsecense	0	67	25.48 (23.27, 27.70)
Marwaha et al. (2018)	n/a	vit D3	2000	EIA/Chemiluminsecense	0	75	24.46 (22.35, 26.57)
ID: 51							
Mortensen et al. (2016)	nr	placebo	0	LC-MS/MS	0	41	55.20 (51.89, 58.51)
Mortensen et al. (2016)	nr	placebo	0	LC-MS/MS	20	41	31.10 (28.80, 33.40)
Mortensen et al. (2016)	nr	vit D3	400	LC-MS/MS	0	38	56.90 (52.86, 60.94)
Mortensen et al. (2016)	nr	vit D3	400	LC-MS/MS	20	38	61.80 (58.43, 65.17)
Mortensen et al. (2016)	nr	vit D3	800	LC-MS/MS	0	40	58.10 (53.92, 62.28)
Mortensen et al. (2016)	nr	vit D3	800	LC-MS/MS	20	40	75.80 (72.24, 79.36)
ID: 69							
Talaat et al. (2016)	n/a	vit D3	400	EIA/Chemiluminsecense	0	196	54.09 (50.76, 57.42)
Talaat et al. (2016)	n/a	vit D3	400	EIA/Chemiluminsecense	16	196	42.28 (39.00, 45.57)
Talaat et al. (2016)	n/a	vit D3	400	EIA/Chemiluminsecense	48	196	25.56 (22.67, 28.45)

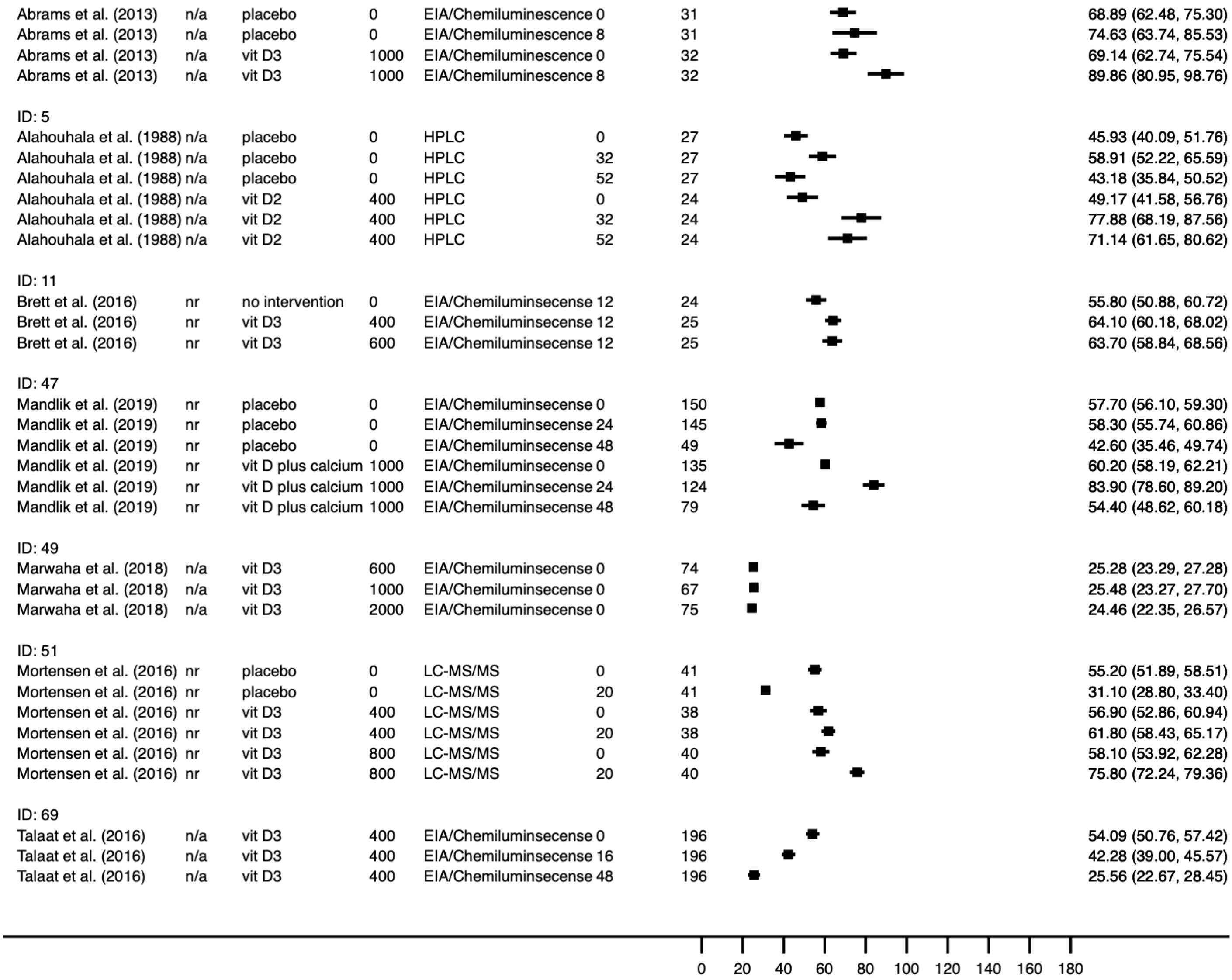

Figure KQ3-5. Summary (panel a) and individual study (panel b) ROB for studies reporting the effect of vitamin D on serum 25(OH)D in children ages 3-9 years

a.

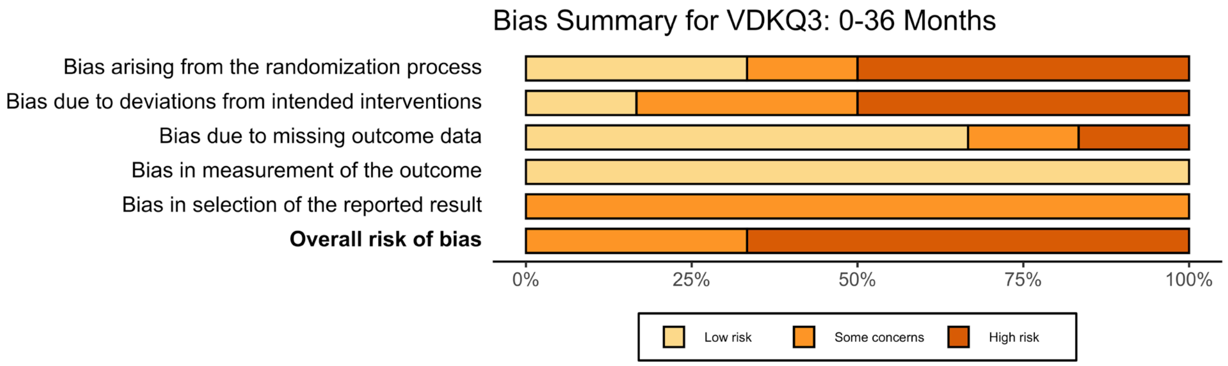

b.

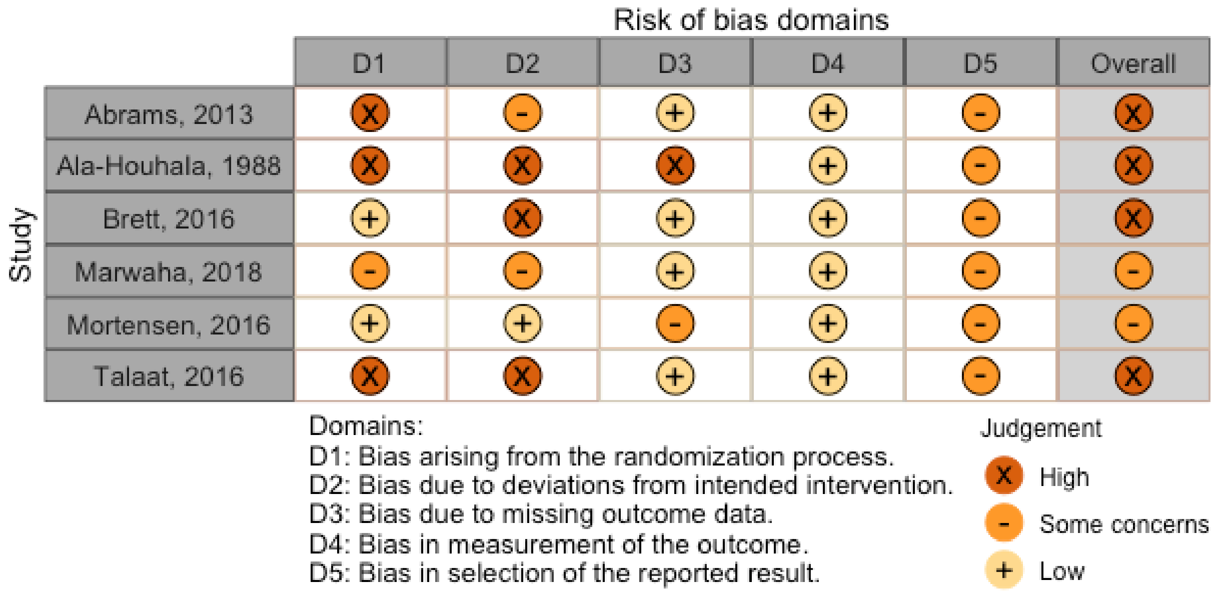

Table KQ3-4. Random-effects meta-regression analysis to examine the association between daily vitamin D supplementation doses and achieved 25(OH)D concentrations at the end of intervention period in children 3-9 years old

Meta-regression	Number of observation = 16[a]
REML estimate of between-study variance	tau^2 = 265.9
% residual variation due to heterogeneity	I^2 residual = 97.75%
Proportion of between-study variance explained with Knapp-Hartung modification	Adj R-squared = 19.96%

	Coef.	Std. Err.	t	P>t	[95% CI]	
Vitamin D daily dose (IU)	0.0249	0.0127	1.9600	0.0710	-0.0024	0.0522

Adj = adjusted; IU = international units; REML = restricted maximum likelihood

[a] 6 RCTs with total 16 intervention arms.

Figure KQ3-6. Random-effects meta-regression analysis to examine the association between daily vitamin D supplementation doses and achieved 25(OH)D concentrations at the end of intervention period in children 3 to 9 years old

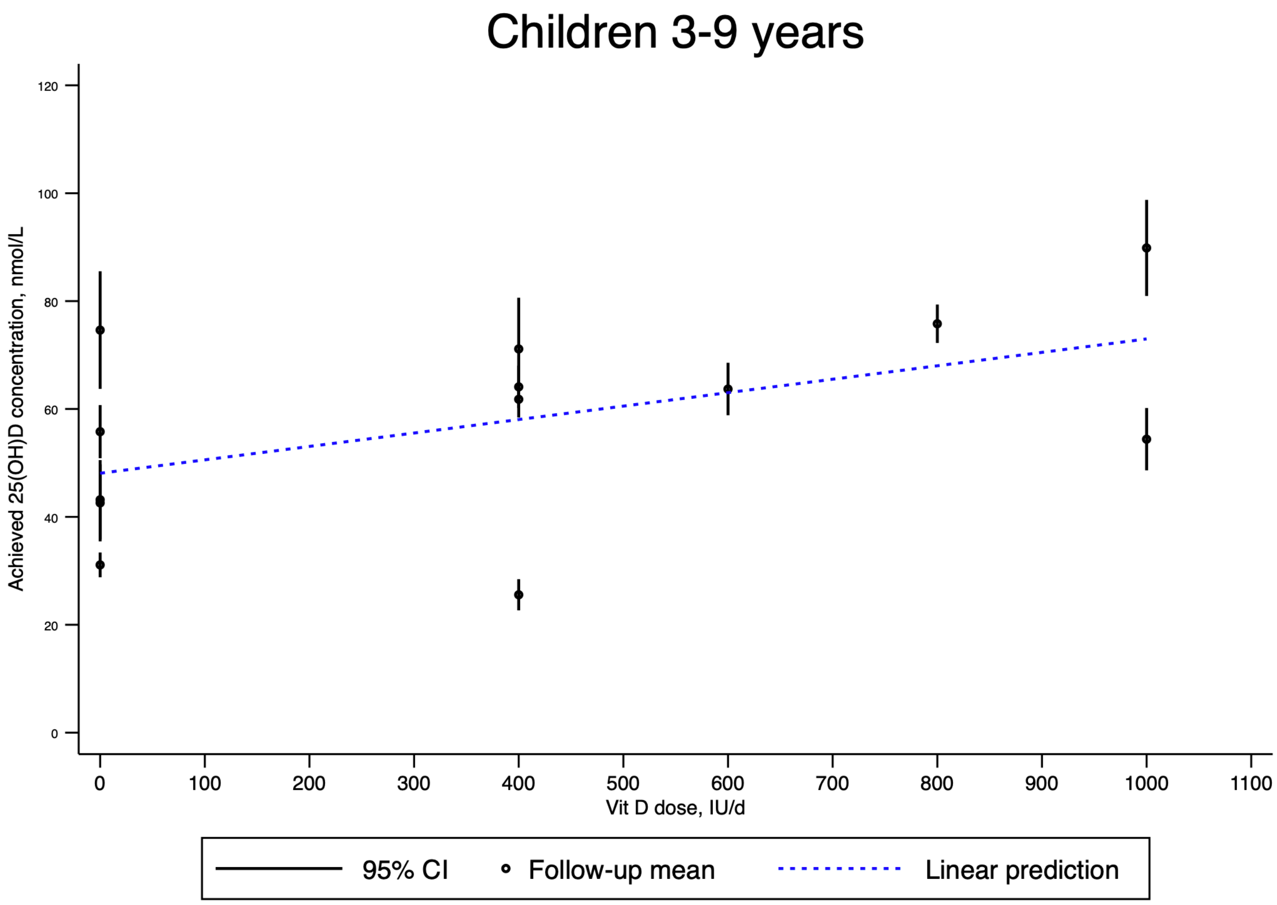

KQ3 Sub-section 2. Studies using non-daily vitamin D supplementation dosing regimens

Figure KQ3-7 shows a forest plot of 11 studies reporting the effect of non-daily vitamin D supplementation dosing regimens on serum 25(OH)D.[36,42,83,87,90,93,96,99,108,112,114] The characteristics of these studies are reported in Table KQ3-1. Among the studies that administered a single dose of vitamin D_3 supplement, the doses ranged from 50,000 to 600,000 IU. Other intervention regimens included intermittent dosing regimens, including weekly, monthly, or bimonthly dosing for variable durations. Many studies included placebo groups, other control groups (no intervention and without placebo), or daily dosing supplementation groups as comparisons. Studies reported serum 25(OH)D assessments at single or multiple time points ranging from one to 48 weeks follow-up. Information on the season or month during which serum 25(OH)D was assessed was not reported in five studies. Two studies reported that serum 25(OH)D was assessed in all seasons,[42,83] while two studies provided a specific timing for baseline serum 25(OH)D assessment and the intervention.[93,114]

Figure KQ3-7. Forest plot of studies reporting the effect of non-daily vitamin D supplementation dosing regimens on serum 25(OH)D (nmol/L) by study arms

Trial ID and Study	BF status	Other intervention info.	25(OH)D assay	Follow-up, wk	n	Mean 25(OH)D, nmol/L (95% CI)
ID: 22						
Gordon et al. (2008)	NR	VD2: 2000 IU/d	EIA/Chemiluminsecense	0	12	39.31 (33.69, 44.93)
Gordon et al. (2008)	NR	VD2: 50,000 IU once weekly	EIA/Chemiluminsecense	0	14	38.06 (33.08, 43.05)
Gordon et al. (2008)	NR	VD3: 2000 IU/d	EIA/Chemiluminsecense	0	14	38.06 (33.08, 43.05)
ID: 35						
Holstgemeiner et al. (1978)	Any BF	VD3: 1200 IU/d	nr	0	10	30.70 (26.70, 34.70)
Holstgemeiner et al. (1978)	Any BF	VD3: 1200 IU/d	nr	1	10	66.89 (46.82, 86.97)
Holstgemeiner et al. (1978)	Any BF	VD3: 1200 IU/d	nr	5	8	153.19 (107.80, 198.59)
Holstgemeiner et al. (1978)	Any BF	VD3: 200,000 IU single dose	nr	0	11	31.99 (16.15, 47.84)
Holstgemeiner et al. (1978)	Any BF	VD3: 200,000 IU single dose	nr	1	11	317.67 (202.02, 433.32)
Holstgemeiner et al. (1978)	Any BF	VD3: 200,000 IU single dose	nr	5	9	245.99 (136.92, 355.07)
ID: 36						
Huynh et al. (2017)	Any BF	VD3: 400 IU/d	EIA/Chemiluminsecense	1.5	31	48.37 (42.95, 53.79)
Huynh et al. (2017)	Any BF	VD3: 400 IU/d	EIA/Chemiluminsecense	14	23	81.29 (73.53, 89.05)
Huynh et al. (2017)	Any BF	VD3: 50,000 IU single dose	EIA/Chemiluminsecense	1.5	31	154.41 (132.76, 176.06)
Huynh et al. (2017)	Any BF	VD3: 50,000 IU single dose	EIA/Chemiluminsecense	14	26	61.90 (55.87, 67.93)
ID: 38						
Ohlund et al. (dark skinned children) (2017)	NA	Fortified food: 1000 IU/d VD3	LC-MS/MS	0	40	48.00 (42.42, 53.58)
Ohlund et al. (dark skinned children) (2017)	NA	Fortified food: 1000 IU/d VD3	LC-MS/MS	12	38	77.00 (71.59, 82.41)
Ohlund et al. (dark skinned children) (2017)	NA	Fortified food: 400 IU/d VD3	LC-MS/MS	0	38	51.00 (45.59, 56.41)
Ohlund et al. (dark skinned children) (2017)	NA	Fortified food: 400 IU/d VD3	LC-MS/MS	12	33	67.00 (61.46, 72.54)
Ohlund et al. (dark skinned children) (2017)	NA	Fortified food: 80 IU/d VD3	LC-MS/MS	0	17	39.00 (31.39, 46.61)
Ohlund et al. (dark skinned children) (2017)	NA	Fortified food: 80 IU/d VD3	LC-MS/MS	12	16	39.00 (31.92, 46.08)
Ohlund et al. (fair-skinned children) (2011)	NA	Fortified food: 1000 IU/d VD3	LC-MS/MS	0	45	66.00 (60.45, 71.55)
Ohlund et al. (fair-skinned children) (2011)	NA	Fortified food: 1000 IU/d VD3	LC-MS/MS	12	45	86.00 (81.03, 90.97)
Ohlund et al. (fair-skinned children) (2011)	NA	Fortified food: 400 IU/d VD3	LC-MS/MS	0	40	61.00 (56.04, 65.96)
Ohlund et al. (fair-skinned children) (2011)	NA	Fortified food: 400 IU/d VD3	LC-MS/MS	12	35	72.00 (67.43, 76.57)
Ohlund et al. (fair-skinned children) (2011)	NA	Fortified food: 80 IU/d VD3	LC-MS/MS	0	22	58.00 (50.90, 65.10)
Ohlund et al. (fair-skinned children) (2011)	NA	Fortified food: 80 IU/d VD3	LC-MS/MS	12	19	58.00 (51.53, 64.47)
ID: 39						
Kumar et al. (2011)	Any BF	Placebo	RIA kits	24	237	36.00 (32.75, 39.25)
Kumar et al. (2011)	Any BF	VD3: 1400 IU once weekly	RIA kits	24	216	55.00 (52.00, 58.00)
ID: 44						
Loeb et al. (2019)	NA	Placebo	EIA/Chemiluminsecense	0	542	65.21 (63.79, 66.63)
Loeb et al. (2019)	NA	VD: 14,000 IU once weekly	EIA/Chemiluminsecense	0	553	65.73 (64.34, 67.12)

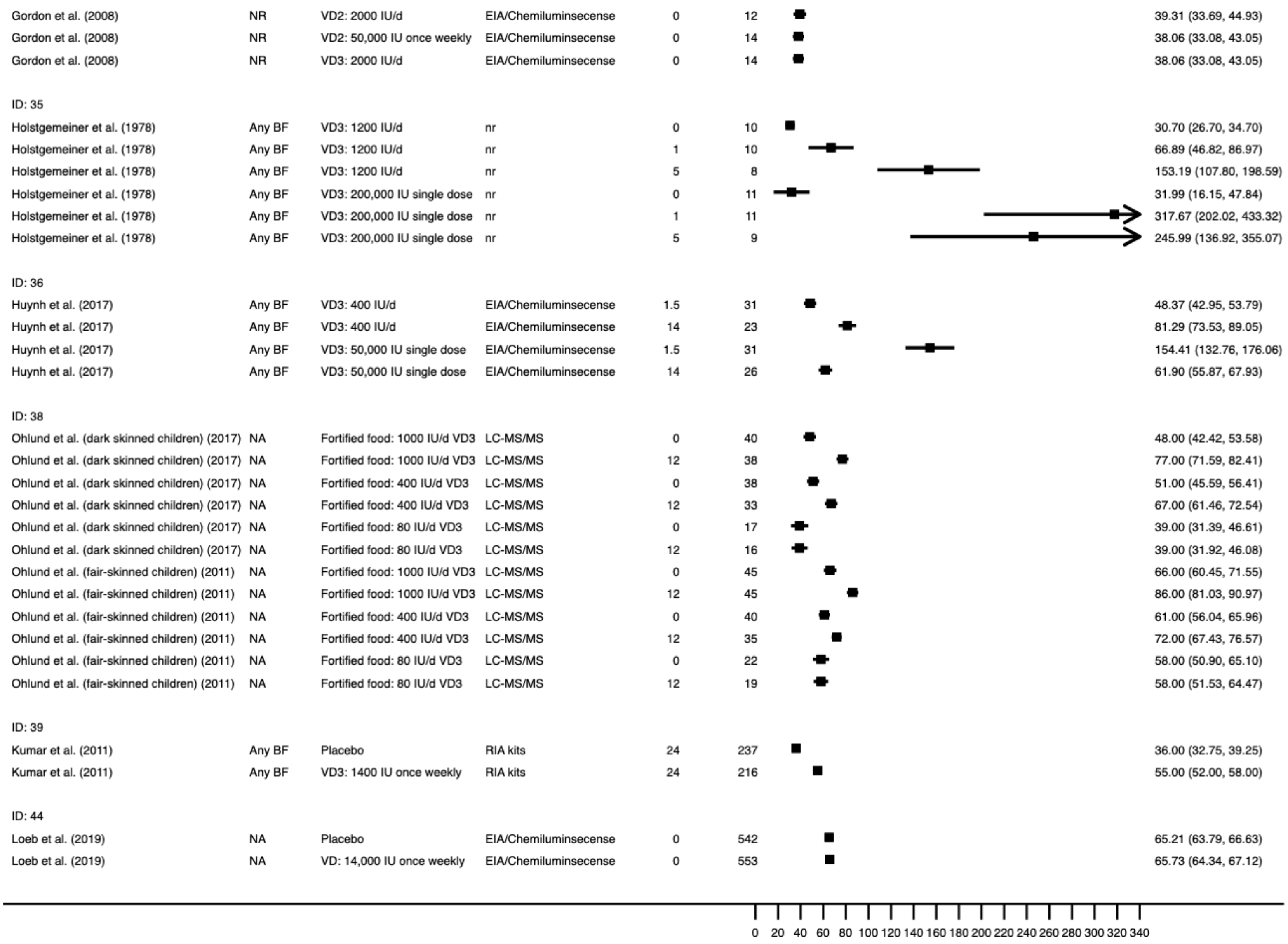

Figure KQ3-7 (continued). Forest plot of studies reporting the effect of non-daily vitamin D supplementation dosing regimens on serum 25(OH)D (nmol/L) by study arms

Trial ID and Study	BF status	Other intervention info.	25(OH)D assay	Follow-up, wk	n	Mean 25(OH)D, nmol/L (95% CI)
ID: 50						
Moodley et al. (2015)	Any BF	Placebo	LC-MS/MS	0	17	50.17 (43.06, 57.28)
Moodley et al. (2015)	Any BF	Placebo	LC-MS/MS	8	15	60.40 (43.80, 77.00)
Moodley et al. (2015)	Any BF	Placebo	LC-MS/MS	24	10	68.39 (54.91, 81.87)
Moodley et al. (2015)	Any BF	VD3: 50,000 IU single dose	LC-MS/MS	0	18	44.18 (37.56, 50.79)
Moodley et al. (2015)	Any BF	VD3: 50,000 IU single dose	LC-MS/MS	8	14	84.61 (72.13, 97.09)
Moodley et al. (2015)	Any BF	VD3: 50,000 IU single dose	LC-MS/MS	24	11	91.10 (73.51, 108.70)
ID: 61						
Shakiba et al. (2010)	Any BF	VD3: 200 IU/d	EIA/Chemiluminsecense	24	19	78.12 (68.59, 87.66)
Shakiba et al. (2010)	Any BF	VD3: 400 IU/d	EIA/Chemiluminsecense	24	26	95.85 (84.91, 106.78)
Shakiba et al. (2010)	Any BF	VD3: 50,000 IU once every two months	EIA/Chemiluminsecense	24	30	134.04 (116.62, 151.45)
ID: 69						
Talaat et al. (2016)	NA	VD3: 2000 IU once daily for 3 months followed by 1000 IU almost daily	EIA/Chemiluminsecense	0	194	32.15 (30.28, 34.02)
Talaat et al. (2016)	NA	VD3: 2000 IU once daily for 3 months followed by 1000 IU almost daily	EIA/Chemiluminsecense	16	194	90.46 (87.99, 92.92)
Talaat et al. (2016)	NA	VD3: 2000 IU once daily for 3 months followed by 1000 IU almost daily	EIA/Chemiluminsecense	48	194	94.10 (92.24, 95.96)
Talaat et al. (2016)	NA	VD3: 400 IU/d	EIA/Chemiluminsecense	0	196	54.09 (50.76, 57.42)
Talaat et al. (2016)	NA	VD3: 400 IU/d	EIA/Chemiluminsecense	16	196	42.28 (39.00, 45.57)
Talaat et al. (2016)	NA	VD3: 400 IU/d	EIA/Chemiluminsecense	48	196	25.56 (22.67, 28.45)
Talaat et al. (2016)	NA	VD3: 45,000 IU once weekly for 2 months followed by 400 IU almost daily	EIA/Chemiluminsecense	0	247	35.67 (33.98, 37.36)
Talaat et al. (2016)	NA	VD3: 45,000 IU once weekly for 2 months followed by 400 IU almost daily	EIA/Chemiluminsecense	16	247	119.06 (112.41, 125.71)
Talaat et al. (2016)	NA	VD3: 45,000 IU once weekly for 2 months followed by 400 IU almost daily	EIA/Chemiluminsecense	48	247	57.58 (53.63, 61.54)
ID: 75						
Zeghoud et al. (1991-1992 study) (1994)	NR	VD3: 100,000 IU single dose at baseline and 3 months	RIA kits	2	15	92.00 (70.75, 113.25)
Zeghoud et al. (1991-1992 study) (1994)	NR	VD3: 200,000 IU single dose	RIA kits	2	15	150.00 (122.17, 177.83)
ID: 81						
Mittal et al. (2014)	NR	VD3: 300,000 IU single dose	RIA kits	12	32	16.10 (12.27, 19.94)
Mittal et al. (2014)	NR	VD3: 600,000 IU single dose	RIA kits	12	28	17.60 (11.73, 23.48)

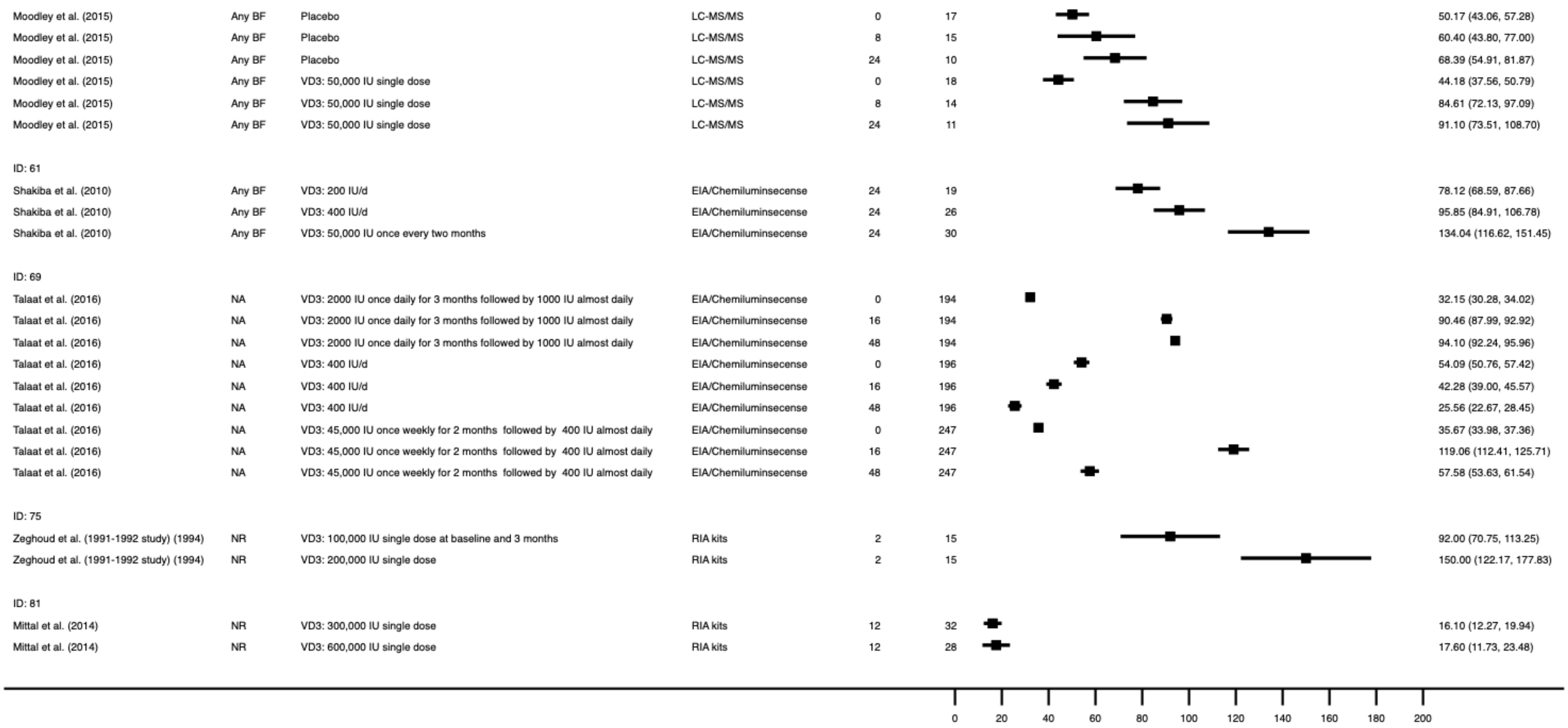

Figure KQ3-8 shows the summary and individual study ROB plots for trials in this section. More than 75% of trials had high ROB due to deviations from intended interventions. Further, 100% of trials had some ROB in selection of the reported outcomes, usually from failing to provide pre-specified analysis plans, and nearly 50% of studies had some or high ROB from the randomization process. Over 50% of studies had some or high ROB due to missing outcome data. Notably, 100% of studies had low ROB in measurement of the outcome.

As seen in Figure KQ3-7, single doses of 200,000 IU of vitamin D_3 increased serum 25(OH)D to above 317 nmol/L at one week and 246 nmol/L at 5 weeks in one study,[87] and 150 nmol/L at two weeks in another study.[96] A single dose of 100,000 IU of vitamin D_3 resulted in serum 25(OH)D levels of 92 at two weeks.[96] Two studies used a single dose of 50,000 IU of vitamin D_3, with serum 25(OH)D measuring 154 and 62 nmol/L at 1.5 and 14 weeks in one study,[36] and measuring 85 and 91 nmol/L at eight and 14 weeks in the other study.[90] One single-dose study compared 300,000 IU to 600,000 IU and found that serum 25(OH)D levels were 16.1 and 17.6 nmol/L, respectively, after 12 weeks.[99] Other dose regimens, including weekly or monthly doses of vitamin D, resulted in increased 25(OH)D. In one study, baseline was assessed in July in the northern hemisphere, and the comparison group of 400 IU/d of vitamin D_3 resulted in decreased serum 25(OH)D at follow-up assessments, while intermittent dosing regimens resulted in variable changes in serum 25(OH)D at follow-up assessments.[114] Another RCT compared the effect of 14,000 IU/wk of vitamin D_3 to placebo among children (mean age = 8.5 years) and found that the mean 25(OH)D increased to 91.8 nmol/L in the vitamin D_3 supplementation group but did not change in the placebo group (mean = 64.5 nmol/L) after eight months.[108]

One RCT reported the mean change (%) in serum 25(OH)D in infants with hypovitaminosis D after six weeks of treatment with either 2,000 IU of vitamin D_2 daily, 50,000 IU of vitamin D_2 weekly, or 2,000 IU of vitamin D_3 daily. They found the daily D_2, weekly D_2, and daily D_3 groups, which had baseline serum 25(OH)D means (SD) of 39 (10), 38 (10), and 38 (10) nmol/L, respectively, had mean (95% CI) percent increases in serum 25(OH)D of 149% (84%, 214%), 169% (111%, 226%), and 159% (100%, 219%), respectively.[83]

Figure KQ3-8. Summary ROB plot (panel a) and individual study ROB (panel b) for trials examining the effect of non-daily vitamin D supplementation dosing regimens on serum 25(OH)D

a.

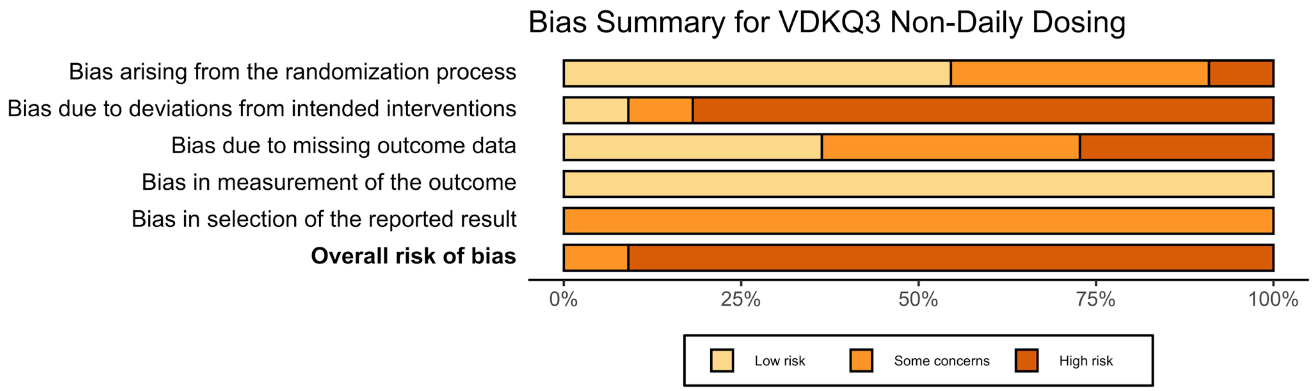

b.

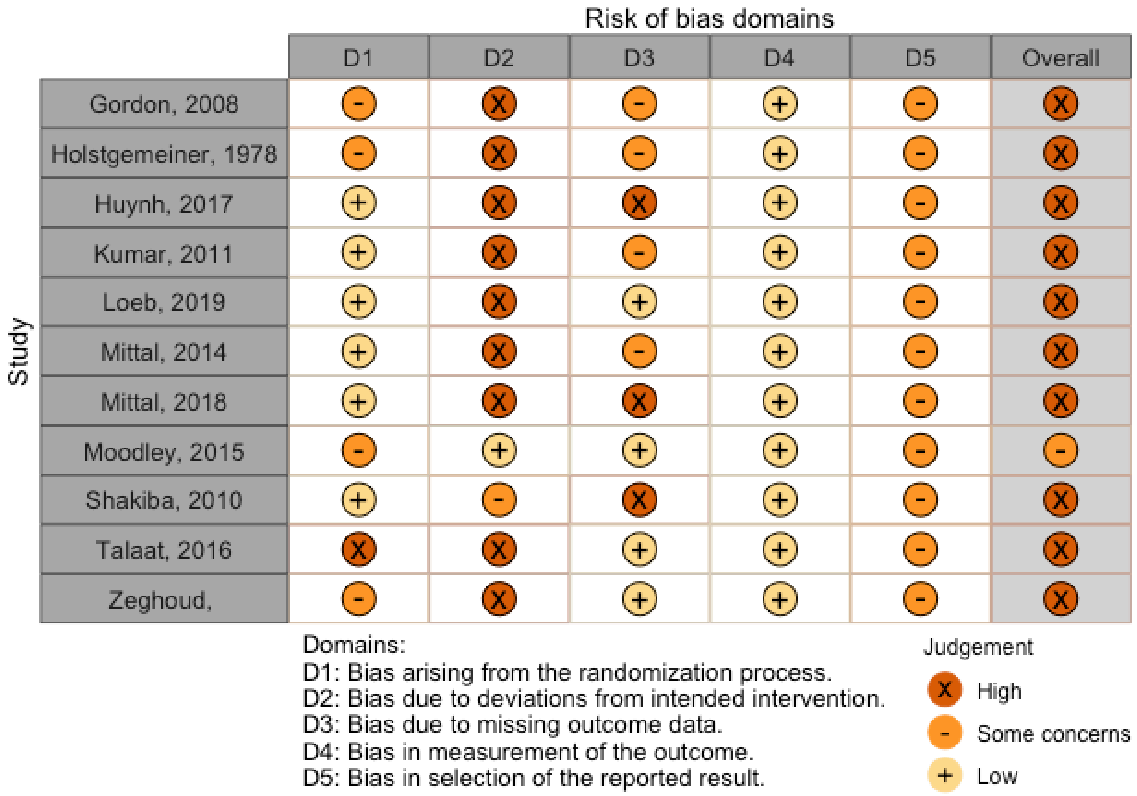

KQ3 Sub-section 3. Studies comparing supplementation for post-partum mothers to supplementation for infants

Table KQ3-5 shows the characteristics of studies comparing supplementation for post-partum mothers to supplementation for infants, including maternal exposure information. **Figure KQ3-9** shows a forest plot of four studies reporting the effect of post-partum maternal vitamin D supplementation on the serum 25(OH)D of their human milk fed infants.[41,45,51,78] Maternal supplementation included interventions of 400, 1,000, 2,000, and 6,400 IU/d of vitamin D, as well as an intermittent large dose of 120,000 IU vitamin D_3. Studies included placebo groups and direct infant supplementation groups (with or without maternal supplementation) as comparisons. Studies reported serum 25(OH)D assessments at eight, 15, 14, 16, and 28 weeks of interventions. Information on the season or month during which serum 25(OH)D was assessed was not reported in one study,[51] one study reported follow-up assessment was completed in January,[78] while the last reported baseline assessment in September and follow-up in June.[45]

Figure KQ3-10 shows the summary and individual study ROB plots for trials in this section. All trials had some ROB in selection of the reported outcomes, and this was usually from failing to provide pre-specified analysis plans. About 75% of trials had some or high ROB in two ROB domains: bias due to deviations from intended interventions and bias due to missing outcome data. Further, 50% of trials had some ROB due to the randomization process, and 25% of trials had some ROB due to measurement of the outcome.

In one RCT, the serum 25(OH)D of human milk fed infants at eight weeks follow-up had decreased in the maternal 1,000 IU/d supplementation group, while serum 25(OH)D increased in both the direct infant supplementation of 400 IU/d and 1,000 IU/d of vitamin D_3 groups.[51] Baseline serum 25(OH)D was not provided in the other trials; however, one study showed maternal supplementation of 120,000 IU vitamin D_3 following delivery and once per month for 3 months (infant placebo), as well as infant supplementation of 400 IU/d vitamin D_3 (maternal placebo), both resulted in higher infant serum 25(OH)D at 14 weeks compared to double placebo (maternal and infant).[45] In the other trial, maternal supplementation of 1,000 IU/d, but not 2,000 IU/d, resulted in infant serum 25(OH)D significantly lower than that of infants receiving direct 400 IU vitamin D_2 daily at eight weeks.[78] This difference was also significant at 15 weeks, but differences between the other groups were not significant. Lastly, in one trial of supplementation with 6,400 IU/d to breastfeeding mothers (infant placebo) compared to infant supplementation of 300 IU/d with maternal supplementation of 400 IU/d found no significant difference in serum 25(OH)D levels at 16 or 28 weeks.[41]

Table KQ3-5. Characteristics of studies assessing the effect of post-partum maternal vitamin D exposure on breastfed infants' serum 25(OH)D

Author (year); study design; N randomized	Enrollment years	Location; latitude	Mean age (SD) [range]	Male (%)	Race or ethnicity	Health status; nutritional status	Assay method	Assay standards	Intervention	Control
Ala-houhala et al. (1985) [51]; RCT; N= 92	1982	Tampere, Finland; 61°	Neonates	NR	NR	100% Healthy; NR	HPLC	CDC: no; NIST: no	Infant 400 IU/d, breastfed<br><br>Infant 1000 IU/d, breastfed	Maternal 1000 IU/d, infant breastfed (not supplemented)
Ala-Houhala et al. (1986) [78]; RCT; N= 16	1984	Tampere, Finland; 61°	Neonates	NR	NR	100% Healthy; NR	Competitive protein binding assay	CDC: no; NIST: no	Infant 400 IU/d, breastfed	Maternal 2000 IU/d, infant breastfed (not supplemented)<br><br>Maternal 1000 IU/d, infant breastfed (not supplemented)
Chandy et al. (2016) [45]; RCT; N= 230	2012-2014	India; 26°	Neonates	NR	100% Asian Indian	100% Healthy; NR	RIA kits	CDC: yes; NIST: no	Infant 400 IU/d, breastfed	Maternal 120,000 IU (within 7d of delivery then at 1.5, 2.5, and 3.5 months), infant breastfed (not supplemented)<br><br>Double placebo
Wagner et al. (2006) [41]; RCT; N= 19	NR	Charleston, South Carolina,	Neonates	47	White: 79%	100% Healthy; NR	NR	NR	Infant 300 IU/d, breastfed plus	Maternal 6400 IU/d, infant

**Author (year); study design; N randomized**	**Enrollment years**	**Location; latitude**	**Mean age (SD) [range]**	**Male (%)**	**Race or ethnicity**	**Health status; nutritional status**	**Assay method**	**Assay standards**	**Intervention**	**Control**
		United States; 33°			Hispanic: 11%<br>Black: 11%				maternal 400 IU/d	breastfed plus 0 IU/d placebo

CDC = Centers for Disease Control and Prevention; d = day; HPLC = high performance liquid chromatography; IU = international units; N = sample size; NIST = National Institute of Standards and Technology; NR = not reported; RCT = randomized controlled trial; RIA = radioimmunoassay; SD = standard deviation

Figure KQ3-9. Forest plot of studies reporting the effect of post-partum maternal vitamin D supplementation on breastfed infants' serum 25(OH)D (nmol/L) by study arms

Trial ID and Study	BF status	Other intervention info.	25(OH)D assay	Follow-up, wk	n	Mean 25(OH)D, nmol/L (95% CI)
ID: 3						
Ala-houhala et al. (1985)	Exclusively BF	Maternal VD: 1000 IU/d; infant breastfed, not supplemented	HPLC	0	32	82.37 (66.80, 97.93)
Ala-houhala et al. (1985)	Exclusively BF	Maternal VD: 1000 IU/d; infant breastfed, not supplemented	HPLC	8	32	59.90 (44.34, 75.47)
Ala-houhala et al. (1985)	Exclusively BF	VD3: 1000 IU/d	HPLC	0	29	67.39 (51.04, 83.74)
Ala-houhala et al. (1985)	Exclusively BF	VD3: 1000 IU/d	HPLC	8	29	174.72 (153.83, 195.61)
Ala-houhala et al. (1985)	Exclusively BF	VD3: 400 IU/d	HPLC	0	31	82.37 (70.95, 93.79)
Ala-houhala et al. (1985)	Exclusively BF	VD3: 400 IU/d	HPLC	8	31	117.31 (97.98, 136.64)
ID: 4						
Ala-Houhala et al. (1986)	Exclusively BF	Maternal VD: 1000 IU/d; infant breastfed, not supplemented	other (specify)	8	16	30.60 (25.86, 35.33)
Ala-Houhala et al. (1986)	Exclusively BF	Maternal VD: 1000 IU/d; infant breastfed, not supplemented	other (specify)	15	16	41.33 (33.44, 49.22)
Ala-Houhala et al. (1986)	Exclusively BF	Maternal VD: 2000 IU/d; infant breastfed, not supplemented	other (specify)	8	17	52.87 (41.83, 63.92)
Ala-Houhala et al. (1986)	Exclusively BF	Maternal VD: 2000 IU/d; infant breastfed, not supplemented	other (specify)	15	17	70.59 (55.86, 85.31)
Ala-Houhala et al. (1986)	Exclusively BF	VD2: 400 IU/d	other (specify)	8	16	59.85 (43.54, 76.16)
Ala-Houhala et al. (1986)	Exclusively BF	VD2: 400 IU/d	other (specify)	15	16	82.66 (57.94, 107.39)
ID: 14						
Chandy et al. (2016)	Exclusively BF	Maternal VD3: 120,000 IU after delivery and 1.5 months, then monthly after that; infant breastfed, not supplemented	RIA kits	14	51	60.90 (52.69, 69.11)
Chandy et al. (2016)	Exclusively BF	Placebo	RIA kits	14	54	42.30 (34.67, 49.93)
Chandy et al. (2016)	Exclusively BF	VD3: 400 IU/d	RIA kits	14	47	59.30 (51.87, 66.73)
ID: 84						
Wagner et al. (2006)	any BF	Maternal VD: 6400 IU/d; infant breastfed, not supplemented	HPLC	16		36.00 (20.32, 51.68)
Wagner et al. (2006)	any BF	Maternal VD: 6400 IU/d; infant breastfed, not supplemented	HPLC	28		46.00 (26.40, 65.60)
Wagner et al. (2006)	any BF	VD3: 300 IU/d; Maternal VD: 400 IU/d	HPLC	16		33.00 (21.24, 44.76)
Wagner et al. (2006)	any BF	VD3: 300 IU/d; Maternal VD: 400 IU/d	HPLC	28		43.00 (29.28, 56.72)

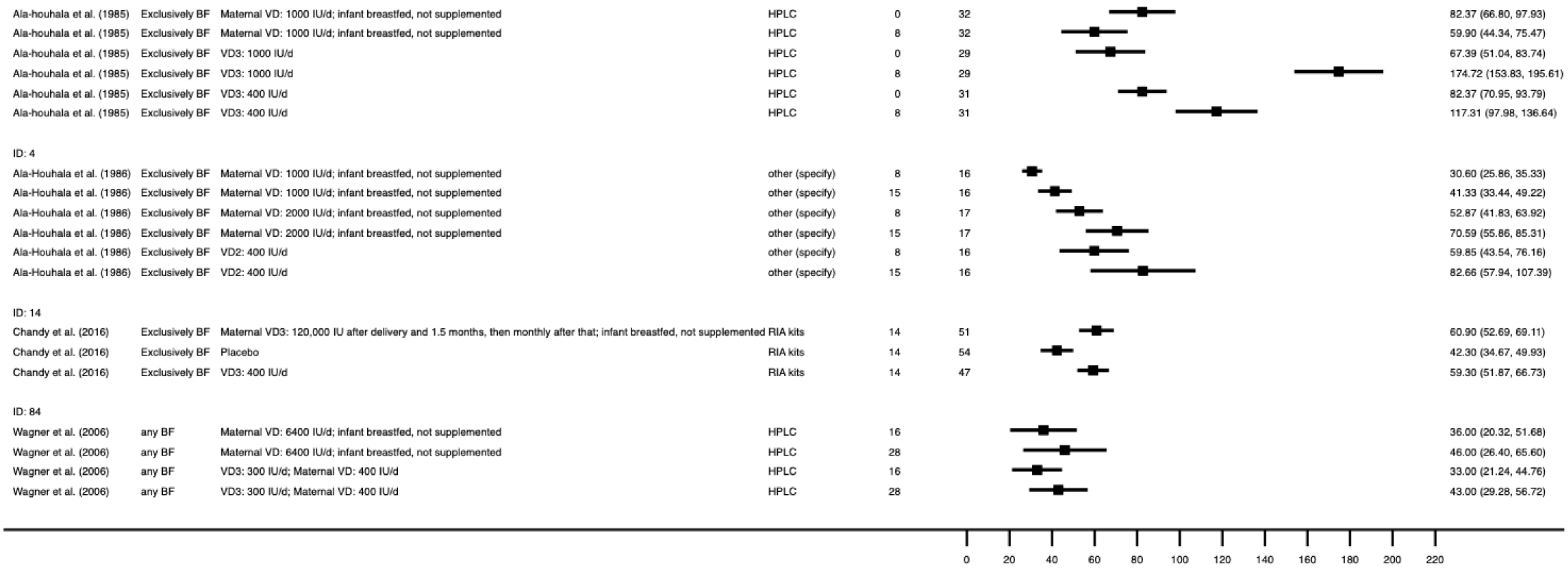

Figure KQ3-10. Summary ROB plot (panel a) and individual study ROB (panel b) for trials examining the effect post-partum maternal vitamin D supplementation on breastfed infants' serum 25(OH)D

a.

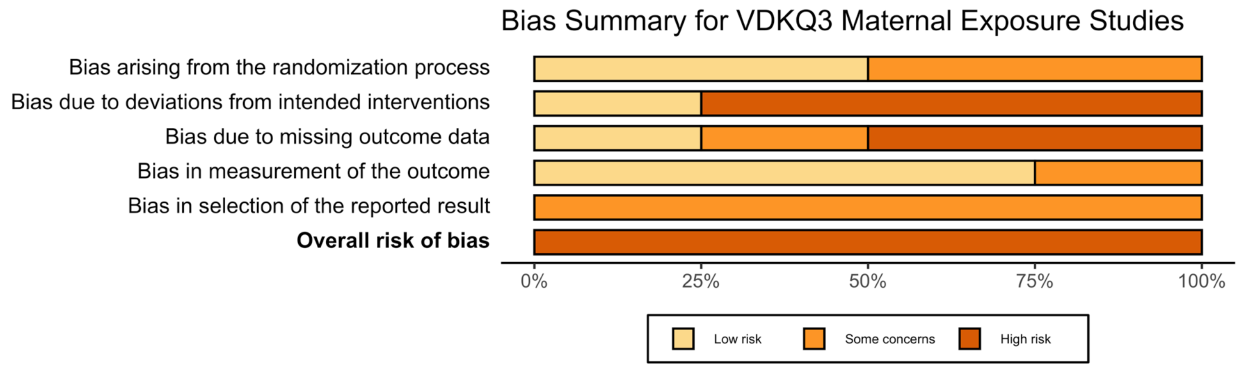

b.

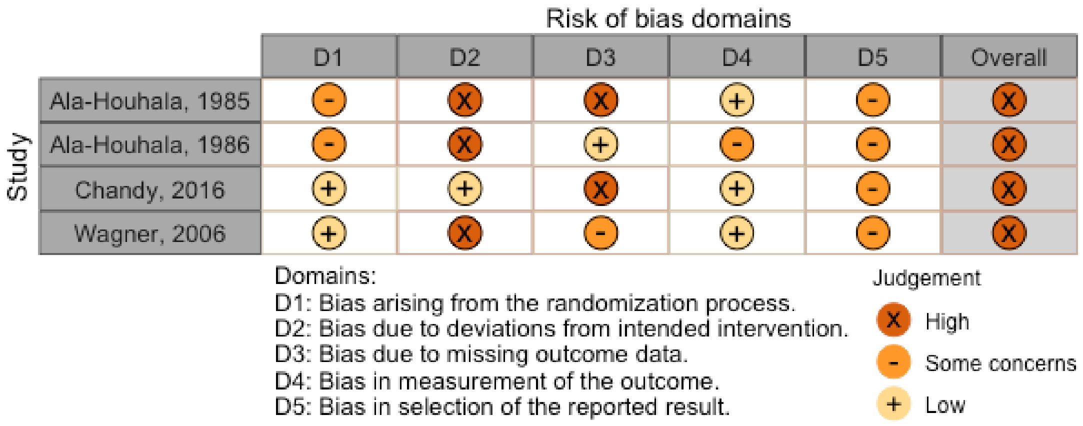

KQ3 Sub-section 4. Studies using food interventions with different levels of vitamin D or comparing food interventions with vitamin D supplements

Figure KQ3-11 shows a forest plot of three trials reporting the effect of vitamin D in fortified and non-fortified food on serum 25(OH)D.[43,104,112] Interventions included food fortified with vitamin D ranging from 80 IU/d to 1,000 IU/d, with non-fortified food, daily vitamin D supplementation, placebo, and other control groups as comparisons. Studies reported serum 25(OH)D assessments ranging from six to 22 weeks of intervention. Information on the season or month during which serum 25(OH)D was assessed was not reported in two studies,[43,104] but one trial reported baseline assessment in early winter with follow-up assessment in late winter stratified by child skin tone.[112]

Figure KQ3-12 shows the summary and individual study ROB plots for trials in this section. All three trials had some ROB in selection of the reported outcomes, usually from failing to provide pre-specified analysis plans, and all had some or high ROB due to deviations from intended interventions. One trial had high ROB due to missing outcome data and some ROB due to the randomization process. Notably, 100% of studies had low ROB in measurement of the outcome.

Results in one study showed that serum 25(OH)D decreased in groups receiving both fortified (with mean vitamin D dose of 466-486 IU/d) and non-fortified food, although none of the changes were significant.[104] In a second study, participants receiving 400 IU of vitamin D_3 supplementation daily had significantly increased serum 25(OH)D at 14 and 22 weeks; however, the placebo and fortified formula (400 IU/L) groups saw no significant increase in serum 25(OH)D.[43] The last trial reported significant increases in 25(OH)D after 12 weeks of food fortified with 1,000 IU/d and 400 IU/d in both fair- and dark-skinned children but no significant increase in the groups receiving 80 IU/d in food.[112]

Figure KQ3-11. Forest plot of studies reporting the effect of vitamin D in fortified and non-fortified foods on serum 25(OH)D (nmol/L) by study arms

Study	BF status	Other intervention info.	25(OH)D assay	Follow-up, wk	n	Mean 25(OH)D, nmol/L (95% CI)
ID: 12						
Brett et al. (2018)	NA	Fortified food: 466 IU/d	HPLC	0	26	65.30 (60.61, 69.99)
Brett et al. (2018)	NA	Fortified food: 466 IU/d	HPLC	12	26	64.70 (60.01, 69.39)
Brett et al. (2018)	NA	Fortified food: 486 IU/d	HPLC	24	26	58.40 (55.06, 61.74)
Brett et al. (2018)	NA	Non-fortified food: 239 IU/d	HPLC	0	25	67.50 (61.58, 73.42)
Brett et al. (2018)	NA	Non-fortified food: 239 IU/d	HPLC	12	23	58.30 (52.05, 64.55)
Brett et al. (2018)	NA	Non-fortified food: 241 IU/d	HPLC	24	23	56.60 (50.92, 62.28)
ID: 13						
Chan et al. (1982)	Any BF	Without intervention	other (specify)	0	20	49.92 (35.24, 64.60)
Chan et al. (1982)	Any BF	Without intervention	other (specify)	6	22	42.43 (32.65, 52.22)
Chan et al. (1982)	Any BF	Without intervention	other (specify)	14	23	42.43 (27.76, 57.11)
Chan et al. (1982)	Any BF	Without intervention	other (specify)	22	19	47.42 (37.64, 57.21)
Chan et al. (1982)	Any BF	VD3: 400 IU/d	other (specify)	0	29	34.94 (30.05, 39.84)
Chan et al. (1982)	Any BF	VD3: 400 IU/d	other (specify)	6	23	47.42 (37.64, 57.21)
Chan et al. (1982)	Any BF	VD3: 400 IU/d	other (specify)	14	24	54.91 (40.24, 69.59)
Chan et al. (1982)	Any BF	VD3: 400 IU/d	other (specify)	22	20	57.41 (42.73, 72.08)
Chan et al. (1982)	Exclusively Formula	Fortified food: dose NR (400 IU/L)	other (specify)	0	40	49.92 (40.14, 59.70)
Chan et al. (1982)	Exclusively Formula	Fortified food: dose NR (400 IU/L)	other (specify)	6	39	67.39 (47.82, 86.96)
Chan et al. (1982)	Exclusively Formula	Fortified food: dose NR (400 IU/L)	other (specify)	14	49	62.40 (52.62, 72.18)
Chan et al. (1982)	Exclusively Formula	Fortified food: dose NR (400 IU/L)	other (specify)	22	30	44.93 (35.14, 54.71)
ID: 38						
Ohlund et al. (dark skinned children) (2017)	NA	Fortified food: 1000 IU/d VD3	LC-MS/MS	0	40	48.00 (42.42, 53.58)
Ohlund et al. (dark skinned children) (2017)	NA	Fortified food: 1000 IU/d VD3	LC-MS/MS	12	38	77.00 (71.59, 82.41)
Ohlund et al. (dark skinned children) (2017)	NA	Fortified food: 400 IU/d VD3	LC-MS/MS	0	38	51.00 (45.59, 56.41)
Ohlund et al. (dark skinned children) (2017)	NA	Fortified food: 400 IU/d VD3	LC-MS/MS	12	33	67.00 (61.46, 72.54)
Ohlund et al. (dark skinned children) (2017)	NA	Fortified food: 80 IU/d VD3	LC-MS/MS	0	17	39.00 (31.39, 46.61)
Ohlund et al. (dark skinned children) (2017)	NA	Fortified food: 80 IU/d VD3	LC-MS/MS	12	16	39.00 (31.92, 46.08)
Ohlund et al. (fair-skinned children) (2011)	NA	Fortified food: 1000 IU/d VD3	LC-MS/MS	0	45	66.00 (60.45, 71.55)
Ohlund et al. (fair-skinned children) (2011)	NA	Fortified food: 1000 IU/d VD3	LC-MS/MS	12	45	86.00 (81.03, 90.97)
Ohlund et al. (fair-skinned children) (2011)	NA	Fortified food: 400 IU/d VD3	LC-MS/MS	0	40	61.00 (56.04, 65.96)
Ohlund et al. (fair-skinned children) (2011)	NA	Fortified food: 400 IU/d VD3	LC-MS/MS	12	35	72.00 (67.43, 76.57)
Ohlund et al. (fair-skinned children) (2011)	NA	Fortified food: 80 IU/d VD3	LC-MS/MS	0	22	58.00 (50.90, 65.10)
Ohlund et al. (fair-skinned children) (2011)	NA	Fortified food: 80 IU/d VD3	LC-MS/MS	12	19	58.00 (51.53, 64.47)

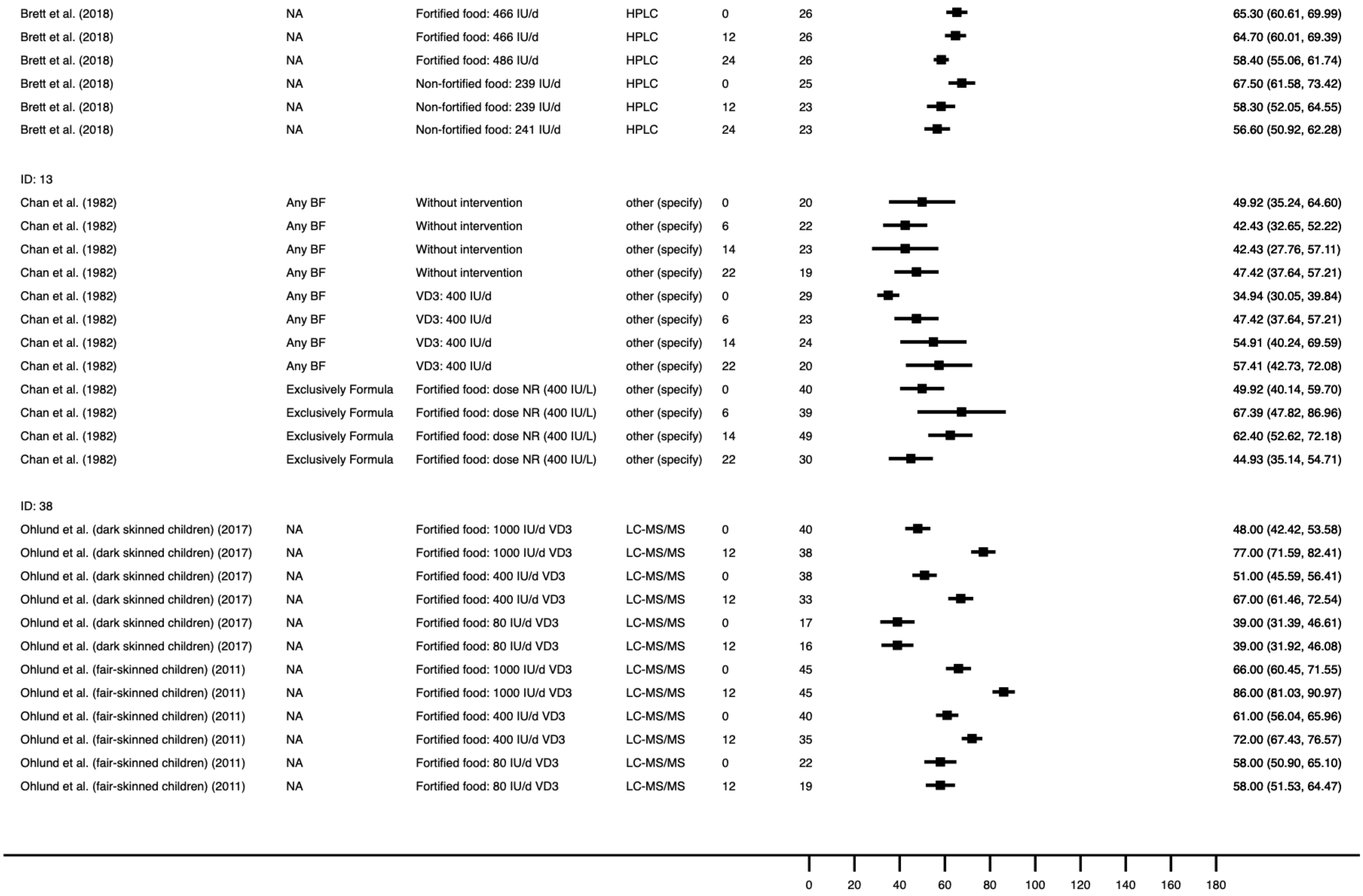

Figure KQ3-12. Summary ROB plot (panel a) and individual study ROB (panel b) for trials examining the effect of vitamin D in fortified and non-fortified foods on serum 25(OH)D

a.

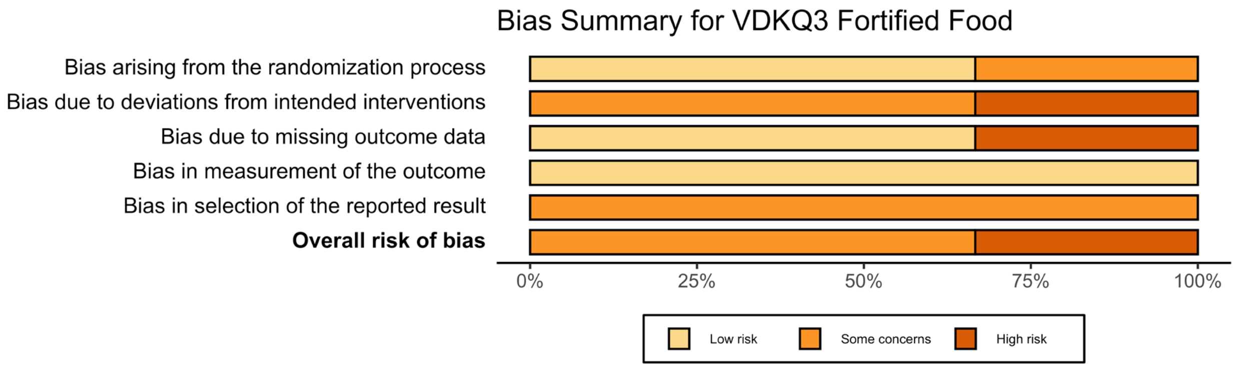

b.

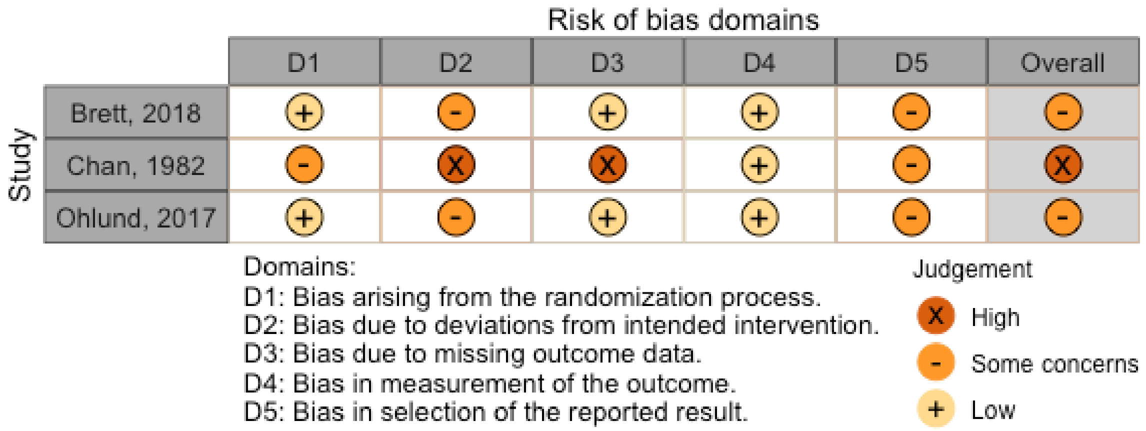

KQ3 Sub-section 5. Studies examining the effects of combined vitamin D and calcium supplementation

Figure KQ3-13 shows a forest plot of three studies reporting the effect of combined vitamin D and calcium on serum 25(OH)D. One study randomized 101 participants to 200 IU/d of vitamin D_3 plus 700 mg/d of calcium supplementation or 700 mg/d of calcium supplementation without vitamin D supplementation. After 12 weeks, mean serum 25(OH)D levels in the vitamin D plus calcium group increased significantly more than that in the calcium only (+12.7 nmol/L [5.09 ng/mL]; 95% CI 1.3, 24.1).[105] The second study randomized 60 participants to 405 mg or 156 mg of calcium five times weekly. Each group also received 30,000 IU of vitamin D_3 once monthly. After 48 weeks, there was no significant difference in serum 25(OH)D concentration between the two groups.[81] The last study randomized 30 infants to either 30,000 IU once weekly or 4,000 IU/d of vitamin D_3.[56] Each group also received 50 mg/d of calcium for every kg of body weight. After 48 weeks, serum 25(OH)D had increased significantly; however, there was no significant difference between groups. Information on season or month of serum 25(OH)D assessment was not reported for any study.

Figure KQ3-14 shows the summary and individual study ROB plots for these three RCTs. All three studies had some or high ROB in three ROB domains. All three trials had low ROB due to measurement of the outcome, but an overall high risk of bias rating.

Figure KQ3-13. Forest plot of studies reporting the effect of the effect of combined vitamin D and calcium on serum 25(OH)D (nmol/L) by study arms

Trial ID and Study	BF status	Other intervention info.	25(OH)D assay	Follow-up, wk	n	Mean 25(OH)D, nmol/L (95% CI)
ID: 10						
Ekbote et al. (2011)	NR	30,000 IU VD3 once monthly + 156 mg Ca 5 times per week	RIA kits	0	30	18.90 (9.24, 28.56)
Ekbote et al. (2011)	NR	30,000 IU VD3 once monthly + 156 mg Ca 5 times per week	RIA kits	48	28	58.10 (53.69, 62.51)
Ekbote et al. (2011)	NR	30,000 IU VD3 once monthly + 405 mg Ca 5 times per week	RIA kits	0	30	25.00 (15.30, 34.70)
Ekbote et al. (2011)	NR	30,000 IU VD3 once monthly + 405 mg Ca 5 times per week	RIA kits	48	30	64.30 (55.75, 72.85)
ID: 16						
Economos et al. (2014)	NA	200 IU/d VD3 + 700 mg/d Ca	other (specify)	0	53	64.20 (39.22, 89.18)
Economos et al. (2014)	NA	200 IU/d VD3 + 700 mg/d Ca	other (specify)	12	34	92.80 (80.70, 104.90)
Economos et al. (2014)	NA	700 mg/d Ca	other (specify)	0	48	64.30 (58.39, 70.21)
Economos et al. (2014)	NA	700 mg/d Ca	other (specify)	12	25	80.80 (68.84, 92.76)
ID: 83						
Rao et al. (2016)	NR	30,000 IU once weekly + 50 mg/kg/d Ca	EIA/Chemiluminsecense	12	15	29.08 (27.23, 30.93)
Rao et al. (2016)	NR	30,000 IU once weekly + 50 mg/kg/d Ca	EIA/Chemiluminsecense	48	15	51.12 (50.17, 52.07)
Rao et al. (2016)	NR	4000 IU/d VD3 + 50 mg/kg/d Ca	EIA/Chemiluminsecense	12	15	28.02 (24.35, 31.69)
Rao et al. (2016)	NR	4000 IU/d VD3 + 50 mg/kg/d Ca	EIA/Chemiluminsecense	48	15	47.58 (44.46, 50.70)

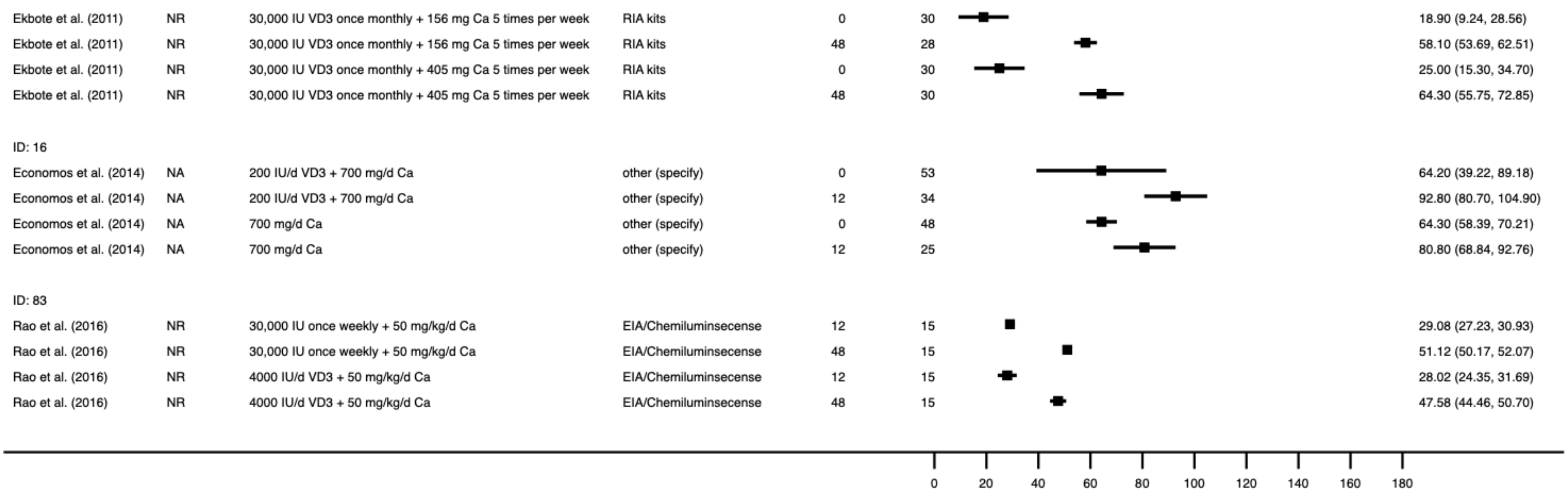

Figure KQ3-14. Summary ROB plot (panel a) and individual study ROB (panel b) for trials examining the effect of combined vitamin D plus calcium on serum 25(OH)D

a.

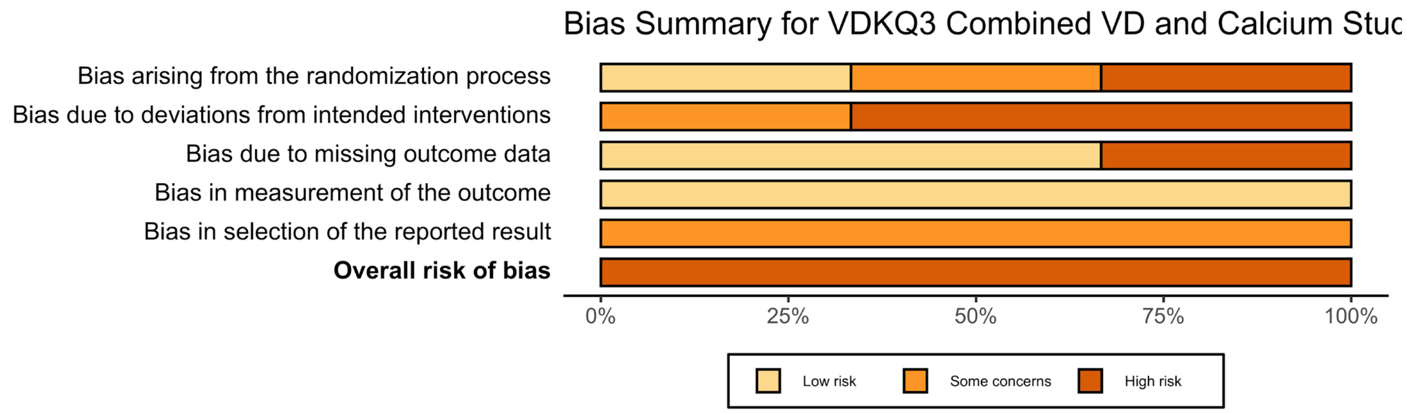

b.

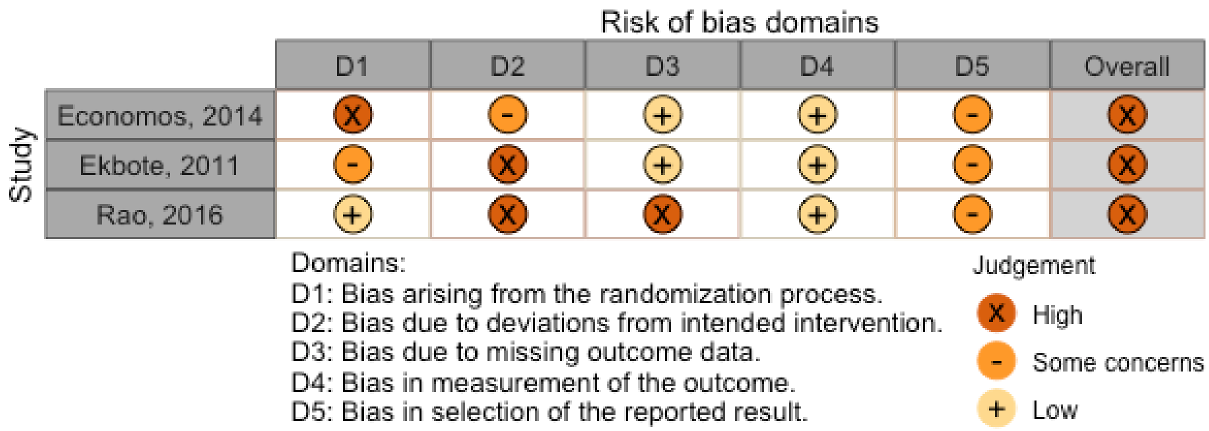

II. Vitamin D Upper Limits

ULs KQ1a. At what levels of vitamin D intake are adverse effects observed in children aged 0 to 4 years?

The below section includes interventional, observational, and case report studies that assess upper limit (UL) outcomes. The UL outcomes include hypercalcemia, hypercalciuria, nephrocalcinosis, kidney stones, and overweight and obesity.

Figure VDUL-1 displays the summary and individual outcome ROB for all interventional studies included in this section. All studies were assessed to be of some or high ROB due to deviations from intended interventions, such as high levels of non-adherence to the intervention. Many of the included studies were also prone to bias due to missing outcome data with no evidence to suggest results were not biased due to this missingness. Other potential sources of bias are attributed to poorly described or inappropriate randomization processes or a lack of evidence that analysis and statistical plans were pre-specified before unblinded outcome data were available. Almost all studies were assessed to have low risk of bias for the measurement of outcomes.

No ROB appraisals were performed for observational studies and case reports. Of note, there are currently no validated ROB assessment tools for cross-sectional studies and case reports.

Interventional Studies

Daily or less frequent vitamin D supplementation

Table VDUL-1 shows the characteristics and results of 11 interventional studies included in our review that investigate the effect of a daily dose or less frequent vitamin D supplementation on upper limit outcomes. All studies were conducted in children 0-12 months.

All studies used an RCT design.[24,31,35,36,38,39,42,44,52,92,116] Most studies were conducted in North America, Europe, and Asia, with one study conducted in Australia. Most studies included infants with any breastfeeding status (mixed feeding) or did not specify feeding status. The health and nutritional status of subjects was characterized as healthy term infants in most studies, but three studies were in preterm or low birthweight infants.[24,39,42] Vitamin D deficiency was usually defined as a serum 25(OH)D level <20 ng/mL (50 nmol/L). Ten studies administered daily vitamin D_3 supplementation ranging from 200 IU/d to 2,000 IU/d, and one study administered 1,400 IU vitamin D_3 supplementation once weekly, versus placebo.[42]

Hypercalcemia was a reported outcome in seven RCTs. Five of these (with administered vitamin D doses ranging from 400 to 1,600 IU/d) reported no hypercalcemia during the study periods.[31,35,39,52,116] In a study with vitamin D interventions of four different doses, the 800, 1,200, and 1,600 IU/d groups each had two participants with suspected hypercalcemia compared to none with suspected hypercalcemia in the 400 IU/d group (no statistical comparisons were reported).[38]In another study among preterm infants, the incidence of hypercalcemia (calcium > 10.7 mg/dL) by age 6 months was lower in the group supplemented with 400 IU/d vitamin D compared to the placebo group, but this difference was not statistically significant (Risk Difference % = - 5.4; 95% CI -13.0, 1.9).[24]

Hypercalciuria was a reported outcome in five RCTs[35,38,39,44,52] and was assessed by spot urine in all five. The incidence of hypercalciuria showed no patterns but was variable across studies and across intervention arms comparing different doses of vitamin D supplementation.

Mortality was a reported outcome in one large RCT (n = 2,079). The study participants were infants with low birthweight and severe vitamin D deficiency at baseline. The results showed that, at six months, 1.92% and 1.83% of infants died from all causes in the 1,400 IU of vitamin D once weekly and

placebo arms, respectively, but the rate per child year in these two groups was not statistically different (Adjusted Rate Ratio = 1.97; 95% CI 0.74, 5.28; $P = 0.18$).[42]

Nephrocalcinosis was a reported outcome in one RCT.[44] No participants were reported to have nephrocalcinosis in either the 400 IU/d of vitamin D supplementation or control group (no intervention).[44]

Single, large-dose vitamin D supplementation

Tables VDUL-2a and **VDUL-2b** show the characteristics and results of studies in our review that investigated the effect of a single, large dose of vitamin D supplementation on upper limit outcomes. This included six interventional studies from five publications (one publication reported two studies[96]) conducted in children 0-12 months (n = 3 studies) and 1-9 years (n = 3 studies).

Children 0-12 months. The studies conducted in children 0-12 months consisted of two single-arm interventions and one RCT reported in two publications (Table VDUL-2a).[96,117] The studies were conducted in healthy children in Germany and Algeria. Two studies were conducted in children with vitamin D deficiency. Administered vitamin D doses ranged from single doses of 100,000 to 600,000 IU of vitamin D_3, with follow-up ranging from 2 weeks to 20 months.

Hypercalcemia was a reported outcome in two studies. One study reported no hypercalcemia during the study periods.[96]. The other study (a single-arm interventional trial), with intervention of 600,000 IU of vitamin D once every 3-5 months, reported that none of the infants were hypercalcemic before the first dose, but 14 of the treated infants (34%) later had calcium values above the high normal limit.[117]

Hypercalciuria was an outcome reported in one study and was assessed by spot urine. The study reported no hypercalciuria at either follow-up (2 weeks and 6 months) after a single, large dose of vitamin D (600,000 IU) was administered.[96]

Children 1-9 years. The studies conducted in children 1-9 years old consisted of two RCTs[85,99] and one single-arm intervention[118] (Table VDUL-2b). The studies were conducted in India and Argentina with mean age ranging from 1.2 to 8.6 years. Health status of enrolled participants ranged from healthy to evidence of vitamin D deficiency with or without clinical evidence of rickets. Administered vitamin D doses ranged from single doses of 150,000 IU of vitamin D_2 to 600,000 IU of vitamin D_3, with follow-up ranging from 3-5 days to five months.

Hypercalcemia was reported in all three studies, but one study reported this outcome as hypercalcemia and/or hypercalciuria.[85] One study reported no hypercalcemia during the six-week follow up period for children given a single 150,000 IU dose of vitamin D_2.[118] Another study reported one case of hypercalcemia in each group (300,000 IU or 600,000 IU vitamin D_3) at four weeks, and one additional child with hypercalcemia at 12 weeks in the 600,000 IU vitamin D_3 group (statistical comparisons not reported).[99] The study reporting hypercalcemia and/or hypercalciuria found a slightly higher incidence in the group receiving 600,000 IU compared to the group with 300,000 IU of vitamin D_3, but the difference was not statistically significant at either 7-10 days or 25-30 days post therapy (for both follow-up periods, RR = 1.73; 95% CI 0.46, 6.54; $P = 0.47$).[85]

Hypercalciuria was a reported outcome in two studies and was assessed by spot urine in both. One study reported no hypercalciuria at six weeks and five months after a single, large dose of vitamin D_2 (150,000 IU) was administered.[118] The other study reported incidences of 7.1% and 18.5% 3-5 days after participants received a single dose of 300,000 or 600,000 IU of vitamin D_3, respectively, but this difference was not statistically significant (RR = 2.59; 95% CI 0.55, 12.24; $P = 0.25$).[85]

Figure VDUL-1. Summary ROB (panel a) and individual outcome ROB (panel b) for interventional studies reporting vitamin D upper limit outcomes

a.

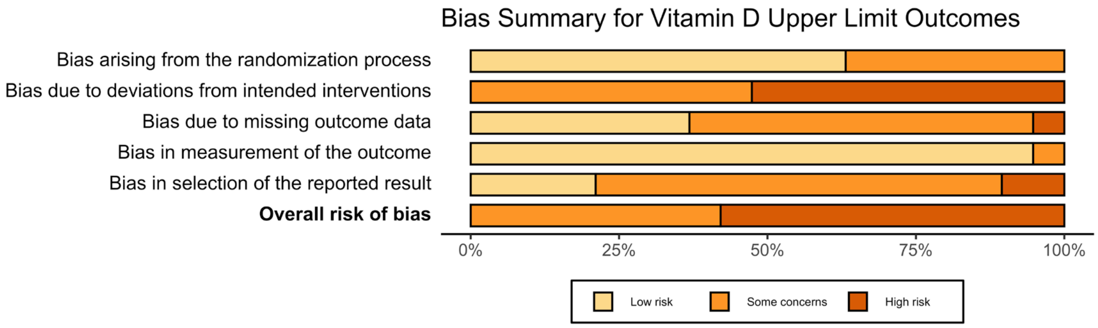

b.

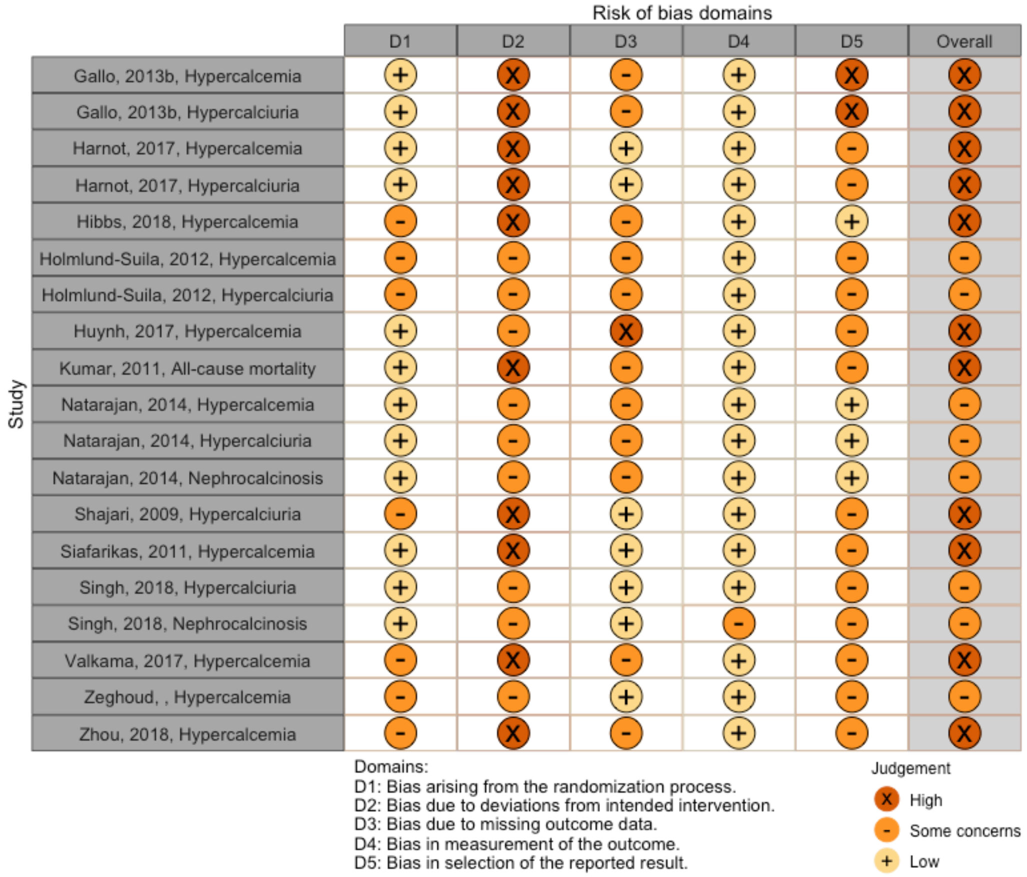

Table VDUL-1. Characteristics and key findings of interventional studies reporting the effects of a daily dose of vitamin D intake on upper limit outcomes in children 0-12 months.[a]

Author (year)	Study design; N randomized	Enrollment years	Location; latitude	Mean age (SD) [range]	Male (%)	Breast feeding status	Race or ethnicity	Health status; nutrition status	Intervention duration	Upper limit outcome incidence by intervention group
Gallo et al. (2013)b 38	RCT; N= 132	2007-2010	Montréal, Québec, Canada; 46°	Neonates	57.6	Any BF	84% white, 16% non-white	100% Healthy; NR	11 months	Hypercalcemia:<br>VD3 400 IU/d: 0.0%;<br>VD3 800 IU/d: 6.25%;<br>VD3 1200 IU/d: 7.41%;<br>VD3 1600 IU/d: 15.5%<br><br>Hypercalciuria [spot urine]:<br>VD3 400 IU/d: 0.0%;<br>VD3 800 IU/d: 3.13%;<br>VD3 1200 IU/d: 3.70%;<br>VD3 1600 IU/d: 7.69%
Hibbs et al. (2018) 24	RCT; N= 300	2013-2016	Cleveland, USA; Charleston, USA; Bronx, NY, USA; ~38°	Neonates	55	Any BF	100% Black or African American	100% preterm (mean GA=33); NR	6 months	Hypercalcemia:<br>VD3 400 IU/d: 6.72%;<br>Placebo: 12.12%
Holmlund-Suila et al. (2012) 35	RCT; N= 113	2010-2011	Helsinki, Finland; 60°	Neonates	50.4	Any BF	NR	100% Healthy; NR	10 weeks	Hypercalciuria [spot urine]:<br>VD3 400 IU/d vs. 1200 IU/d vs. 1600 IU/d: "Hypercalciuria occurred in 39% of all subjects, but U-Ca/Cr level was the same between intervention groups (ANOVA, p=0.623)." Gender was used a covariate for this assessment.<br><br>Symptoms of hypercalcemia:<br>VD3 400 IU/d: 0%;<br>VD3 1200 IU/d: 0%;<br>VD3 1600 IU/d: 0%
Huynh et al. (2017) 36	RCT; N= 70	2013-2014	St. Albans, Australia; -38°	Neonates	NR	Any BF	NR	100% Healthy; VD deficiency	3.5 months	Hypercalcemia:<br>VD3 400 IU/d: 26.9%;<br>VD3 50,000 IU single dose: 6.9%

Author (year)	Study design; N randomized	Enrollment years	Location; latitude	Mean age (SD) [range]	Male (%)	Breastfeeding status	Race or ethnicity	Health status; nutrition status	Intervention duration	Upper limit outcome incidence by intervention group
Kumar et al. (2011) 42	RCT; N= 2079	2007-2010	New Delhi, India; 29°	Neonates	46.7	Any BF	Presumed 100% Asian Indian	100% with low birthweight (range 1.8-2.5 kg); 100% with severe VD deficiency	6 months	All-cause incidence of death:<br>VD3 1400 IU once weekly: 1.92%;<br>Placebo: 1.83%
Natarajan et al. (2014) 39	RCT; N= 96	2011-2012	North India; ~29°	Neonates	56.3	Any BF	Presumed 100% Asian Indian	100% preterm infants (mean GA = 32.5); 81% with VD deficiency	12 weeks	Hypercalcemia:<br>VD3 400 IU/d: 0%;<br>VD3 800 IU/d: 0%<br><br>Hypercalciuria [spot urine]:<br>VD3 400 IU: 0%;<br>VD3 800 IU: 0%
Shajari et al. (2009) 92	RCT; N= 90	NR	Yazd, Iran; 32°	Neonates	NR	Exclusively BF	Presumed 100% Iranian	100% Healthy; Presumed normal	10 weeks	Hypercalciuria [spot urine]:<br>VD3 200 IU/d: 83.3%;<br>VD3 400 IU/d: 76.7%;<br>VD3 50,000 IU at baseline and 6 weeks: 93.3%
Siafarikas et al. (2011) 52	RCT; N= 40	NR	Berlin, Germany; 52.5°	Neonates	NR	Any BF	NR	100% Healthy; NR	6 weeks	Hypercalcemia:<br>VD3 250 IU/d: 0%;<br>VD3 500 IU/d: 0%
Singh et al. (2018) 44	RCT; N= 100	2013-2014	New Delhi, India; 29°	Neonates	55	Exclusively BF	Presumed 100% Asian Indian	100% Healthy; ~47% with VD deficiency	6 months	Hypercalciuria [spot urine]:<br>VD3 400 IU/d: 0%;<br>Without intervention (no placebo was used): 0%<br><br>Nephrocalcinosis:<br>VD3 400 IU/d: 0%;<br>Without intervention (no placebo was used): 0%
Valkama et al. (2017) 116	RCT; N= 987	2013	Helsinki, Finland [60.2]	Neonates	50	Any BF	Mothers >90% Caucasian	100% Healthy; NR	12 months	Severe hypercalcemia:<br>VD3 400 IU/d: 0%<br>VD3 1200 IU/d: 0%

Author (year)	Study design; N randomized	Enrollment years	Location; latitude	Mean age (SD) [range]	Male (%)	Breastfeeding status	Race or ethnicity	Health status; nutrition status	Intervention duration	Upper limit outcome incidence by intervention group
Zhou et al. (2018) [31]	RCT; N= 400	2015-2016	Yongkang, China; Wenzhou, China; Jinhua, China; ~29°	0.65 (0.22) years	52.3	Any BF	Presumed 100% Chinese	Generally healthy; Presumed normal	4 months	Hypercalcemia:<br>VD3 400 IU/d: 0%;<br>VD3 1200 IU/d: 0%

BF = breastfeeding; d = day; GA = gestational age; IU = international units; N = sample size; NR = not reported; RCT = randomized controlled trial; SD = standard deviation; U-Ca/Cr = urinary calcium to creatinine ratio; VD = vitamin D; VD3 = vitamin D_3

[a] No studies conducted in children 1-9 years were identified were identified.

Table VDUL-2a. Characteristics and key findings of interventional studies reporting the effects of a single large dose of vitamin D on upper limit outcomes in children 0-12 months

Author (year)	Study design; N randomized	Enrollment years	Location; latitude	Mean age (SD) [range]	Male (%)	Breast feeding status	Race or ethnicity	Health status; nutrition status	Follow-up	Upper limit outcome incidence by intervention group
Markestad et al. (1987) [117]	Single-arm intervention; N= 43	NR	Jena, Germany; 50.9°	~8.7 (~5.6) [1-20] months	NR	Any BF	NR	100% Healthy; NR	20 months	Hypercalcemia:<br>600,000 IU once every 3-5 months: "None of the infants were hypercalcemic before the first dose but 14 of the treated infants (34%) later had one or both Ca values above the high normal limit. The infants with high Ca levels did not differ from the others with respect to the vitamin D metabolites."<br>“All infants had normal Ca levels before the first dose but 14 infants (34%) later had one or both Ca values above the upper normal limit of 2.80 mmol/L (2.81-3.32 mmol/L), indicating that the vitamin D doses were excessive despite the lack of accumulative increases in serum vitamin D concentrations.”
Zeghoud et al. (1994) (1984-1985 study) [96]	Single-arm intervention; N= 30	1984-1992	Constantine, Algeria; 36.4°	Neonates	NR	NR	NR	100% Healthy; 100% with VD deficiency	2 weeks<br><br>6 months	Hypercalciuria [spot urine]:<br>VD3 600,000 IU single dose: 0%<br><br>Hypercalciuria [spot urine]:<br>VD3 600,000 IU single dose: 0%
Zeghoud et al. (1994) (1991-1992 study) [96]	RCT; N= 30	1991-1992	Constantine, Algeria; 36.4°	NR [0-0.75 years]	NR	NR	NR	100% Healthy; 100% with VD deficiency	9 months	Hypercalcemia:<br>VD3 100,000 IU single dose: 0%;<br>VD3 200,000 IU single dose: 0%;<br>VD3 600,000 IU single dose: 0%

BF = breastfeeding; IU = international units; N = sample size; NR = not reported; RCT = randomized controlled trial; SD = standard deviation; VD = vitamin D; VD3 = vitamin D_3

Table VDUL-2b. Characteristics and key findings of interventional studies reporting the effects of a single large dose of vitamin D on upper limit outcomes in children 1-9 years

**Author (year)**	**Study design; N randomized**	**Enrollment years**	**Location; latitude**	**Mean age (SD) [range]**	**Male (%)**	**Breastfeeding status**	**Race or ethnicity**	**Health status; nutrition status**	**Follow-up**	**Upper limit outcome incidence by intervention group**
Harnot et al. (2017) [85]	RCT; N= 60	2012-2013	Chandigarh, India; 31°	1.2 (0.79) years	68.3	NR	Presumed 100% Asian Indian	100% with evidence of VD deficiency; VD deficiency	30 days	Hypercalcemia and hypercalciuria [spot urine] at 7-10th day post therapy:<br>VD3 300,000 IU single dose: 10.71%;<br>VD3 600,000 IU single dose: 18.52%<br><br>Hypercalcemia and hypercalciuria [spot urine] at 25-30th day post therapy:<br>VD3 300,000 IU single dose: 10.71%;<br>VD3 600,000 IU single dose: 18.52%<br><br>Hypercalciuria [spot urine] at 3-5th day post therapy:<br>VD3 300,000 IU single dose: 7.14%;<br>VD3 600,000 IU single dose: 18.52%
Mittal et al. (2014) [99]	RCT; N= 76	2010-2012	Delhi, India; ~29°	~17.5 (13.2-14.4) months	55	NR	Presumed 100% Asian Indian	100% with clinical evidence of rickets	12 weeks	Hypercalcemia:<br>VD3 300,000 IU single dose: 2.6%<br>VD3 600,000 IU single dose: 5.3%
Oliveri et al. (1996) [118]	Single-arm intervention; N= 79	NR	Ushuaia, Argentina; -55°	8.6 (1.4) years	58.2	NR	NR	100% Healthy; NR	6 weeks	Hypercalcemia:<br>VD2 150,000 IU single dose: 0%
									5 months	Hypercalcemia:<br>VD2 150,000 IU single dose: 0%
									6 weeks	Hypercalciuria [spot urine]:<br>VD2 150,000 IU single dose: 0%
									5 months	Hypercalciuria [spot urine]:<br>VD2 150,000 IU single dose: 0%

BF = breastfeeding; d = day; IU = international units; N = sample size; NR = not reported; RCT = randomized controlled trial; SD = standard deviation; VD = vitamin D; VD2 = vitamin D_2; VD3 = vitamin D_3

Observational Studies

Table VDUL-3 shows the characteristics and results for three cross-sectional studies, one case-cohort study, and one cohort study included in our review. The studies investigated the association between vitamin D intake and upper limit outcomes,[119] serum 25(OH)D,[77,120,121] or both.[122] Sample sizes ranged from six to 1,718. Most studies were conducted in the northern hemisphere in healthy or generally healthy children or in children who were presumed healthy before nutrient intoxication. Three studies were conducted in infants,[77,119,122] and two were conducted in seven- and nine-year-old children.[120,121]

Hypercalcemia was a reported outcome in two studies. Both studies included children with acute or subacute vitamin D intoxication and found a high proportion with hypercalcemia. One study including children with a median vitamin D dose of 600,000 IU and serum 25(OH)D level of 322 ng/mL (805 nmol/L) found almost 60% had severe hypercalcemia, which was defined as serum calcium levels >14 mg/dL.[122] They found a significant association between serum 25(OH)D and serum calcium (rs = 0.402; $P<0.001$). The other study included children with a total vitamin D intake ranging from 264,000 to 1,500,000 IU who were found to have serum calcium levels ranging from 15.2 to 19.1 mg/dL.[119] These children were also found to have elevated calcium to creatinine ratios ranging from 1.17-2.08 (mg/mg).

Nephrocalcinosis and kidney stone findings were reported together in one study of children suffering from vitamin D intoxication. The study found that almost 50% of children had clinical findings of Nephrocalcinosis and/or kidney stones.[122]

Lastly, obesity or overweight was reported in three studies, two of which found an association between lower serum 25(OH)D and overweight or obesity in children.[120,121] The other study reported an association between higher serum 25(OH)D at birth and overweight and obesity in women, but not in men, at age 35 years.[77]

Case Reports

Table VDUL-4 summarizes 26 unique case reports of excessive vitamin D intake from 14 articles included in our review.

Table VDUL-3. Characteristics and key findings of observational studies reporting the association between vitamin D intake and upper limit outcomes

Author (year)	Study design; N enrolled	Enrolment years	Location; latitude	Mean age (SD); median age [IQR]	Male (%)	Breastfeeding status	Race or ethnicity	Health status; nutritional status	Follow-up	Exposure or comparisons	Results [a]
Demir et al. (2019) [122]	Cross-sectional; N= 74	2002-2014	Turkey; 39°	1.06 [0.65-1.60] years	61	NR	Presumed 100% Turkish	Generally healthy; 100% VD intoxication	NA	VD [median dose = 600,000 IU]; Median serum 25(OH)D = 803 nmol/L	Serum Ca mean (SD): 15 (3.2) mg/dL;<br><br>Severe hypercalcemia (>14 mg/dL) rate: 58.1%;<br><br>Serum Ca associated with serum 25(OH)D (rs=0.402, p<0.001);<br><br>Nephrocalcinosis and/or kidney stone rate: 48.5%
Jensen et al. (2017) [120]	Case-cohort; N= 1718	1981-1991	Copenagan, Denmark; 56°	7 (NR) years	Cases (49); Cohort (51)	NR	Maternal ethnicity: 73% Danish, 3% Western, 24% Non-western	Generally healthy; NR	7 years	Serum 25(OH)D quintiles at birth (nmol/l): <12.0, 12.0-19.6, 19.6-28.0, 28.0-40.8, >40.8	Overweight (>90th percentile of sex-specific BMI in parent cohort): 0 (serum 25(OH)D quintiles vs. middle quintile (ref.)) [b]
Lee et al. (2013) [121]	Cross-sectional; N= 1660	2006	Seoul, Korea; 37.6°	9 (NR) years	54	NR	100% Asian	100% healthy; NR	NA	Serum 25(OH)D quartiles (nmol/L): <38.4, 38.4-45.9, 45.9-54.2, >54.2	Obesity: ++ (all serum 25(OH)D quartiles; 4 vs 1, OR = 2.59; 4 vs. 2, OR=1.87; 4 vs. 3, OR=1) [c, d]<br><br>Increased waist circumference: ++ (all serum 25(OH)D quartiles; 4 vs 1, OR=2.96; 4 vs. 2, OR=2.32, 4 vs. 3, OR = 2.08) [c, e]
Sezer et al. (2012) [119]	Cross-sectional; N= 6	2005-2010	Istanbul, Turkey; 41°	8.0 (2.1) months	NR	NR	NR	Presumed previously healthy; 100% with	NA	264,000-1,500,000 IU total	Hypercalcemia (serum Ca concentration range) = 15.2-19.1 mg/dL;

								VD intoxication			Hypercalciuria (Urinary Ca/Cr ratio range) [spot urine] = 1.17-2.08 mg/mg
Tornhammar et al. (2014) [77]	Cohort; N= 282	1975-2010	Sweden	Neonates	56	NR	100% Swedish	Generally healthy; NR	35 years	Serum 25(OH)D at birth	Obesity in women: ++<br>Obesity in men: 0<br><br>Overweight in women: ++<br>Overweight in men: 0

Ca = Calcium; IU = international units; NA = not applicable; NR = not reported; OR = odds ratio; RCT = randomized controlled trial; ref. = reference group; rs = Spearman's rank correlation coefficient; SD = standard deviation; VD = vitamin D

[a] Results: ++ Significant difference indicating benefit of higher serum 25(OH)D levels ($p < 0.05$); + Marginally significant difference indicating benefit ($0.05 < p < 0.1$); 0 No significant difference; - Marginally significant difference indicating detriment ($0.05 < p < 0.1$); -- Significant difference indicating detriment ($p < 0.05$).
[b] Adjusts for maternal ethnicity, educational level, civil status, parity, season and year of birth, and offspring PI.
[c] Adjusts for age and sex.
[d] Quartiles of serum 25(OH)D levels: 1 indicates lowest group with 4 indicating highest group.
[e] Quintiles of serum 25(OH)D levels: 1 indicates lowest group with 5 indicating highest group.

Table VDUL-4. Case reports of excessive vitamin D intake in children 0 to 4 years

Author, year	Age, sex	Location; latitude	Vitamin D exposure	Clinical findings	Serum 25(OH)D	Serum calcium	Urinary Ca or Ca/Cr Ratio [a]	Nephro-calcinosis	Kidney stones
Gurkan, 2004 [123]	6 mo, F	Diyarbakir, Turkey; 40°	300,000 IU/d for 10 days	Anorexia, nausea, vomiting, polydipsia, polyuria, constipation, hypertension, positive craniotabes	340 ng/ml (850 nmol/L)	16.8 mg/dL	UCa/Cr: 2.0 mg/mg	No abnormality on renal US	
Vanstone, 2012 [124]	2 mo, F	NR	1400 IU/d for 2 months	NR	84 ng/mL (210 nmol/L)	10.7 mg/dL	NR		
Vanstone, 2012 [124]	2 yr, F	NR	2000 IU/d for 3 months	NR	102 ng/mL (255 nmol/L)	10.9 mg/dL	NR		
Andersen, 1954 [125]	16 mo, F	Oslo, Norway; 59.9°	250000 IU/d for 6 weeks	Vomiting, lethargy, anorexia, polydipsia, polyuria	NR	17.9 mg/dL	NR		No calcifications on ABX
Barrueto, 2005 [126]	2 yr, M	New York, USA; 40.7°	600,000 IU/d for 4 days	Vomiting, constipation, abdominal pain, lethargy, hypertension, anorexia	106 ng/mL (265 nmol/L)	14.4 mg/dL	NR		
Elarqam, 2007 [127]	3 mo, M	Fes, Morocco; 34°	600,000 IU/d for 3 weeks	Vomiting, dehydration, polyuria, polydipsia, cardiac arrest	NR	18.1 mg/dL	UCa: 270 mg/24 hours; UCa/Cr: 1.06	Hyperechoic lesions of renal pyramids on renal US	
Evliyaoglu, 2001 [128]	11 mo, F	NR	400 IU/d for 1 mo; 600,000 IU in two doses, 15 days apart	Polyuria, polydipsia, vomiting, dehydration, dyslipidemia	NR	22.0 mg/dL	NR		
Garbim, 2017 [129]	8 yr, M	NR	2,000 IU/d	Vomiting, anorexia, myalgias, polyuria, nocturia, weight loss	>150 ng/mL (375 nmol/L)	18.5 mg/dL	UCa: 439 mg/24 hrs		
Hoppe, 1992 [130]	3 mo, F	NR	300,000 IU twice daily	Failure to thrive, microscopic hematuria	108 ng/mL (270 nmol/L)	15.6 mg/dL	UCa/Cr:4.8 mol/mol	Medullary nephron-calcinosis on renal US	
Hoppe, 1992 [130]	4 mo, F	NR	300,000 IU twice daily	Hematuria, abdominal pain,	NR	NR	UCa/Cr: 1.0 mol/mol	No findings on renal US	Possible stones

Author, year	Age, sex	Location; latitude	Vitamin D exposure	Clinical findings	Serum 25(OH)D	Serum calcium	Urinary Ca or Ca/Cr Ratio [a]	Nephro-calcinosis	Kidney stones
Misselwitz, 1986 [131]	8 mo, M	NR	600,000 IU once at 2, 4, and 7 months	Vomiting, constipation, fever, hypotonia, areflexia	NR	18.4 mg/dL	NR		
Misselwitz, 1986 [131]	7 mo, F	NR	600,000 IU at 2, 4, and 6 months of age	Vomiting, constipation, polyuria, polydipsia, anorexia, intermittent fevers, hypotonia	176 ng/mL (>440 nmol/L)	14.0 mg/dL	UCa/Cr: 1.04	No findings on renal US	No calcifications on ABX
Misselwitz, 1986 [131]	4 mo, M	NR	600,000 IU at 2 and 4 months	Anorexia, constipation, lethargy	NR	10.4 mgl/dL	UCa/Cr: 0.361	No findings on renal US	No calcifications on ABX
Otto-Buczkowska, 1982 [132]	11 wk, F	Poland	300,000 IU at 5 weeks and 10 weeks	Lethargy, irritable, anorexia, vomiting, general malaise	NR	16.0 mg/dL	NR		Contrasting deposits in the urinary tract were not detected
Otto-Buczkowska, 1982 [132]	5 mo, F	Poland	300,000 IU twice followed by daily unknown dose	Constipation, vomiting, alacrima, irritability,	250 ng/mL (625 nmol/L)	12.7 mg/dL	NR		
Ross, 1952 [133]	14 mo, F	NR	10,000 IU/d for 10 months followed by 30,000 IU/d for 2 months	Fever, irritability, weight loss, vomiting, polyuria, facial palsy, leukocytosis	NR	17.8 mg/dL	NR		No calcifications on ABX
Ross, 1952 [133]	8 mo, F	NR	10,000-30,000 IU/d for 4 months	Anorexia, constipation, vomiting, failure to thrive	NR	18.7 mg/dL	NR		
Ünal, 2007 [134]	2 mo, F	NR	300,000 IU once weekly	Irritability, failure to thrive, pallor, weight loss	>160 ng/mL (400 nmol/L)	18 mg/dL	UCa/Cr: 3.1	Bilateral medullary nephrocalcinosis on renal US	
Ünal, 2007 [134]	8 mo, M	NR	300,000 IU twice	Diarrhea, anorexia, dehydration, renal parenchymal calcification	90 ng/mL (225 nmol/L)	11.7 mg/dL	UCa/Cr: 0.58	Bilateral minimal calcification of renal parenchyma on renal US	

Author, year	Age, sex	Location; latitude	Vitamin D exposure	Clinical findings	Serum 25(OH)D	Serum calcium	Urinary Ca or Ca/Cr Ratio [a]	Nephro-calcinosis	Kidney stones
Besbas, 1989 [135]	3 mo, M	Ankara, Turkey; 39.9°	45,000 IU/d for 45 days	Polyuria, vomiting, lethargy, decreased muscle tone, decreased turgor, dry mucosa, sunken eyes	NR	19.5 mg/dL	UCa/Cr: 1.3	Bilateral medullary nephrocalcinosis on renal US	No findings on intravenous pyelogram
Besbas, 1989 [135]	4 mo, M	Ankara, Turkey; 39.9°	60,000 IU/d for 4 weeks	Vomiting, lethargy, failure to thrive	NR	17.6 mg/dL	UCa/Cr: 0.7	Densely echogenic pyramids on renal US	No calcifications on ABX
Nanulescu, 1984 [136]	3 mo	NR	9,000 IU/d for total 800000 IU	Anorexia, vomiting, polyuria, hypotonia	NR	15.4 mg%	NR		
Nanulescu, 1984 [136]	6 mo	NR	10000 IU/d for total 1800000 IU	Anorexia, vomiting, constipation, polyuria, dehydration, failure-to-thrive	NR	12.4 mg%	NR		
Nanulescu, 1984 [136]	6 mo	NR	13000 IU/d for total 1600000 IU	Anorexia, vomiting, constipation, polyuria, irritability	NR	14.4 mg%	NR		
Nanulescu, 1984 [136]	10 mo	NR	16000 IU/d for total 4200000 IU	Anorexia, vomiting, failure to thrive, arrythmia, hypotonia	NR	20 mg%	NR		
Nanulescu, 1984 [136]	14 mo	NR	8500 IU/d for total 3600000 IU	Anorexia, polyuria, failure-to-thrive, hypotonia	NR	13 mg%	NR		

ABX = abdominal x-ray (plain film); d = day; F = female; IU = international units; M = male; mo = month; NR = not reported; UCa = urinary calcium; UCa/Cr = urinary calcium to creatinine ratio; US = Ultrasound; wk = week

[a] Reported as mg/mg unless otherwise specified.

ULs KQ1b. What are levels of vitamin D intake at which a prespecified threshold of serum 25(OH)D is reached in children aged 0 to 4 years?

This section includes all randomized and nonrandomized controlled trials assessing the effect of vitamin D intake on achieving prespecified thresholds of serum 25(OH)D. As indicated below, separate tables show the results of the included studies using daily dose interventions, single and intermittent large dose interventions, or interventions with fortified and non-fortified foods. The characteristics of these studies are reported in Table KQ3-1.

Daily dose interventions

Table VDUL-5 shows the 21 included studies that reported the effect of a daily dose of vitamin D on achieving prespecified thresholds of serum 25(OH)D. The table reports studies by prespecified thresholds of serum 25(OH)D, with one study reporting thresholds of 30 nmol/L (12 ng/mL), nine studies of 50 nmol/L (20 ng/mL), five studies of 75 nmol/L (30 ng/mL), five studies of 125 nmol/L (50 ng/mL), and one study of 150 nmol/L (60 ng/mL). Interventions ranged from 200 to 2,000 IU/d with intervention durations ranging from 6 weeks to 12 months. A variety of assay methods were used. In addition to daily vitamin D administration, some studies in this section also reported results for intake of placebos, no intervention (no placebo), once weekly vitamin D dosing, and maternal vitamin D exposures. Intervention groups ranged in size from 13 to 354.

Figure VDUL-2 displays the summary and individual study risk of bias (ROB) results for studies included in this section. Many studies were prone to bias for deviations from intended interventions, and this was often due to high levels of non-adherence to the intervention or the use of statistical analyses that did not aim to measure the effect of adhering to the intervention. Some included studies were also prone to bias for missing outcome data with no evidence to suggest results were not biased due to this missingness. Other potential bias arose from poorly described or inappropriate randomization processes and a lack of evidence or indication that detailed analysis and statistical plans were pre-specified before unblinded outcome data were available. Of note, all included studies had a low risk of bias in measurement of the outcome, serum 25(OH)D.

Table VDUL-5 reports the last follow-up data, but one study's primary outcome was the percentage of participants achieving serum 25(OH)D levels of 75 nmol/L (30 ng/mL).[38] In a model adjusted for race, gender, and period of birth, the results for achieving the 75 nmol/L (30 ng/mL) threshold were as follows: 800 IU/d vs. 400 IU/d (OR = 3.5; 95% CI 1.1, 11.0); 1,200 IU/d vs. 400 IU/d (OR = 9.7; 95% CI 1.9, 49.7).

As seen in Table VDUL-5, the percentage of participants reaching the prespecified threshold was variable and may have depended on the threshold level, intervention dose, and intervention duration. One study reported that 100% and 90% of participants achieved a threshold of 30 nmol/L (12 ng/mL) in the 400 IU/d of vitamin D_3 and control arms, respectfully.[113] At a threshold of 50 nmol/L (20 ng/mL), with participants receiving 400 IU/d of vitamin D_3 for durations of seven weeks to eight months, the percentage above threshold varied from 60% to 97%. For participants receiving 800 IU/d of vitamin D_3 for durations of 12 weeks to eight months, the percentage above threshold varied from 88% to 100%. At a threshold of 75 nmol/L (30 ng/mL), with participants receiving 400 IU/d of vitamin D_3 for durations of four to 11 months, the percentage above threshold varied from 55% to 93%. In two studies where participants received 800 IU/d of vitamin D_3 for durations of 6 weeks and 11 months, the percent above this threshold was 73% to 81%. The percentage of participants achieving a threshold of 75 nmol/L (30 ng/mL) was variable for other interventions and duration; however, similar to studies using a threshold of 50 nmol/L (20 ng/mL), the results consistently show that higher doses of vitamin D resulted in more

participants achieving a threshold of 75 nmol/L (30 ng/mL). Finally, at a threshold of 125 nmol/L (50 ng/mL), no participants in any of the four studies, with interventions of 400 IU/d or 800 IU/d vitamin D_3 for durations of 12 weeks to 12 months, achieved serum 25(OH)D levels above threshold. Serum 25(OH)D levels of 125 nmol/L (50 ng/mL) was achieved in 6% of participants receiving 1,000 IU/d of vitamin D_3 for eight weeks.[101] Notably, in one study reporting a threshold of 150 nmol/L (60 ng/mL), one participant (8%) receiving 400 IU/d achieved this threshold after 6 months.[93]

Single and intermittent large dose interventions

Table VDUL-6 shows the six included studies that reported the effect of a single, large dose of vitamin D or intermittent large doses of vitamin D on prespecified thresholds of serum 25(OH)D. One study used a prespecified threshold of serum 25(OH)D of 50 nmol/L (20 ng/mL), three studies used 75 nmol/L (30 ng/mL), one study used 120 nmol/L (48 ng/mL), and one study used 250 nmol/L (100 ng/mL). Interventions ranged from a single dose of 50,000 to 600,00 IU at baseline, with some arms reporting a second, large dose of vitamin D administered one month to three months after baseline dose. Comparison groups included daily vitamin D dosing and placebo groups. Studies ranged in intervention duration from 2 weeks to 6 months.

Figure VDUL-3 displays the summary and individual ROB results for studies included in this section. Almost all included studies had a high risk of bias for deviations from intended interventions, and this was most often due to high levels of non-adherence to the intervention or the use of statistical analyses that did not aim to measure the effect of adhering to the intervention. Some included studies were also prone to bias for missing outcome data with no evidence to suggest results were not biased due to this missingness. Other potential bias arose from poorly described or inappropriate randomization processes and a lack of evidence or indication that detailed analysis and statistical plans were pre-specified before unblinded outcome data were available. Of note, all included studies had a low risk of bias in measurement of the outcome, serum 25(OH)D.

As seen in Table VDUL-6, the percentage of participants reaching the prespecified threshold after receiving a single, large dose of vitamin D or intermittent large doses of vitamin D is variable. At a threshold of 120 nmol/L (48 ng/mL), 23% of participants receiving a single dose of 100,000 IU of vitamin D had achieved threshold at two weeks, while 58% receiving a single dose of 200,000 IU of vitamin D had achieved threshold.[96] In a different study, 86% of participants who received a single dose of 50,000 IU of vitamin D had achieved the lower threshold of 50 nmol/L (20 ng/mL) despite a longer follow up of 3.5 months.[36] Relatedly, 64% of participants who received a single dose of 50,000 IU of vitamin D had achieved the moderate threshold of 75 nmol/L (20 ng/mL) at six months.[90] Despite the heterogenicity of intervention dose, form of vitamin D used, follow-up duration, and threshold used, the results demonstrate higher doses of vitamin D resulted in more participants achieving the prespecified thresholds.

Food and fortified food interventions

Table VDUL-7 shows the two included studies that reported the effect of vitamin D from food and fortified food on prespecified thresholds of serum 25(OH)D. One study used an intervention of food fortified with vitamin D with a comparison group without food fortified with vitamin D.[104] The duration of this study was six months, but results were reported at both three and six months. The other study had two intervention arms with food fortified with different levels of vitamin D as well as one intervention arm without food fortified with vitamin D for a duration of three months.[112] Both studies used prespecified thresholds of vitamin D of 50 nmol/L (20 ng/mL). Intervention group sizes ranged from 23 to 84.

Figure VDUL-4 displays the summary and individual study ROB results for studies included in this section. Both studies had some risk of bias for deviations from intended interventions and bias due to reporting of results. Both studies had low risk of bias due to the randomization process, missing outcome data, and measurement of the outcome.

The two studies in this section included interventions arms of fortified food containing 466 IU/d and 480 IU/d of vitamin D_3.[104,112] The percentage of participants with serum 25(OH)D levels higher than 50 nmol/L (20 ng/mL) after three months was 88% and 92%, respectively. All participants in the group receiving 880 IU/d of vitamin D_3 through fortified food achieved this threshold.[112] Lower percentages of participants in arms receiving food not fortified with vitamin D achieved the threshold in both studies.

Table VDUL-5. Characteristics and key findings of studies that report the effect of a daily dose of vitamin D on prespecified thresholds of serum 25(OH)D

Author (year)	Assay	Intervention	Follow-up	N analyzed	N (%) above threshold
**25(OH)D threshold: 30 nmol/L (12 ng/mL)**					
Stellinga-Boelen et al. (2007) [113]	RIA kits	VD3 400 IU/d	3 month	26	26 (100)
		Without intervention (no placebo)		31	28 (90)
**25(OH)D threshold: 50 nmol/L (20 ng/mL)**					
Dawodu et al. (2019) [46]	EIA/Chemiluminescence	Maternal VD3 6000 IU/day	6 month	55	49 (89)
		Maternal VD3 600 IU/day + infant VD3 400 IU/day		47	43 (91)
Gallo et al. (2013) [82]	LC-MS/MS	VD2 400 IU/d	3 month	24	~18 (75)
		VD3 400 IU/d		26	~25 (96)
Gordon et al. (2008) [83]	EIA/Chemiluminescence	VD2 2000 IU/d	6 week	NR	NR (100)
		VD2 50,000 IU once weekly		NR	All but one participant
		VD3 2000 IU/d		NR	All but two participants
Kumar et al. (2011) [42]	RIA kits	VD3 1400 IU once weekly	6 month	216	122 (57)
		Placebo		237	63 (27)
Madar et al. (2009) [89]	HPLC	VD2 400 IU/d	7 week	22	19 (86)
		Without intervention (no placebo)		29	19 (66)
Mortensen et al. (2019) [137]	LC-MS/MS	VD3 400 IU/d	20 week	38	35 (92)
		VD3 800 IU/d		39	39 (100)
		Placebo		40	0 (0)
Natarajan et al. (2014) [39]	EIA/Chemiluminescence	VD3 400 IU/d	12 week	45	31 (65)
		VD3 800 IU/d		42	37 (88)
Singh et al. (2018) [44]	EIA/Chemiluminescence	VD3 400 IU/d	6 month	49	~24 (60)
		Without intervention (no placebo)		48	~12 (24)

Author (year)	Assay	Intervention	Follow-up	N analyzed	N (%) above threshold
Ziegler et al. (2014) [40]	RIA kits	VD3 200 IU/d	8 month	38	37 (97)
		VD3 400 IU/d		30	29 (97)
		VD3 600 IU/d		27	26 (96)
		VD3 800 IU/d		24	24 (100)
**25(OH)D threshold: 75 nmol/L (30 ng/mL)**					
Aglipay et al. (2017) [32]	Protein-binding assay	VD3 400 IU/d	4 month	354	276 (78)
		VD3 2000 IU/d		349	329 (94)
Atas et al. (2013) [80]	HPLC	VD 200 IU/d	3.5 month	75	59 (79)
		VD 400 IU/d		64	64 (100)
Gallo et al. (2013) [38]	LC-MS/MS	VD3 400 IU/d	11 month	29	~16 (55)
		VD3 800 IU/d		32	~26 (81)
		VD3 1200 IU/d		27	~25 (92)
		VD3 1600 IU/d		13	13 (100)
Grant et al. (2014) [84]	LC-MS/MS	VD3 400 IU/d	6 month	77	55 (74)
		VD3 800 IU/d		74	51 (73)
		Placebo		70	44 (57)
Holmlund-Suila et al. (2012) [35]	EIA/Chemiluminescence	VD3 400 IU/d	10 week	29	~27 (93)
		VD3 1200 IU/d		32	32 (100)
		VD3 1600 IU/d		32	32 (100)
**25(OH)D threshold: 125 nmol/L (50 ng/mL)**					
Abrams et al. (2013) [101]	EIA/Chemiluminescence	VD3 1000 IU/d	8 week	32	2 (6)
		Placebo		31	1 (3)
Brett et al. (2016) [103]	EIA/Chemiluminescence	VD3 400 IU/d	12 week	27	0 (0)
		VD3 600 IU/d		26	0 (0)
		Without intervention (no placebo)		24	0 (0)

Author (year)	Assay	Intervention	Follow-up	N analyzed	N (%) above threshold
Brett et al. (2016) [103]	RIA kits	Placebo	9 month	54	0 (0)
		VD3 400 IU/d		47	0 (0)
		Maternal VD3 120,000 IU once monthly		51	1 (2)
Mortensen et al. (2016) [111]	LC-MS/MS	VD3 400 IU/d	20 week	38	0 (0)
		VD3 800 IU/d		39	0 (0)
		Placebo		40	0 (0)
Talaat et al. (2016) [114]	EIA/Chemiluminescence	VD3 400 IU/d	12 month	196	0 (0)
		VD3 45,000 IU once weekly for 2 months followed by VD3 400 IU almost daily		247	2 (1)
		VD3 2000 IU once daily for 3 months followed by VD3 1000 IU almost daily		194	0 (0)
**25(OH)D threshold: 150 nmol/L (60 ng/mL)**					
Shakiba et al. (2010) [93]	EIA/Chemiluminescence	VD3 200 IU/d	6 month	19	0 (0)
		VD3 400 IU/d		26	1 (8)
		VD3 50,000 IU once every two months		30	6 (20)

d = day; EIA = enzyme immunoassay; HPLC = high performance liquid chromatography; IU = international units; LC-MS = liquid chromatography mass spectrometry; RIA = radioimmunoassay; VD = vitamin D; VD2 = vitamin D_2; VD3 = vitamin D_3

Figure VDUL-2. Summary ROB plot (panel a) and individual study ROB plot (panel b) for daily dose studies included in vitamin D upper limit key question 1b

a.

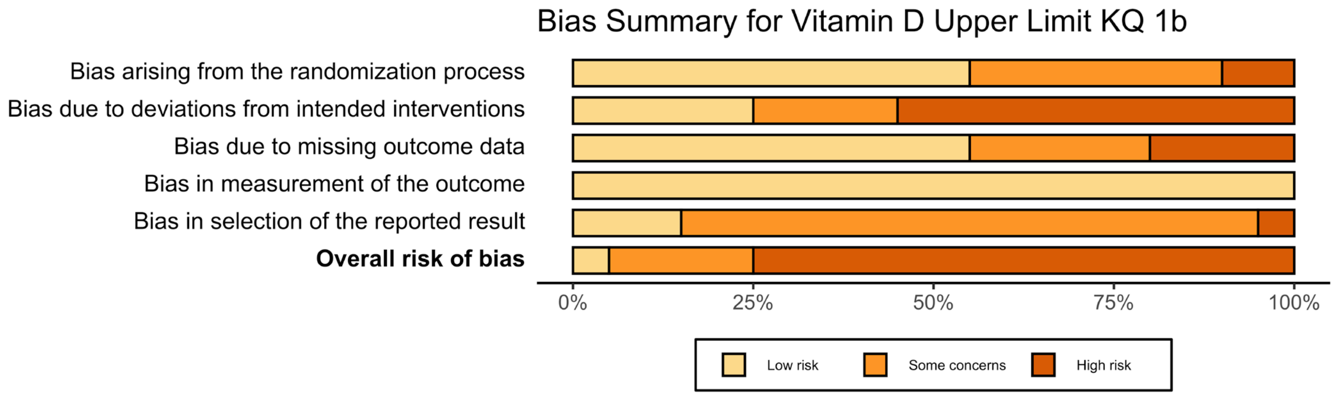

b.

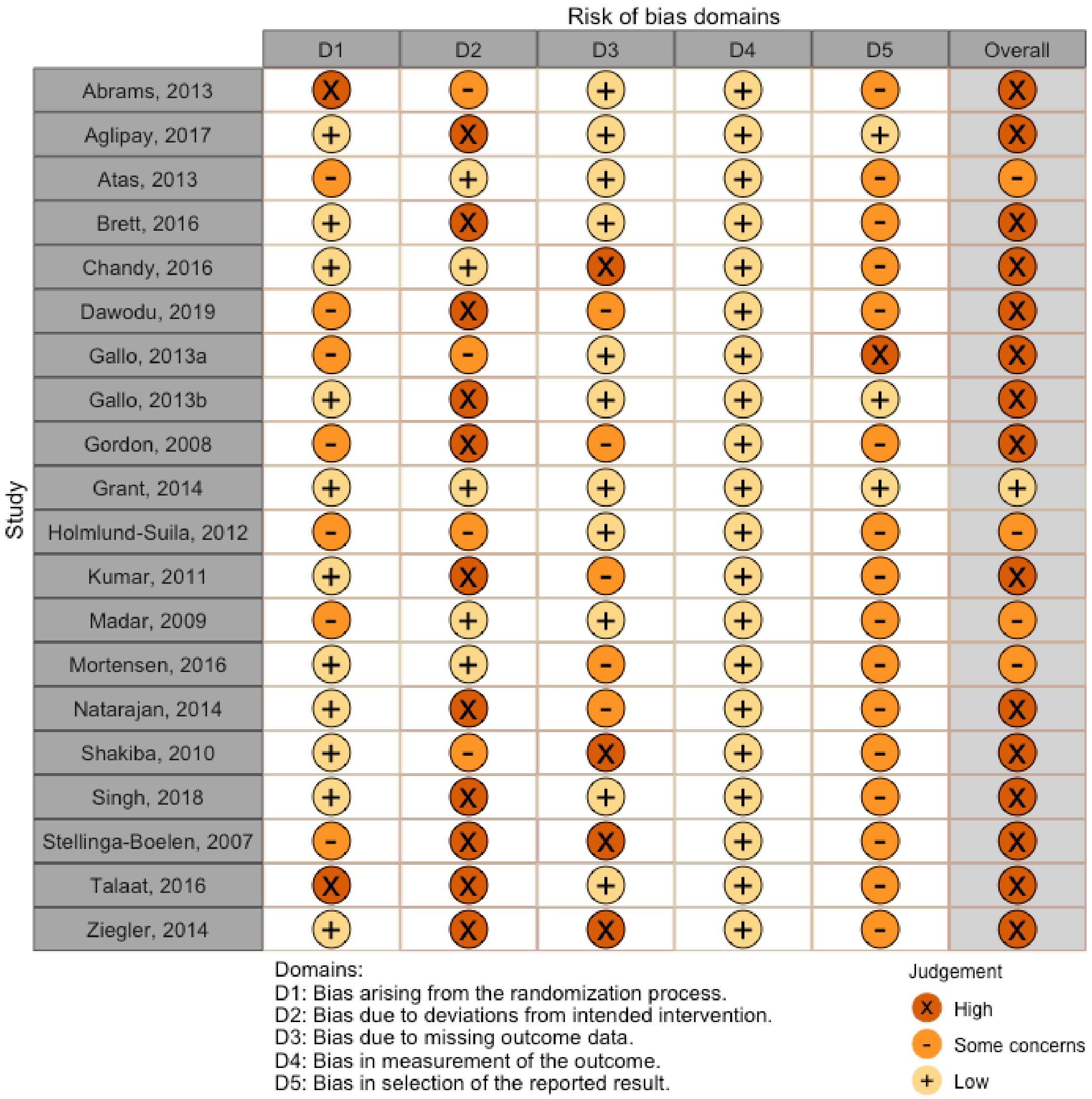

Table VDUL-6. Characteristics and key findings of studies that report the effect of a single large dose or intermittent large doses of vitamin D on prespecified thresholds of serum 25(OH)D

Author (year)	Assay	Intervention	Follow-up	N analyzed	N (%) above threshold
**25(OH)D threshold: 50 nmol/L (20 ng/mL)**					
Huynh et al. (2017) [36]	EIA/ Chemiluminescense	VD3 400 IU/d	3.5 month	22	20 (91)
		VD3 50,000 IU single dose		26	23 (86)
**25(OH)D threshold: 75 nmol/L (30 ng/mL)**					
Hirschler et al. (2014) [106]	EIA/ Chemiluminescense	VD 50,000 IU once at baseline and once at one month	2 month	36	6 (18)
		VD 100,000 IU once at baseline and once at one month		60	32 (53)
Moodley et al. (2015) [90]	LC-MS/MS	VD3 50,000 IU single dose	6 month	11	7 (64)
		Placebo		10	4 (40)
Shakiba et al. (2014) [94]	EIA/ Chemiluminescense	VD3 300,000 single dose	4 month	30	28 (93)
		VD3 400 IU/d		43	12 (28)
**25(OH)D threshold: 120 nmol/L (48 ng/mL)**					
Zeghoud et al. (1994) [96]	RIA kits	VD3 100,000 IU single dose	2 week	13	3 (23)
		VD3 200,000 IU single dose		NR	NR (58)
**25(OH)D threshold: 250 nmol/L (100 ng/mL)**					
Harnot et al. (2017) [85]	EIA/ Chemiluminescence	VD3 600,000 IU single dose	1 month	27	0 (0)
		VD3 300,000 IU single dose		28	0 (0)

d = day; EIA = enzyme immunoassay; IU = international units; LC-MS = liquid chromatography mass spectrometry; NR = not reported; RIA = radioimmunoassay; VD = vitamin D; VD3 = vitamin D_3

Figure VDUL-3. Summary ROB plot (panel a) and individual study ROB plot (panel b) for single or large dose studies included in vitamin D upper limit key question 1b

a.

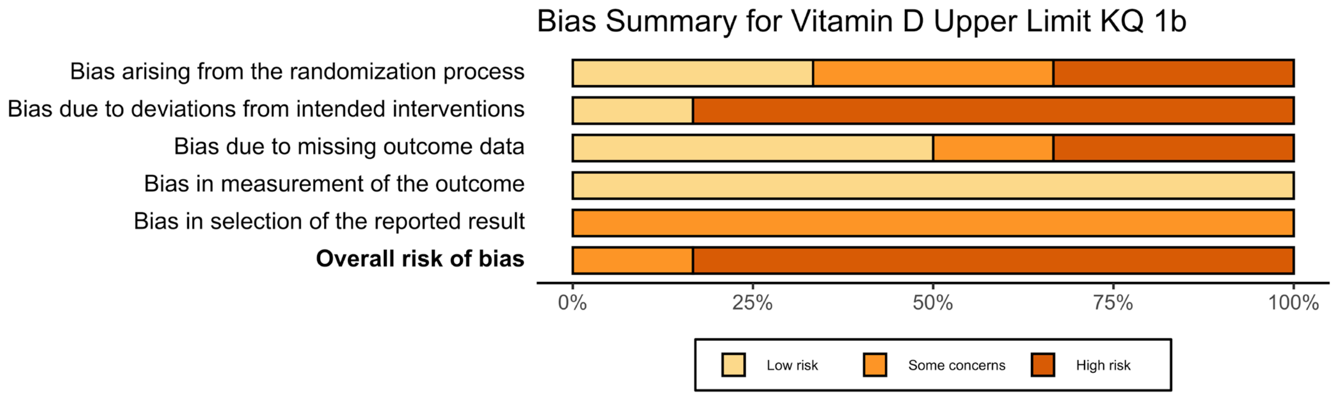

b.

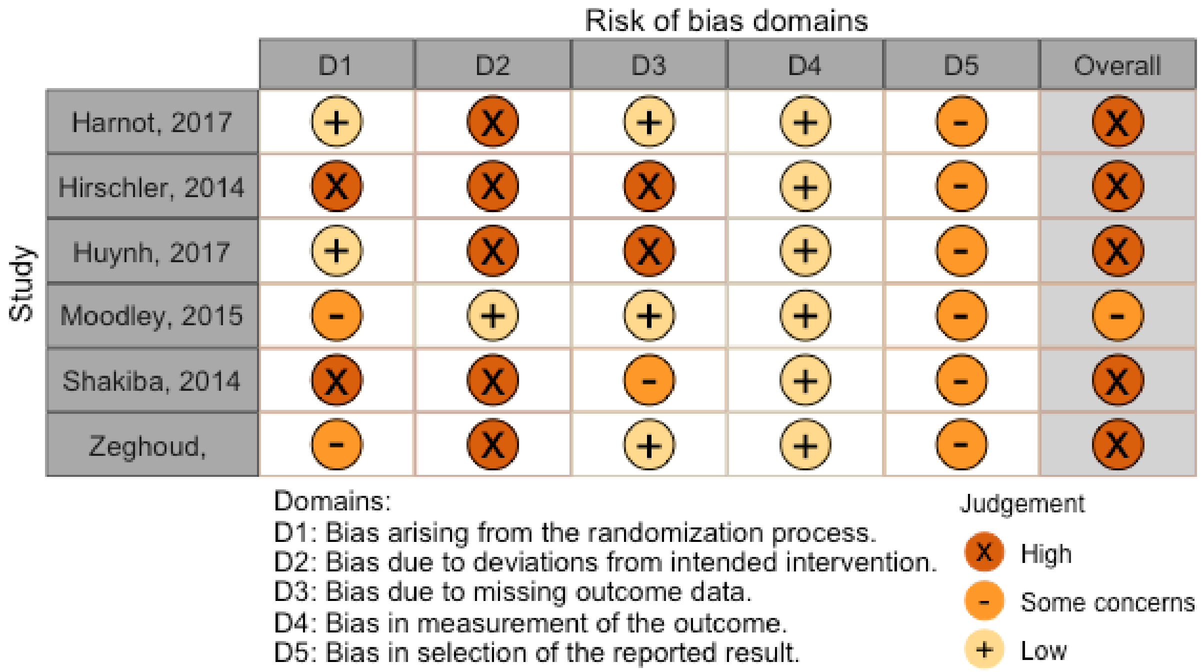

Table VDUL-7. Characteristics and key findings of studies that report the effect of vitamin D in food or fortified food on prespecified thresholds of serum 25(OH)D

Author (year)	Assay	Intervention	Follow-up	N analyzed	N (%) above threshold
**25(OH)D threshold: 50 nmol/L (20 ng/mL)**					
Brett et al. (2018) [104]	HPLC	VD3 fortified food: 466 IU/d	3 month	26	~23 (88)
			6 month	26	21 (85)
		VD3 fortified food: 486 IU/d	3 month	23	~15 (67)
		Food: 239 IU/d	6 month	23	~16 (70)
		Food: 241 IU/d			
Ohlund et al. (2017) [112]	LC-MS/MS	VD3 fortified food: 480 IU/d	3 month	69	~64 (92)
				84	84 (100)
		VD3 fortified food: 880 IU/d		35	~17 (48)
		Food: 80 IU/d			

d = day; HPLC = high performance liquid chromatography; LC-MS/MS = liquid chromatography mass spectrometry; IU = international units; N = sample size; VD3 = vitamin D_3

Figure VDUL-4. Individual study ROB plot for food and fortified food studies included in vitamin D ULs KQ1b

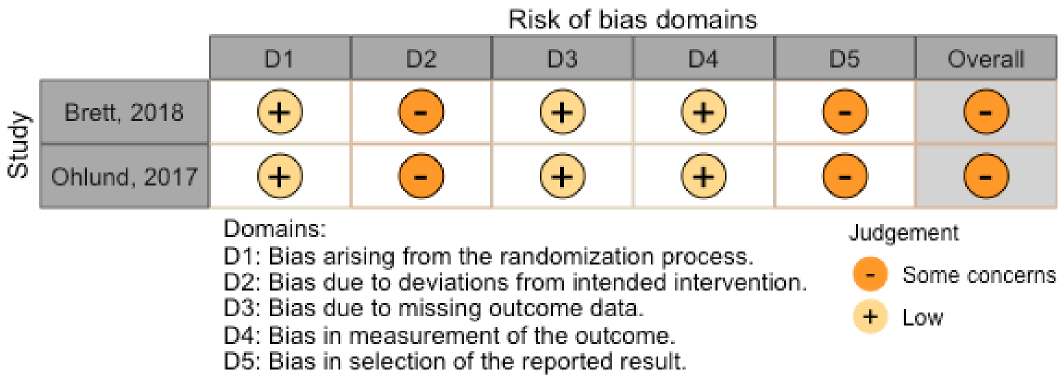

Conclusions

The following conclusions focusing on are based on evidence described above as well as strength of evidence results evaluated by the GRADE approach and summarized in **Appendix 1: GRADE Evidence Profile Tables**. A bibliography is presented in **Appendix 2**.

Vitamin D Requirements

KQ1. What is the effect of different levels of vitamin D intake on health outcomes in children aged 0 to 4 years?

- Evidence is **low** on the effect of different levels of vitamin D intake in children 0 to 4 years old for several health outcomes including atopic outcomes (i.e., asthma, wheeze, eczema), infectious diseases, growth, neurodevelopment, rickets, bone mineral content, and bone mineral density. For all or some of these outcomes, evidence was imprecise, inconsistent, without dose-response relationship, and with some or serious limitations due to risk-of-bias.
    - For asthma outcomes, the three identified RCTs reported mixed results comparing different levels of vitamin D supplementation. Two of these studies reported no significant findings,[24,25] and the other reported neonates receiving 400 IU/d of vitamin D had lower risk of asthma compared to placebo (RR = 0.06; 95% CI 0.003, 0.94).[26] For eczema,[24,25,27] three RCTs found no significant differences between groups. Results from two RCTs were mixed for wheeze outcome such that one study reported no significant findings,[27] but the other, an RCT in preterm Black infants, found significantly reduced risk of recurrent wheezing at 12 months with sustained vitamin D supplementation compared to diet-limited supplementation (adjusted RR = 0.62; 95% CI 0.44, 0.87; *P* = 0.005).[24]
    - For infectious disease outcomes, the eight identified RCTs reported a total of 20 infectious disease outcomes. Of these, 19 outcomes were not significantly different between intervention groups. One RCT found participants who received 1,200 IU/d of vitamin D_3 were significantly less likely to develop influenza A after 4 months compared to those receiving 400 IU/d of vitamin D_3 (RR = 0.54; 95% CI 0.42, 0.77).[31]
    - For growth and neurodevelopment outcomes, 12 RCTs in 100% healthy infants and two RCTs in low birth weight and/or preterm infants were identified. Ten of the studies in healthy infants reported no significant findings, and the other two reported mixed results. One study with a non-randomized comparison group reported benefits to length at 12 months for infants given formula with vitamin D vs. a placebo.[37] Another study reported significantly lower Alberta Infant Motor Scale (AIMS) total scores, prone scores, and/or sitting scores after six months for infants randomized to higher vs. lower doses of vitamin D_3 (800 vs. 400 IU/d; 1,200 vs. 400 IU/d).[47] The two RCTs in low birth weight and/or preterm infants reported mixed results. One reported no significant findings for all growth measures.[39] The other reported significant benefits with 1,400 IU/week of vitamin D_3 vs. placebo for weight- and length-for-age z scores and arm circumference at age 6 months but no difference in weight-for-length z scores or head circumference.[42] At 3-6 years post intervention, a follow-up study found significantly lower body mass index (BMI), BMI z scores, and arm muscle area in the vitamin D supplemented group but no significant differences for all other measures.[48]
    - For rickets, eight RCTs and one non-randomized controlled trial were identified, and all eight RCTs found no significant findings. The non-randomized controlled trial included

interventions with calcium, vitamin D, or calcium plus vitamin D supplementation and reported rickets in <2% of the study population.[50] While results showed no difference in rickets incidence by supplementation ($P = 0.214$), there was a significant interaction for time and supplementation over the 3-year study period ($P = 0.001$).

- o For bone mineral content and density (BMC/BMD) outcomes, nine RCTs and one non-randomized controlled trial were identified and these reported mixed results. Five RCTs plus one three-year follow-up reported no difference in outcomes between any study groups.[29,38,39,43,56,57] Two studies reported benefits to BMC/BMD outcomes for vitamin D supplementation vs. placebo but did not report p-values or confidence intervals.[24,37] Two studies reported significant benefits for BMC/BMD when comparing higher vs. lower doses of vitamin D (1,600 IU/d vs. 400 IU/d; 1,600 IU/d vs. 1,200 IU/d)[35] or when comparing vitamin D supplementation with human milk alone.[55] One study reported moderately significant ($0.05 < P < 0.1$) benefits for one bone measurement when comparing vitamin D supplementation to a placebo.[48]

- Evidence is **insufficient** on the effect of different levels of vitamin D intake on blood pressure, as only one RCT was identified that assessed this outcome.
- Evidence is **insufficient** on the effect of different levels of vitamin D intake on autoimmune disease and fracture, as no interventional studies were identified that assessed these outcomes.

KQ2. What is the association between serum 25(OH)D concentrations and health outcomes in children aged 0 to 4 years?

- Evidence is **very low** on the association between serum 25(OH)D concentration and several health outcomes including atopic outcomes (i.e., asthma, wheeze, eczema), autoimmune diseases, and infectious diseases.
  - o For asthma, three cohort studies reported mixed results with two cohorts finding no association[61,62] and one cohort reporting that lower 25(OH)D concentrations were associated with increased risk of non-medicated asthma.[63] Wheeze and eczema outcomes also had mixed results. For wheezing, one cohort study found no association,[62] while another reported lower 25(OH)D concentrations being associated with increased risk of wheezing.[63] For eczema, one cohort found no association,[60] while another reported lower 25(OH)D concentrations being associated with increased risk of eczema.[63]
  - o For autoimmune disease outcomes, seven studies reporting on three outcomes (i.e., type 1 diabetes, islet autoimmunity, and JIA) were identified. For type 1 diabetes, four studies from three publications found no association.[66-68] For islet autoimmunity, one case-cohort found no association,[70] while a nested case-control study found high serum 25(OH)D in the first year of life and in childhood associated with decreased risk of islet autoimmunity.[69] For JIA, one case-cohort study found no association.[71]
  - o For infectious disease outcomes, four cohort studies were identified which reported on eight infectious diseases. Higher serum 25(OH)D was associated with three of the eight outcomes: decreased risk of oral candidiasis,[65] increased risk for URTI (in underweight children),[64] and increased risk of malaria infection (between highest and second highest quartiles of serum 25OHD).[65]
- Evidence is **low** on the association between serum 25(OH)D concentration and growth and neurodevelopment in children 0 to 4 years of age.
  - o For growth and neurological development outcomes, six observational studies, including four cohorts and two case-control studies, were identified. No linear associations were

found between serum 25(OH)D and these outcomes; however, when serum 25(OH)D was analyzed as a categorical exposure, one study found higher 25(OH)D levels were positively associated with IQ scores at age 19,[74] and another study showed significantly positive associations with weight-for-length z scores for serum 25(OH)D levels of 20-29.9 ng/mL compared to < 10 ng/mL.[65]

- Evidence is **insufficient** on the association between serum 25(OH)D concentration and fracture, as only one case-cohort study was identified that assessed the association.
- Evidence is **insufficient** on the association between serum 25(OH)D concentration and blood pressure, as only one cohort study was identified that assessed the association.

KQ3. What is the effect of vitamin D intake on serum 25(OH)D concentrations in children aged 0 to 4 years?

- Evidence is **moderate** on the effect of vitamin D intake on serum 25(OH)D concentration in children aged 0 to 4 years.
    - o Thirty trials (reported in 31 publications) conducted in children aged 0-12 months, and one RCT in children aged 1 to 4 years, were identified. A random effects meta-regression analysis of these age groups combined showed that each 100 IU/d increase in vitamin D supplementation was associated with an average of 1.92 (95% CI 0.28, 3.56) nmol/L increase in achieved 25(OH)D concentration (n = 53 intervention arms; $P = 0.022$; adjusted $R^2 = 9.07\%$).
    - o Seven studies were identified in children aged 3-9 years old, and a random-effects meta-regression for this age group showed that each 100 IU/d increase in vitamin D supplementation was associated with an average of 2.49 (95% CI -0.24, 5.22) nmol/L increase in achieved 25(OH)D concentration (n = 16 intervention arms; $P = 0.071$; adjusted $R^2 = 19.96\%$).

Vitamin D Upper Limits

ULs KQ1a. At what levels of vitamin D intake are adverse effects observed in children aged 0 to 4 years?

- Evidence is **very low** on the association between vitamin D intake or serum 25(OH)D and two upper limit outcomes, hypercalcemia and hypercalciuria. Generally, the rate of hypercalcemia increased with dose of vitamin D; however, no significant differences between groups were reported, and studies were graded as inconsistent and imprecise. The rate of hypercalciuria was variable among studies and intervention arms.
- Evidence is **insufficient** on the association between vitamin D intake or serum 25(OH)D and other upper limit outcomes (including nephrocalcinosis, kidney stones, and mortality) due to limited high-quality studies assessing the outcomes.

ULs KQ1b. What are levels of vitamin D intake at which a prespecified threshold of serum 25(OH)D is reached in children aged 0 to 4 years?

- The levels of vitamin D intake at which prespecified thresholds of serum 25(OH)D is reached in children aged 0 to 4 years was considered in studies with daily dose interventions (n = 21), single and intermittent large dose interventions (n = 6), and food and fortified food interventions (n = 2). It is difficult to draw a conclusion since the included studies reported the percentage of participants achieving various prespecified serum 25(OH)D thresholds as the outcome, and the

percentage of participants reaching the prespecified thresholds was variable and may have depended on the threshold level, intervention dose, and intervention duration.

References

1. World Health Organization. FAO/WHO nutrient requirements for children aged 0–36 months. https://apps.who.int/nutrition/topics/nutrient-requirements-children-overview/en/index.html. Published 2020. Accessed June 6, 2022.
2. Chung M, Ruan M, Cara KC, Yao Q, Penkert LP, Chen J. Vitamin D and Calcium in Children 0-36 Months: A Scoping Review of Health Outcomes. *J Am Coll Nutr.* 2021;40(4):367-396.
3. Russell R, Chung M, Balk EM, et al. Opportunities and challenges in conducting systematic reviews to support the development of nutrient reference values: vitamin A as an example. *Am J Clin Nutr.* 2009;89(3):728-733.
4. Chung M, Balk EM, Brendel M, et al. Vitamin D and calcium: a systematic review of health outcomes. *Evid Rep Technol Assess (Full Rep).* 2009(183):1-420.
5. Yetley EA, MacFarlane AJ, Greene-Finestone LS, et al. Options for basing Dietary Reference Intakes (DRIs) on chronic disease endpoints: report from a joint US-/Canadian-sponsored working group. *Am J Clin Nutr.* 2017;105(1):249S-285S.
6. National Academies of Sciences E, and Medicine. *Harmonization of Approaches to Nutrient Reference Values: Applications to Young Children and Women of Reproductive Age.* Washington (DC): The National Academies Press;2018. 9780309477697

0309477697.

7. Holden JM, Lemar LE. Assessing vitamin D contents in foods and supplements: challenges and needs. *Am J Clin Nutr.* 2008;88(2):551S-553S.
8. In: Eden J, Levit L, Berg A, Morton S, eds. *Finding What Works in Health Care: Standards for Systematic Reviews.* Washington (DC)2011.
9. Moher D, Liberati A, Tetzlaff J, Altman DG, Group P. Preferred reporting items for systematic reviews and meta-analyses: the PRISMA statement. *PLoS Med.* 2009;6(7):e1000097.
10. Huey SL, Acharya N, Silver A, et al. Effects of oral vitamin D supplementation on linear growth and other health outcomes among children under five years of age. *Cochrane Database Syst Rev.* 2020;12:CD012875.
11. Ouzzani M, Hammady H, Fedorowicz Z, Elmagarmid A. Rayyan-a web and mobile app for systematic reviews. *Syst Rev.* 2016;5(1):210.
12. Sterne JAC, Savovic J, Page MJ, et al. RoB 2: a revised tool for assessing risk of bias in randomised trials. *BMJ.* 2019;366:l4898.
13. Wells G, Shea B, O'Connell D, et al. The Newcastle-Ottawa Scale (NOS) for assessing the quality of nonrandomised studies in meta-analyses. http://www.ohri.ca/programs/clinical_epidemiology/oxford.asp. Published 2013. Accessed June 27, 2020.
14. Lichtenstein AH, Yetley EA, Lau J. Application of systematic review methodology to the field of nutrition. *J Nutr.* 2008;138(12):2297-2306.
15. Johansson ALV. The Case-Cohort design: What it is and how it can be used in register-based research. Stata. https://www.stata.com/meeting/nordic-and-baltic16/slides/norway16_johansson.pdf. Published 2016. Accessed.
16. Barlow WE, Ichikawa L, Rosner D, Izumi S. Analysis of case-cohort designs. *J Clin Epidemiol.* 1999;52(12):1165-1172.
17. Breslow NE, Lumley T, Ballantyne CM, Chambless LE, Kulich M. Using the whole cohort in the analysis of case-cohort data. *Am J Epidemiol.* 2009;169(11):1398-1405.

18. Kim RS. Analysis of Nested Case-Control Study Designs: Revisiting the Inverse Probability Weighting Method. *Commun Stat Appl Methods.* 2013;20(6):455-466.
19. Guyatt GH, Oxman AD, Kunz R, et al. What is "quality of evidence" and why is it important to clinicians? *BMJ.* 2008;336(7651):995-998.
20. Guyatt GH, Oxman AD, Vist GE, et al. GRADE: an emerging consensus on rating quality of evidence and strength of recommendations. *BMJ.* 2008;336(7650):924-926.
21. Guyatt G, Oxman AD, Akl EA, et al. GRADE guidelines: 1. Introduction-GRADE evidence profiles and summary of findings tables. *J Clin Epidemiol.* 2011;64(4):383-394.
22. Harbord R, Higgins J. Meta-Regression in Stata. *Stata Journal.* 2008;8:493-519.
23. Jackson D, White IR, Riley RD. A matrix-based method of moments for fitting the multivariate random effects model for meta-analysis and meta-regression. *Biom J.* 2013;55(2):231-245.
24. Hibbs AM, Ross K, Kerns LA, et al. Effect of Vitamin D Supplementation on Recurrent Wheezing in Black Infants Who Were Born Preterm: The D-Wheeze Randomized Clinical Trial. *Jama.* 2018;319(20):2086-2094.
25. Rosendahl J, Pelkonen AS, Helve O, et al. High-Dose Vitamin D Supplementation Does Not Prevent Allergic Sensitization of Infants. *J Pediatr.* 2019;209:139-145 e131.
26. Grant CC, Crane J, Mitchell EA, et al. Vitamin D supplementation during pregnancy and infancy reduces aeroallergen sensitization: a randomized controlled trial. *Allergy.* 2016;71(9):1325-1334.
27. Rueter K, Jones AP, Siafarikas A, et al. Direct infant UV light exposure is associated with eczema and immune development. *J Allergy Clin Immunol.* 2019;143(3):1012-1020 e1012.
28. Manaseki-Holland S, Maroof Z, Bruce J, et al. Effect on the incidence of pneumonia of vitamin D supplementation by quarterly bolus dose to infants in Kabul: a randomised controlled superiority trial. *Lancet.* 2012;379(9824):1419-1427.
29. Rosendahl J, Valkama S, Holmlund-Suila E, et al. Effect of Higher vs Standard Dosage of Vitamin D3 Supplementation on Bone Strength and Infection in Healthy Infants: A Randomized Clinical Trial. *JAMA Pediatr.* 2018;172(7):646-654.
30. Grant CC, Kaur S, Waymouth E, et al. Reduced primary care respiratory infection visits following pregnancy and infancy vitamin D supplementation: a randomised controlled trial. *Acta Paediatr.* 2015;104(4):396-404.
31. Zhou J, Du J, Huang L, Wang Y, Shi Y, Lin H. Preventive Effects of Vitamin D on Seasonal Influenza A in Infants: A Multicenter, Randomized, Open, Controlled Clinical Trial. *Pediatr Infect Dis J.* 2018;37(8):749-754.
32. Aglipay M, Birken CS, Parkin PC, et al. Effect of High-Dose vs Standard-Dose Wintertime Vitamin D Supplementation on Viral Upper Respiratory Tract Infections in Young Healthy Children. *Jama.* 2017;318(3):245-254.
33. Alonso A, Rodriguez J, Carvajal I, et al. Prophylactic vitamin D in healthy infants: assessing the need. *Metabolism.* 2011;60(12):1719-1725.
34. Enlund-Cerullo M, Koljonen L, Holmlund-Suila E, et al. Genetic Variation of the Vitamin D Binding Protein Affects Vitamin D Status and Response to Supplementation in Infants. *J Clin Endocrinol Metab.* 2019;104(11):5483-5498.
35. Holmlund-Suila E, Viljakainen H, Hytinantti T, Lamberg-Allardt C, Andersson S, Makitie O. High-dose vitamin d intervention in infants--effects on vitamin d status, calcium homeostasis, and bone strength. *J Clin Endocrinol Metab.* 2012;97(11):4139-4147.
36. Huynh J, Lu T, Liew D, et al. Vitamin D in newborns. A randomised controlled trial comparing daily and single oral bolus vitamin D in infants. *J Paediatr Child Health.* 2017;53(2):163-169.

37. Greer FR, Searcy JE, Levin RS, Steichen JJ, Steichen-Asche PS, Tsang RC. Bone mineral content and serum 25-hydroxyvitamin D concentrations in breast-fed infants with and without supplemental vitamin D: one-year follow-up. *J Pediatr.* 1982;100(6):919-922.
38. Gallo S, Comeau K, Vanstone C, et al. Effect of different dosages of oral vitamin D supplementation on vitamin D status in healthy, breastfed infants: a randomized trial. *Jama.* 2013;309(17):1785-1792.
39. Natarajan CK, Sankar MJ, Agarwal R, et al. Trial of daily vitamin D supplementation in preterm infants. *Pediatrics.* 2014;133(3):e628-634.
40. Ziegler EE, Nelson SE, Jeter JM. Vitamin D supplementation of breastfed infants: a randomized dose-response trial. *Pediatr Res.* 2014;76(2):177-183.
41. Wagner CL, Hulsey TC, Fanning D, Ebeling M, Hollis BW. High-dose vitamin D3 supplementation in a cohort of breastfeeding mothers and their infants: a 6-month follow-up pilot study. *Breastfeed Med.* 2006;1(2):59-70.
42. Kumar GT, Sachdev HS, Chellani H, et al. Effect of weekly vitamin D supplements on mortality, morbidity, and growth of low birthweight term infants in India up to age 6 months: randomised controlled trial. *BMJ.* 2011;342:d2975.
43. Chan GM, Roberts CC, Folland D, Jackson R. Growth and bone mineralization of normal breast-fed infants and the effects of lactation on maternal bone mineral status. *Am J Clin Nutr.* 1982;36(3):438-443.
44. Singh H, Maria A, Shukla A, Sharma N, Mani K, Das MK. Relation of Vitamin D supplementation, upto six months of age with total and bone specifc alkaline phosphatase: a randomised control trial. *Journal of clinical and diagnostic research.* 2018;12(10):SC01-SC05.
45. Chandy DD, Kare J, Singh SN, et al. Effect of vitamin D supplementation, directly or via breast milk for term infants, on serum 25 hydroxyvitamin D and related biochemistry, and propensity to infection: a randomised placebo-controlled trial. *Br J Nutr.* 2016;116(1):52-58.
46. Dawodu A, Salameh KM, Al-Janahi NS, Bener A, Elkum N. The Effect of High-Dose Postpartum Maternal Vitamin D Supplementation Alone Compared with Maternal Plus Infant Vitamin D Supplementation in Breastfeeding Infants in a High-Risk Population. A Randomized Controlled Trial. *Nutrients.* 2019;11(7):17.
47. Wicklow B, Gallo S, Majnemer A, et al. Impact of Vitamin D Supplementation on Gross Motor Development of Healthy Term Infants: A Randomized Dose-Response Trial. *Phys Occup Ther Pediatr.* 2016;36(3):330-342.
48. Trilok-Kumar G, Kaur M, Rehman AM, et al. Effects of vitamin D supplementation in infancy on growth, bone parameters, body composition and gross motor development at age 3-6 years: follow-up of a randomized controlled trial. *Int J Epidemiol.* 2015;44(3):894-905.
49. Hazell TJ, Gallo S, Vanstone CA, Agellon S, Rodd C, Weiler HA. Vitamin D supplementation trial in infancy: body composition effects at 3 years of age in a prospective follow-up study from Montreal. *Pediatr Obes.* 2017;12(1):38-47.
50. Rooze S, Mathieu F, Claus W, et al. Effect of calcium and vitamin D on growth, rickets and Kashin-Beck disease in 0- to 5-year-old children in a rural area of central Tibet. *Trop Med Int Health.* 2016;21(6):768-775.
51. Ala-Houhala M. 25-Hydroxyvitamin D levels during breast-feeding with or without maternal or infantile supplementation of vitamin D. *J Pediatr Gastroenterol Nutr.* 1985;4(2):220-226.
52. Siafarikas A, Piazena H, Feister U, Bulsara MK, Meffert H, Hesse V. Randomised controlled trial analysing supplementation with 250 versus 500 units of vitamin D3, sun exposure and surrounding factors in breastfed infants. *Arch Dis Child.* 2011;96(1):91-95.

53. Ponnapakkam T, Bradford E, Gensure R. A treatment trial of vitamin D supplementation in breast-fed infants: universal supplementation is not necessary for rickets prevention in Southern Louisiana. *Clin Pediatr (Phila).* 2010;49(11):1053-1060.
54. Alizade P, Naderi F, Sotoudeh K. A randomized clinical trial of prophylactic effects of vitamin D on different indices of osteopenia of prematurity. *Iranian Journal of Public Health.* 2006;35(3):58-63.
55. Bagnoli F, Casucci M, Toti S, et al. Is vitamin D supplementation necessary in healthy full-term breastfed infants? A follow-up study of bone mineralization in healthy full-term infants with and without supplemental vitamin D. *Minerva Pediatr.* 2013;65(3):253-260.
56. Rao YK, Midha T, Singh S, Bajpai A, Tilak A. Increment in vitamin D level and bone mineral accrual in children with vitamin D deficiency. *Korean J Pediatr.* 2016;59(7):292-297.
57. Gallo S, Hazell T, Vanstone CA, et al. Vitamin D supplementation in breastfed infants from Montreal, Canada: 25-hydroxyvitamin D and bone health effects from a follow-up study at 3 years of age. *Osteoporos Int.* 2016;27(8):2459-2466.
58. Zhang H, Liu S, Si Y, et al. Natural sunlight plus vitamin D supplementation ameliorate delayed early motor development in newborn infants from maternal perinatal depression. *J Affect Disord.* 2019;257:241-249.
59. Ho ML, Yen HC, Tsang RC, Specker BL, Chen XC, Nichols BL. Randomized study of sunshine exposure and serum 25-OHD in breast-fed infants in Beijing, China. *J Pediatr.* 1985;107(6):928-931.
60. Molloy J, Koplin JJ, Allen KJ, et al. Vitamin D insufficiency in the first 6 months of infancy and challenge-proven IgE-mediated food allergy at 1 year of age: a case-cohort study. *Allergy.* 2017;72(8):1222-1231.
61. Navas-Nazario A, Fangyong L, Northrup V, et al. Effect of vitamin D binding protein (DBP) genotype on the development of asthma in young children. *American Journal of Respiratory and Critical Care Medicine.* 2011;183(1).
62. Anderson LN, Chen Y, Omand JA, et al. Vitamin D exposure during pregnancy, but not early childhood, is associated with risk of childhood wheezing. *J Dev Orig Health Dis.* 2015;6(4):308-316.
63. Hollams EM, Teo SM, Kusel M, et al. Vitamin D over the first decade and susceptibility to childhood allergy and asthma. *J Allergy Clin Immunol.* 2017;139(2):472-481 e479.
64. Ahmed AMS, Ahmed T, Soares Magalhaes RJ, et al. Association between serum Vitamin D, retinol and zinc status, and acute respiratory infections in underweight and normal-weight children aged 6-24 months living in an urban slum in Bangladesh. *Epidemiology and Infection.* 2016;144(16):3494-3506.
65. Sudfeld CR, Duggan C, Aboud S, et al. Vitamin D status is associated with mortality, morbidity, and growth failure among a prospective cohort of HIV-infected and HIV-exposed Tanzanian infants. *J Nutr.* 2015;145(1):121-127.
66. Cadario F, Savastio S, Pagliardini V, et al. Vitamin D levels at birth and risk of type 1 diabetes in childhood: a case-control study. *Acta Diabetol.* 2015;52(6):1077-1081.
67. Jacobsen R, Thorsen SU, Cohen AS, et al. Neonatal vitamin D status is not associated with later risk of type 1 diabetes: results from two large Danish population-based studies. *Diabetologia.* 2016;59(9):1871-1881.
68. Makinen M, Mykkanen J, Koskinen M, et al. Serum 25-Hydroxyvitamin D Concentrations in Children Progressing to Autoimmunity and Clinical Type 1 Diabetes. *J Clin Endocrinol Metab.* 2016;101(2):723-729.

69. Norris JM, Lee HS, Frederiksen B, et al. Plasma 25-Hydroxyvitamin D Concentration and Risk of Islet Autoimmunity. *Diabetes.* 2018;67(1):146-154.
70. Simpson M, Brady H, Yin X, et al. No association of vitamin D intake or 25-hydroxyvitamin D levels in childhood with risk of islet autoimmunity and type 1 diabetes: the Diabetes Autoimmunity Study in the Young (DAISY). *Diabetologia.* 2011;54(11):2779-2788.
71. Thorsen SU, Pipper CB, Alberdi-Saugstrup M, et al. No association between vitamin D levels around time of birth and later risk of developing oligo- and polyarticular juvenile idiopathic arthritis: a Danish case-cohort study. *Scand J Rheumatol.* 2017;46(2):104-111.
72. Chowdhury R, Taneja S, Bhandari N, Kvestad I, Strand TA, Bhan MK. Vitamin-D status and neurodevelopment and growth in young north Indian children: a secondary data analysis. *Nutr J.* 2017;16(1):59.
73. Pludowski P, Jaworski M, Niemirska A, et al. Vitamin D status, body composition and hypertensive target organ damage in primary hypertension. *J Steroid Biochem Mol Biol.* 2014;144 Pt A:180-184.
74. Specht IO, Janbek J, Thorsteinsdottir F, Frederiksen P, Heitmann BL. Neonatal vitamin D levels and cognitive ability in young adulthood. *Eur J Nutr.* 2020;59(5):1919-1928.
75. Wu DM, Wen X, Han XR, et al. Relationship Between Neonatal Vitamin D at Birth and Risk of Autism Spectrum Disorders: the NBSIB Study. *J Bone Miner Res.* 2018;33(3):458-466.
76. Handel MN, Frederiksen P, Cohen A, Cooper C, Heitmann BL, Abrahamsen B. Neonatal vitamin D status from archived dried blood spots and future risk of fractures in childhood: results from the D-tect study, a population-based case-cohort study. *Am J Clin Nutr.* 2017;106(1):155-161.
77. Tornhammar P, Ueda P, Hult M, Simila H, Eyles D, Norman M. Season of birth, neonatal vitamin D status, and cardiovascular disease risk at 35 y of age: a cohort study from Sweden. *Am J Clin Nutr.* 2014;99(3):472-478.
78. Ala-Houhala M, Koskinen T, Terho A, Koivula T, Visakorpi J. Maternal compared with infant vitamin D supplementation. *Arch Dis Child.* 1986;61(12):1159-1163.
79. Aluisio AR, Maroof Z, Chandramohan D, et al. Vitamin D(3)supplementation and childhood diarrhea: a randomized controlled trial. *Pediatrics.* 2013;132(4):e832-840.
80. Atas E, Karademir F, Ersen A, et al. Comparison between daily supplementation doses of 200 versus 400 IU of vitamin D in infants. *Eur J Pediatr.* 2013;172(8):1039-1042.
81. Ekbote VH, Khadilkar AV, Chiplonkar SA, Hanumante NM, Khadilkar VV, Mughal MZ. A pilot randomized controlled trial of oral calcium and vitamin D supplementation using fortified laddoos in underprivileged Indian toddlers. *Eur J Clin Nutr.* 2011;65(4):440-446.
82. Gallo S, Phan A, Vanstone CA, Rodd C, Weiler HA. The change in plasma 25-hydroxyvitamin D did not differ between breast-fed infants that received a daily supplement of ergocalciferol or cholecalciferol for 3 months. *J Nutr.* 2013;143(2):148-153.
83. Gordon CM, Williams AL, Feldman HA, et al. Treatment of hypovitaminosis D in infants and toddlers. *J Clin Endocrinol Metab.* 2008;93(7):2716-2721.
84. Grant CC, Stewart AW, Scragg R, et al. Vitamin D during pregnancy and infancy and infant serum 25-hydroxyvitamin D concentration. *Pediatrics.* 2014;133(1):e143-153.
85. Harnot J, Verma S, Singhi S, Sankhyan N, Sachdeva N, Bharti B. Comparison of 300,000 and 600,000 IU Oral Vitamin-D Bolus for Vitamin-D Deficiency in Young Children. *Indian J Pediatr.* 2017;84(2):111-116.
86. Hollis BW, Wagner CL, Howard CR, et al. Maternal Versus Infant Vitamin D Supplementation During Lactation: A Randomized Controlled Trial. *Pediatrics.* 2015;136(4):625-634.

87. Holstgemeiner D, Gemeiner M, Pilz I, Swoboda W. PLASMA 25-HYDROXYCHOLECALCIFEROL AFTER DAILY VITAMIN-D ADMINISTRATION IN COMPARISON WITH MASSIVE SINGLE-DOSE PROPHYLAXIS. *Wiener Klinische Wochenschrift.* 1978;90(14):509-512.
88. Kunz C, von Lilienfeld-Toal H, Niesen M, Burmeister W. [25-hydroxy-vitamin-D in serum of newborns and infants during continuous oral vitamin D treatment (author's transl)]. *Padiatr Padol.* 1982;17(2):181-185.
89. Madar AA, Klepp KI, Meyer HE. Effect of free vitamin D(2) drops on serum 25-hydroxyvitamin D in infants with immigrant origin: a cluster randomized controlled trial. *Eur J Clin Nutr.* 2009;63(4):478-484.
90. Moodley A, Spector SA. Single high-dose vitamin D at birth corrects vitamin D deficiency in infants in Mexico. *Int J Food Sci Nutr.* 2015;66(3):336-341.
91. Pittard WB, 3rd, Geddes KM, Hulsey TC, Hollis BW. How much vitamin D for neonates? *Am J Dis Child.* 1991;145(10):1147-1149.
92. Shajari A, Shakiba M, Nourani F, Zaki M, Kheirandish M. Urinary Calcium/Creatinin Ratio with Different Dosages of Vitamin D3 Prophylaxis in Infants. *Iranian Journal of Pediatrics.* 2009;19(2):159-163.
93. Shakiba M, Sadr S, Nefei Z, Mozaffari-Khosravi H, Lotfi MH, Bemanian MH. Combination of bolus dose vitamin D with routine vaccination in infants: a randomised trial. *Singapore Med J.* 2010;51(5):440-445.
94. Shakiba M, Pahloosye A, Mirouliaei M, Islami Z. Comparison of two regimens of vitamin D supplementation for vitamin D-deficient neonates. *Singapore Med J.* 2014;55(5):266-270.
95. Sharma AK, Gallo S, Vanstone CA, et al. Parathyroid hormone-ionized calcium dynamics over the first year of life. *J Pediatr Endocrinol Metab.* 2016;29(6):709-714.
96. Zeghoud F, Benmekhbi H, Djeghri N, Garabedian M. VITAMIN-D PROPHYLAXIS DURING INFANCY COMPARISON OF THE LONG-TERM EFFECTS OF 3 INTERMITTENT DOSES (15-MG, 5-MG, OR 2.5-MG) ON 25-HYDROXYVITAMIN-D CONCENTRATIONS. *American Journal of Clinical Nutrition.* 1994;60(3):393-396.
97. Zeghoud F, Vervel C, Guillozo H, Walrant-Debray O, Boutignon H, Garabedian M. Subclinical vitamin D deficiency in neonates: definition and response to vitamin D supplements. *Am J Clin Nutr.* 1997;65(3):771-778.
98. Greer FR, Searcy JE, Levin RS, Steichen JJ, Asch PS, Tsang RC. Bone mineral content and serum 25-hydroxyvitamin D concentration in breast-fed infants with and without supplemental vitamin D. *The Journal of Pediatrics.* 1981;98(5):696-701.
99. Mittal H, Rai S, Shah D, et al. 300,000 IU or 600,000 IU of oral vitamin D3 for treatment of nutritional rickets: a randomized controlled trial. *Indian Pediatr.* 2014;51(4):265-272.
100. Mittal M, Yadav V, Khadgawat R, Kumar M, Sherwani P. Efficacy and Safety of 90,000 IU versus 300,000 IU Single Dose Oral Vitamin D in Nutritional Rickets: A Randomized Controlled Trial. *Indian J Endocrinol Metab.* 2018;22(6):760-765.
101. Abrams SA, Hawthorne KM, Chen Z. Supplementation with 1000 IU vitamin D/d leads to parathyroid hormone suppression, but not increased fractional calcium absorption, in 4-8-y-old children: a double-blind randomized controlled trial. *Am J Clin Nutr.* 2013;97(1):217-223.
102. Alahouhala M, Koskinen T, Koskinen M, Visakorpi JK. DOUBLE-BLIND STUDY ON THE NEED FOR VITAMIN-D SUPPLEMENTATION IN PREPUBERTAL CHILDREN. *Acta Paediatrica Scandinavica.* 1988;77(1):89-93.

103. Brett NR, Lavery P, Agellon S, et al. Dietary vitamin D dose-response in healthy children 2 to 8 y of age: a 12-wk randomized controlled trial using fortified foods. *Am J Clin Nutr.* 2016;103(1):144-152.
104. Brett NR, Parks CA, Lavery P, et al. Vitamin D status and functional health outcomes in children aged 2-8 y: a 6-mo vitamin D randomized controlled trial. *Am J Clin Nutr.* 2018;107(3):355-364.
105. Economos CD, Moore CE, Hyatt RR, et al. Multinutrient-fortified juices improve vitamin D and vitamin E status in children: a randomized controlled trial. *J Acad Nutr Diet.* 2014;114(5):709-717.
106. Hirschler V, Maccallini G, Tamborenea MI, et al. Improvement in lipid profile after vitamin D supplementation in indigenous argentine school children. *Cardiovasc Hematol Agents Med Chem.* 2014;12(1):42-49.
107. Karlsland Akeson P, Akesson KE, Lind T, Hernell O, Silfverdal SA, Ohlund I. Vitamin D Intervention and Bone: A Randomized Clinical Trial in Fair- and Dark-skinned Children at Northern Latitudes. *J Pediatr Gastroenterol Nutr.* 2018;67(3):388-394.
108. Loeb M, Dang AD, Thiem VD, et al. Effect of Vitamin D supplementation to reduce respiratory infections in children and adolescents in Vietnam: A randomized controlled trial. *Influenza Other Respir Viruses.* 2019;13(2):176-183.
109. Mandlik RM, Mughal ZM, Khadilkar AV, et al. Paradoxical Response of Parathyroid Hormone to Vitamin Da[Euro sign]"Calcium Supplementation in Indian Children. *J Pediatr.* 2019.
110. Marwaha RK, Mithal A, Bhari N, et al. Supplementation with Three Different Daily Doses of Vitamin D3 in Healthy Pre-pubertal School Girls: A Cluster Randomized Trial. *Indian Pediatr.* 2018;55(11):951-956.
111. Mortensen C, Damsgaard CT, Hauger H, et al. Estimation of the dietary requirement for vitamin D in white children aged 4-8 y: a randomized, controlled, dose-response trial. *Am J Clin Nutr.* 2016;104(5):1310-1317.
112. Ohlund I, Lind T, Hernell O, Silfverdal SA, Karlsland Akeson P. Increased vitamin D intake differentiated according to skin color is needed to meet requirements in young Swedish children during winter: a double-blind randomized clinical trial. *Am J Clin Nutr.* 2017;106(1):105-112.
113. Stellinga-Boelen AA, Wiegersma PA, Storm H, Bijleveld CM, Verkade HJ. Vitamin D levels in children of asylum seekers in The Netherlands in relation to season and dietary intake. *Eur J Pediatr.* 2007;166(3):201-206.
114. Talaat IM, Kamal NM, Alghamdi HA, Alharthi AA, Alshahrani MA. A randomized clinical trial comparing 3 different replacement regimens of vitamin D in clinically asymptomatic pediatrics and adolescents with vitamin D insufficiency. *Ital J Pediatr.* 2016;42(1):106.
115. Mandlik R, Mughal Z, Khadilkar A, et al. Occurrence of infections in schoolchildren subsequent to supplementation with vitamin D-calcium or zinc: a randomized, double-blind, placebo-controlled trial. *Nutr Res Pract.* 2020;14(2):117-126.
116. Valkama S, Holmlund-Suila E, Enlund-Cerullo M, et al. No Severe Hypercalcemia with Daily Vitamin D3 Supplementation of up to 30 microg during the First Year of Life. *Horm Res Paediatr.* 2017;88(2):147-154.
117. Markestad T, Hesse V, Siebenhuner M, et al. Intermittent high-dose vitamin D prophylaxis during infancy: effect on vitamin D metabolites, calcium, and phosphorus. *Am J Clin Nutr.* 1987;46(4):652-658.
118. Oliveri B, Cassinelli H, Mautalen C, Ayala M. Vitamin D prophylaxis in children with a single dose of 150000 IU of vitamin D. *Eur J Clin Nutr.* 1996;50(12):807-810.

119. Sezer RG, Guran T, Paketci C, Seren LP, Bozaykut A, Bereket A. Comparison of oral alendronate versus prednisolone in treatment of infants with vitamin D intoxication. *Acta Paediatr.* 2012;101(3):e122-125.
120. Jensen CB, Lundqvist M, Sorensen TIA, Heitmann BL. Neonatal Vitamin D Levels in Relation to Risk of Overweight at 7 Years in the Danish D-Tect Case-Cohort Study. *Obes Facts.* 2017;10(3):273-283.
121. Lee SH, Kim SM, Park HS, et al. Serum 25-hydroxyvitamin D levels, obesity and the metabolic syndrome among Korean children. *Nutr Metab Cardiovasc Dis.* 2013;23(8):785-791.
122. Demir K, Doneray H, Kara C, et al. Comparison of Treatment Regimens in Management of Severe Hypercalcemia Due to Vitamin D Intoxication in Children. *J Clin Res Pediatr Endocrinol.* 2019;11(2):140-148.
123. Gurkan F, Davutoglu M, Bosnak M, et al. Pamidronate treatment in acute vitamin D intoxication. *J Endocrinol Invest.* 2004;27(7):680-682.
124. Vanstone MB, Oberfield SE, Shader L, Ardeshirpour L, Carpenter TO. Hypercalcemia in children receiving pharmacologic doses of vitamin D. *Pediatrics.* 2012;129(4):e1060-1063.
125. Andersen B. [Vitamin D poisoning]. *Tidsskr Nor Laegeforen.* 1954;74(12):419-420.
126. Barrueto F, Jr., Wang-Flores HH, Howland MA, Hoffman RS, Nelson LS. Acute vitamin D intoxication in a child. *Pediatrics.* 2005;116(3):e453-456.
127. Elarqam L, Babakhouya A, Chaouki S, Atmani S, Bouharrou A, Hida M. L'intoxication par la vitamine D chez le nourrisson. *Journal de Pédiatrie et de Puériculture.* 2007;20(5):203-205.
128. Evliyaoglu O, Berberoglu M, Ocal G, Adiyaman P, Aycan Z. Severe hypercalcemia of an infant due to vitamin D toxicity associated with hypercholesterolemia. *J Pediatr Endocrinol Metab.* 2001;14(7):915-919.
129. Garbim BB, L DA, Rigatto SZP, Quadros K, Belangero VMS, Oliveira RB. Hypercalcemia in children: three cases report with unusual clinical presentations. *J.* 2017;39(2):213-216.
130. Hoppe B, Gnehm HE, Wopmann M, Neuhaus T, Willi U, Leumann E. [Vitamin D poisoning in infants: a preventable cause of hypercalciuria and nephrocalcinosis]. *Schweiz Med Wochenschr.* 1992;122(8):257-262.
131. Misselwitz J, Hesse V. [Hypercalcemia following prophylactic vitamin D administration]. *Kinderarztl Prax.* 1986;54(8):431-438.
132. Otto-Buczkowska E, Kokot F. [Hypercalcemia after oral administration of preventive doses of vitamin D]. *Pol Tyg Lek.* 1982;37(32):943-945.
133. Ross SG. Vitamin D intoxication in infancy; a report of four cases. *J Pediatr.* 1952;41(6):815-822.
134. Ünal E, Koksal Y, Keles S, Artac H, Reisli I, Energin M. Iatrogenic vitamin D intoxiciation in infancy: Three cases. *Marmara Medical Journal.* 2007;20(1):47-51.
135. Besbas N, Oner A, Akhan O, Saatci U, Bakkaloglu A, Topaloglu R. Nephrocalcinosis due to vitamin D intoxication. *Turk J Pediatr.* 1989;31(3):239-244.
136. Nanulescu M, Manasia R. Hypercalcemia due to intoxication with vitamin D. *Pediatrie.* 1984;33(3):279-284.
137. Mortensen C, Molgaard C, Hauger H, Kristensen M, Damsgaard CT. Winter vitamin D3 supplementation does not increase muscle strength, but modulates the IGF-axis in young children. *Eur J Nutr.* 2019;58(3):1183-1192.

Appendix 1: GRADE Evidence Profile Tables

Vitamin D Requirements and Upper Limits

The effect of vitamin D intake on blood pressure and fracture outcomes were rated insufficient due to less than three unique studies meeting the inclusion criteria for this systematic review. Therefore, these outcomes were not included in the GRADE evidence profile table below.

GRADE evidence profile table: vitamin D requirements and upper limits

**Quality assessment**							**Summary of findings**	**Strength of evidence**
**No. of studies**	**Design**	**Limitations**	**Inconsistency**	**Indirectness**	**Imprecision**	**Dose-response**		
**KQ1. Atopic outcomes: asthma, wheeze, eczema**								
4	RCTs	**Some limitations**: 100% of trials have some or high ROB in at least 1 ROB domain, and 50% of trials have high or some ROB in 3 domains.	**No serious inconsistency**: Most trials reported no significant differences in atopic outcomes comparing higher to lower doses of Vit D supplementation.	**Direct**: Clinical outcomes.	**Imprecise**: Small number of events with large confidence intervals.	No dose-response is present.	**Asthma:** 3 RCTs reported mixed results comparing higher to lower doses of Vit D supplementation. Two RCTs found no significant differences between groups. One RCT found participants who received 400 IU/d of vitamin D3 were at lower risk of developing asthma at 6 months, compared to those who received placebo (RR = 0.055; 95% CI 0.003, 0.94), but there were no significant differences in the risk of asthma when comparing 800 IU/d of vitamin D_3 to placebo, or when comparing 800 IU/d to 400 IU/d of vitamin D_3 supplementation.[26]<br>**Wheeze:** 2 RCTs reported mixed results. One study reported no significant findings,[27] but another RCT in preterm Black infants, found significantly reduced risk of recurrent wheezing at 12 months with sustained vitamin D supplementation compared to diet-limited supplementation (adjusted RR = 0.62; 95% CI 0.44, 0.87; $P = 0.005$).[24]<br>**Eczema:** 3 RCTs found no significant differences between groups.	LOW
**KQ1. Infectious diseases**								

8	RCTs	**Some limitations**: 88% of trials have some or high ROB in at least 2 ROB domains. The other trial (12%) has high risk in one ROB domain.	**No serious inconsistency**: Most trials reported no significant differences in infectious disease outcomes comparing higher to lower doses of vitamin D supplementati on	**Direct**: Clinical outcomes.	**Imprecise**: Studies reported variable effect measures with large confidence intervals.	No dose-response is present.	Out of the 20 infectious disease outcomes (respiratory, n = 15; gastrointestinal, n = 1; and other or unspecified infections, n = 4), 19 were not significantly different between intervention groups. One RCT found participants who received 1,200 IU/d of vitamin D_3 were significantly less likely to develop influenza A after 4 months compared to those receiving 400 IU/d of vitamin D_3 (RR = 0.54; 95% CI 0.42, 0.77).[31]	LOW
**KQ1. Growth and neurodevelopment**								
13	RCT (1 study with a non-randomized control group [37])	**Serious limitations:** All trials have some concern or high ROB in at least 2 domains, and 85% had high ROB for deviations from intended intervention. For all ROB domains, ≥ 30% of studies had	**Consistent:** 85% of studies showed no significant association between VD intervention and growth outcomes. Only 2 studies reported on neurological development outcomes.	**Direct:** Clinical outcome.	**Some imprecision:** Most studies reported small confidence intervals, but studies were not powered for development outcomes.	No dose-response is present.	Eleven RCTs reported no association between VD interventions and growth and development outcomes when comparing higher to lower doses or when comparing VD supplementation to a placebo.	LOW

		some concern or high ROB.						
**KQ1. Rickets**								
9	RCTs, non-randomized controlled trial	**Serious limitations:** All trials have some concern or high ROB in at least 3 domains. For all ROB domains, > 50% of studies had some concern or high ROB.	**Consistent:** 89% of trials reported no rickets, and 11% reported no significant association between rickets and VD supplements.	**Direct:** Clinical outcome.	**Imprecise:** Very small number of events.	No dose-response is present.	Eight RCTs reported no rickets. One non-randomized controlled trial reported rickets in <2% of the study population, and while there was no association with study arm (calcium, vitamin D, or calcium plus vitamin D supplementation) ($P = 0.214$), there was a significant interaction between time and supplementation over the three-year study period ($P = 0.001$).[50]	LOW
**KQ1. Bone mineral content and bone mineral density (BMC/BMD)**								
10	RCTs (1 study with a non-randomized control group [37]), non-randomized controlled trial	**Serious limitations:** All trials have some or high ROB in at least 1 ROB domain. For each ROB domain, except for measurement of outcome, at least 50% of studies have some or high ROB.	**Some inconsistency:** 50% of the studies reported no association between VD interventions and BMC/BMD outcomes. 30% reported benefits of VD supplementation vs. placebo for	**Indirect:** Surrogate outcome.	**Some imprecision:** 50% of studies with BMC/BMD as primary outcomes; 70% of studies with small sample sizes per study group;	Dose-response is present.	There were mixed results for BMC/BMD outcomes. Five RCTs from six publications reported no difference in BMC/BMD outcomes between any study groups.[29,38,39,43,56,57] Two studies reported benefits to BMC/BMD outcomes for VD supplementation vs. placebo but did not report p-values or confidence intervals, and one included a non-randomized comparison group.[24,37] Two studies reported statistically significant ($P<0.05$) benefits for various BMC/BMD measures when comparing randomized groups with higher vs. lower doses of VD (1,600 IU/d vs. 400 IU/d; 1,600 IU/d vs. 1,200 IU/d)[35] or when comparing non-randomized groups with VD supplementation or breast milk alone.[55] One study reported moderately significant ($0.05 < P < 0.1$) benefits for one bone measurement when comparing VD supplementation to a placebo.[48]	LOW

			BMC/BMD outcomes (0.05 < P<0.1, or p-values and 95% CI not reported). 20% reported significant associations between higher vs. lower VD doses or VD supplementation vs. breast milk alone and BMC/BMD outcomes.		Mostly narrow confidence intervals for BMD/BMC outcomes.			
**KQ2. Atopic outcomes: asthma, wheezing, and eczema**								
4	Cohorts, case-cohorts	**Serious limitations**: 63% of outcomes of interest were not demonstrated to be absent at start of study, 75% of outcomes were assessed by self-report, and 75% of	**No serious inconsistency**: Most studies reported no significant association between serum 25(OH)D and risk of atopic outcomes.	**Direct**: Clinical outcomes.	**Imprecise**: Wide confidence intervals.	No dose-response is present.	**Asthma:** Three cohort studies had mixed results measuring the association between serum 25(OH)D and asthma outcomes. Two cohort studies found no association. A third cohort study found participants with higher numbers of follow-up visits with deficient serum 25(OH)D had significantly increased risk of asthma, but not medicated asthma.[63]<br>**Wheeze:** Two cohort studies had mixed results measuring serum 25(OH)D and wheeze outcomes. One study found no association, while the other found participants with higher numbers of follow-up visits with deficient serum 25(OH)D had significantly increased risk of wheeze.[63]	VERY LOW

		outcomes had significant lost to follow-up					**Eczema:** Two studies had mixed results measuring serum 25(OH)D and eczema outcomes. One case-cohort study found no association, while the other cohort found participants with higher numbers of follow-up visits with deficient serum 25(OH)D had significantly increased risk of eczema.[63]	
**KQ2. Autoimmune diseases**								
7	Case-cohorts, nested case-controls	**Serious limitations**: 57% of studies with significant lost to follow-up or no statement, 71% not selecting all cases, and 71% using non-optimal or poorly described analytic methods	**No serious inconsistency**: Most studies reported no significant association between serum 25(OH)D and risk of autoimmune disease outcomes	**No serious indirectness**: Clinical outcomes or immediate precursor to clinical outcome (e.g., islet autoimmunity)	**Imprecise**: Most studies with wide confidence intervals or large measures of variability.	No dose-response is present.	**Type 1 diabetes:** Four observational studies found no association between serum vitamin D and type 1 diabetes.<br>**Islet autoimmunity:** Two observational studies reported mixed results. One case-cohort found no association between serum 25(OH)D and islet autoimmunity.[70] One nested case-control study found an association between serum 25(OH)D (in the first year of life and in childhood) and decreased risk of islet autoimmunity.[69]<br>**Juvenile idiopathic arthritis (JIA):** One case-cohort study found no association between serum 25(OH)D and oligoarticular and polyarticular JIA.[71]	VERY LOW
**KQ2. Infectious diseases**								
4	Cohorts	**Some limitations**: One study reporting one outcome (14% of outcomes) had major	**Some inconsistency**: Most studies found no significant association or an association between serum	**Direct**: Clinical outcomes.	**Imprecise**: Most studies with wide confidence intervals or large measures	No dose-response is present.	Most associations between serum 25(OH)D and infectious disease outcomes were not significant. Significant associations were found for three of eight total infectious disease outcomes, with higher serum 25(OH)D associated with a reduced risk of oral candidiasis[65] but an increased risk for URTI (in underweight children) and malaria infection (between	VERY LOW

		limitations: outcome assessed via self-report, outcome not demonstrated to be absent at start of study, analysis not optimally controlled, and poor adequacy of cohort follow-up	25(OH)D and decreased risk of infection, with one study reporting increased risk for one infectious disease outcome (oral candidiasis)		of variability.		highest and second highest quartiles of serum 25OHD).[64,65]	
**KQ2. Growth and neurological development**								
6	Cohorts (n=4), nested case-controls (n=2)	**Some limitations**: 83% of studies had ROB in at least 1 domain, and 50% had ROB in two or more domains. 50% reported high loss to follow-up rates or gave no statement.	**Consistent:** 100% of studies reported no significant linear association between 25(OH)D and growth and development outcomes.	**Direct:** Clinical outcome.	**Some imprecision:** Power calculations not reported for most studies, but most had large sample sizes; studies reported wide CIs	Dose response, but relationship with growth and development and neurological development appears to be non-linear.	In 6 observational studies assessing 25(OH)D levels and growth and development or neurological development outcomes, no linear association was found between 25(OH)D in infancy and any development outcomes. Categorical 25(OH)D analyses showed some statistically significant benefits in development outcomes with higher 25(OH)D levels compared to the lowest levels.	LOW

					or did not report CIs.			
**KQ3. Daily vitamin D supplementation on serum 25(OH)D**								
39	RCTs	**Some limitations:** In 4 of 5 ROB domains, greater than 50% of trials were assessed as having some or high ROB.	**No serious inconsistency:** Consistency in direction but some inconsistency in magnitude of the achieved 25(OH)D concentration at the end of the intervention period.	**Indirect**: serum 25(OH)D, a marker of vitamin D status.	**Some imprecision:** Meta-regression analysis demonstrated wide CIs. Also, the residual heterogeneity in meta-regressions was large.	Dose-response is present within most studies comparing different levels of daily vitamin D supplementation.	In infants 0-12 months old, random-effects meta-regression analysis showed that each 100 IU/d increase in vitamin D supplementation was associated with an average of 1.92 (95% CI 0.28, 3.56) nmol/L increase in achieved 25(OH)D concentration (n=53 intervention arms; *P*=0.022; adjusted R^2 = 9.07%). Only one study was in infants 1 to 4 years, which showed serum 25(OH)D unchanged in the 400 IU/d group but significantly increased from 89.6 to 121.6 nmol/L in the 2000 IU/d group after 16 weeks. In children 3-9 years old, random-effects meta-regression showed that each 100 IU/d increase in vit D supplementation was associated with an average of 2.49 (95% CI -0.24, 5.22) nmol/L increase in achieved 25(OH)D concentration (n = 16 intervention arms; *P* = 0.071; adjusted R^2 = 19.96%).	Moderate
**KQ3. Non-daily vitamin D supplementation on serum 25(OH)D**								
11	RCTs	**Some limitations:** In 4 of 5	**Some inconsistency:** Consistency of	**Indirect**: serum 25(OH)D,	**Some Imprecision:** Wide	Dose-response is present	Single doses of 200,000 IU of vitamin D_3 increased serum 25(OH)D to 317 nmol/L at one week and 246 nmol/L at 5 weeks in one study,[87] and 150 nmol/L at 2	LOW

		ROB domains, approximately 50% or more trials were assessed as having some or high ROB.	direction but some inconsistency in magnitude of the achieved 25(OH)D concentration.	a marker of vitamin D status.	CIs within some trial arms.	within most studies comparing different levels of vitamin D supplementation.	weeks in another study.[96] A single dose of 100,000 IU of vitamin D_3 resulted in serum 25(OH)D levels of 92 at 2 weeks.[96] Single doses of 50,000 IU of vitamin D_3 resulted in serum 25(OH)D levels of 154 and 62 nmol/L at 1.5 and 14 weeks in one study,[36] and 85 and 91 nmol/L at 8 and 14 weeks in the other study.[90] Single doses of 300,000 and 600,000 IU resulted in serum 25(OH)D levels of 16.1 and 17.6 nmol/L, respectively, after 12 weeks.[99] Other dose regimens, including weekly or monthly doses of vitamin D, resulted in increased 25(OH)D. 14,000 IU of vitamin D_3 weekly to resulted in mean 25(OH)D increased to 91.8 nmol/L in the vitamin D_3 supplementation.[108]	
**KQ3. Supplementation to post-partum mothers on infant serum 25(OH)D**								
4	RCTs	**Some limitations:** In 4 of 5 ROB domains, at least 50% of trials were assessed as having some or high ROB.	**Serious inconsistency:** Studies reported differences in magnitude and direction of effect across maternal supplementation arms.	**Indirect**: serum 25(OH)D, a marker of vitamin D status.	**Some Imprecision:** Some studies with wide CIs or small sample sizes.	Dose-response unable to assess.	Breastfed infant serum 25(OH)D had decreased in one maternal 1000 IU/d supplementation group in one trial.[51] Baseline serum 25(OH)D was not provided in the other three trials; however, maternal supplementation of 400 IU daily resulted in higher infant serum 25(OH)D compared to placebo at 14 weeks in one study,[45] and no significant difference between maternal 6400 IU/d and infant 300 IU/d (with maternal 400 IU/d) supplementation in another study.[41] In the last trial, maternal supplementation of 1000 IU daily, but not 2000 IU daily, resulted in infant serum 25(OH)D significantly lower than that of infants receiving direct 400 IU vitamin D_2 daily at 8 weeks. This difference was also significant at 15 weeks, but differences between the other groups were not significant.[78]	VERY LOW
**KQ3. Food interventions containing vitamin D on serum 25(OH)D**								

3	RCTs	**Some limitations:** 100% of studies had some or high ROB in 2 ROB domains.	**Some inconsistency:** Studies reported differences in the effect magnitude despite food intervention arms containing similar amounts of vitamin D.	**Indirect**: serum 25(OH)D, a marker of vitamin D status.	**Some imprecision:** Some studies with wide CIs or small sample sizes.	Dose-response unable to assess.	Serum 25(OH)D decreased in groups receiving both fortified (with mean vitamin D dose of 466-486 IU/d) and non-fortified food, although none of the changes were significant in one study.[104] In a second study, fortified formula (400 IU/L) saw no significant increase in serum 25(OH)D.[43] The last trial reported significant increases in 25(OH)D after 12 weeks of food fortified with 1,000 IU daily and 400 IU daily in both fair- and dark-skinned children, but no significant increase in the groups receiving 80 IU daily in food.[112]	VERY LOW
**KQ3. Combined vitamin D and calcium supplementation on infant serum 25(OH)D**								
3	RCTs	**Some limitations:** 100% of studies had some or high ROB in 3 ROB domains.	**No serious inconsistency:** Direction of effect consistent; unable to assess consistency of effect magnitude.	**Indirect**: serum 25(OH)D, a marker of vitamin D status.	**Some imprecision:** Studies with wide CIs.	Dose-response unable to assess.	In one study, serum 25(OH)D levels increased significantly more in the 200 IU/d of vitamin D3 plus 700 mg/d of calcium supplementation group compared to the calcium only group after 12 weeks (+12.7 nmol/L [5.09 ng/mL]; 95% CI 1.3, 24.1).[105] In another study, mean serum 25(OH)D levels did not significantly differ between groups that received 30,000 IU once monthly of vitamin D_3 plus either 405 mg or 156 mg of calcium 5 times weekly after 48 weeks; however, both groups resulted in significantly higher 25(OH)D at the end of the study.[81] In the last study where both groups got 50 mg/kg/d of calcium supplementation, there was no significant difference in mean serum 25(OH)D levels at 48 weeks between the 30,000 IU once weekly group and the 4,000 IU/d group.	VERY LOW
**UL KQ1a. Adverse effects: hypercalcemia, hypercalciuria, nephrocalcinosis, mortality, and kidney stones**								

47	RCTs, single-arm interventions, cohorts, case-cohorts, nested case-controls, cross-sectional studies, and case reports	**Some limitations**: 100% of outcomes from RCTs have high or some concerns in at least 2 ROB domains	**Some inconsistency**: Studies showed consistency among hypercalcemia outcome, but inconsistency among hypercalciuria outcome. Other upper limit outcomes were unable to be assessed due to few data (i.e., mortality, nephrocalcinosis).	**Direct**: Clinical outcomes.	**Imprecise**: Rates of upper limit outcomes are variable across studies, even among groups with similar dose and follow-up durations.	Dose-response is present within some studies assessing hypercalcemia and hypercalciuria	**Hypercalcemia:** Generally, the rate of hypercalcemia increased with the dose of vitamin D administered; however, the rate of hypercalcemia was variable, even comparing the same or similar intervention dose and durations. **Hypercalciuria:** The rate of hypercalciuria was variable among studies and interventions arms. **Other upper limit outcomes:** few high-quality studies reported on nephrocalcinosis, kidney stones, and mortality.	VERY LOW

BMC = bone mineral content; BMD = bone mineral density; CI = confidence interval; d = day; GRADE = Grading of Recommendations, Assessment, Development and Evaluations; IU = international units; KQ = key question; RCT = randomized controlled trial; ROB = risk of bias; UL = upper limit; URTI = upper respiratory tract infection; VD= Vitamin D

Appendix 2: Bibliography

Cochrane Handbook for Systematic Reviews of Interventions version 6.0 (updated July 2019). 2019. ed J.P.T. Higgins, J. Thomas, J. Chandler, M. Cumpston, T. Li, M.J. Page and V.A. Welch: Cochrane.

Abrams, S. A., K. C. Copeland, S. K. Gunn, J. E. Stuff, L. L. Clarke, and K. J. Ellis. 1999. "Calcium absorption and kinetics are similar in 7- and 8-year-old Mexican-American and Caucasian girls despite hormonal differences." *Journal of Nutrition* 129 (3): 666-71.

Abrams, Steven A., Keli M. Hawthorne, and Zhensheng Chen. 2013. "Supplementation with 1000 IU vitamin D/d leads to parathyroid hormone suppression, but not increased fractional calcium absorption, in 4-8-y-old children: a double-blind randomized controlled trial." *American Journal of Clinical Nutrition* 97 (1): 217-223. https://doi.org/10.3945/ajcn.112.046102.

Abrams, S. A., and J. E. Stuff. 1994. "Calcium metabolism in girls: current dietary intakes lead to low rates of calcium absorption and retention during puberty." *American Journal of Clinical Nutrition* 60 (5): 739-43.

Abrams, S. A., J. Wen, and J. E. Stuff. 1997. "Absorption of calcium, zinc, and iron from breast milk by five- to seven-month-old infants." *Pediatric Research* 41 (3): 384-90.

Aglipay, M., C. S. Birken, P. C. Parkin, M. B. Loeb, K. Thorpe, Y. Chen, A. Laupacis, M. Mamdani, C. Macarthur, J. S. Hoch, T. Mazzulli, J. L. Maguire, and T. ARGet Kids! Collaboration. 2017. "Effect of High-Dose vs Standard-Dose Wintertime Vitamin D Supplementation on Viral Upper Respiratory Tract Infections in Young Healthy Children." *JAMA* 318 (3): 245-254. https://doi.org/https://dx.doi.org/10.1001/jama.2017.8708.

Ahmed, A. M. S., T. Ahmed, R. J. Soares Magalhaes, K. Z. Long, M. A. Alam, M. I. Hossain, M. M. Islam, M. Mahfuz, D. Mondal, R. Haque, and A. Al Mamun. 2016. "Association between serum Vitamin D, retinol and zinc status, and acute respiratory infections in underweight and normal-weight children aged 6-24 months living in an urban slum in Bangladesh." *Epidemiology and Infection* 144 (16): 3494-3506.

Ala-Houhala, M., T. Koskinen, A. Terho, T. Koivula, and J. Visakorpi. 1986. "Maternal compared with infant vitamin D supplementation." *Archives of Disease in Childhood* 61 (12): 1159-63.

Alahouhala, M. 1985. "25-HYDROXYVITAMIN-D LEVELS DURING BREAST-FEEDING WITH OR WITHOUT MATERNAL OR INFANTILE SUPPLEMENTATION OF VITAMIN-D." *Journal of Pediatric Gastroenterology and Nutrition* 4 (2): 220-226. https://doi.org/10.1097/00005176-198504000-00011.

Alahouhala, M., T. Koskinen, M. Koskinen, and J. K. Visakorpi. 1988. "DOUBLE-BLIND STUDY ON THE NEED FOR VITAMIN-D SUPPLEMENTATION IN PREPUBERTAL CHILDREN." *Acta Paediatrica Scandinavica* 77 (1): 89-93.

Alonso, A., J. Rodriguez, I. Carvajal, M. A. Prieto, R. M. Rodriguez, A. M. Perez, A. Cepeda, F. Nuno, F. Santos, and D. in Asturias Collaborative Group on Prophylaxis with Vitamin. 2011. "Prophylactic vitamin D in healthy infants: assessing the need." *Metabolism: Clinical & Experimental* 60 (12): 1719-25. https://doi.org/https://dx.doi.org/10.1016/j.metabol.2011.04.011.

Aluisio, A. R., Z. Maroof, D. Chandramohan, J. Bruce, M. Z. Mughal, Z. Bhutta, G. Walraven, M. I. Masher, J. H. Ensink, and S. Manaseki-Holland. 2013. "Vitamin D3supplementation and childhood

diarrhea: a randomized controlled trial." *Pediatrics* 132 (4): e832-40. https://doi.org/https://dx.doi.org/10.1542/peds.2012-3986.

Ames, S. K., B. M. Gorham, and S. A. Abrams. 1999. "Effects of high compared with low calcium intake on calcium absorption and incorporation of iron by red blood cells in small children." *American Journal of Clinical Nutrition* 70 (1): 44-8.

Andersen, B. 1954. "[Vitamin D poisoning]." *Tidsskrift for Den Norske Laegeforening* 74 (12): 419-20.

Anderson, L. N., Y. Chen, J. A. Omand, C. S. Birken, P. C. Parkin, T. To, J. L. Maguire, and T. ARGet Kids Collaboration. 2015. "Vitamin D exposure during pregnancy, but not early childhood, is associated with risk of childhood wheezing." *Journal of Developmental Origins of Health and Disease* 6 (4): 308-16. https://doi.org/https://dx.doi.org/10.1017/S2040174415001063.

Atas, E., F. Karademir, A. Ersen, C. Meral, S. Aydinoz, S. Suleymanoglu, M. Gultepe, and I. Gocmen. 2013. "Comparison between daily supplementation doses of 200 versus 400 IU of vitamin D in infants." *European Journal of Pediatrics* 172 (8): 1039-42. https://doi.org/https://dx.doi.org/10.1007/s00431-013-1997-4.

Bagnoli, F., M. Casucci, S. Toti, S. Cecchi, C. Iurato, G. Coriolani, M. Tiezzi, and L. Vispi. 2013. "Is vitamin D supplementation necessary in healthy full-term breastfed infants? A follow-up study of bone mineralization in healthy full-term infants with and without supplemental vitamin D." *Minerva Pediatrica* 65 (3): 253-60.

Barlow, William E., Laura Ichikawa, Dan Rosner, and Shizue Izumi. 1999. "Analysis of Case-Cohort Designs." *Journal of Clinical Epidemiology* 52 (12): 1165-1172. https://doi.org/https://doi.org/10.1016/S0895-4356(99)00102-X.

Barltrop, D., R. H. Mole, and A. Sutton. 1977. "Absorption and endogenous faecal excretion of calcium by low birthweight infants on feeds with varying contents of calcium and phosphate." *Archives of Disease in Childhood* 52 (1): 41-9.

Barnes, L. A., G. Morrow, 3rd, J. Silverio, L. P. Finnegan, and S. E. Heitman. 1974. "Calcium and fat absorption from infant formulas with different fat blends." *Pediatrics* 54 (2): 217-21.

Barrueto, F., Jr., H. H. Wang-Flores, M. A. Howland, R. S. Hoffman, and L. S. Nelson. 2005. "Acute vitamin D intoxication in a child." *Pediatrics* 116 (3): e453-6.

Begum, A., and S. M. Pereira. 1969. "Calcium balance studies on children accustomed to low calcium intakes." *British Journal of Nutrition* 23 (4): 905-11.

Besbas, N., A. Oner, O. Akhan, U. Saatci, A. Bakkaloglu, and R. Topaloglu. 1989. "Nephrocalcinosis due to vitamin D intoxication." *Turkish Journal of Pediatrics* 31 (3): 239-44.

Bonjour, J. P., A. L. Carrie, S. Ferrari, H. Clavien, D. Slosman, G. Theintz, and R. Rizzoli. 1997. "Calcium-enriched foods and bone mass growth in prepubertal girls: a randomized, double-blind, placebo-controlled trial." *Journal of Clinical Investigation* 99 (6): 1287-94.

Boteju, P. R. D., P. Bhaskaram, and V. Reddy. 1982. "Vitamin D requirements of Indian children." *Nutrition Reports International* 26 (1): 135-139.

Breslow, Norman E., Thomas Lumley, Christie M. Ballantyne, Lloyd E. Chambless, and Michal Kulich. 2009. "Using the whole cohort in the analysis of case-cohort data." *American journal of epidemiology* 169 (11): 1398-1405. https://doi.org/10.1093/aje/kwp055.

Brett, N. R., P. Lavery, S. Agellon, C. A. Vanstone, J. L. Maguire, F. Rauch, and H. A. Weiler. 2016. "Dietary vitamin D dose-response in healthy children 2 to 8 y of age: a 12-wk randomized controlled trial using fortified foods." *American Journal of Clinical Nutrition* 103 (1): 144-52. https://doi.org/https://dx.doi.org/10.3945/ajcn.115.115956.

Brett, N. R., C. A. Parks, P. Lavery, S. Agellon, C. A. Vanstone, M. Kaufmann, G. Jones, J. L. Maguire, F. Rauch, and H. A. Weiler. 2018. "Vitamin D status and functional health outcomes in children aged 2-8 y: a 6-mo vitamin D randomized controlled trial." *American Journal of Clinical Nutrition* 107 (3): 355-364. https://doi.org/https://dx.doi.org/10.1093/ajcn/nqx062.

Cadario, F., S. Savastio, V. Pagliardini, M. Bagnati, M. Vidali, F. Cerutti, I. Rabbone, F. Fontana, R. Lera, V. De Donno, A. Valori, G. Gruden, G. Bona, and G. Bruno. 2015. "Vitamin D levels at birth and risk of type 1 diabetes in childhood: a case-control study." *Acta Diabetologica* 52 (6): 1077-81.

Chan, G. M., C. C. Roberts, D. Folland, and R. Jackson. 1982. "Growth and bone mineralization of normal breast-fed infants and the effects of lactation on maternal bone mineral status." *American Journal of Clinical Nutrition* 36 (3): 438-43.

Chandy, D. D., J. Kare, S. N. Singh, A. Agarwal, V. Das, U. Singh, V. Ramesh, and V. Bhatia. 2016. "Effect of vitamin D supplementation, directly or via breast milk for term infants, on serum 25 hydroxyvitamin D and related biochemistry, and propensity to infection: a randomised placebo-controlled trial." *British Journal of Nutrition* 116 (1): 52-8. https://doi.org/https://dx.doi.org/10.1017/S0007114516001756.

Chen, Y., Y. Liu, H. Xue, Y. Bao, J. Luo, G. Tian, and G. Cheng. 2016. "[Association between dietary calcium/dairy intakes and overweight/obesity]." *Wei Sheng Yen Chiu/Journal of Hygiene Research* 45 (3): 402-8.

Chowdhury, R., S. Taneja, N. Bhandari, I. Kvestad, T. A. Strand, and M. K. Bhan. 2017. "Vitamin-D status and neurodevelopment and growth in young north Indian children: a secondary data analysis." *Nutrition Journal* 16 (1): 59.

Chung, M., E. M. Balk, M. Brendel, S. Ip, J. Lau, J. Lee, A. Lichtenstein, K. Patel, G. Raman, A. Tatsioni, T. Terasawa, and T. A. Trikalinos. 2009. "Vitamin D and calcium: a systematic review of health outcomes." *Evid Rep Technol Assess (Full Rep)* (183): 1-420.

Chung, M., M. Ruan, K.C. Cara, Q. Yao, L.P. Penkert, and J. Chen. 2020. "Vitamin D and calcium in children 0-36 months: a scoping review of health outcomes." *Journal of the American College of Nutrition.* [in press].

Clemente Yago, F., E. Cortes Castell, M. Moya Benavent, I. Ballester Galindo, A. Herrero Galiana, and M. J. Campello Oltra. 1989. "[Mineral metabolism of calcium, magnesium and phosphorus in the normal newborn infant]." *Anales Espanoles de Pediatria* 31 (6): 533-6.

Creo, A. L., J. S. Rosen, A. J. Ariza, K. M. Hidaka, and H. J. Binns. 2013. "Vitamin D levels, insulin resistance, and cardiovascular risks in very young obese children." *Journal of Pediatric Endocrinology & Metabolism* 26 (1): 97-104.

Dawodu, A., K. M. Salameh, N. S. Al-Janahi, A. Bener, and N. Elkum. 2019. "The Effect of High-Dose Postpartum Maternal Vitamin D Supplementation Alone Compared with Maternal Plus Infant Vitamin D Supplementation in Breastfeeding Infants in a High-Risk Population. A Randomized Controlled Trial." *Nutrients* 11 (7): 17. https://doi.org/https://dx.doi.org/10.3390/nu11071632.

Demir, K., H. Doneray, C. Kara, Z. Atay, S. Cetinkaya, A. Cayir, A. Anik, E. Eren, A. Ucakturk, G. Can Yilmaz, A. Torel Ergur, M. Kendirci, Z. Aycan, A. Bereket, M. Aydin, Z. Orbak, and B. Ozkan. 2019. "Comparison of Treatment Regimens in Management of Severe Hypercalcemia Due to Vitamin D Intoxication in Children." *Journal of clinical research in pediatric endocrinology* 11 (2): 140-148. https://doi.org/https://dx.doi.org/10.4274/jcrpe.galenos.2018.2018.0131.

DerSimonian, R., and N. Laird. 1986. "Meta-analysis in clinical trials." *Control Clin Trials* 7 (3): 177-88.

DeVizia, B., S. J. Fomon, S. E. Nelson, B. E. Edwards, and E. E. Ziegler. 1985. "Effect of dietary calcium on metabolic balance of normal infants." *Pediatric Research* 19 (8): 800-6.

Döneray, H., B. Özkan, A. Özkan, C. Koşan, Z. Orbak, and C. Karakelleoğlu. 2009. "The clinical and laboratory characteristics of vitamin D intoxication in children." *Turkish Journal of Medical Sciences* 39 (1): 1-4.

Down, P. F., A. Polak, and R. J. Regan. 1979. "A family with massive acute vitamin D intoxication." *Postgraduate Medical Journal* 55 (654): 897-902.

Economos, C. D., C. E. Moore, R. R. Hyatt, J. Kuder, T. Chen, S. N. Meydani, M. Meydani, E. Klein, R. M. Biancuzzo, and M. F. Holick. 2014. "Multinutrient-fortified juices improve vitamin D and vitamin E status in children: a randomized controlled trial." *Journal of the Academy of Nutrition & Dietetics* 114 (5): 709-17. https://doi.org/https://dx.doi.org/10.1016/j.jand.2013.07.027.

Eden, Jill. *Finding What Works in Health Care: Standards for Systematic Reviews*, edited by J. Eden, L. Levit, A. Berg and S. Morton. Washington (DC).:National Academies Press, 2011.

Ekbote, V. H., A. V. Khadilkar, S. A. Chiplonkar, N. M. Hanumante, V. V. Khadilkar, and M. Z. Mughal. 2011. "A pilot randomized controlled trial of oral calcium and vitamin D supplementation using fortified laddoos in underprivileged Indian toddlers." *European Journal of Clinical Nutrition* 65 (4): 440-6.

Elarqam, L., A. Babakhouya, S. Chaouki, S. Atmani, A. Bouharrou, and M. Hida. 2007. "Intoxication by vitamin D in infant." *Journal de Pediatrie et de Puericulture* 20 (5): 203-205. https://doi.org/10.1016/j.jpp.2007.07.001.

Enlund-Cerullo, M., L. Koljonen, E. Holmlund-Suila, H. Hauta-Alus, J. Rosendahl, S. Valkama, O. Helve, T. Hytinantti, H. Viljakainen, S. Andersson, O. Makitie, and M. Pekkinen. 2019. "Genetic Variation of the Vitamin D Binding Protein Affects Vitamin D Status and Response to Supplementation in Infants." *J Clin Endocrinol Metab* 104 (11): 5483-5498. https://doi.org/10.1210/jc.2019-00630.

Escribano, J., C. Rubio-Torrents, N. Ferre, V. Luque, V. Grote, M. Zaragoza-Jordana, M. Gispert-Llaurado, R. Closa-Monasterolo, and Group European Childhood Obesity Project. 2014. "Reduced bone mass in 7-year-old children with asymptomatic idiopathic hypercalciuria." *Annals of Nutrition & Metabolism* 64 (3-4): 304-13. https://doi.org/https://dx.doi.org/10.1159/000365038.

Evliyaoglu, O., M. Berberoglu, G. Ocal, P. Adiyaman, and Z. Aycan. 2001. "Severe hypercalcemia of an infant due to vitamin D toxicity associated with hypercholesterolemia." *Journal of Pediatric Endocrinology & Metabolism* 14 (7): 915-9.

Fabian, K., L. Velkey, and E. Fodor. 1990. "[Preventive value of vitamin D in various doses based on a prospective study of healthy infants]." *Orvosi Hetilap* 131 (9): 447-50.

Gallo, Sina, Kathryn Comeau, Catherine Vanstone, Sherry Agellon, Atul Sharma, Glenville Jones, Mary L'Abbe, Ali Khamessan, Celia Rodd, and Hope Weiler. 2013. "Effect of Different Dosages of Oral Vitamin D Supplementation on Vitamin D Status in Healthy, Breastfed Infants A Randomized Trial." *Jama-Journal of the American Medical Association* 309 (17): 1785-1792. https://doi.org/10.1001/jama.2013.3404.

Gallo, S., T. Hazell, C. A. Vanstone, S. Agellon, G. Jones, M. L'Abbe, C. Rodd, and H. A. Weiler. 2016. "Vitamin D supplementation in breastfed infants from Montreal, Canada: 25-hydroxyvitamin D and bone health effects from a follow-up study at 3 years of age." *Osteoporosis International* 27 (8): 2459-66. https://doi.org/https://dx.doi.org/10.1007/s00198-016-3549-z.

Gallo, S., A. Phan, C. A. Vanstone, C. Rodd, and H. A. Weiler. 2013. "The change in plasma 25-hydroxyvitamin D did not differ between breast-fed infants that received a daily supplement of ergocalciferol or cholecalciferol for 3 months." *Journal of Nutrition* 143 (2): 148-53. https://doi.org/https://dx.doi.org/10.3945/jn.112.167858.

Garbim, B. B., D. Avila L, S. Z. P. Rigatto, Krds Quadros, V. M. S. Belangero, and R. B. Oliveira. 2017. "Hypercalcemia in children: three cases report with unusual clinical presentations." *Jornal Brasileiro de Nefrologia* 39 (2): 213-216. https://doi.org/https://dx.doi.org/10.5935/0101-2800.20170036.

Giunta, J. L. 1998. "Dental changes in hypervitaminosis D." *Oral Surgery Oral Medicine Oral Pathology Oral Radiology & Endodontics* 85 (4): 410-3.

Gordon, Catherine M., Avery LeBoff Williams, Henry A. Feldman, Jessica May, Linda Sinclair, Alex Vasquez, and Joanne E. Cox. 2008. "Treatment of hypovitaminosis D in infants and toddlers." *Journal of Clinical Endocrinology & Metabolism* 93 (7): 2716-2721. https://doi.org/10.1210/jc.2007-2790.

Grant, C. C., J. Crane, E. A. Mitchell, J. Sinclair, A. Stewart, T. Milne, J. Knight, C. Gilchrist, and C. A. Camargo, Jr. 2016. "Vitamin D supplementation during pregnancy and infancy reduces aeroallergen sensitization: a randomized controlled trial." *Allergy* 71 (9): 1325-34. https://doi.org/https://dx.doi.org/10.1111/all.12909.

Grant, C. C., S. Kaur, E. Waymouth, E. A. Mitchell, R. Scragg, A. Ekeroma, A. Stewart, J. Crane, A. Trenholme, and C. A. Camargo, Jr. 2015. "Reduced primary care respiratory infection visits following pregnancy and infancy vitamin D supplementation: a randomised controlled trial." *Acta Paediatrica* 104 (4): 396-404. https://doi.org/https://dx.doi.org/10.1111/apa.12819.

Grant, C. C., A. W. Stewart, R. Scragg, T. Milne, J. Rowden, A. Ekeroma, C. Wall, E. A. Mitchell, S. Crengle, A. Trenholme, J. Crane, and C. A. Camargo, Jr. 2014. "Vitamin D during pregnancy and infancy and infant serum 25-hydroxyvitamin D concentration." *Pediatrics* 133 (1): e143-53. https://doi.org/https://dx.doi.org/10.1542/peds.2013-2602.

Greenland, S., and M. P. Longnecker. 1992. "Methods for trend estimation from summarized dose-response data, with applications to meta-analysis." *Am J Epidemiol* 135 (11): 1301-9.

Greer, F. R., J. E. Searcy, R. S. Levin, J. J. Steichen, P. S. Steichen-Asche, and R. C. Tsang. 1982. "Bone mineral content and serum 25-hydroxyvitamin D concentrations in breast-fed infants with and without supplemental vitamin D: one-year follow-up." *Journal of Pediatrics* 100 (6): 919-22.

Gurkan, F., M. Davutoglu, M. Bosnak, A. Ece, B. Dikici, M. Bilici, and K. Haspolat. 2004. "Pamidronate treatment in acute vitamin D intoxication." *Journal of Endocrinological Investigation* 27 (7): 680-2.

Guyatt, G., A. D. Oxman, E. A. Akl, R. Kunz, G. Vist, J. Brozek, S. Norris, Y. Falck-Ytter, P. Glasziou, H. DeBeer, R. Jaeschke, D. Rind, J. Meerpohl, P. Dahm, and H. J. Schunemann. 2011. "GRADE guidelines: 1. Introduction-GRADE evidence profiles and summary of findings tables." *J Clin Epidemiol* 64 (4): 383-94. https://doi.org/10.1016/j.jclinepi.2010.04.026.

Guyatt, G. H., A. D. Oxman, R. Kunz, G. E. Vist, Y. Falck-Ytter, H. J. Schunemann, and Grade Working Group. 2008. "What is "quality of evidence" and why is it important to clinicians?" *BMJ* 336 (7651): 995-8. https://doi.org/10.1136/bmj.39490.551019.BE.

Guyatt, G. H., A. D. Oxman, G. E. Vist, R. Kunz, Y. Falck-Ytter, P. Alonso-Coello, H. J. Schunemann, and Grade Working Group. 2008. "GRADE: an emerging consensus on rating quality of evidence and strength of recommendations." *BMJ* 336 (7650): 924-6. https://doi.org/10.1136/bmj.39489.470347.AD.

Handel, M. N., P. Frederiksen, A. Cohen, C. Cooper, B. L. Heitmann, and B. Abrahamsen. 2017. "Neonatal vitamin D status from archived dried blood spots and future risk of fractures in childhood: results from the D-tect study, a population-based case-cohort study." *American Journal of Clinical Nutrition* 106 (1): 155-161.

Hanna, F. M., D. A. Navarrete, and F. A. Hsu. 1970. "Calcium fatty acid absorption in term infants fed human milk and prepared formulas simulating human milk." *Pediatrics* 45 (2): 216-224.

Harbord, RM, and JPT Higgins. 2008. "Meta-Regression in Stata." *The Stata Journal* 8 (4): 493-519.

Harmonization of Approaches to Nutrient Reference Values: Applications to Young Children and Women of Reproductive Age. Washington (DC). The National Academies Press, 2018.

Harnot, J., S. Verma, S. Singhi, N. Sankhyan, N. Sachdeva, and B. Bharti. 2017. "Comparison of 300,000 and 600,000 IU Oral Vitamin-D Bolus for Vitamin-D Deficiency in Young Children." *Indian Journal of Pediatrics* 84 (2): 111-116. https://doi.org/https://dx.doi.org/10.1007/s12098-016-2233-9.

Hazell, T. J., S. Gallo, C. A. Vanstone, S. Agellon, C. Rodd, and H. A. Weiler. 2017. "Vitamin D supplementation trial in infancy: body composition effects at 3 years of age in a prospective follow-up study from Montreal." *Pediatric Obesity* 12 (1): 38-47. https://doi.org/https://dx.doi.org/10.1111/ijpo.12105.

Heras Gironella, M., M. J. Olivan del Cacho, and J. Sierra Sirvent. 1982. "Renal complaints in vitamin D intoxication." *Revista Espanola de Pediatria* 38 (227): 319-326.

Hibbs, A. M., K. Ross, L. A. Kerns, C. Wagner, M. Fuloria, S. Groh-Wargo, T. Zimmerman, N. Minich, and C. Tatsuoka. 2018. "Effect of Vitamin D Supplementation on Recurrent Wheezing in Black Infants Who Were Born Preterm: The D-Wheeze Randomized Clinical Trial." *JAMA* 319 (20): 2086-2094. https://doi.org/https://dx.doi.org/10.1001/jama.2018.5729.

Hillman, L. S., E. Tack, D. G. Covell, N. E. Vieira, and A. L. Yergey. 1988. "Measurement of true calcium absorption in premature infants using intravenous 46Ca and oral 44Ca." *Pediatric Research* 23 (6): 589-94.

Hirschler, V., G. Maccallini, M. I. Tamborenea, C. Gonzalez, M. Sanchez, C. Molinari, Group San Antonio de Los Cobres Study, L. Castano, G. Colque, M. Hidalgo, and M. Urzagasti. 2014. "Improvement in lipid profile after vitamin D supplementation in indigenous argentine school children." *Cardiovascular & Hematological Agents in Medicinal Chemistry* 12 (1): 42-9.

Ho, M. L., H. C. Yen, R. C. Tsang, B. L. Specker, X. C. Chen, and B. L. Nichols. 1985. "Randomized study of sunshine exposure and serum 25-OHD in breast-fed infants in Beijing, China." *Journal of Pediatrics* 107 (6): 928-31.

Holden, J. M., and L. E. Lemar. 2008. "Assessing vitamin D contents in foods and supplements: challenges and needs." *Am J Clin Nutr* 88 (2): 551S-553S. https://doi.org/10.1093/ajcn/88.2.551S.

Hollams, E. M., S. M. Teo, M. Kusel, B. J. Holt, K. E. Holt, M. Inouye, N. H. De Klerk, G. Zhang, P. D. Sly, P. H. Hart, and P. G. Holt. 2017. "Vitamin D over the first decade and susceptibility to childhood allergy and asthma." *Journal of Allergy & Clinical Immunology* 139 (2): 472-481.e9.

Hollis, B. W., C. L. Wagner, C. R. Howard, M. Ebeling, J. R. Shary, P. G. Smith, S. N. Taylor, K. Morella, R. A. Lawrence, and T. C. Hulsey. 2015. "Maternal Versus Infant Vitamin D Supplementation During Lactation: A Randomized Controlled Trial." *Pediatrics* 136 (4): 625-34. https://doi.org/https://dx.doi.org/10.1542/peds.2015-1669.

Holmlund-Suila, Elisa, Heli Viljakainen, Timo Hytinantti, Christel Lamberg-Allardt, Sture Andersson, and Outi Makitie. 2012. "High-Dose Vitamin D Intervention in Infants-Effects on Vitamin D Status, Calcium Homeostasis, and Bone Strength." *Journal of Clinical Endocrinology & Metabolism* 97 (11): 4139-4147. https://doi.org/10.1210/jc.2012-1575.

Holstgemeiner, D., M. Gemeiner, I. Pilz, and W. Swoboda. 1978. "PLASMA 25-HYDROXYCHOLECALCIFEROL AFTER DAILY VITAMIN-D ADMINISTRATION IN COMPARISON WITH MASSIVE SINGLE-DOSE PROPHYLAXIS." *Wiener Klinische Wochenschrift* 90 (14): 509-512.

Hoppe, B., H. E. Gnehm, M. Wopmann, T. Neuhaus, U. Willi, and E. Leumann. 1992. "[Vitamin D poisoning in infants: a preventable cause of hypercalciuria and nephrocalcinosis]." *Schweizerische Medizinische Wochenschrift. Journal Suisse de Medecine* 122 (8): 257-62.

Huynh, J., T. Lu, D. Liew, J. C. Doery, R. Tudball, M. Jona, R. Bhamjee, and C. P. Rodda. 2017. "Vitamin D in newborns. A randomised controlled trial comparing daily and single oral bolus vitamin D in infants." *Journal of Paediatrics & Child Health* 53 (2): 163-169. https://doi.org/https://dx.doi.org/10.1111/jpc.13338.

Institute of Medicine, . 2011. *Finding What Works in Health Care: Standards for Systematic Reviews*. Edited by J. Eden, L. Levit, A. Berg and S. Morton. Washington, D.C.

Jackson, D., I. R. White, and R. D. Riley. 2013. "A matrix-based method of moments for fitting the multivariate random effects model for meta-analysis and meta-regression." *Biom J* 55 (2): 231-45. https://doi.org/10.1002/bimj.201200152.

Jacobsen, R., S. U. Thorsen, A. S. Cohen, M. Lundqvist, P. Frederiksen, C. B. Pipper, F. Pociot, L. C. Thygesen, A. Ascherio, J. Svensson, and B. L. Heitmann. 2016. "Neonatal vitamin D status is not associated with later risk of type 1 diabetes: results from two large Danish population-based studies." *Diabetologia* 59 (9): 1871-81.

Jensen, C. B., M. Lundqvist, T. I. A. Sorensen, and B. L. Heitmann. 2017. "Neonatal Vitamin D Levels in Relation to Risk of Overweight at 7 Years in the Danish D-Tect Case-Cohort Study." *Obesity Facts* 10 (3): 273-283. https://doi.org/https://dx.doi.org/10.1159/000471881.

Johansson, Anna L.V. 2016. "The Case-Cohort design: What it is and how it can be used in register-based research." 13 September, 2016, Presentation for Stata Users Group Meeting, Oslo. stata.com: Stata.

Jung, H., H. Borowiec, and R. Grenda. 1984. "[Renal failure in hypercalcemic nephropathy caused by vitamin D 3 poisoning]." *Pediatria Polska* 59 (1): 51-5.

Karlsland Akeson, P., K. E. Akesson, T. Lind, O. Hernell, S. A. Silfverdal, and I. Ohlund. 2018. "Vitamin D Intervention and Bone: A Randomized Clinical Trial in Fair- and Dark-skinned Children at Northern Latitudes." *Journal of Pediatric Gastroenterology & Nutrition* 67 (3): 388-394.

Ketha, H., H. Wadams, A. Lteif, and R. J. Singh. 2015. "Iatrogenic vitamin D toxicity in an infant--a case report and review of literature." *Journal of Steroid Biochemistry & Molecular Biology* 148: 14-8. https://doi.org/https://dx.doi.org/10.1016/j.jsbmb.2015.01.022.

Kienzle, E. 1998. "Factorial calculation of nutrient requirements in lactating queens." *J Nutr* 128 (12 Suppl): 2609S-2614S. https://doi.org/10.1093/jn/128.12.2609S.

Kim, Ryung S. 2013. "Analysis of Nested Case-Control Study Designs: Revisiting the Inverse Probability Weighting Method." *Communications for statistical applications and methods* 20 (6): 455-466. https://doi.org/10.5351/CSAM.2013.20.6.455.

Kumar, G. T., H. S. Sachdev, H. Chellani, A. M. Rehman, V. Singh, H. Arora, and S. Filteau. 2011. "Effect of weekly vitamin D supplements on mortality, morbidity, and growth of low birthweight term infants in India up to age 6 months: randomised controlled trial." *BMJ* 342: d2975. https://doi.org/https://dx.doi.org/10.1136/bmj.d2975.

Kunz, C., H. Vonlilienfeldtoal, M. Niesen, and W. Burmeister. 1982. "25-HYDROXY-VITAMIN-D IN SERUM OF NEWBORNS AND INFANTS DURING CONTINUOUS ORAL VITAMIN-D TREATMENT." *Padiatrie Und Padologie* 17 (2): 181-185.

Lee, S. H., S. M. Kim, H. S. Park, K. M. Choi, G. J. Cho, B. J. Ko, and J. H. Kim. 2013. "Serum 25-hydroxyvitamin D levels, obesity and the metabolic syndrome among Korean children." *Nutrition Metabolism & Cardiovascular Diseases* 23 (8): 785-91. https://doi.org/https://dx.doi.org/10.1016/j.numecd.2012.04.013.

Lee, W. T., S. S. Leung, S. J. Fairweather-Tait, D. M. Leung, H. S. Tsang, J. Eagles, T. Fox, S. H. Wang, Y. C. Xu, W. P. Zeng, and et al. 1994. "True fractional calcium absorption in Chinese children measured with stable isotopes (42Ca and 44Ca)." *British Journal of Nutrition* 72 (6): 883-97.

Lee, W. T., S. S. Leung, D. M. Leung, H. S. Tsang, J. Lau, and J. C. Cheng. 1995. "A randomized double-blind controlled calcium supplementation trial, and bone and height acquisition in children." *British Journal of Nutrition* 74 (1): 125-39.

Lee, W. T., S. S. Leung, D. M. Leung, S. H. Wang, Y. C. Xu, W. P. Zeng, and J. C. Cheng. 1997. "Bone mineral acquisition in low calcium intake children following the withdrawal of calcium supplement." *Acta Paediatrica* 86 (6): 570-6.

Lee, W. T., S. S. Leung, S. H. Wang, Y. C. Xu, W. P. Zeng, J. Lau, S. J. Oppenheimer, and J. C. Cheng. 1994. "Double-blind, controlled calcium supplementation and bone mineral accretion in children accustomed to a low-calcium diet." *American Journal of Clinical Nutrition* 60 (5): 744-50.

Lichtenstein, A. H., E. A. Yetley, and J. Lau. 2008. "Application of systematic review methodology to the field of nutrition." *J Nutr* 138 (12): 2297-306. https://doi.org/10.3945/jn.108.097154.

Loeb, M., A. D. Dang, V. D. Thiem, V. Thanabalan, B. Wang, N. B. Nguyen, H. T. M. Tran, T. M. Luong, P. Singh, M. Smieja, J. Maguire, and E. Pullenayegum. 2019. "Effect of Vitamin D supplementation to reduce respiratory infections in children and adolescents in Vietnam: A randomized controlled trial." *Influenza & Other Respiratory Viruses* 13 (2): 176-183. https://doi.org/https://dx.doi.org/10.1111/irv.12615.

Lynch, M. F., I. J. Griffin, K. M. Hawthorne, Z. Chen, M. Hamzo, and S. A. Abrams. 2007. "Calcium balance in 1-4-y-old children." *American Journal of Clinical Nutrition* 85 (3): 750-4.

Madar, A. A., K. I. Klepp, and H. E. Meyer. 2009. "Effect of free vitamin D(2) drops on serum 25-hydroxyvitamin D in infants with immigrant origin: a cluster randomized controlled trial." *European Journal of Clinical Nutrition* 63 (4): 478-84. https://doi.org/https://dx.doi.org/10.1038/sj.ejcn.1602982.

Makinen, M., J. Mykkanen, M. Koskinen, V. Simell, R. Veijola, H. Hyoty, J. Ilonen, M. Knip, O. Simell, and J. Toppari. 2016. "Serum 25-Hydroxyvitamin D Concentrations in Children Progressing to Autoimmunity and Clinical Type 1 Diabetes." *Journal of Clinical Endocrinology & Metabolism* 101 (2): 723-9. https://doi.org/https://dx.doi.org/10.1210/jc.2015-3504.

Manaseki-Holland, S., Z. Maroof, J. Bruce, M. Z. Mughal, M. I. Masher, Z. A. Bhutta, G. Walraven, and D. Chandramohan. 2012. "Effect on the incidence of pneumonia of vitamin D supplementation by quarterly bolus dose to infants in Kabul: a randomised controlled superiority trial." *Lancet* 379 (9824): 1419-27. https://doi.org/https://dx.doi.org/10.1016/S0140-6736(11)61650-4.

Mandlik, R., Z. Mughal, A. Khadilkar, S. Chiplonkar, V. Ekbote, N. Kajale, V. Patwardhan, R. Padidela, and V. Khadilkar. 2020. "Occurrence of infections in schoolchildren subsequent to supplementation with vitamin D-calcium or zinc: a randomized, double-blind, placebo-controlled trial." *Nutr Res Pract* 14 (2): 117-126. https://doi.org/10.4162/nrp.2020.14.2.117.

Mandlik, R. M., Z. M. Mughal, A. V. Khadilkar, V. H. Ekbote, N. A. Kajale, V. G. Patwardhan, V. V. Khadilkar, and R. Padidela. 2019. "Paradoxical Response of Parathyroid Hormone to Vitamin Da[Euro sign]"Calcium Supplementation in Indian Children." *Journal of pediatrics*.

Manz, F., L. Diekmann, and G. J. Stock. 1989. "Effect of calcium supplementation on calcium and phosphorus balance and renal net acid excretion in preterm infants fed a standard formula." *Acta Paediatrica Scandinavica* 78 (4): 525-31.

Markestad, T., V. Hesse, M. Siebenhuner, G. Jahreis, L. Aksnes, W. Plenert, and D. Aarskog. 1987. "INTERMITTENT HIGH-DOSE VITAMIN-D PROPHYLAXIS DURING INFANCY - EFFECT ON VITAMIN-D METABOLITES, CALCIUM, AND PHOSPHORUS." *American Journal of Clinical Nutrition* 46 (4): 652-658.

Marwaha, R. K., A. Mithal, N. Bhari, G. Sethuraman, S. Gupta, M. Shukla, A. Narang, A. Chadda, N. Gupta, V. Sreenivas, and M. A. Ganie. 2018. "Supplementation with Three Different Daily Doses of Vitamin D3 in Healthy Pre-pubertal School Girls: A Cluster Randomized Trial." *Indian Pediatrics* 55 (11): 951-956.

Misselwitz, J., and V. Hesse. 1986. "[Hypercalcemia following prophylactic vitamin D administration]." *Kinderarztliche Praxis* 54 (8): 431-8.

Misselwitz, J., and E. Kauf. 1981. "[Urinary calcium in healthy children as the basis for hypercalciuria in calcium nephrolithiasis]." *Kinderarztliche Praxis* 49 (2): 64-71.

Moher, D., A. Liberati, J. Tetzlaff, D. G. Altman, and Prisma Group. 2009. "Preferred reporting items for systematic reviews and meta-analyses: the PRISMA statement." *PLoS Med* 6 (7): e1000097. https://doi.org/10.1371/journal.pmed.1000097.

Molloy, J., J. J. Koplin, K. J. Allen, M. L. K. Tang, F. Collier, J. B. Carlin, R. Saffery, D. Burgner, S. Ranganathan, T. Dwyer, A. C. Ward, M. Moreno-Betancur, M. Clarke, A. L. Ponsonby, P. Vuillermin, and B. I. S. Investigator Group. 2017. "Vitamin D insufficiency in the first 6 months of infancy and challenge-proven IgE-mediated food allergy at 1 year of age: a case-cohort study." *Allergy* 72 (8): 1222-1231. https://doi.org/https://dx.doi.org/10.1111/all.13122.

Moodley, A., and S. A. Spector. 2015. "Single high-dose vitamin D at birth corrects vitamin D deficiency in infants in Mexico." *International Journal of Food Sciences & Nutrition* 66 (3): 336-41. https://doi.org/https://dx.doi.org/10.3109/09637486.2014.992006.

Morawska, Z., D. Zwolinska, U. Berny, and I. Makulska. 1988. "[Urinary calculi in children]." *Wiadomosci Lekarskie* 41 (17): 1147-50.

Moreira, P., C. Padez, I. Mourao, and V. Rosado. 2005. "Dietary calcium and body mass index in Portuguese children." *European Journal of Clinical Nutrition* 59 (7): 861-7.

Mortensen, Charlotte, Camilla T. Damsgaard, Hanne Hauger, Christian Ritz, Susan A. Lanham-New, Taryn J. Smith, Aine Hennessy, Kirsten Dowling, Kevin D. Cashman, Mairead Kiely, and Christian Molgaard. 2016. "Estimation of the dietary requirement for vitamin D in white children aged 4-8 y: a randomized, controlled, dose-response trial." *American Journal of Clinical Nutrition* 104 (5): 1310-1317. https://doi.org/10.3945/ajcn.116.136697.

Mortensen, C., C. Molgaard, H. Hauger, M. Kristensen, and C. T. Damsgaard. 2019. "Winter vitamin D₃ supplementation does not increase muscle strength, but modulates the IGF-axis in young children." *European Journal of Nutrition* 58 (3): 1183-1192.

Moya, M., E. Cortes, M. Juste, A. Vera, and J. Gonzalez de Dios. 1998. "[Absorptive pattern of individual fatty acids and total fat in full term babies. Its stability in the absence of lactose]." *Anales Espanoles de Pediatria* 48 (5): 515-21.

Moya, M., and E. Domenech. 1982. "Role of calcium-phosphate ratio of milk formulae on calcium balance in low weight infants during the first three days of life." *Pediatric Research* 16 (8): 675-81.

Murthy, H. B., S. K. Reddy, M. Swaminathan, and V. Subrahmanyan. 1955. "The metabolism of nitrogen, calcium and phosphorus in undernourished children. 1. Adaptation to low intake of calories, protein, calcium and phosphorus." *British Journal of Nutrition* 9 (3): 203-9.

Nanulescu, M., and R. Manasia. 1984. "Hypercalcemia due to intoxication with vitamin D." *Pediatrie* 33 (3): 279-284.

Natarajan, C. K., M. J. Sankar, R. Agarwal, O. T. Pratap, V. Jain, N. Gupta, A. K. Gupta, A. K. Deorari, V. K. Paul, and V. Sreenivas. 2014. "Trial of daily vitamin D supplementation in preterm infants." *Pediatrics* 133 (3): e628-34. https://doi.org/https://dx.doi.org/10.1542/peds.2012-3395.

Navas-Nazario, A., L. Fangyong, V. Northrup, P. Weiss, D. Cole, T. Carpenter, and A. Bazzy-Asaad. 2011. "Effect of vitamin D binding protein (DBP) genotype on the development of asthma in young children." *American Journal of Respiratory and Critical Care Medicine* 183 (1).

Norris, J. M., H. S. Lee, B. Frederiksen, I. Erlund, U. Uusitalo, J. Yang, A. Lernmark, O. Simell, J. Toppari, M. Rewers, A. G. Ziegler, J. X. She, S. Onengut-Gumuscu, W. M. Chen, S. S. Rich, J.

Sundvall, B. Akolkar, J. Krischer, S. M. Virtanen, W. Hagopian, and Teddy Study Group. 2018. "Plasma 25-Hydroxyvitamin D Concentration and Risk of Islet Autoimmunity." *Diabetes* 67 (1): 146-154. https://doi.org/https://dx.doi.org/10.2337/db17-0802.

O'Brien, K. O., and S. A. Abrams. 1994. "Effects of development on techniques for calcium stable isotope studies in children." *Biological Mass Spectrometry* 23 (6): 357-61.

Ohlund, Inger, Torbjorn Lind, Olle Hernell, Sven-Arne Silfverdal, and Pia Karlsland Akeson. 2017. "Increased vitamin D intake differentiated according to skin color is needed to meet requirements in young Swedish children during winter: a double-blind randomized clinical trial." *American Journal of Clinical Nutrition* 106 (1): 105-112. https://doi.org/10.3945/ajcn.116.147108.

Oliveri, B., H. Cassinelli, C. Mautalen, and M. Ayala. 1996. "Vitamin D prophylaxis in children with a single dose of 150000 IU of vitamin D." *European Journal of Clinical Nutrition* 50 (12): 807-810.

Otto-Buczkowska, E., and F. Kokot. 1982. "[Hypercalcemia after oral administration of preventive doses of vitamin D]." *Polski Tygodnik Lekarski* 37 (32): 943-5.

Ouzzani, Mourad, Hossam Hammady, Zbys Fedorowicz, and Ahmed Elmagarmid. 2016. "Rayyan—a web and mobile app for systematic reviews." *Systematic Reviews* 5 (1): 210. https://doi.org/10.1186/s13643-016-0384-4.

Park, M. J., R. Namgung, D. H. Kim, and R. C. Tsang. 1998. "Bone mineral content is not reduced despite low vitamin D status in breast milk-fed infants versus cow's milk based formula-fed infants." *J Pediatr* 132 (4): 641-5. https://doi.org/10.1016/s0022-3476(98)70353-1.

Pittard, W. B., 3rd, K. M. Geddes, T. C. Hulsey, and B. W. Hollis. 1991. "How much vitamin D for neonates?" *American Journal of Diseases of Children* 145 (10): 1147-9.

Pludowski, P., M. Jaworski, A. Niemirska, M. Litwin, M. Szalecki, E. Karczmarewicz, and J. Michalkiewicz. 2014. "Vitamin D status, body composition and hypertensive target organ damage in primary hypertension." *Journal of Steroid Biochemistry & Molecular Biology* 144 Pt A: 180-4. https://doi.org/https://dx.doi.org/10.1016/j.jsbmb.2013.10.026.

Ponnapakkam, T., E. Bradford, and R. Gensure. 2010. "A treatment trial of vitamin D supplementation in breast-fed infants: universal supplementation is not necessary for rickets prevention in Southern Louisiana." *Clinical Pediatrics* 49 (11): 1053-60. https://doi.org/https://dx.doi.org/10.1177/0009922810376320.

Rooze, S., F. Mathieu, W. Claus, T. Yangzom, D. Yangzom, P. Goyens, and V. de Maertelaer. 2016. "Effect of calcium and vitamin D on growth, rickets and Kashin-Beck disease in 0- to 5-year-old children in a rural area of central Tibet." *Tropical Medicine & International Health* 21 (6): 768-75. https://doi.org/https://dx.doi.org/10.1111/tmi.12706.

Rosendahl, J., A. S. Pelkonen, O. Helve, H. Hauta-Alus, E. Holmlund-Suila, S. Valkama, M. Enlund-Cerullo, H. Viljakainen, T. Hytinantti, O. Makitie, S. Andersson, and M. J. Makela. 2019. "High-Dose Vitamin D Supplementation Does Not Prevent Allergic Sensitization of Infants." *Journal of Pediatrics* 209: 139-145.e1. https://doi.org/https://dx.doi.org/10.1016/j.jpeds.2019.02.021.

Rosendahl, J., S. Valkama, E. Holmlund-Suila, M. Enlund-Cerullo, H. Hauta-Alus, O. Helve, T. Hytinantti, E. Levalahti, E. Kajantie, H. Viljakainen, O. Makitie, and S. Andersson. 2018. "Effect of Higher vs Standard Dosage of Vitamin D3 Supplementation on Bone Strength and Infection in Healthy

Infants: A Randomized Clinical Trial." *JAMA Pediatrics* 172 (7): 646-654. https://doi.org/https://dx.doi.org/10.1001/jamapediatrics.2018.0602.

Ross, S. G. 1952. "Vitamin D intoxication in infancy; a report of four cases." *Journal of Pediatrics* 41 (6): 815-22.

Rowicka, G., J. Ambroszkiewicz, M. Strucinska, H. Dylag, and M. Golebiowska-Wawrzyniak. 2012. "[The evaluation of selected parameters of calcium and phosphorus metabolism in children with cow's milk allergy]." *Medycyna Wieku Rozwojowego* 16 (2): 109-16.

Rueter, K., A. P. Jones, A. Siafarikas, E. M. Lim, N. Bear, P. S. Noakes, S. L. Prescott, and D. J. Palmer. 2019. "Direct infant UV light exposure is associated with eczema and immune development." *Journal of Allergy & Clinical Immunology* 143 (3): 1012-1020.e2. https://doi.org/https://dx.doi.org/10.1016/j.jaci.2018.08.037.

Russell, R., M. Chung, E. M. Balk, S. Atkinson, E. L. Giovannucci, S. Ip, A. H. Lichtenstein, S. T. Mayne, G. Raman, A. C. Ross, T. A. Trikalinos, K. P. West, Jr., and J. Lau. 2009. "Opportunities and challenges in conducting systematic reviews to support the development of nutrient reference values: vitamin A as an example." *Am J Clin Nutr* 89 (3): 728-33. https://doi.org/10.3945/ajcn.2008.27154.

Santos, F., D. Suarez, S. Malaga, and M. Crespo. 1987. "Idiopathic hypercalciuria in children: pathophysiologic considerations of renal and absorptive subtypes." *Journal of Pediatrics* 110 (2): 238-43.

Sezer, R. G., T. Guran, C. Paketci, L. P. Seren, A. Bozaykut, and A. Bereket. 2012. "Comparison of oral alendronate versus prednisolone in treatment of infants with vitamin D intoxication." *Acta Paediatrica* 101 (3): e122-5. https://doi.org/https://dx.doi.org/10.1111/j.1651-2227.2011.02497.x.

Shajari, Ahmad, Mehrdad Shakiba, Forough Nourani, Maryam Zaki, and Maryam Kheirandish. 2009. "Urinary Calcium/Creatinin Ratio with Different Dosages of Vitamin D3 Prophylaxis in Infants." *Iranian Journal of Pediatrics* 19 (2): 159-163.

Shakiba, M., A. Pahloosye, M. Mirouliaei, and Z. Islami. 2014. "Comparison of two regimens of vitamin D supplementation for vitamin D-deficient neonates." *Singapore Medical Journal* 55 (5): 266-70.

Shakiba, M., S. Sadr, Z. Nefei, H. Mozaffari-Khosravi, M. H. Lotfi, and M. H. Bemanian. 2010. "Combination of bolus dose vitamin D with routine vaccination in infants: a randomised trial." *Singapore Medical Journal* 51 (5): 440-445.

Sharma, A. K., S. Gallo, C. A. Vanstone, S. Agellon, M. L'Abbe, A. Khamessan, K. Comeau, H. A. Weiler, and C. Rodd. 2016. "Parathyroid hormone-ionized calcium dynamics over the first year of life." *Journal of Pediatric Endocrinology & Metabolism* 29 (6): 709-14. https://doi.org/https://dx.doi.org/10.1515/jpem-2015-0240.

Siafarikas, A., H. Piazena, U. Feister, M. K. Bulsara, H. Meffert, and V. Hesse. 2011. "Randomised controlled trial analysing supplementation with 250 versus 500 units of vitamin D3, sun exposure and surrounding factors in breastfed infants." *Archives of Disease in Childhood* 96 (1): 91-5. https://doi.org/https://dx.doi.org/10.1136/adc.2009.178301.

Simpson, M., H. Brady, X. Yin, J. Seifert, K. Barriga, M. Hoffman, T. Bugawan, A. E. Baron, R. J. Sokol, G. Eisenbarth, H. Erlich, M. Rewers, and J. M. Norris. 2011. "No association of vitamin D intake or 25-hydroxyvitamin D levels in childhood with risk of islet autoimmunity and type 1 diabetes: the

Diabetes Autoimmunity Study in the Young (DAISY)." *Diabetologia* 54 (11): 2779-88. https://doi.org/https://dx.doi.org/10.1007/s00125-011-2278-2.

Singh, H., A. Maria, A. Shukla, N. Sharma, K. Mani, and M. K. Das. 2018. "Relation of Vitamin D supplementation, upto six months of age with total and bone specifc alkaline phosphatase: a randomised control trial." *Journal of clinical and diagnostic research* 12 (10): SC01-SC05.

Specht, I. O., J. Janbek, F. Thorsteinsdottir, P. Frederiksen, and B. L. Heitmann. 2019. "Neonatal vitamin D levels and cognitive ability in young adulthood." *European Journal of Nutrition*.

Specker, B., and T. Binkley. 2003. "Randomized trial of physical activity and calcium supplementation on bone mineral content in 3- to 5-year-old children." *Journal of Bone & Mineral Research* 18 (5): 885-92.

Specker, B. L., A. Beck, H. Kalkwarf, and M. Ho. 1997. "Randomized trial of varying mineral intake on total body bone mineral accretion during the first year of life." *Pediatrics* 99 (6): E12.

Stellinga-Boelen, A. A., P. A. Wiegersma, H. Storm, C. M. Bijleveld, and H. J. Verkade. 2007. "Vitamin D levels in children of asylum seekers in The Netherlands in relation to season and dietary intake." *European Journal of Pediatrics* 166 (3): 201-6.

Sterne, J. A. C., J. Savovic, M. J. Page, R. G. Elbers, N. S. Blencowe, I. Boutron, C. J. Cates, H. Y. Cheng, M. S. Corbett, S. M. Eldridge, J. R. Emberson, M. A. Hernan, S. Hopewell, A. Hrobjartsson, D. R. Junqueira, P. Juni, J. J. Kirkham, T. Lasserson, T. Li, A. McAleenan, B. C. Reeves, S. Shepperd, I. Shrier, L. A. Stewart, K. Tilling, I. R. White, P. F. Whiting, and J. P. T. Higgins. 2019. "RoB 2: a revised tool for assessing risk of bias in randomised trials." *BMJ* 366: l4898. https://doi.org/10.1136/bmj.l4898.

Sudfeld, C. R., C. Duggan, S. Aboud, R. Kupka, K. P. Manji, R. Kisenge, and W. W. Fawzi. 2015. "Vitamin D status is associated with mortality, morbidity, and growth failure among a prospective cohort of HIV-infected and HIV-exposed Tanzanian infants." *Journal of Nutrition* 145 (1): 121-7. https://doi.org/https://dx.doi.org/10.3945/jn.114.201566.

Sutton, A., R. H. Mole, and D. Barltrop. 1977. "Urinary and faecal excretion of marker calcium (46Ca) by low birthweight infants." *Archives of Disease in Childhood* 52 (1): 50-55.

Talaat, I. M., N. M. Kamal, H. A. Alghamdi, A. A. Alharthi, and M. A. Alshahrani. 2016. "A randomized clinical trial comparing 3 different replacement regimens of vitamin D in clinically asymptomatic pediatrics and adolescents with vitamin D insufficiency." *Italian Journal of Pediatrics* 42 (1): 106.

Thacher, T. D., P. R. Fischer, C. O. Isichei, A. I. Zoakah, and J. M. Pettifor. 2012. "Prevention of nutritional rickets in Nigerian children with dietary calcium supplementation." *Bone* 50 (5): 1074-80. https://doi.org/10.1016/j.bone.2012.02.010.

Thorsen, S. U., C. B. Pipper, M. Alberdi-Saugstrup, S. Nielsen, A. Cohen, M. Lundqvist, L. C. Thygesen, A. Ascherio, and J. Svensson. 2017. "No association between vitamin D levels around time of birth and later risk of developing oligo- and polyarticular juvenile idiopathic arthritis: a Danish case-cohort study." *Scandinavian Journal of Rheumatology* 46 (2): 104-111.

Tornhammar, P., P. Ueda, M. Hult, H. Simila, D. Eyles, and M. Norman. 2014. "Season of birth, neonatal vitamin D status, and cardiovascular disease risk at 35 y of age: a cohort study from Sweden." *American Journal of Clinical Nutrition* 99 (3): 472-8.

Trilok-Kumar, G., M. Kaur, A. M. Rehman, H. Arora, M. M. Rajput, R. Chugh, A. Kurpad, H. S. Sachdev, and S. Filteau. 2015. "Effects of vitamin D supplementation in infancy on growth, bone parameters, body composition and gross motor development at age 3-6 years: follow-up of a randomized controlled trial." *International Journal of Epidemiology* 44 (3): 894-905. https://doi.org/https://dx.doi.org/10.1093/ije/dyv116.

Umaretiya, P. J., T. D. Thacher, P. R. Fischer, S. S. Cha, and J. M. Pettifor. 2013. "Bone mineral density in Nigerian children after discontinuation of calcium supplementation." *Bone* 55 (1): 64-8. https://doi.org/https://dx.doi.org/10.1016/j.bone.2013.03.017.

Ünal, E., Y. Koksal, S. Keles, H. Artac, I. Reisli, and M. Energin. 2007. "Iatrogenic vitamin D intoxiciation in infancy: Three cases." *Marmara Medical Journal* 20 (1): 47-51.

Valkama, S., E. Holmlund-Suila, M. Enlund-Cerullo, J. Rosendahl, H. Hauta-Alus, O. Helve, T. Hytinantti, H. Viljakainen, S. Andersson, and O. Makitie. 2017. "No Severe Hypercalcemia with Daily Vitamin D3 Supplementation of up to 30 micro g during the First Year of Life." *Hormone research in paediatrics* 88 (2): 147-154. https://doi.org/https://dx.doi.org/10.1159/000477298.

van Stuijvenberg, M. E., J. Nel, S. E. Schoeman, C. J. Lombard, L. M. du Plessis, and M. A. Dhansay. 2015. "Low intake of calcium and vitamin D, but not zinc, iron or vitamin A, is associated with stunting in 2- to 5-year-old children." *Nutrition* 31 (6): 841-6.

Vanstone, M. B., S. E. Oberfield, L. Shader, L. Ardeshirpour, and T. O. Carpenter. 2012. "Hypercalcemia in children receiving pharmacologic doses of vitamin D." *Pediatrics* 129 (4): e1060-3.

Videhult, F. K., I. Öhlund, O. Hernell, and C. E. West. 2016. "Body mass but not Vitamin D status is associated with bone mineral content and density in young school children in northern Sweden." *Food and Nutrition Research* 60.

Wadia, Ushma, Wayne Soon, Paola Chivers, Aesen Thambiran, David Burgner, Sarah Cherian, and Aris Siafarikas. 2018. "Randomised Controlled Trial Comparing Daily Versus Depot Vitamin D3 Therapy in 0-16-Year-Old Newly Settled Refugees in Western Australia Over a Period of 40 Weeks." *Nutrients* 10 (3). https://doi.org/10.3390/nu10030348.

Wells, G, B Shea, D O'Connell, J Peterson, V Welch, M Losos, and P Tugwell. 2013. The Newcastle-Ottawa Scale (NOS) for assessing the quality of nonrandomised studies in meta-analyses. Accessed 6/27/2020.

Wicklow, B., S. Gallo, A. Majnemer, C. Vanstone, K. Comeau, G. Jones, M. L'Abbe, A. Khamessan, A. Sharma, H. Weiler, and C. Rodd. 2016. "Impact of Vitamin D Supplementation on Gross Motor Development of Healthy Term Infants: A Randomized Dose-Response Trial." *Physical & Occupational Therapy in Pediatrics* 36 (3): 330-42. https://doi.org/https://dx.doi.org/10.3109/01942638.2015.1050150.

World Health Organization, . 2020. "FAO/WHO nutrient requirements for children aged 0–36 months.". https://www.who.int/nutrition/topics/nutrient-requirements-children-overview/en/.

Wu, D. M., X. Wen, X. R. Han, S. Wang, Y. J. Wang, M. Shen, S. H. Fan, J. Zhuang, M. Q. Li, B. Hu, C. H. Sun, Y. X. Bao, J. Yan, J. Lu, and Y. L. Zheng. 2018. "Relationship Between Neonatal Vitamin D at Birth and Risk of Autism Spectrum Disorders: the NBSIB Study." *Journal of Bone & Mineral Research* 33 (3): 458-466. https://doi.org/https://dx.doi.org/10.1002/jbmr.3326.

Yetley, E. A., A. J. MacFarlane, L. S. Greene-Finestone, C. Garza, J. D. Ard, S. A. Atkinson, D. M. Bier, A. L. Carriquiry, W. R. Harlan, D. Hattis, J. C. King, D. Krewski, D. L. O'Connor, R. L. Prentice, J. V. Rodricks, and G. A. Wells. 2017. "Options for basing Dietary Reference Intakes (DRIs) on chronic disease endpoints: report from a joint US-/Canadian-sponsored working group." *Am J Clin Nutr* 105 (1): 249S-285S. https://doi.org/10.3945/ajcn.116.139097.

Zeghoud, F., H. Benmekhbi, N. Djeghri, and M. Garabedian. 1994. "VITAMIN-D PROPHYLAXIS DURING INFANCY COMPARISON OF THE LONG-TERM EFFECTS OF 3 INTERMITTENT DOSES (15-MG, 5-MG, OR 2.5-MG) ON 25-HYDROXYVITAMIN-D CONCENTRATIONS." *American Journal of Clinical Nutrition* 60 (3): 393-396.

Zeghoud, F., C. Vervel, H. Guillozo, O. WalrantDebray, H. Boutignon, and M. Garabedian. 1997. "Subclinical vitamin D deficiency in neonates: Definition and response to vitamin D supplements." *American Journal of Clinical Nutrition* 65 (3): 771-778.

Zhang, H., S. Liu, Y. Si, S. Zhang, Y. Tian, Y. Liu, H. Li, and Z. Zhu. 2019. "Natural sunlight plus vitamin D supplementation ameliorate delayed early motor development in newborn infants from maternal perinatal depression." *Journal of Affective Disorders* 257: 241-249.

Zhou, J., J. Du, L. Huang, Y. Wang, Y. Shi, and H. Lin. 2018. "Preventive Effects of Vitamin D on Seasonal Influenza A in Infants: A Multicenter, Randomized, Open, Controlled Clinical Trial." *Pediatric Infectious Disease Journal* 37 (8): 749-754. https://doi.org/https://dx.doi.org/10.1097/INF.0000000000001890.

Ziegler, E. E., S. E. Nelson, and J. M. Jeter. 2014. "Vitamin D supplementation of breastfed infants: a randomized dose-response trial." *Pediatric Research* 76 (2): 177-83. https://doi.org/https://dx.doi.org/10.1038/pr.2014.76.
